# Chemoselective carbene insertion into the N−H bonds of NH_3_·H_2_O

**DOI:** 10.1038/s41467-022-35394-z

**Published:** 2022-12-10

**Authors:** Zhaohong Liu, Yong Yang, Qingmin Song, Linxuan Li, Giuseppe Zanoni, Shaopeng Liu, Meng Xiang, Edward A. Anderson, Xihe Bi

**Affiliations:** 1 Department of Chemistry, Northeast Normal University, 130024 Changchun, China; 2 Department of Chemistry, University of Pavia, Viale Taramelli 12, 27100 Pavia, Italy; 3 Chemistry Research Laboratory, University of Oxford, 12 Mansfield Road, Oxford, OX1 3TA UK; 4 State Key Laboratory of Elemento-Organic Chemistry, Nankai University, 300071 Tianjin, China

**Keywords:** Synthetic chemistry methodology, Catalytic mechanisms, Organometallic chemistry

## Abstract

The conversion of inexpensive aqueous ammonia (NH_3_·H_2_O) into value-added primary amines by N−H insertion persists as a longstanding challenge in chemistry because of the tendency of Lewis basic ammonia (NH_3_) to bind and inhibit metal catalysts. Herein, we report a chemoselective carbene N−H insertion of NH_3_·H_2_O using a Tp^Br3^Ag-catalyzed two-phase system. Coordination by a homoscorpionate Tp^Br3^ ligand renders silver compatible with NH_3_ and H_2_O and enables the generation of electrophilic silver carbene. Water promotes subsequent [1,2]-proton shift to generate N−H insertion products with high chemoselectivity. The result of the reaction is the coupling of an inorganic nitrogen source with either diazo compounds or *N-*triftosylhydrazones to produce useful primary amines. Further investigations elucidate the reaction mechanism and the origin of chemoselectivity.

## Abstract (web summary)

Converting low-cost inorganic chemicals into value-added organic chemicals is a longstanding goal in chemistry. Here the authors describe a silver-catalysed chemoselective carbene N−H insertion reaction, providing access to primary amines from aqueous ammonia and diazo compounds.

## Introduction

**Fig. 1:**
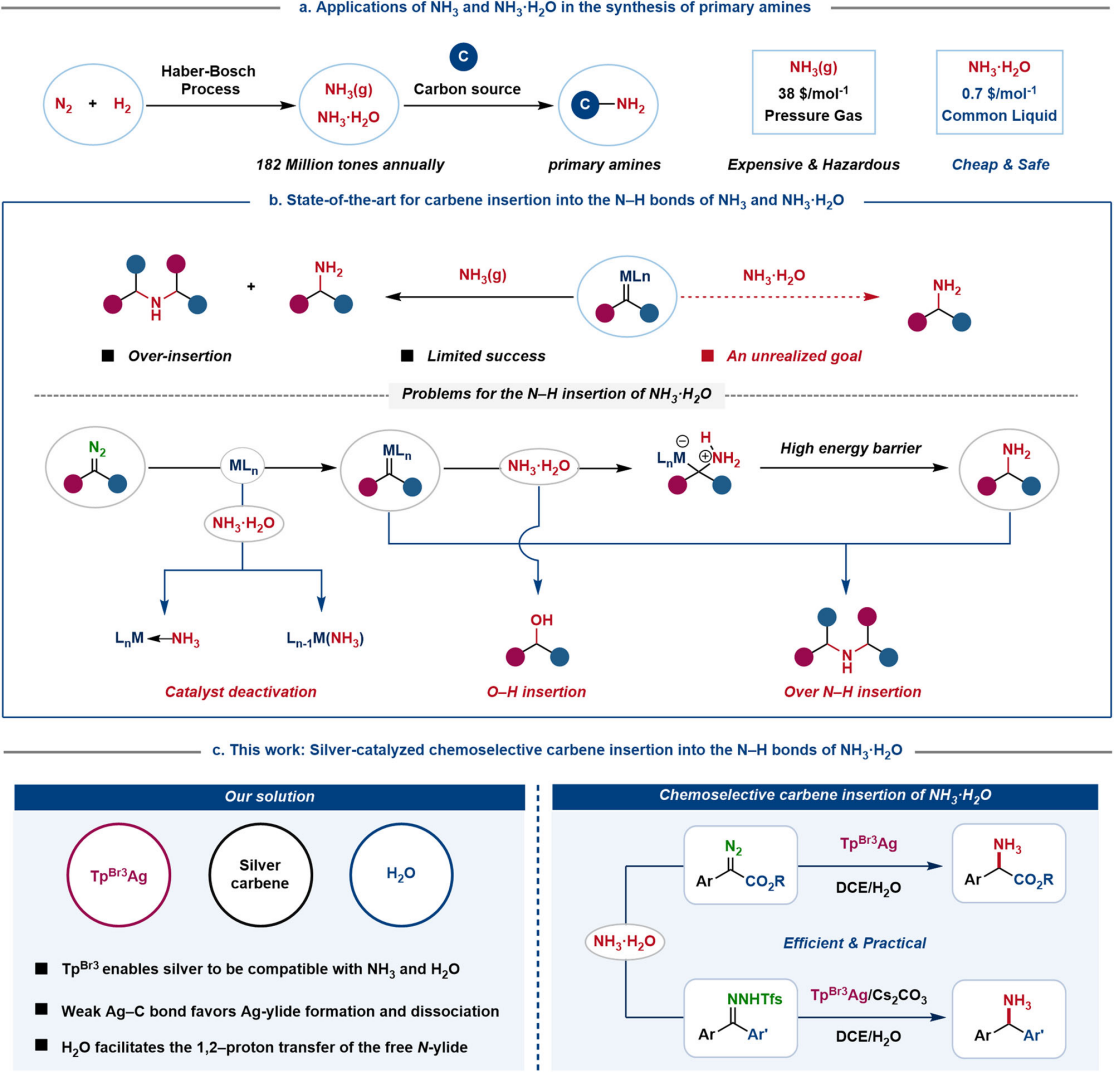
Direct route to primary amines from inorganic NH_3_ and NH_3_·H_2_O: strategies and challenges. **a** Strategies to primary amines using NH_3_ and NH_3_·H_2_O as nitrogen source. **b** Challenges of N−H insertion of NH_3_·H_2_O. **c** Tp^Br3^Ag-catalyzed two-phase system enables chemoselective carbene insertion into the N−H bonds of NH_3_·H_2_O. Tp, tris(pyrazolyl)borate; Ar, aryl; Tfs, 2-(trifluoromethyl)benzenesulfonyl.

Ammonia (NH_3_) is arguably the most readily available nitrogen feedstock, with an annual production over 182 million tons from elemental nitrogen and hydrogen via the Haber−Bosch process^1,2^. The conversion of inorganic NH_3_ or NH_3_·H_2_O into organic amines thus continues to attract the attention of academia and industry (Fig. 1a)^3–7^. Among those, direct catalytic syntheses of primary amines (-NH_2_) from NH_3_·H_2_O attracts special attention due to the cost and step economy^8–10^. Moreover, primary amines are not only commonly found in pharmaceuticals, natural products and agrochemicals^11–15^, but also could be readily derived to more complex nitrogen-containing compounds^16,17^. However, the development of such a process by transition-metal catalysis is hampered by the high strength of the N−H bond (107 kcal mol^−1^) and Lewis basicity of NH_3_, resulting in poisoning electrophilic metal catalysts^18,19^. The transition-metal-catalyzed N−H insertion reactions constitute a well-established strategy for C−N bond formation^20–29^, while the few known examples of N−H insertion with NH_3_ typically report a mixture of primary, secondary, and even tertiary amines^30,31^. Zhou and co-workers very recently disclosed a milestone progress of asymmetrical carbene insertion into the N−H bonds of NH_3_ (in MTBE) by the cooperative action of copper complexes and chiral hydrogen-bond donor, albeit the scope of this chemistry was limited to alkyl diazoesters^32^. NH_3_·H_2_O is a cheaper and safer nitrogen source than pressurized liquid NH_3_, not requiring special equipment for transportation, storage, and handling, thus at a reduced cost per mole of NH_3_ equivalents^10,33^. However, such a carbene insertion into the N−H bonds of NH_3_·H_2_O has not yet been achieved to date, presumably as the easy deactivation of transition-metal complexes by the formation of a stable Werner complex or ligand exchange with Lewis basic NH_3_ inhibits the generation of metal-carbene complexes^18,19,32^. Moreover, even if the desired metal carbenes were generated, competitive O−H insertion with water^34,35^ and multiple N−H insertion^30,31^ with initially formed primary amines still exist (Fig. 1b).

Herein, we report a promising solution to this long-term challenge by Ag-catalyzed two-phase reaction system for the chemoselective carbene insertion into the N−H bonds of NH_3_·H_2_O using a variety of diazo compounds^24,25^ and *N-*triftosylhydrazones^36–40^ as carbene precursors (Fig. 1c). Our work stems from the following initial assumptions. First, coordination by a homoscorpionate Tp ligand protects the silver center, which enables it to react with diazo compound to generate a silver carbene even in the presence of NH_3_^32^ and water^41–43^. Second, water acts as a proton-transporter to facilitate the 1,2-proton shift of *N*-ylide, thereby ensuring the selectivity of carbene N−H insertion^43–46^. This process represents an efficient and practical chemoselective carbene insertion into the N−H bonds of NH_3_·H_2_O, enabling the efficient synthesis of primary amines, including diaryl methylamines and α-amino acid esters, valuable building blocks for pharmaceuticals and agrochemicals^11–15^.

## Results and discussion

### Investigation of reaction conditions

Initial screening studies were conducted on the N−H insertion of NH_3_·H_2_O with methyl phenyldiazoacetate **1** (Table 1). After evaluating multiple reaction parameters, the desired N–H insertion product **2** was obtained under optimized conditions in 92% yield (in 12 h from treating **1** with 8.0 equiv of NH_3_·H_2_O at 60 ^o^C in 1,2-dichloroethane (DCE) in the presence of 10 mol % Tp^Br3^Ag(thf))^47^, along with 5% of O–H insertion product **3** (Table 1, entry 1). When Tp^Br3^Ag(thf) was replaced with Tp^(CF3)2^Ag(thf)^48^, the yield increased to 40% (entry 7). All other tested transition-metal catalysts failed to deliver even a trace amount of the insertion product **2**, instead leading to products of side reactions of carbene or diazo compounds (entries 8–12).

**Table 1:** Optimization of N–H insertion of NH_3_·H_2_O with diazo compound


Entry	Cat.	Solvent	*T* (°C)	2 Yield^a^	3 Yield^b^
1	Tp^Br3^Ag(thf) (10 mol %)	DCE	60	92%	<5%
2	Tp^Br3^Ag(thf) (10 mol %)	DCM	60	57%	<5%
3	Tp^Br3^Ag(thf) (10 mol %)	THF	60	N.D.	N.D.
4	Tp^Br3^Ag(thf) (10 mol %)	1,4-Dioxane	60	N.D.	N.D.
5	Tp^Br3^Ag(thf) (10 mol %)	Toluene	60	23%	N.D.
6	Tp^Br3^Ag(thf) (10 mol %)	CHCl_3_	60	75%	<5%
7	Tp^(CF3)2^Ag(thf) (10 mol %)	CHCl_3_	60	40%	N.D.
8	AgOAc (10 mol %)	CHCl_3_	60	N.D.	N.D.
9	Fe(TPP)Cl (10 mol %)	CHCl_3_	60	N.D.	N.D.
10	Rh_2_(OAc)_4_ (5 mol %)	CHCl_3_	60	N.D.	N.D.
11	Cu(OAc)_2_ (10 mol %)	CHCl_3_	60	N.D.	N.D.
12	Pd(OAc)_2_ (10 mol %)	CHCl_3_	60	Trace	N.D.
13	Tp^Br3^Ag(thf) (10 mol %)	DCE	80	75%	N.D.

Reaction conditions: methyl phenyldiazoacetate **1** (0.3 mmol), NH_3_**·**H_2_O (2.4 mmol, 8.0 equiv) and Cat. (5–10 mol %) in solvent (4.0 mL) was stirred at 60 °C under nitrogen atmosphere for 12 h.

*N.D*. not detected, *DCE* 1,2-dichlorethane, *DCM* dichloromethane, *TPP* tetraphenylporphyrin.

^a^Isolated yield.

^b^Yield was determined by ^1^H NMR with dibromomethane as the internal standard.

### Substrate scope

**Fig. 2:**
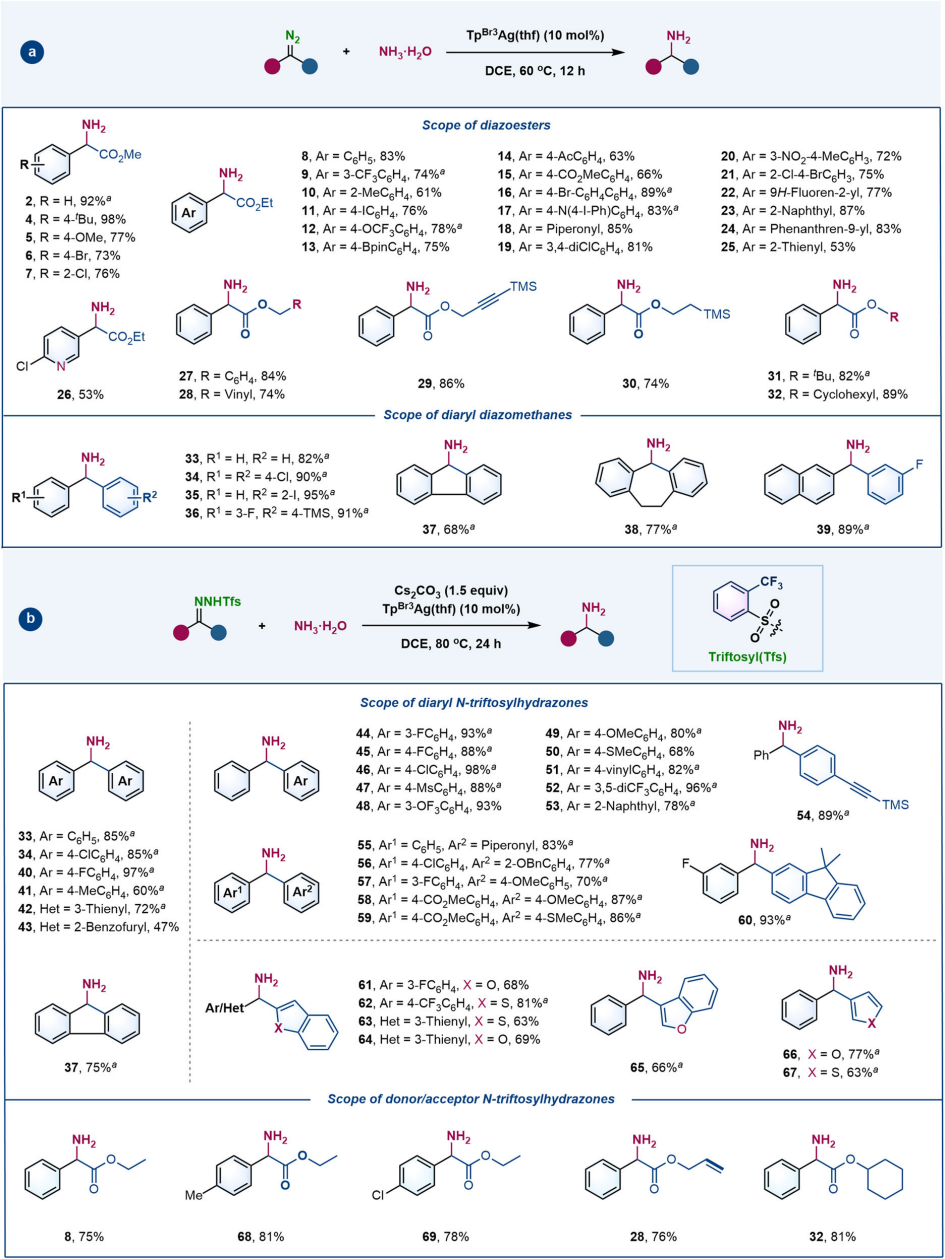
Reaction scope of carbene insertion into the N−H bonds of NH_3_·H_2_O. **a** Silver-catalyzed N–H insertion of NH_3_·H_2_O with diazo compounds. **b** Silver-catalyzed N–H insertion of NH_3_·H_2_O with *N*-triftosylhydrazones. Reactions were carried out on a 0.3-mmol scale. Isolated yield reported. ^*a*^Isolated as hydrochloride salt. ^*t*^Bu *tert*-butyl, Bpin boronic acid pinacol ester, Ac acetyl, Ms methanesulfonyl, Ph phenyl, TMS trimethylsilyl, Bn benzyl.

The scope of substrate diazo compounds was then explored under the optimized conditions (Fig. 2a). The tested aryl and heteroaryl diazoacetates resulted in the desired α-amino acid esters (**4**–**26**) in 53–98% yield, regardless of electronic character or position of the substituents on the aromatic ring. In addition to the methyl and ethyl esters, benzyl (**27**), allyl (**28**), propargyl (**29**), 2-(trimethylsilyl)ethyl (**30**), *tert*-butyl (**31**), and cyclohexyl (**32**) phenyldiazoacetates also furnished the corresponding insertion products in good yield. The reaction is not limited to donor/acceptor diazo compounds, and can be successfully expanded to donor/donor diazo compounds. A broad range of symmetric and unsymmetric diaryl diazomethanes afforded the target diaryl methylamines (**33**–**39**) in good to excellent yield. Unfortunately, alkyl diazo compounds are a current limitation, undergoing competing 1,2-H shift to form alkenes.

As the toxicity and potential explosivity of high-energy diazo compounds prevent scale-up of this transformation^24,25^, we explored the possible use of easily prepared, bench-stable *N-*sulfonylhydrazones as carbene precursors^36,37,49–51^. Another round of optimization studies with diphenyl *N*-triftosylhydrazone as model substrate resulted in the desired diarylmethylamine (**33**) being obtained in 85% yield, when the reaction was performed in DCE at 80 ^o^C with Cs_2_CO_3_ as the base (see Supplementary Table 2 for details). In contrast, *N*-tosylhydrazone proved unsuitable substrate, as the same product was obtained in a much lower 33% yield under identical conditions (entry 11, Supplementary Table 2). As shown in Fig. 2b, under these modified conditions, various diaryl *N*-triftosylhydrazones provided the desired diaryl methylamines in good to excellent yield along with a trace amount of O−H insertion products (**33**, **34**, **37**, and **40**–**60**)—the electronic and steric effects did not impact the reaction efficiency and chemoselectivity. Heteroaryl methylamines, including benzofuryl (**61**, **64**, and **65**), benzothienyl (**62** and **63**), furyl (**66**) and thienyl (**67**) methylamines, were analogously isolated in moderate to good yield from the corresponding *N*-triftosylhydrazones. Donor/acceptor *N*-triftosylhydrazones could also undergo effective N−H insertion reactions (**8**, **28**, **32**, **68**, and **69**). Notably, this in situ diazo generation protocol proved to be equally effective as the corresponding diazo-initiated reactions (**8**, **28**, **32**–**34**, and **37**). The reaction exhibited excellent functional group tolerance of a range of functional groups, including halogen, aniline, ketone, ester, nitro, olefin, alkyne, *tert*-butyl, methoxy, trifluoromethyl and trimethylsilyl groups, predominantly providing the desired N−H insertion products along with only trace amounts of O−H insertion products.

### Gram-scale synthesis and synthetic applications

**Fig. 3:**
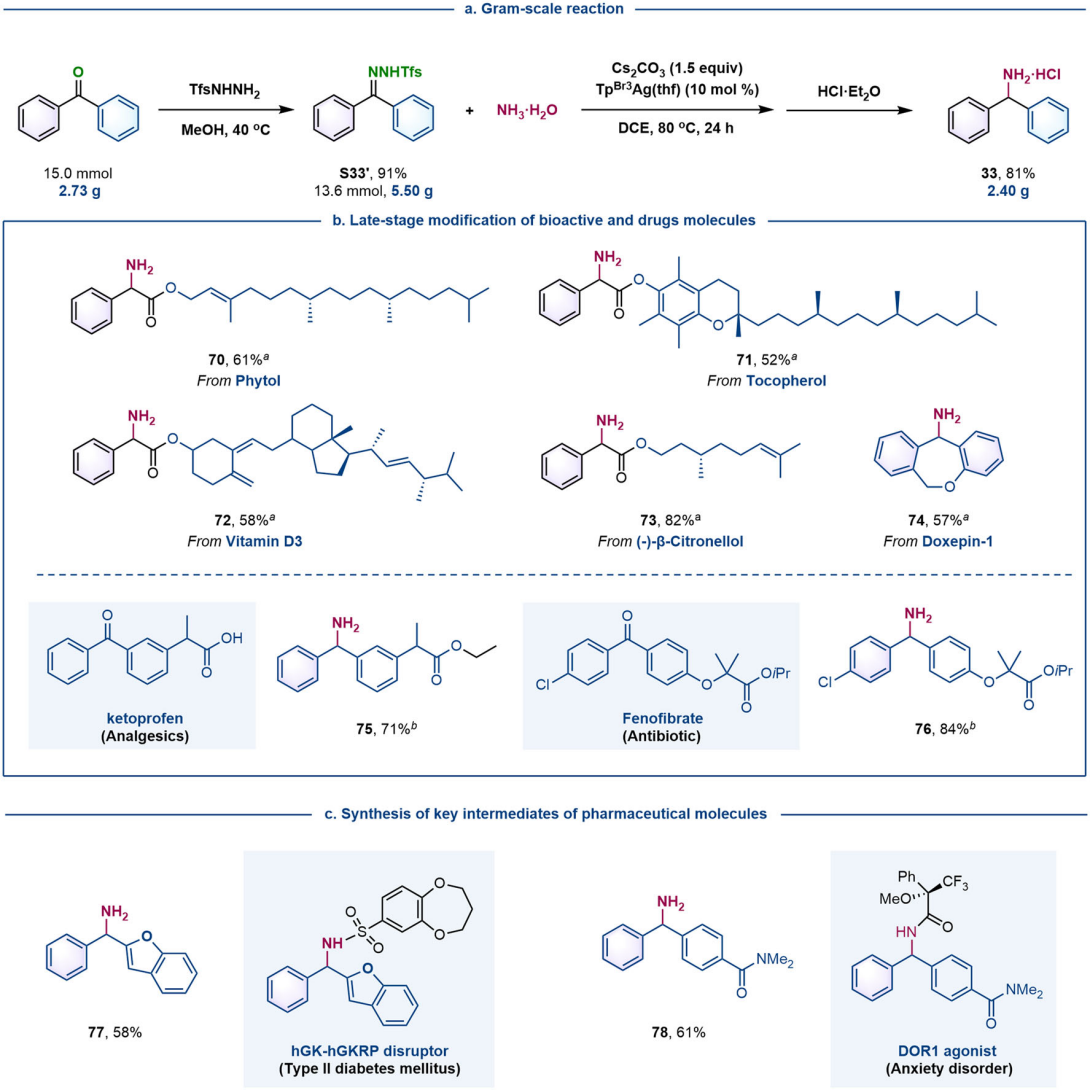
Synthetic applications. **a** Gram-scale reaction. **b** Late-stage transformation of bioactive and drug molecules. ^a^Started from the corresponding diazo compound. ^*b*^Started from the corresponding *N*-triftosylhydrazone. **c** Shortened synthesis of key intermediates of drug molecule. Detailed reaction conditions are provided in the supplementary materials.

When the reaction of NH_3_·H_2_O with diphenyl *N*-triftosylhydrazone was conducted on a gram-scale, hydrochloride **33**·HCl was obtained pure, without chromatography, in a two-step 74% yield from diphenyl ketone (Fig. 3a). Our silver-catalyzed protocol could also be applied to late-stage modification of bioactive molecules (Fig. 3b). For instance, natural products containing a hydroxy group, such as phytol, tocopherol, vitamin D3, and (-)-β-citronellol, were first converted into the corresponding phenyldiazoacetates, then subjected to the optimized reaction conditions, affording the corresponding α-amino acid esters in moderate to good yield (**70**–**73**). Similarly, doxepin-1, a precursor of doxopin hydrochloride (a psychotropic drug) was easily converted into diarylmethylamine **74** in moderate yield through the corresponding diazo compound. The diarylmethylamine moiety is present in many agrochemical and pharmaceutical compounds, for example, the hydrochloride salts of cetirizine, hydroxyzine, and meclizine possessing the structural motif of **46**^12^, and the antimigraine drug lomerizine containing the structural motif of **40**^13^. Ketoprofen (an oral analgesic) and fenofibrate (an oral drug used to lower cholesterol levels) were converted to the corresponding primary amines **75** and **76**, respectively, via the corresponding *N*-triftosylhydrazones. Compounds **77** and **78**, intermediates in the synthesis of pharmaceutical compounds (GK-GKRP disruptor^14^ and DOR1^15^ agonist, respectively), could also be obtained by this N−H insertion of NH_3_·H_2_O with *N-*triftosylhydrazones, showcasing the potential of our protocol for applications in drug discovery (Fig. 3c).

### Mechanistic investigations

**Fig. 4:**
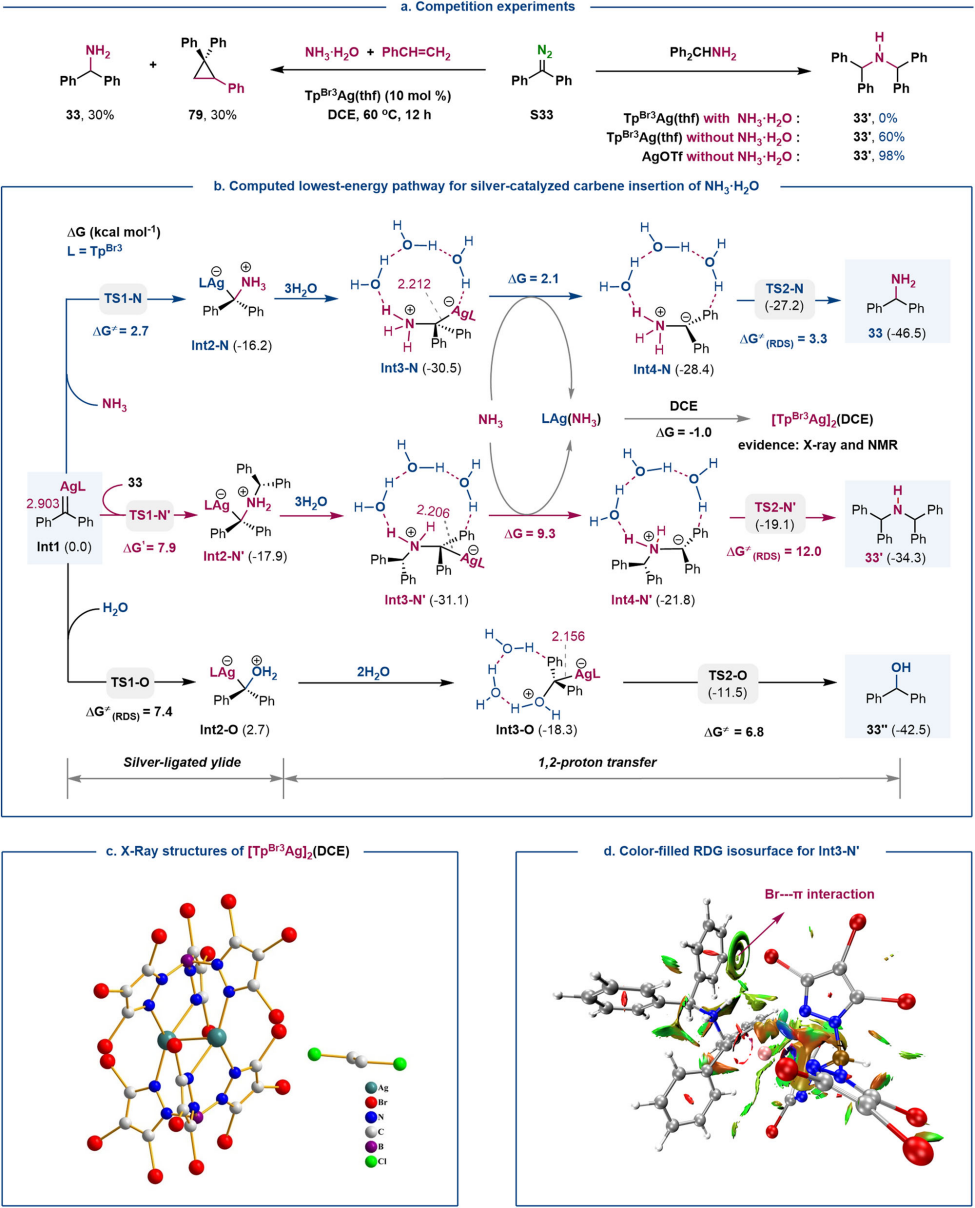
Experimental and DFT computational insights into the mechanism. **a** Control experiments. **b** Computed lowest-energy pathways for silver-catalyzed carbene insertion of NH_3_, H_2_O, and primary amine **33**. The relative free energies present in parentheses and the (RDS) energy barriers (kcal mol^–1^) were calculated at the SMD(DCE)-M06/[6-311 + G(d,p)-SDD(Ag/Br)] level. All distances are in angstroms. **c** X-Ray structure of [Tp^Br3^Ag]_2_(DCE). **d** The Color-filled RDG isosurface for **Int3-N’**(isovalue set to 0.5): the water molecules are omitted for clarity: (blue) areas of attraction (covalent bonding); (green) vdW interactions; (red) areas of repulsion (steric and ring effects).

To gain insights into the reaction mechanism and the origin of chemoselectivity, we performed control experiments and density functional theory (DFT) calculations. A competition experiment between NH_3_·H_2_O and styrene resulted in a mixture of insertion product **33** and cyclopropane **79**, suggesting that the ylide intermediate may be generated from silver carbene (Fig. 4a)^21^. DFT calculations were carried out to explore the possible reaction pathways of silver carbene **Int1** with NH_3_, diphenylmethylamine **33**, and H_2_O, respectively (see Supplementary Figs. 3–8 for details). Computed lowest-energy pathways for single N−H, double N−H, and O−H insertion are shown in Fig. 4b. The calculated Ag−C distance in **Int1** of 2.093 Å points to a weak silver carbene Ag=C bond with an electrophilic carbenic carbon, which favors nucleophilic attack of X−H bonds onto **Int1**^21,36^. DFT calculations show that the free energy activation for the formation of silver-ligated *N*-ylide intermediate **Int2-N** from NH_3_ (2.7 kcal mol^−1^) is much lower than that for the formation of **Int2-N’** from primary amine **33** and **Int2-O** from H_2_O (7.9 and 7.4 kcal mol^−1^, respectively). These results are consistent with the nucleophilicity of NH_3_, primary amine **33**, and H_2_O, respectively (for the comparison of their nucleophilicity by natural population analysis, see Supplementary Fig. 9).

The weak Ag−C bond (2.212 Å) in the silver-ligated ylide favors the ligand exchange of NH_3_ with **Int3-N** to release a free ylide **Int4-N** and silver complex **LAg(NH**_**3**_**)**. The water-assisted 1,2-proton shift from free *N*-ylide **Int4-N** opens the lowest-energy route to the N−H insertion of NH_3_ (Supplementary Figs. 3 and 4)^43–46^. Because of the weak silver coordination, **LAg(NH**_**3**_**)** can react with the solvent (DCE) to generate reactive **[Tp**^**Br3**^**Ag]**_**2**_**(DCE)**, thus suggesting **LAg(NH**_**3**_**)** differs from the generally stable metal−ammine complexes^8,9^. The structure of **[Tp**^**Br3**^**Ag]**_**2**_**(DCE)** was confirmed by X-ray and NMR on a sample of isolated material (Fig. 4c), which proved as effective as Tp^Br3^Ag(thf) in the N−H insertion of NH_3_·H_2_O (see Supplementary Fig. 2). These results are consistent with our initial hypothesis that the homoscorpionate Tp^Br3^ ligand renders the silver catalyst compatible with NH_3_, thus achieving the challenging N−H insertion of NH_3_·H_2_O.

Albeit similar to that of NH_3_, the reaction pathway for the N−H insertion of diphenylmethylamine **33** (Supplementary Fig. 6) encounters a higher energy barrier for the cleavage of the Ag−C bond in ylide **Int3-N’** (9.3 vs. 2.1 kcal mol^−1^), owing to the stronger Br···π weak interaction between the phenyl and Tp^Br3^ ligand in **Int3-N’**, as determined by color-filled reduced density gradient (RDG, Fig. 4d and Supplementary Fig. 9)^52,53^. This hypothesis was confirmed by the reaction between primary amine **33** and diphenyldiazomethane **S33** without NH_3_·H_2_O, whereby in the absence of the Br···π weak interaction AgOTf led to a higher product yield (60% vs. 98%, Fig. 4a). The dual N−H insertion proceeds through an activation barrier of 12.0 kcal mol^−1^, which is 8.7 kcal mol^−1^ higher than that for the N−H insertion of NH_3_, thus accounting for the absence of over N−H insertion product **33’** under the optimized reaction condition. We also investigated the reaction of the silver carbene **Int1** with water (Fig. S4). The Ag−C distance in **Int3-O** is shorter than that in **Int3-N** (2.156 vs. 2.212 Å), which favors O−H insertion as the lowest-energy route for the 1,2-proton shift assisted by two molecules of water, with an activation free energy of 6.8 kcal mol^−1^. Therefore, the formation of ylide **Int2-O**, with an activation free energy of 7.4 kcal mol^−1^, is rate-limiting step for the O−H insertion.

The overall values for activation free energy for the turnover limiting-steps (1,2-H shift or *O-*ylide formation) of the N−H, dual N−H, and O−H insertion paths were 3.3, 12.0, and 7.4 kcal mol^−1^, respectively, consistent with the experimental 10:0:1 chemoselectivity in the respective products. The weak interaction between the silver center and the carbenic carbon is beneficial to the formation and dissociation of silver-ligated ylide, which favors N−H insertion of NH_3_ over O−H insertion. On the other hand, the significant Br···π weak interaction between the phenyl group in the substrate and the bulky Tp^Br3^ ligand inhibits the dual N−H insertion.

In this work, we disclose an efficient methodology for the chemoselective carbene insertion into the N−H bonds of NH_3_·H_2_O by a silver-catalyzed two-phase system, providing access to value-added primary amines from industrial inorganic nitrogen source. Considering the easy availability and environmental safety of NH_3_·H_2_O and the significance of the obtained nitrogen-containing compounds, the discovery of the compatibility between the metal-carbene catalyst with the NH_3_·H_2_O adduct may have broader applications in transition-metal-catalyzed N−H activation of NH_3_·H_2_O.

## Methods

### General procedure for silver-catalyzed N–H insertion of NH_3_·H_2_O with diazo compounds

A Schlenk tube was charged with Tp^Br3^Ag(thf) (33.0 mg, 0.03 mmol, 10 mol %). The tube was evacuated and filled with N_2_ for three times. A mixture of NH_3_·H_2_O (308 μL, 28–30% wt%, 0.6 mmol, 8.0 equiv) and DCE (2 mL) was injected into the tube by syringe, followed by DCE (2 mL) solution of diazo compound (0.3 mmol, 1.0 equiv). The resulting mixture was stirred at 60 °C for 12 h in the dark. When the reaction was completed, the crude reaction mixture was allowed to reach room temperature and concentrated in vacuo and purified by column chromatography on silica gel (petroleum ether/EtOAc) to afford the corresponding N–H insertion product.

### General procedure for silver-catalyzed N–H insertion of NH_3_·H_2_O with *N*-triftosylhydrazones

A Schlenk tube was charged with Tp^Br3^Ag(thf) (33.0 mg, 0.03 mmol, 10 mol%) and Cs_2_CO_3_ (146.6 mg, 0.45 mmol, 1.5 equiv). The tube was evacuated and filled with N_2_ for three times. A mixture of NH_3_·H_2_O (308 μL, 28–30% wt%, 0.6 mmol, 8.0 equiv) and DCE (2 mL) was injected into the tube by syringe, followed by DCE (2 mL) solution of *N*-triftosylhydrazone (0.3 mmol, 1.0 equiv). The resulting mixture was stirred at 80 °C for 24 h in the dark. When the reaction was completed, the crude reaction mixture was allowed to reach room temperature and concentrated in vacuo and purified by column chromatography on silica gel (petroleum ether/EtOAc) to afford the corresponding N–H insertion product.

## Supplementary information

Supplementary Information

Peer Review File

Description of Additional Supplementary Files

Supplementary Data 1

## Data Availability

The X-ray crystallographic coordinates for structures reported in this study have been deposited at the Cambridge Crystallographic Data Centre (CCDC), under deposition number 2166126. These data can be obtained free of charge from The Cambridge Crystallographic Data Centre via www.ccdc.cam.ac.uk/data_request/cif. The data that support the findings of this study are available within the paper and its Supplementary Information and Supplementary Data files. Raw data are available from the corresponding author on request. Materials and methods, computational studies, experimental procedures, characterization data, ^1^H, ^13^C, ^19^F NMR spectra, and mass spectrometry data are available in the Supplementary Information. Supplementary Data File 1 contains the cartesian coordinates and energies for the computed structures.
