# Supplementary material for: Chemoselective carbene insertion into the N−H bonds of NH3·H2O
Source: Nat Commun. 2022 Dec 10;13:7649. doi: 10.1038/s41467-022-35394-z (PMC9741638; doi:10.1038/s41467-022-35394-z)
Supplement: Supplementary file 1 — Supplementary Information [file 41467_2022_35394_MOESM1_ESM.pdf]

Supplementary Information for

Chemoselective carbene insertion into the N–H bonds of $NH_3 \cdot H_2O$

Zhaohong Liu,[1,+] Yong Yang,[1,+] Qingmin Song,[1] Linxuan Li,[1] Giuseppe Zanoni,[2] Shaopeng Liu[1], Meng Xiang,[1] Edward A. Anderson[3] and Xihe Bi[1,4]*

[1] Department of Chemistry, Northeast Normal University, Changchun 130024, China

[2] Department of Chemistry, University of Pavia; Viale Taramelli 12, 27100, Pavia, Italy

[3] Chemistry Research Laboratory, University of Oxford, 12 Mansfield Road, Oxford, OX1 3TA, U.K.

[4] State Key Laboratory of Elemento-Organic Chemistry, Nankai University, Tianjin 300071, China

+ These authors contributed equally to this work: Zhaohong Liu and Yong Yang

*Corresponding author: Xihe Bi, E-mail: bixh507@nenu.edu.cn.

Table of Contents

Supplementary Methods S2

1. General Information S2

2. Optimization of Reaction Conditions S3

3. Typical Procedure For N–H Bond Insertion of $NH_3 \cdot H_2O$ And Analytical Data S4

4. Preparation of Starting Materials S28

4.1 Synthesis and analytical data of new diazo compounds S28

4.2 Synthesis and analytical data of *N*-triftosylhydrazones S32

5. Synthetic Applications S36

5.1 Gram-scale experiments S36

5.2 Late-stage modification of bioactive and drugs molecules S37

6. Mechanistic Studies S40

6.1 Control experiments S40

6.2 Computational studies S43

7. NMR Spectra of Products S50

Supplementary References S136

Supplementary Methods

1. General Information

1.1 Equipment and methods

The products were purified by column chromatography over silica gel. NMR spectra were recorded on a Brüker Advance 600 (^{1}H: 600 MHz, ^{13}C:151 MHz) and Brüker Advance 500 (^{1}H: 500 MHz; ^{13}C: 126 MHz) at ambient temperature. The following residual solvent signals were used as references for ^{1}H and ^{13}C NMR spectra: $CDCl_3$, δ_H 0.00 ppm (relative to TMS), δ_C 77.00 ppm; DMSO-d_6, δ_H 2.50 ppm, δ_C 40.00 ppm. ^{19}F NMR chemical shifts were determined relative to $CFCl_3$ as outside standard and low field is positive. The following abbreviations were used to explain the multiplicities: s = singlet, d = doublet, t = triplet, q = quartet, qi = quintet, m = multiplet, br = broad. Thin layer chromatographic (TLC) analysis was performed with glass-backed silica gel plates, visualizing with UV light (254 nm) and/or staining with aqueous $KMnO_4$ stain. High-resolution mass spectra (HRMS) were recorded on Magnetic Sector High Resolution Gas Chromatography-Mass Spectra and Q Exactive Focus (Thermal) by using ESI method. IR was recorded on Nicolet 6700 spectrometer. Melting points were determined with XRC-1 and are uncorrected.

1.2 Solvents, reagents and catalysts

All solvents and reagents were purchased from commercial sources and used without purification unless otherwise mentioned.

Tp^{Br3}Ag(thf) was prepared according to literature procedure with slight variation.[1] A 500 mL Schlenk tube was charged with pre-sublimated 1*H*–3,4,5-tribromopyrazole (12.19 g, 40.0 mmol) and $TlBH_4$ (2.19 g, 10.0 mmol). The tube was fitted with a reflux condenser and a nitrogen balloon (to balance the increased pressure of hydrogen production during the reaction). The reaction was heated at 180 °C for 2 h, then the temperature was raised to 200 °C and the reaction was continued for 2 h. After cooling to room temperature, unreacted pyrazole was removed by vacuum sublimation (150 °C, 2 mbar) to give $TlTp^{Br3}$ as a white solid. AgOTf (1.29 g, 5.0 mmol) was added to a solution of $TlTp^{Br3}$ (5.64 g, 5.0 mmol) in acetone. After stirring in the dark for 20 h, a white solid precipitated from the initially colorless solution. The solid was filtered off and dried under vacuum to give complex $[Tp^{Br3}Ag]_2 \cdot CH_3COCH_3$, which was stirred in freshly distilled tetrahydrofuran (100 mL) for 30 min in the dark. The solvent was removed under reduced pressure, and white solid Tp^{Br3}Ag(thf) was obtained after vacuum drying.

$Tp^{(CF3)2}$Ag(thf) was prepared according to literature procedure with slight variation.[2] AgOTf (514 mg, 2 mmol) was added to a THF solution of $NaTp^{(CF3)2}$ (1290 mg, 2.0 mmol). The resulted mixture was stirred for 20 h in the dark. The solvent was removed under reduced pressure, and residuum was extracted into hexane and filtered through Celite. The filtrate was evaporated under reduced pressure, and white solid $Tp^{(CF3)2}$Ag(thf) was obtained in 85% yield after vacuum drying.

2. Optimization of Reaction Conditions

Supplementary Table 1. Optimization of N–H insertion of $NH_3 \cdot H_2O$ with diazo compound.

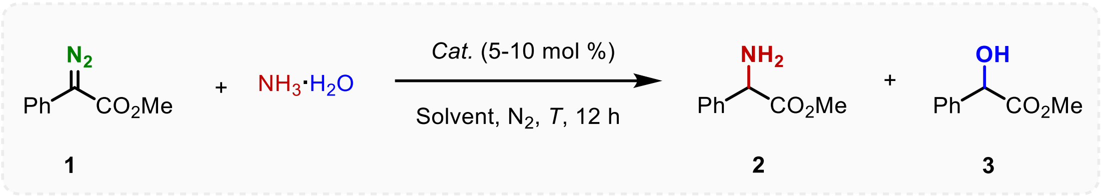

Entry	Cat.	Solvent	T (°C)	**2** Yield[a]	**3** Yield[b]
1	Fe(TPP)Cl (10 mol %)	$CHCl_3$	60	N.D.	N.D.
2	$Rh_2(OAc)_4$ (5 mol %)	$CHCl_3$	60	N.D.	N.D.
3	$Cu(OAc)_2$ (10 mol %)	$CHCl_3$	60	N.D.	N.D.
4	$Pd(OAc)_2$ (10 mol %)	$CHCl_3$	60	trace	N.D.
5	AgOAc (10 mol %)	$CHCl_3$	60	N.D.	N.D.
7	$Tp^{(CF3)2}$Ag(thf) (10 mol %)	$CHCl_3$	60	40%	N.D.
8	Tp^{Br3}Ag(thf) (10 mol %)	$CHCl_3$	60	75%	<5%
9	Tp^{Br3}Ag(thf) (10 mol %)	DCE	60	92%	<5%
10	Tp^{Br3}Ag(thf) (10 mol %)	DCM	60	57%	<5%
11	Tp^{Br3}Ag(thf) (10 mol %)	THF	60	N.D.	N.D.
12	Tp^{Br3}Ag(thf) (10 mol %)	1,4-dioxane	60	N.D.	N.D.
13	Tp^{Br3}Ag(thf) (10 mol %)	Toluene	60	23%	N.D.
14	Tp^{Br3}Ag(thf) (10 mol %)	DCE	40	trace	N.D.
15	Tp^{Br3}Ag(thf) (10 mol %)	DCE	80	75%	N.D.

Reaction conditions: methyl phenyldiazoacetate **1** (0.3 mmol), $NH_3 \cdot H_2O$ (2.4 mmol, 8.0 equiv) and Cat. (5-10 mol %) in solvent (4.0 mL) was stirred at 60 ℃ under nitrogen atmosphere for 12 h. [a]Isolated yield. [b]Yield was determined by ^1H NMR with dibromomethane as the internal standard. *N.D.*, not detected.

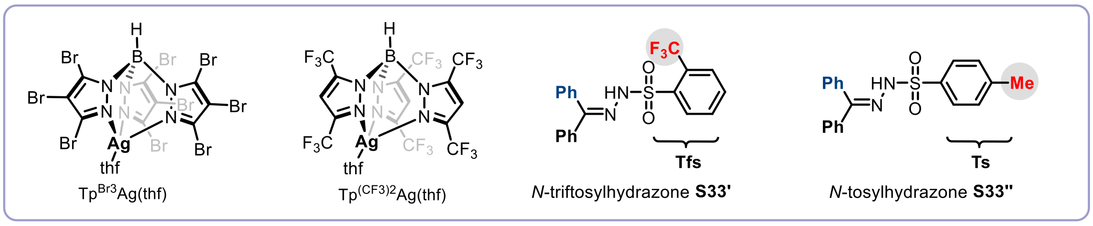

Supplementary Table 2. Optimization of N–H insertion of $NH_3 \cdot H_2O$ with *N*-sulfonylhydrazone.

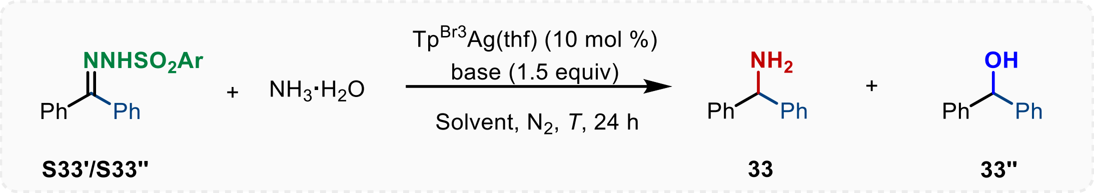

Entry	Hydrazone	Base	Solvent	T (°C)	**33** Yield[a]	**33''** Yield[b]
1	S33'	$NaOCH_3$	DCE	80	N.D.	N.D.
2	S33'	Cs_2CO_3	DCE	80	85%	8%
3	S33'	K_2CO_3	DCE	80	62	7%
4	S33'	NaOH	DCE	80	26	5%
5	S33'	DIPEA	DCE	80	N.D.	N.D.
6	S33'	Cs_2CO_3	DCE	100	80	8%
7	S33'	Cs_2CO_3	DCE	60	60	6%
8	S33'	Cs_2CO_3	DCM	80	58	6%
9	S33'	Cs_2CO_3	$CHCl_3$	80	63	3%
10	S33'	Cs_2CO_3	Toluene	80	trace	trace
11	S33''	Cs_2CO_3	DCE	80	33	18%

Reaction conditions: *N*-sulfonylhydrazone **S33'** or **S33''** (0.3 mmol), $NH_3 \cdot H_2O$ (2.4 mmol, 8.0 equiv), base (0.45 mmol, 1.5 equiv) and $Tp^{Br3}Ag(thf)$ (10 mol %) in solvent (3.0 mL) was stirred at 80 ℃ for 24 h under nitrogen atmosphere. [a]Isolated yield. [b]Yield was determined by ^1H NMR with dibromomethane as the internal standard. *N.D.*, not detected.

3. Typical Procedure for N–H Insertion of $NH_3 \cdot H_2O$ and Analytical Data

Silver-catalyzed N–H insertion of $NH_3 \cdot H_2O$ with diazo compounds

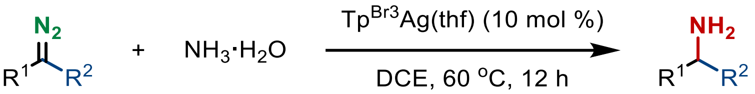

General Procedure A: A Schlenk tube was charged with $Tp^{Br3}Ag(thf)$ (33.0 mg, 0.03 mmol, 10 mol %). The tube was evacuated and filled with N_2 for three times. A mixture of $NH_3 \cdot H_2O$ (308 μL, 28%-30% wt%, 0.6 mmol, 8.0 equiv) and DCE (2 mL) was injected into the tube by syringe, followed by DCE (2 mL) solution of diazo compound (0.3 mmol, 1.0 equiv). The resulting mixture was stirred at 60 °C for 12 h in the dark. When the reaction was completed, the crude reaction mixture was allowed to reach room temperature and concentrated in vacuo and purified by column chromatography on silica gel (petroleum ether/EtOAc) to afford the corresponding N–H insertion product.

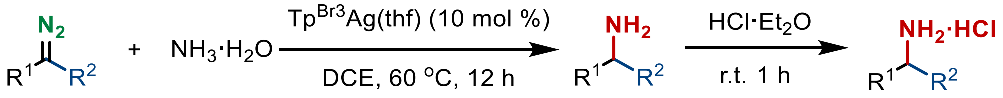

General Procedure B: A Schlenk tube was charged with $Tp^{Br3}Ag(thf)$ (33.0 mg, 0.03 mmol, 10 mol %). The tube was evacuated and filled with N_2 for three times. A mixture of $NH_3{\cdot}H_2O$ (308 μL, 28%-30% wt%, 0.6 mmol, 8.0 equiv) and DCE (2 mL) was injected into the tube by syringe, followed by DCE (2 mL) solution of diazo compound (0.3 mmol, 1.0 equiv). The resulting mixture was stirred at 60 °C for 12 h in the dark. When the reaction was completed, the crude reaction mixture was allowed to reach room temperature and concentrated in vacuo. The residue was treated with Et_2O solution of HCl and stirred for 1 h at room temperature to to precipitate a white solid, which was filtered, washed with cold ether and dried under vacuo to yield hydrochloride of N–H insertion product.

Silver-catalyzed N–H insertion of $NH_3{\cdot}H_2O$ with *N*-triftosylhydrazones

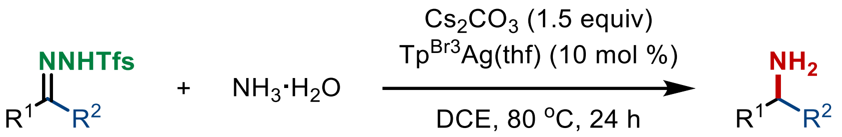

General Procedure C: A Schlenk tube was charged with $Tp^{Br3}Ag(thf)$ (33.0 mg, 0.03 mmol, 10 mol %) and Cs_2CO_3 (146.6 mg, 0.45 mmol, 1.5 equiv). The tube was evacuated and filled with N_2 for three times. A mixture of $NH_3{\cdot}H_2O$ (308 μL, 28%-30% wt%, 0.6 mmol, 8.0 equiv) and DCE (2 mL) was injected into the tube by syringe, followed by DCE (2 mL) solution of *N*-triftosylhydrazone (0.3 mmol, 1.0 equiv). The resulting mixture was stirred at 80 °C for 24 h in the dark. When the reaction was completed, the crude reaction mixture was allowed to reach room temperature and concentrated in vacuo and purified by column chromatography on silica gel (petroleum ether/EtOAc) to afford the corresponding N–H insertion product.

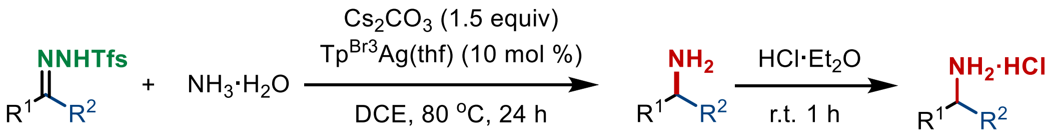

General Procedure D: A Schlenk tube was charged with $Tp^{Br3}Ag(thf)$ (33.0 mg, 0.03 mmol, 10 mol %) and Cs_2CO_3 (146.6 mg, 0.45 mmol, 1.5 equiv). The tube was evacuated and filled with N_2 for three times. A mixture of $NH_3{\cdot}H_2O$ (308 μL, 28%-30% wt%, 0.6 mmol, 8.0 equiv) and DCE (2 mL) was injected into the tube by syringe, followed by DCE (2 mL) solution of *N*-triftosylhydrazone (0.3 mmol, 1.0 equiv). The resulting mixture was stirred at 80 °C for 24 h in the dark. When the reaction was completed, the crude reaction mixture was allowed to reach room temperature and concentrated in vacuo. The residue was treated with Et_2O solution of HCl and stirred for 1 h at room temperature to precipitate a white solid, which was filtered, washed with cold ether and dried under vacuo to yield hydrochloride of N–H insertion product.

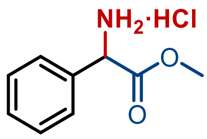

2

(2) methyl 2-amino-2-phenylacetate hydrochloride

Prepared according to **General Procedure B** using methyl 2-diazo-2-phenylacetate **1** (0.3 mmol, 25.9 mg) and $NH_3·H_2O$ (308 µL, 2.4 mmol) afforded compound **2** (55.7 mg, 92% yield) as a white solid (mp: 168-170 °C). **^{1}H NMR** (500 MHz, DMSO) δ 8.97 (s, 3H), 7.51-7.46 (m, 5H), 5.29 (d, *J* = 1.5 Hz, 1H), 3.72 (s, 3H). **^{13}C NMR** (126 MHz, DMSO) δ 169.4, 133.2, 130.0, 129.5, 128.7, 55.8, 53.7. **HRMS** (ESI) m/z calcd for $C_9H_{12}NO_2$ $[M+H]^+$ 166.0863, Found: 166.0862.

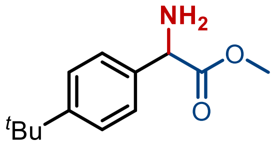

4

(4) methyl 2-amino-2-(4-(tert-butyl)phenyl)acetate

Prepared according to **General Procedure A** using methyl 2-(4-(*tert*-butyl) phenyl)-2-diazoacetate **S4** (0.3 mmol, 69.7 mg) and $NH_3·H_2O$ (308 µL, 2.4 mmol) afforded compound **4** (65.1 mg, 98% yield) as a colourless oil. **^{1}H NMR** (600 MHz, $CDCl_3$) δ 7.39-7.35 (m, 2H), 7.31-7.28 (m, 2H), 4.60 (s, 1H), 3.70 (s, 3H), 2.02 (s, 2H), 1.31 (s, 9H). **^{13}C NMR** (126 MHz, $CDCl_3$) δ 174.6, 151.0, 137.3, 126.5, 125.8, 58.5, 52.4, 34.6, 31.3. **HRMS** (ESI) m/z calcd for $C_{13}H_{19}NNaO_2$ $[M+Na]^+$ 244.1308. Found: 244.1314.

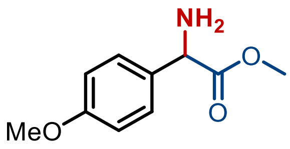

5

(5) methyl 2-amino-2-(4-methoxyphenyl)acetate

Prepared according to **General Procedure A** using methyl 2-diazo-2-(4-methoxyphenyl) acetate **S5** (0.3 mmol, 61.9 mg) and $NH_3·H_2O$ (308 µL, 2.4 mmol) afforded compound **5** (45.1 mg, 77% yield) as a colourless oil. **^{1}H NMR** (500 MHz, $CDCl_3$) δ 7.28 (d, *J* = 8.7 Hz, 2H), 6.87 (d, *J* = 8.7 Hz, 2H), 4.60 (s, 1H), 3.78 (s, 3H), 3.69 (s, 3H), 2.29 (s, 2H). **^{13}C NMR** (151 MHz, $CDCl_3$) δ 174.5, 159.5, 132.0, 128.0, 114.2, 58.1, 55.3, 52.5. **HRMS** (ESI) m/z calcd for $C_{10}H_{13}NNaO_3$ $[M+Na]^+$ 218.0788, Found: 218.0793.

Spectroscopic data are in agreement with those reported in the literature.[3]

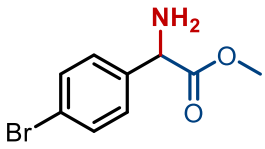

6

(6) methyl 2-amino-2-(4-bromophenyl)acetate

Prepared according to **General Procedure A** using methyl 2-(4-bromophenyl)-2-diazoacetate **S6** (0.3 mmol, 76.5 mg) and $NH_3·H_2O$ (308 µL, 2.4 mmol) afforded compound **6** (53.5 mg, 73% yield) as a

colourless oil. **^{1}H NMR** (500 MHz, $CDCl_3$) δ 7.47 (d, *J* = 8.5 Hz, 2H), 7.26 (d, *J* = 8.5 Hz, 2H), 4.58 (s, 1H), 3.69 (s, 3H), 1.86 (s, 2H). **^{13}C NMR** (126 MHz, $CDCl_3$) δ 174.0, 139.2, 131.9, 128.6, 122.1, 58.1, 52.6. **HRMS** (ESI) m/z calcd for $C_9H_{10}BrNNaO_2$ $[M+Na]^+$ 265.9787, Found: 265.9777.

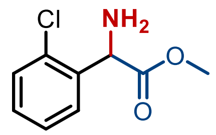

7

(7) methyl 2-amino-2-(2-chlorophenyl)acetate

Prepared according to **General procedure A** using methyl 2-(2-chlorophenyl)-2-diazoacetate **S7** (0.3 mmol, 63.2 mg) and $NH_3{\cdot}H_2O$ (308 μL, 2.4 mmol) afforded compound **7** (45.5 mg, 76% yield) as a colourless oil. **^{1}H NMR** (500 MHz, $CDCl_3$) δ 7.37 (dd, *J* = 7.5, 1.5 Hz, 1H), 7.33 (dd, *J* = 7.0, 2.0 Hz, 1H), 7.29-7.21 (m, 2H), 4.99 (s, 1H), 3.71 (s, 3H), 2.07 (s, 2H). **^{13}C NMR** (151 MHz, $CDCl_3$) δ 173.9, 138.2, 133.4, 130.0, 129.2, 128.5, 127.4, 56.1, 52.6. **HRMS** (ESI) m/z calcd for $C_9H_{10}ClNNaO_2$ $[M+Na]^+$ 222.0292, Found: 222.0295.

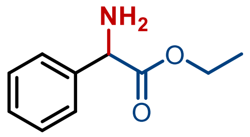

8

(8) ethyl 2-amino-2-phenylacetate

Prepared according to **General Procedure A** using ethyl 2-diazo-2-phenylacetate **S8** (0.3 mmol, 57.1 mg) and $NH_3{\cdot}H_2O$ (308 μL, 2.4 mmol) afforded compound **8** (44.6 mg, 83% yield) as a colourless oil.
Prepared according to **General Procedure C** using ethyl 2-phenyl-2-(2-((2-(trifluoromethyl) phenyl) sulfonyl) hydrazineylidene) acetate **S8'** (0.3 mmol, 120.1 mg) and $NH_3{\cdot}H_2O$ (308 μL, 2.4 mmol) afforded compound **8** (40.3 mg, 75% yield) as a colourless oil. **^{1}H NMR** (500 MHz, $CDCl_3$) δ 7.39-7.31 (m, 4H), 7.28 (t, *J* = 7.0 Hz, 1H), 4.58 (s, 1H), 4.22-4.07 (m, 2H), 2.18 (s, 2H), 1.19 (t, *J* = 7.5 Hz, 3H). **^{13}C NMR** (151 MHz, $CDCl_3$) δ 173.9, 140.3, 128.7, 127.9, 126.7, 61.2, 58.7, 14.0. **HRMS** (ESI) m/z calcd for $C_{10}H_{13}NNaO_2$ $[M+Na]^+$ 202.0838, Found: 202.0835.

Spectroscopic data are in agreement with those reported in the literature .[4]

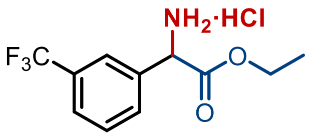

9

(9) ethyl 2-amino-2-(3-(trifluoromethyl) phenyl)acetate hydrochloride

Prepared according to **General Procedure B** using ethyl 2-diazo-2-(3-(trifluoromethyl) phenyl) acetate **S9** (0.3 mmol, 77.5 mg) and $NH_3{\cdot}H_2O$ (308 μL, 2.4 mmol) afforded compound **9** (63.0 mg, 74% yield)

as a white solid (mp: 171-172 ºC). **^{1}H NMR** (500 MHz, DMSO) δ 9.37 (s, 3H), 7.99 (s, 1H), 7.87 (d, *J* = 8.0 Hz, 1H), 7.81 (d, *J* = 7.5 Hz, 1H), 7.71 (t, *J* = 8.0 Hz, 1H), 5.45 (s, 1H), 4.25-4.13 (m, 2H), 1.12 (t, *J* = 7.0 Hz, 3H). **^{13}C NMR** (151 MHz, DMSO) δ 168.7, 134.9, 133.5, 131.0, 130.4 (q, *J* = 32.0 Hz), 127.1, (q, *J* = 3.0 Hz), 126.2 (q, *J* = 12.7 Hz), 124.8 (q, *J* = 272.7 Hz), 63.2, 55.7, 14.7. **^{19}F NMR** (564 MHz, DMSO) δ -62.67. **IR** (Film): 3446, 2712, 2248, 2124, 1734, 1025, 819, 757, 727 cm^{-1}.

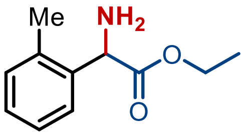

10

(10) ethyl 2-amino-2-(o-tolyl)acetate

Prepared according to **General Procedure A** using ethyl 2-diazo-2-(o-tolyl) acetate **S10** (0.3 mmol, 61.3 mg) and $NH_3{\cdot}H_2O$ (308 μL, 2.4 mmol) afforded compound **10** (35.4 mg, 61% yield) as a colourless oil. **^{1}H NMR** (500 MHz, $CDCl_3$) δ 7.26-7.23 (m, 1H), 7.21-7.17 (m, 3H), 4.82 (s, 1H), 4.24-4.09 (m, 2H), 2.44 (s, 3H), 2.10 (s, 2H), 1.20 (t, *J* = 7.0 Hz, 3H). **^{13}C NMR** (126 MHz, $CDCl_3$) δ 174.4, 138.7, 136.0, 130.8, 127.8, 126.5, 125.9, 61.3, 55.2, 19.3, 14.1. **IR** (Film): 3395, 2985, 2689, 1731, 903, 724 cm^{-1}.
Spectroscopic data are in agreement with those reported in the literature.[5]

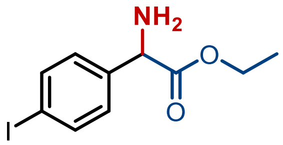

11

(11) ethyl 2-amino-2-(4-iodophenyl)acetate

Prepared according to **General Procedure A** using ethyl 2-diazo-2-(4-iodophenyl) acetate **S11** (0.3 mmol, 94.8 mg) and $NH_3{\cdot}H_2O$ (308 μL, 2.4 mmol) afforded compound **11** (69.6 mg, 76% yield) as a colourless oil. **^{1}H NMR** (500 MHz, $CDCl_3$) δ 7.67 (d, *J* = 8.0 Hz, 2H), 7.14 (d, *J* = 8.0 Hz, 2H), 4.55 (s, 1H), 4.24-4.08 (m, 2H), 2.05 (s, 2H), 1.20 (t, *J* = 7.0 Hz, 3H). **^{13}C NMR** (126 MHz, $CDCl_3$) δ 173.4, 139.9, 137.8, 128.8, 93.5, 61.5, 58.2, 14.0. **HRMS** (ESI) m/z calcd for $C_{10}H_{13}INO_2$ $[M+H]^+$ 305.9986, Found: 305.9981.

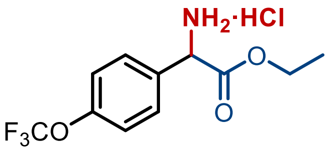

12

(12) ethyl 2-amino-2-(4-(trifluoromethoxy)phenyl)acetate hydrochloride

Prepared according to **General Procedure B** using ethyl 2-diazo-2-(4-(trifluoromethoxy) phenyl) acetate **S12** (0.3 mmol, 82.3 mg) and $NH_3{\cdot}H_2O$ (308 μL, 2.4 mmol) afforded compound **12** (70.1 mg, 78% yield) as a white solid (mp: 169-171 ºC). **^{1}H NMR** (600 MHz, DMSO) δ 9.26 (s, 3H), 7.68 (d, *J* =

8.4 Hz, 2H), 7.47 (d, J = 8.4 Hz, 2H), 5.34 (s, 1H), 4.24-4.13 (m, 2H), 1.14 (t, J = 7.2 Hz, 3H). **^{13}C NMR** (151 MHz, DMSO) δ 168.9, 149.9, 133.0, 131.6, 122.4, 120.9 (q, J = 257.0 Hz), 63.2, 55.5, 14.8. **^{19}F NMR** (564 MHz, DMSO) δ -56.8. **HRMS** (ESI) m/z calcd for $C_{11}H_{13}F_3NO_3$ $[M+H]^+$ 264.0842, Found: 264.0848.

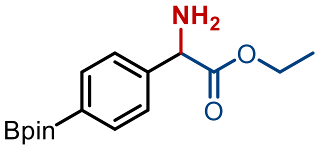

13

(13) ethyl 2-amino-2-(4-(4,4,5,5-tetramethyl-1,3,2-dioxaborolan-2-yl)henyl)acetate

Prepared according to **General Procedure A** using ethyl 2-diazo-2-(4-(4,4,5,5-tetramethyl-1,3,2-dioxaborolan-2-yl) henyl) acetate **S13** (0.3 mmol, 94.8 mg) and $NH_3 \cdot H_2O$ (308 μL, 2.4 mmol) afforded compound **13** (68.7 mg, 75% yield) as a colourless oil. **^{1}H NMR** (500 MHz, $CDCl_3$) δ 7.79 (d, J = 8.0 Hz, 2H), 7.37 (d, J = 8.0 Hz, 2H), 4.62 (s, 1H), 4.21-4.07 (m, 2H), 2.40 (s, 2H), 1.33 (s, 12H), 1.18 (t, J = 7.0 Hz, 3H). **^{13}C NMR** (126 MHz, $CDCl_3$) δ 173.5, 143.0, 135.2, 128.6, 126.1, 83.8, 61.4, 58.8, 24.8, 14.0. **HRMS** (ESI) m/z calcd for $C_{16}H_{24}BNNaO_4$ $[M+Na]^+$ 328.1694, Found: 328.1703.

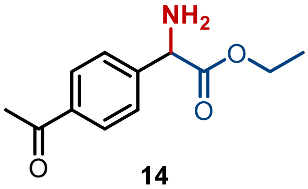

14

(14) ethyl 2-(4-acetylphenyl)-2-aminoacetate

Prepared according to **General Procedure A** using ethyl 2-(4-acetylphenyl)-2-diazoacetate **S14** (0.3 mmol, 69.7 mg) and $NH_3 \cdot H_2O$ (308 μL, 2.4 mmol) afforded compound **14** (43.8 mg, 66% yield) as a colourless oil. **^{1}H NMR** (500 MHz, $CDCl_3$) δ 7.91 (d, J = 8.5 Hz, 2H), 7.47 (d, J = 8.5 Hz, 2H), 4.69 (s, 1H), 4.22-4.08 (m, 2H), 2.57 (s, 3H), 2.35 (s, 2H), 1.18 (t, J = 7.0 Hz, 3H). **^{13}C NMR** (126 MHz, $CDCl_3$) δ 197.7, 173.3, 145.0, 136.8, 128.8, 127.2, 61.8, 58.5, 26.7, 14.1. **IR** (Film): 3378, 3053, 2985, 2672, 1264, 907, 727 cm^{-1}.

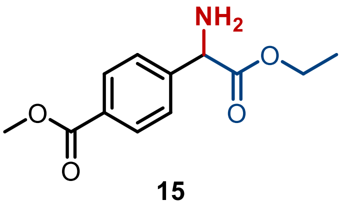

15

(15) methyl 4-(1-amino-2-ethoxy-2-oxoethyl)benzoate

Prepared according to **General Procedure A** using methyl 4-(1-diazo-2-ethoxy-2-oxoethyl) benzoate **S15** (0.3 mmol, 74.5 mg) and $NH_3 \cdot H_2O$ (308 μL, 2.4 mmol) afforded compound **15** (44.8 mg, 63% yield) as a colourless oil. **^{1}H NMR** (500 MHz, $CDCl_3$) δ 7.99 (d, J = 8.0 Hz, 2H), 7.44 (d, J = 8.5 Hz, 2H), 4.64 (s, 1H), 4.21-4.07 (m, 2H), 3.88 (s, 3H), 2.07 (s, 2H), 1.17 (t, J = 7.0 Hz, 3H). **^{13}C NMR**

(126 MHz, $CDCl_3$) δ 173.3, 166.6, 145.0, 129.9, 129.7, 126.8, 61.5, 58.4, 52.1, 14.0. **HRMS** (ESI) m/z calcd for $C_{12}H_{15}NNaO_4$ $[M+Na]^+$ 260.0893, Found: 260.0894.

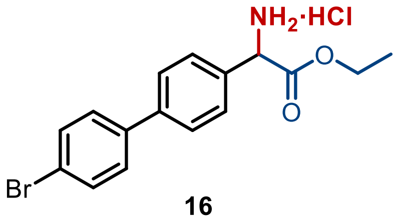

16

(16) ethyl 2-amino-2-(4'-bromo-[1,1'-biphenyl]-4-yl)acetate hydrochloride

Prepared according to **General Procedure B** using ethyl 2-(4'-bromo-[1,1'-biphenyl]-4-yl)-2-diazoacetate **S16** (0.3 mmol, 103.6 mg) and $NH_3\cdot H_2O$ (308 µL, 2.4 mmol) afforded compound **16** (99.0 mg, 89% yield) as a white solid (mp: 182-184 °C). **^{1}H NMR** (500 MHz, DMSO) δ 9.22 (s, 3H), 7.77 (d, *J* = 8.0 Hz, 2H), 7.66 (s, 4H), 7.62 (d, *J* = 8.5 Hz, 2H), 5.29 (s, 1H), 4.26-4.13 (m, 2H), 1.15 (t, *J* = 7.0 Hz, 3H). **^{13}C NMR** (126 MHz, DMSO) δ 169.2, 140.8, 139.2, 133.0, 132.8, 129.9, 129.8, 128.0, 122.4, 63.1, 56.0, 14.8. HRMS (ESI) m/z calcd for $C_{16}H_{17}BrNO_2$ $[M+H]^+$ 334.0437, Found: 334.0438.

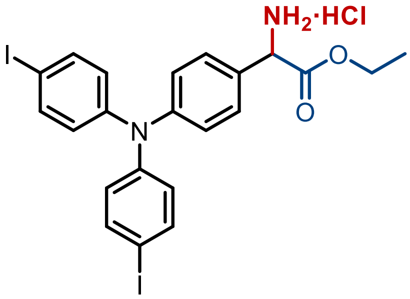

17

(17) ethyl 2-amino-2-(4-(*bis* 4-iodophenyl)mino)henyl)acetate hydrochloride

Prepared according to **General Procedure B** using ethyl 2-(4-(*bis*-4-iodophenyl)mino)henyl)-2-*di*-azoacetate **S17** (0.3 mmol, 182.8 mg) and $NH_3\cdot H_2O$ (308 µL, 2.4 mmol) afforded compound **17** (158.0 mg, 83% yield) as a white solid, m.p. 178-180 °C. **^{1}H NMR** (500 MHz, DMSO) δ 9.11 (s, 3H), 7.65 (d, *J* = 8.5 Hz, 4H), 7.44 (d, *J* = 8.5 Hz, 2H), 7.05 (d, *J* = 8.5 Hz, 2H), 6.81 (d, *J* = 8.5 Hz, 4H), 5.17 (s, 1H), 4.28-4.13 (m, 2H), 1.17 (t, *J* = 7.0 Hz, 3H). **^{13}C NMR** (126 MHz, DMSO) δ 169.2, 148.1, 147.1, 139.3, 130.7, 127.9, 127.3, 124.2, 88.6, 63.0, 55.8, 14.8. **IR** (Film): 3441, 3009, 2663, 2249, 1053,1024, 1005, 819, 757 cm^{-1}.

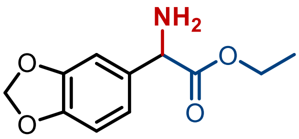

18

(18) ethyl 2-amino-2-(benzo[*d*][1,3]dioxol-5-yl)acetate

Prepared according to **General Procedure A** using ethyl 2-(benzo[*d*][1,3]dioxol-5-yl)-2-diazoacetate **S18** (0.3 mmol, 70.3 mg) and $NH_3 \cdot H_2O$ (308 μL, 2.4 mmol) afforded compound **18** (85 mg, 56.9% yield) as a colourless oil. **^{1}H NMR** (500 MHz, $CDCl_3$) δ 6.87 (d, *J* = 1.8 Hz, 1H), 6.83 (dd, *J* = 8.0, 1.8 Hz, 1H), 6.76 (d, *J* = 8.0 Hz, 1H), 5.94 (s, 2H), 4.50 (s, 1H), 4.23-4.08 (m, 2H), 1.94 (s, 2H), 1.21 (t, *J* = 7.2 Hz, 3H). **^{13}C NMR** (151 MHz, $CDCl_3$) δ 173.9, 147.9, 147.3, 134.3, 120.2, 108.3, 107.2, 101.1, 61.3, 58.5, 14.1. **HRMS** (ESI) m/z calcd for $C_{11}H_{14}NO_4$ $[M+H]^+$ 224.0917, Found: 224.0914.

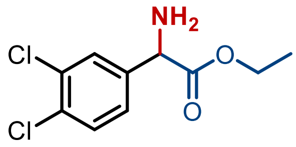

19

(19) ehyl 2-amino-2-(3,4-dichlorophenyl)acetate

Prepared according to **General Procedure A** using ethyl 2-diazo-2-(3,4-dichlorophenyl) acetate **S19** (0.3 mmol, 77.7 mg) and $NH_3 \cdot H_2O$ (308 μL, 2.4 mmol) afforded compound **19** (45.4 mg, 61% yield) as a colourless oil. **^{1}H NMR** (500 MHz, $CDCl_3$) δ 7.53 (d, *J* = 2.0 Hz, 1H), 7.42 (d, *J* = 8.5 Hz, 1H), 7.25 (dd, *J* = 8.5, 2.0 Hz, 1H), 4.57 (s, 1H), 4.25-4.11 (m, 2H), 1.89 (s, 2H), 1.23 (t, *J* = 7.0 Hz, 3H). **^{13}C NMR** (126 MHz, $CDCl_3$) δ 173.0, 140.3, 132.8, 132.1, 130.6, 129.0, 126.2, 61.7, 57.7, 14.1. **HRMS** (ESI) m/z calcd for $C_{10}H_{11}Cl_2NNaO_2$ $[M+Na]^+$ 270.0059, Found: 270.0064.

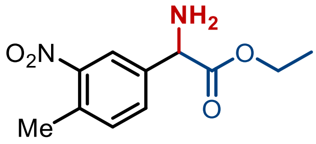

20

(20) ethyl 2-amino-2-(4-methyl-3-nitrophenyl)acetate

Prepared according to **General Procedure A** using ethyl 2-diazo-2-(4-methyl-3-nitrophenyl) acetate **S20** (0.3 mmol, 75.1 mg) and NH_3 H_2O (308 μL, 2.4 mmol) afforded compound **20** (50.7 mg, 71% yield) as a colourless oil. **^{1}H NMR** (500 MHz, $CDCl_3$) δ 8.03 (d, *J* = 1.5 Hz, 1H), 7.55 (dd, *J* = 8.0, 1.5 Hz, 1H), 7.31 (d, *J* = 8.0 Hz, 1H), 4.65 (s, 1H), 4.23-4.09 (m, 2H), 2.56 (s, 3H), 2.06 (s, 2H), 1.21 (t, *J* = 7.5 Hz, 3H). **^{13}C NMR** (126 MHz, $CDCl_3$) δ 172.9, 149.1, 139.5, 133.1, 133.0, 131.3, 123.1, 61.7, 57.6, 20.1, 14.0. **HRMS** (ESI) m/z calcd for $C_{11}H_{14}N_2NaO_4$ $[M+Na]^+$ 261.0846, Found: 261.0849.

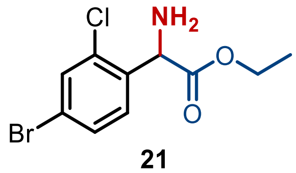

21

(21) ethyl 2-amino-2-(4-bromo-2-chlorophenyl)acetate

Prepared according to **General Procedure A** using ethyl 2-(4-bromo-2-chlorophenyl)-2-diazoacetate **S21** (0.3 mmol, 91.1 mg) and $NH_3 \cdot H_2O$ (308 μL, 2.4 mmol) afforded compound **21** (65.8 mg, 75%

yield) as a colourless oil. **^{1}H NMR** (500 MHz, $CDCl_3$) δ 7.57 (d, *J* = 2.0 Hz, 1H), 7.41 (dd, *J* = 8.5, 2.0 Hz, 1H), 7.27 (d, *J* = 8.5 Hz, 1H), 4.97 (s, 1H), 4.26-4.14 (m, 2H), 2.08 (s, 2H), 1.22 (t, *J* = 7.2 Hz, 3H). **^{13}C NMR** (151 MHz, $CDCl_3$) δ 172.7, 137.1, 134.4, 132.5, 130.6, 129.8, 122.2, 61.8, 55.9, 14.0. **HRMS** (ESI) m/z calcd for $C_{10}H_{11}BrClNNaO_2$ $[M+Na]^+$ 313.9554, Found: 313.9546.

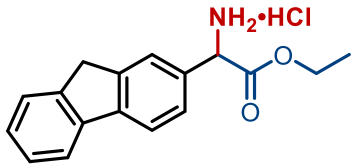

22

(22) ethyl 2-amino-2-(9*H*-fluoren-2-yl)acetate hydrochloride

Prepared according to **General Procedure B** using ethyl 2-diazo-2-(9H-fluoren-2-yl) acetate **S22** (0.3 mmol, 72.1 mg) and $NH_3{\cdot}H_2O$ (308 μL, 2.4 mmol) afforded compound **22** (79.3 mg, 87% yield) as a colourless oil. **^{1}H NMR** (500 MHz, DMSO) δ 9.19 (s, 3H), 7.96 (dd, *J* = 24.0, 8.0 Hz, 2H), 7.74 (s, 1H), 7.57 (dd, *J* = 34.0, 7.5 Hz, 2H), 7.42-7.34 (m, 2H), 5.29 (s, 1H), 4.27-4.12 (m, 2H), 3.96 (s, 2H), 1.14 (t, *J* = 7.0 Hz, 3H). **^{13}C NMR** (126 MHz, DMSO) δ 169.4, 144.4, 144.3, 143.2, 141.1, 131.9, 128.3, 128.0, 127.8, 126.2, 126.0, 121.4, 121.3, 63.0, 56.5, 37.4, 14.8. **HRMS** (ESI) m/z calcd for $C_{17}H_{18}NO_2$ $[M+H]^+$ 268.1332, Found: 268.1327.

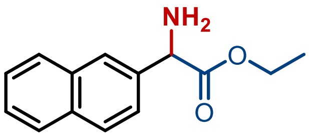

23

(23) ethyl 2-amino-2-(naphthalen-2-yl)acetate

Prepared according to **General Procedure A** using ethyl 2-diazo-2-(naphthalen-2-yl) acetate **S23** (0.3 mmol, 72.1 mg) and $NH_3{\cdot}H_2O$ (308 μL, 2.4 mmol) afforded compound **23** (59.8 mg, 87% yield) as a colourless oil. **^{1}H NMR** (500 MHz, $CDCl_3$) δ 7.86-7.81 (m, 4H), 7.52-7.45 (m, 3H), 4.77 (s, 1H), 4.26-4.09 (m, 2H), 2.15 (s, 2H), 1.20 (t, *J* = 7.0 Hz, 3H). **^{13}C NMR** (126 MHz, $CDCl_3$) δ 173.8, 137.7, 133.3, 132.9, 128.5, 127.9, 127.6, 126.2, 126.1, 125.7, 124.6, 61.3, 58.9, 14.0. **HRMS** (ESI) m/z calcd for $C_{14}H_{15}NNaO_2$ $[M+Na]^+$ 252.0995, Found: 252.1003.

Spectroscopic data are in agreement with those reported in the literature.[5]

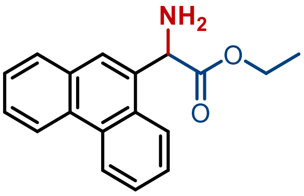

24

(24) ethyl 2-amino-2-(phenanthren-9-yl)acetate

Prepared according to **General Procedure A** using ethyl 2-diazo-2-(phenanthren-9-yl) acetate **S24** (0.3 mmol, 87.1 mg) and $NH_3 \cdot H_2O$ (308 μL, 2.4 mmol) afforded compound **24** (69.6 mg, 83% yield) as a colourless oil. **^{1}H NMR** (500 MHz, $CDCl_3$) δ 8.76 (d, J = 7.5 Hz, 1H), 8.67 (d, J = 8.0 Hz, 1H), 8.21 (d, J = 8.0 Hz, 1H), 7.87 (d, J = 8.0 Hz, 1H), 7.74 (s, 1H), 7.71-7.64 (m, 3H), 7.61 (t, J = 7.0 Hz, 1H), 5.36 (s, 1H), 4.30-4.16 (m, 2H), 2.44 (s, 2H), 1.17 (t, J = 7.5 Hz, 3H). **^{13}C NMR** (151 MHz, $CDCl_3$) δ 174.7, 134.4, 131.2, 131.0, 130.3, 129.7, 128.7, 127.1, 126.9, 126.8, 126.6, 126.1, 124.0, 123.4, 122.5, 61.7, 56.4, 14.0. **HRMS** (ESI) m/z calcd for $C_{18}H_{17}NNaO_2$ $[M+Na]^+$ 302.1151, Found: 302.1156.

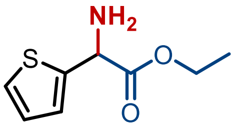

25

(25) ethyl 2-amino-2-(thiophen-2-yl)acetate

Prepared according to **General Procedure A** using ethyl 2-diazo-2-(thiophen-2-yl) acetate **S25** (0.3 mmol, 58.9 mg) and $NH_3 \cdot H_2O$ (308 μL, 2.4 mmol) afforded compound **25** (29.5 mg, 53% yield) as a colourless oil. **^{1}H NMR** (500 MHz, $CDCl_3$) δ 7.24 (dd, J = 5.0, 1.0 Hz, 1H), 7.05 (d, J = 3.5 Hz, 1H), 6.97 (dd, J = 5.0, 3.5 Hz, 1H), 4.86 (s, 1H), 4.29-4.18 (m, 2H), 2.09 (s, 2H), 1.28 (t, J = 7.0 Hz, 3H). **^{13}C NMR** (126 MHz, $CDCl_3$) δ 172.7, 143.5, 126.9, 125.1, 124.7, 61.7, 54.6, 14.1. **IR** (Film): 3714, 2845, 2528, 1734, 903, 718 cm^{-1}.

Spectroscopic data are in agreement with those reported in the literature.[6]

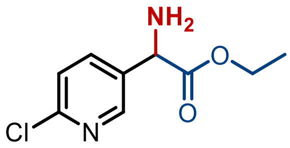

26

(26) ethyl 2-amino-2-(6-chloropyridin-3-yl)acetate

Prepared according to **General Procedure A** using ethyl 2-(6-chloropyridin-3-yl)-2-diazoacetate **S26** (0.3 mmol, 67.7 mg) and $NH_3 \cdot H_2O$ (308 μL, 2.4 mmol) afforded compound **26** (35.4 mg, 55% yield) as a colourless oil. **^{1}H NMR** (500 MHz, $CDCl_3$) δ 8.42 (s, 1H), 7.72 (d, J = 8.0 Hz, 1H), 7.30 (d, J = 8.0 Hz, 1H), 4.63 (s, 1H), 4.22-4.08 (m, 2H), 2.04 (s, 2H), 1.20 (t, J = 7.0 Hz, 3H). **^{13}C NMR** (151 MHz, $CDCl_3$) δ 172.7, 150.9, 148.6, 137.2, 134.7, 124.2, 61.8, 55.7, 14.0. **HRMS** (ESI) m/z calcd for $C_9H_{12}ClN_2O_2$ $[M+H]^+$ 215.0582, Found: 215.0586.

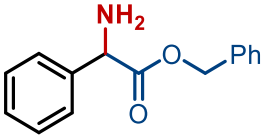

27

(27) benzyl 2-amino-2-phenylacetate

Prepared according to **General Procedure A** using benzyl 2-diazo-2-phenylacetate **S27** (0.3 mmol, 75.7 mg) and $NH_3 \cdot H_2O$ (308 μL, 2.4 mmol) afforded compound **27** (60.8 mg, 84% yield) as a colourless oil. **^{1}H NMR** (500 MHz, $CDCl_3$) δ 7.37-7.32 (m, 4H), 7.32-7.27 (m, 4H), 7.21-7.18 (m, 2H), 5.13 (ABq, J = 12.5 Hz, 2H), 4.66 (s, 1H), 2.27 (s, 2H). **^{13}C NMR** (126 MHz, $CDCl_3$) δ 173.6, 139.8, 135.4, 128.7, 128.4, 128.2, 128.0, 127.9, 126.8, 66.9, 58.7.

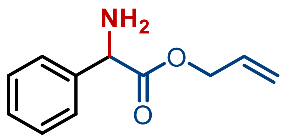

28

(28) allyl 2-amino-2-phenylacetate

Prepared according to **General Procedure A** using allyl 2-diazo-2-phenylacetate **S28** (0.3 mmol, 60.7 mg) and $NH_3 \cdot H_2O$ (308 μL, 2.4 mmol) afforded compound **28** (49.3 mg, 86% yield) as a colourless oil. Prepared according to **General Procedure C** using allyl (*Z*)-2-phenyl-2-(2-((2-(trifluoromethyl) phenyl) sulfonyl) hydrazineylidene) acetate **S28'** (0.3 mmol, 123.7 mg) and $NH_3 \cdot H_2O$ (308 μL, 2.4 mmol) afforded compound **28** (49.3 mg, 86% yield) as a colourless oil.
^{1}H NMR (500 MHz, $CDCl_3$) δ 7.41-7.33 (m, 4H), 7.33-7.28 (m, 1H), 5.88-5.79 (m, 1H), 5.22-5.15 (m, 2H), 4.64 (s, 1H), 4.63-4.56 (m, 2H), 1.90 (s, 2H). **^{13}C NMR** (126 MHz, $CDCl_3$) δ 173.6, 140.2, 131.7, 128.8, 128.0, 126.8, 118.3, 65.7, 58.8. **IR** (Film): 3202, 2944, 2627, 2253, 1442, 1375, 1039, 918 cm^{-1}.

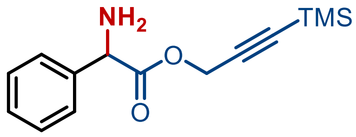

29

(29) 3-(trimethylsilyl)prop-2-yn-1-yl 2-amino-2-phenylacetate

Prepared according to **General Procedure A** using 3-(trimethylsilyl)prop-2-yn-1-yl 2-diazo-2-phenylacetate **S29** (0.3 mmol, 81.7 mg) and $NH_3 \cdot H_2O$ (308 μL, 2.4 mmol) afforded compound **29** (58.0 mg, 74% yield) as a colourless oil. **^{1}H NMR** (500 MHz, $CDCl_3$) δ 7.41-7.28 (m, 5H), 4.81-4.60 (m, 3H), 2.03 (s, 2H), 0.15 (s, 9H). **^{13}C NMR** (151 MHz, $CDCl_3$) δ 173.2, 139.7, 128.8, 128.1, 126.8, 98.4, 92.4, 58.6, 53.4, -0.4. **IR** (Film): 3164, 3000, 2760, 2253, 1742, 1265, 905, 726 cm^{-1}.

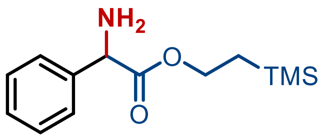

30

(30) 2-(trimethylsilyl)ethyl 2-amino-2-phenylacetate

Prepared according to **General Procedure A** using 2-(trimethylsilyl)ethyl 2-diazo-2-phenylacetate **S30** (0.3 mmol, 78.7 mg) and $NH_3 \cdot H_2O$ (308 μL, 2.4 mmol) afforded compound **30** (65.6 mg, 87% yield) as

a colourless oil. **^{1}H NMR** (500 MHz, $CDCl_3$) δ 7.39-7.33 (m, 4H), 7.33-7.28 (m, 1H), 4.56 (s, 1H), 4.27-4.11 (m, 2H), 1.90 (s, 2H), 0.96-0.90 (m, 2H), 0.02 (s, 9H). **^{13}C NMR** (126 MHz, $CDCl_3$) δ 174.1, 140.5, 128.7, 127.9, 126.8, 63.7, 58.9, 17.2, -1.6. **HRMS** (ESI) m/z calcd for $C_{13}H_{21}NNaO_2Si$ $[M+H]^+$ 274.1234, Found: 274.1235.

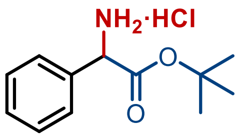

31

(31) *tert*-butyl 2-amino-2-phenylacetate hydrochloride

Prepared according to **General Procedure B** using tert-butyl 2-diazo-2-phenylacetate **S31** (0.3 mmol, 65.5 mg) and $NH_3 \cdot H_2O$ (308 μL, 2.4 mmol) afforded compound **31** (65.1 mg, 89% yield) as a white solid, m.p. 166-168 °C. **^{1}H NMR** (500 MHz, DMSO) δ 8.81 (s, 3H), 7.46-7.40 (m, 5H), 5.06 (s, 1H), 1.33 (s, 9H). **^{13}C NMR** (126 MHz, DMSO) δ 168.3, 134.0, 130.3, 129.8, 129.0, 84.1, 56.8, 28.3. **HRMS** (ESI) m/z calcd for $C_{12}H_{18}NO_2$ $[M+H]^+$ 208.1332, Found: 208.1338.

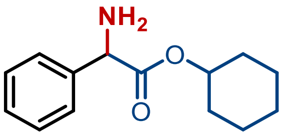

32

(32) tyclohexyl 2-amino-2-phenylacetate

Prepared according to **General Procedure A** using cyclohexyl 2-diazo-2-phenylacetate **S32** (0.3 mmol, 73.3 mg) and $NH_3 \cdot H_2O$ (308 μL, 2.4 mmol) afforded compound **32** (62.3 mg, 89% yield) as a colourless oil. Prepared according to **General Procedure C** using cyclohexyl (*Z*)-2-phenyl-2-(2-((2-(trifluoromethyl) phenyl) sulfonyl) hydrazineylidene) acetate **S19** (0.3 mmol, 136.3 mg) and $NH_3 \cdot H_2O$ (308 μL, 2.4 mmol) afforded **32** (56.7 mg, 81% yield) as a colourless oil.

^{1}H NMR (600 MHz, $CDCl_3$) δ 7.40-7.32 (m, 4H), 7.31-7.26 (m, 1H), 4.83-4.75 (m, 1H), 4.58 (s, 1H), 1.88 (s, 2H), 1.85-1.78 (m, 1H), 1.72-1.63 (m, 2H), 1.57-1.40 (m, 3H), 1.39-1.17 (m, 4H). **^{13}C NMR** (126 MHz, $CDCl_3$) δ 173.4, 140.7, 128.6, 127.8, 126.7, 73.5, 58.9, 31.4, 31.1, 25.2, 23.5, 23.3. **HRMS** (ESI) m/z calcd for $C_{14}H_{19}NNaO_2$ $[M+Na]^+$ 256.1308, Found: 256.1308.

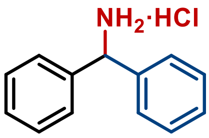

33

(33) diphenylmethanamine hydrochloride

Prepared according to **General Procedure B** using (diazomethylene) dibenzene **S33** (0.3 mmol, 58.2 mg) and $NH_3 \cdot H_2O$ (308 μL, 2.4 mmol) afforded compound **33** (45.0 mg, 82% yield) as a white solid, m.p. 181-183 °C. Prepared according to **General Procedure D** using

N'-(diphenylmethylene)-2-(trifluoromethyl) benzenesulfonohydrazide **S33'** (0.3 mmol, 121.3 mg) and $NH_3 \cdot H_2O$ (308 µL, 2.4 mmol) afforded compound **33** (46.7 mg, 85% yield) as a white solid.

^{1}H NMR (600 MHz, DMSO) δ 9.30 (d, *J* = 38.4 Hz, 3H), 7.58-7.56 (m, 4H), 7.41-7.38 (m, 4H), 7.35-7.31 (m, 2H), 5.61 (s, 1H). **^{13}C NMR** (151 MHz, DMSO) δ 139.0, 129.1, 128.6, 127.9, 57.6. **HRMS** (ESI) m/z calcd for $C_{13}H_{14}N$ $[M+H]^+$ 184.1121, Found: 184.1129.

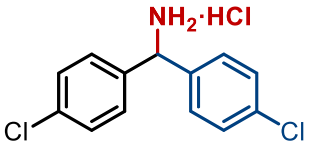

34

(34) *bis*(4-chlorophenyl)methanamine hydrochloride

Prepared according to **General Procedure B** using 4,4'-(diazomethylene)*bis*(chlorobenzene) **S34** (0.3 mmol, 78.9 mg) and $NH_3 \cdot H_2O$ (308 µL, 2.4 mmol) afforded compound **34** (77.9 mg, 90% yield) as a white solid, m.p. 165-167 ºC. Prepared according to **General Procedure D** using *N'*-(*bis*(4-chlorophenyl)methylene)-2-(trifluoromethyl)benzenesulfonohydrazide **S34'** (0.3 mmol, 142.0 mg) and $NH_3 \cdot H_2O$ (308 µL, 2.4 mmol) afforded compound **34** (64.3 mg, 85% yield) as a white solid.

^{1}H NMR (500 MHz, DMSO) δ 9.38 (s, 3H), 7.65-7.44 (m, 8H), 5.73 (s, 1H). **^{13}C NMR** (126 MHz, DMSO) δ 137.9, 134.1, 130.3, 129.7, 56.5. **HRMS** (ESI) m/z calcd for $C_{13}H_{12}Cl_2N$ $[M+H]^+$ 252.0341, Found: 252.0340.
Spectroscopic data are in agreement with those reported in the literature.[7]

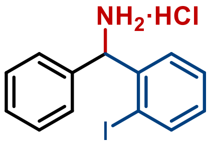

35

(35) (2-iodophenyl)(phenyl)methanamine hydrochloride

Prepared according to **General Procedure B** using 1-(diazo(phenyl)methyl)-2-iodobenzene **S35** (0.3 mmol, 96.0 mg) and $NH_3 \cdot H_2O$ (308 µL, 2.4 mmol) afforded compound **35** (98.5 mg, 95% yield) as a white solid, m.p. 177-179 ºC. **^{1}H NMR** (500 MHz, DMSO) δ 9.46 (s, 3H), 7.94 (ddd, *J* = 8.0, 3.5, 1.5 Hz, 2H), 7.57-7.52 (m, 1H), 7.51-7.47 (m, 2H), 7.43-7.33 (m, 3H), 7.14 (td, *J* = 8.0, 1.5 Hz, 1H), 5.61 (s, 1H). **^{13}C NMR** (126 MHz, DMSO) δ 140.8, 140.6, 137.4, 131.3, 129.8, 129.6, 129.5, 129.3, 128.9, 101.2, 62.0. **IR** (Film): 3477, 3004, 2845, 2251, 1661, 1023, 1003, 821, 758 cm^{-1}.

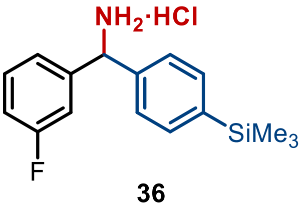

36

(36) (3-fluorophenyl)(4-(trimethylsilyl)phenyl)methanamine hydrochloride

Prepared according to **General Procedure B** using (4-(diazo(3-fluorophenyl)methyl)phenyl) trimethylsilane **S36** (0.3 mmol, 85.3 mg) and $NH_3 \cdot H_2O$ (308 μL, 2.4 mmol) afforded compound **36** (75.3 mg, 81% yield) as a white solid, m.p. 155-157 °C. **^{1}H NMR** (500 MHz, DMSO) δ 9.23 (s, 3H), 7.42-7.30 (m, 5H), 7.27-7.19 (m, 2H), 6.99-6.92 (m, 1H), 5.45 (s, 1H), 0.00 (s, 9H). **^{13}C NMR** (151 MHz, DMSO) δ 163.0 (d, J = 244.0 Hz), 142.1 (d, J = 7.6 Hz), 141.1, 139.5, 134.5, 131.8 (d, J = 7.6 Hz), 127.7, 124.5 (d, J = 3.0 Hz), 116.0 (d, J = 21.0 Hz), 115.3 (d, J = 22.7 Hz), 57.5, -0.3. **^{19}F NMR** (564 MHz, DMSO) δ (-112.13)–(-112.23) (m, 1F). **IR** (Film): 3434, 2249, 2124, 1024, 1004, 821, 758 cm^{-1}.

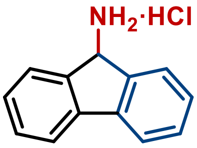

37

(37) 9*H*-fluoren-9-amine hydrochloride

Prepared according to **General Procedure B** using 9-diazo-9*H*-fluorene **S37** (0.3 mmol, 57.7 mg) and $NH_3 \cdot H_2O$ (308 μL, 2.4 mmol) afforded compound **37** (44.4 mg, 68% yield) as a white solid, m.p. 147-149 °C. **^{1}H NMR** (500 MHz, DMSO) δ 9.35 (s, 3H), 8.09 (d, J = 5.0 Hz, 2H), 7.92 (d, J = 5.5 Hz, 2H), 7.55-7.36 (m, 4H), 5.37 (s, 1H). **^{13}C NMR** (151 MHz, DMSO) δ 141.3, 141.1, 130.5, 128.7, 126.9, 121.4, 54.4. **IR** (Film): 3392, 2253, 1715, 903, 724 cm^{-1}.

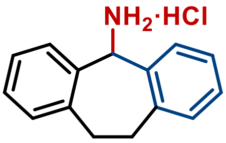

38

(38) 10,11-dihydro-5*H*-dibenzo[*a*,*d*][7]annulen-5-amine

Prepared according to **General Procedure B** using 5-diazo-10,11-dihydro-5H-dibenzo[*a*,*d*][7]annulene **S38** (0.3 mmol, 66.1 mg) and $NH_3 \cdot H_2O$ (308 μL, 2.4 mmol) afforded compound **38** (48.4 mg, 77% yield) as a white solid, m.p. 198-200 °C. **^{1}H NMR** (500 MHz, DMSO) δ 9.32 (s, 3H), 7.52 (d, J = 7.5 Hz, 2H), 7.30-7.20 (m, 6H), 5.85 (s, 1H) 3.20 (s, 4H). **^{13}C NMR** (151 MHz, DMSO) δ 139.5, 136.4, 131.3, 129.5, 128.1, 127.1, 55.9, 32.4. **IR** (Film): 3435, 3045, 2977, 2250, 2125, 1645, 1278, 1024, 1005, 820, 758, 727, cm^{-1}.

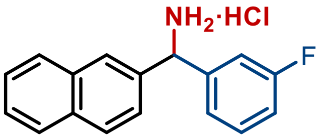

39

(39) (3-fluorophenyl)(naphthalen-2-yl) methanamine

Prepared according to **General Procedure B** using 2-(diazo(3-fluorophenyl)methyl)naphthalene **S39** (0.3 mmol, 78.7 mg) and $NH_3 \cdot H_2O$ (308 μL, 2.4 mmol) afforded compound **39** (67.1 mg, 89% yield) as a white solid, m.p. 161-163 ºC. **^{1}H NMR** (500 MHz, DMSO) δ 9.48 (s, 3H), 8.20-8.12 (m, 1H), 8.06-7.95 (m, 3H), 7.67 (t, *J* = 7.5 Hz, 1H), 7.61-7.54 (m, 2H), 7.53-7.49 (m, 1H), 7.45-7.35 (m, 2H), 7.18 (t, *J* = 7.5 Hz, 1H), 6.48 (q *J* = 4.5 Hz, 1H). **^{13}C NMR** (126 MHz, DMSO) δ 162.4 (d, *J* = 244.0 Hz), 140.8 (d, *J* = 7.6 Hz), 133.9, 133.5, 131.2 (d, *J* = 8.4 Hz), 130.1, 129.5, 129.3, 127.4, 126.7, 125.8, 125.0 (d, *J* = 1.3 Hz), 124.6, 123.8, 115.9 (d, *J* = 21.0 Hz), 115.8 (d, *J* = 22.5 Hz), 53.3. **^{19}F NMR** (564 MHz, DMSO) δ (-112.27)–(-112.43) (m, 1F). **HRMS** (ESI) m/z calcd for $C_{17}H_{15}FN$ $[M+H]^+$ 252.1183, Found: 252.1183.

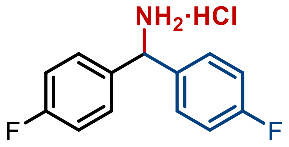

40

(40) *bis*(4-fluorophenyl)methanamine hydrochloride

Prepared according to **General Procedure D** using *N'*-((4-fluorophenyl)methylene)-2-(trifluoromethyl) benzenesulfonohydrazide **S40'** (0.3 mmol, 132.1 mg) and $NH_3 \cdot H_2O$ (308 μL, 2.4 mmol) afforded compound **40** (74.4 mg, 97% yield) as awhite solid, m.p. 163-165 ºC. **^{1}H NMR** (500 MHz, DMSO) δ 9.39 (s, 3H), 7.64 (dd, *J* = 8.0, 5.5 Hz, 4H), 7.24 (t, *J* = 8.5 Hz, 4H), 5.70 (s, 1H). **^{13}C NMR** (126 MHz, DMSO) δ162.8 (d, *J* = 245.7 Hz), 135.6 (d, *J* = 2.5 Hz), 130.7 (d, *J* = 8.8 Hz), 116.5 (d, *J* = 22.7 Hz), 56.7. **^{19}F NMR** (564 MHz, DMSO) δ -113.80 (s, 2F). **HRMS** (ESI) m/z calcd for $C_{13}H_{12}F_2N$ $[M+H]^+$ 220.0932, Found: 220.0939.

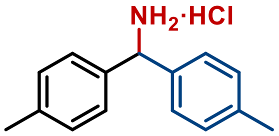

41

(41) di-*p*-tolylmethanamine hydrochloride

Prepared according to **General Procedure D** using *N'*-(di-*p*-tolylmethylene)-2-(trifluoromethyl) benzenesulfonohydrazide **S41'** (0.3 mmol, 129.7 mg) and $NH_3 \cdot H_2O$ (308 μL, 2.4 mmol) afforded compound **41** (44.6 mg, 60% yield) as a white solid, m.p. 171-173 ºC). **^{1}H NMR** (500 MHz, DMSO) δ 9.18 (d, *J* = 3.5 Hz, 3H), 7.41 (d, *J* = 8.0 Hz, 4H), 7.19 (d, *J* = 8.0 Hz, 4H), 5.50 (q, *J* = 5.5 Hz, 1H), 2.27 (s, 6H). **^{13}C NMR** (126 MHz, DMSO) δ 138.4, 136.6, 130.1, 128.2, 57.60, 21.6. **HRMS** (ESI) m/z calcd for $C_{15}H_{18}N$ $[M+H]^+$ 212.1434, Found: 212.1428.

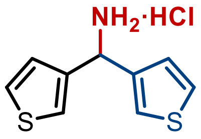

42

(42) di(thiophen-3-yl)methanamine hydrochloride

Prepared according to **General Procedure D** using *N'*-(di(thiophen-3-yl)methylene)-2-(trifluoromethyl)benzenesulfonohydrazide **S42'** (0.3 mmol, 124.9 mg) and $NH_3 \cdot H_2O$ (308 μL, 2.4 mmol) afforded compound **42** (50.1 mg, 72% yield) as a white solid (mp: 154-156 °C). ^{1}**H NMR** (500 MHz, DMSO) δ 9.13 (s, 3H), 7.64 (s, 2H), 7.60 (dd, *J* = 4.9, 3.0 Hz, 2H), 7.66-7.63 (m, 2H), 5.78-5.73 (m, 1H). ^{13}**C NMR** (126 MHz, DMSO) δ 139.9, 128.3, 127.9, 124.9, 50.1. **IR** (Film): 3477, 3009, 2250, 1052, 1024, 1005, 820, 757 cm^{-1}.

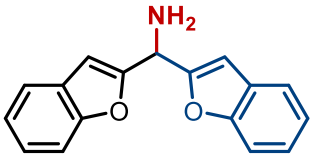

43

(43) di(benzofuran-2-yl)methanamine

Prepared according to **General Procedure C** using *N'*-(di(benzofuran-2-yl)methylene)-2-(trifluoromethyl) benzenesulfonohydrazide **S43'** (0.3 mmol, 145.3 mg) and $NH_3 \cdot H_2O$ (308 μL, 2.4 mmol) afforded compound **43** (43.4 mg, 55% yield) as a colourless oil. ^{1}**H NMR** (500 MHz, $CDCl_3$) δ 7.54 (d, *J* = 7.0 Hz, 2H), 7.45 (d, *J* = 8.5 Hz, 2H), 7.26 (td, *J* = 7.5, 1.5 Hz, 2H), 7.24-7.21 (m, 2H), 6.67 (s, 2H), 5.45 (s, 1H), 2.08 (s, 2H). ^{13}**C NMR** (126 MHz, $CDCl_3$) δ 157.4, 154.9, 128.1, 124.2, 122.8, 121.0, 111.3, 103.3, 49.2. **IR** (Film): 3442, 3052, 2361, 2253, 1264, 904, 725 cm^{-1}.

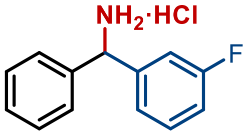

44

(44) (2-fluorophenyl)(phenyl)methanamine hydrochloride

Prepared according to **General Procedure D** using *N'*-((2-fluorophenyl)(phenyl)methylene)-2-(trifluoromethyl) benzenesulfonohydrazide **S44'** (0.3 mmol, 126.7 mg) and $NH_3 \cdot H_2O$ (308 μL, 2.4 mmol) afforded compound **44** (56.1 mg, 93% yield) as a white solid (mp: 150-152 °C). ^{1}**H NMR** (500 MHz, DMSO) δ 9.41 (s, 3H), 7.60 (d, *J* = 7.5 Hz, 2H), 7.54 (d, *J* = 10.5 Hz, 1H), 7.48-7.39 (m, 4H), 7.34 (t, *J* = 7.5 Hz, 1H), 7.21-7.15 (m, 1H), 5.67 (q, *J* = 4.5 Hz, 1H). ^{13}**C NMR** (126 MHz, DMSO) δ 163.0 (d, *J* = 244.4 Hz), 142.1 (d, *J* = 7.3 Hz), 139.0, 131.8 (d, *J* = 8.4 Hz), 129.7, 129.3, 128.4, 124.6 (d, *J* = 2.2 Hz), 116.0 (d, *J* = 21.4 Hz), 115.3 (d, *J* = 23.9 Hz), 57.5. ^{19}**F NMR** (470 MHz, DMSO) δ (-112.17)-(-112.27) (m). **HRMS** (ESI) m/z calcd for $C_{13}H_{13}FN$ $[M+H]^+$ 202.1027, Found: 202.1027.

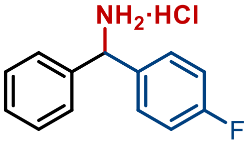

45

(45) (4-fluorophenyl)(phenyl)methanamine hydrochloride

Prepared according to **General Procedure D** using *N'*-((4-fluorophenyl)(phenyl) methylene)-2-(trifluoromethyl) benzenesulfonohydrazide **S45'** (0.3 mmol, 126.7 mg) and $NH_3 \cdot H_2O$ (308 μL, 2.4 mmol) afforded compound **45** (72.3 mg, 88% yield) as a white solid (mp: 154-156 °C). ^{1}**H NMR** (500 MHz, DMSO) δ 9.31 (s, 3H), 7.65-7.59 (m, 2H), 7.56 (d, J = 7.5 Hz, 2H), 7.41 (t, J = 7.5 Hz, 2H), 7.34 (t, J = 7.5 Hz, 1H), 7.29-7.23 (m, 2H), 5.66 (s, 1H). ^{13}**C NMR** (126 MHz, DMSO) δ162.8 (d, J = 244.4 Hz), 139.2, 135.7 (d, J = 2.5 Hz), 130.7 (d, J = 8.8 Hz), 129.7, 129.2, 128.2, 116.5 (d, J = 21.4 Hz), 57.3. ^{19}**F NMR** (470 MHz, DMSO) δ (-113.77)-(-113.86) (m). **HRMS** (ESI) m/z calcd for $C_{13}H_{13}FN$ $[M+H]^+$ 202.1027, Found: 202.1018.

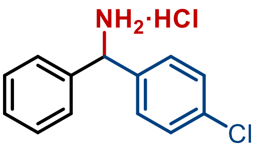

46

(46) (4-chlorophenyl)(phenyl)methanamine hydrochloride

Prepared according to **General Procedure D** using *N'*-((4-chlorophenyl)(phenyl) methylene)-2-(trifluoromethyl) benzenesulfonohydrazide **S46** (0.3 mmol, 131.7 mg) and $NH_3 \cdot H_2O$ (308 μL, 2.4 mmol) afforded compound **46** (74.7 mg, 98% yield) as a white solid (mp: 159-161 °C). ^{1}**H NMR** (500 MHz, DMSO) δ 9.39 (s, 3H), 7.63-7.55 (m, 4H), 7.48 (d, J = 8.0 Hz, 2H), 7.40 (t, J = 7.0 Hz, 2H), 7.36-7.31 (m, 1H), 5.66 (s, 1H). ^{13}**C NMR** (126 MHz, DMSO) δ 139.0, 138.4, 133.9, 130.4, 129.7, 129.6, 129.3, 128.3, 57.3. HRMS (ESI) m/z calcd for $C_{13}H_{13}ClN$ $[M+H]^+$ 218.0731, Found: 218.0729.

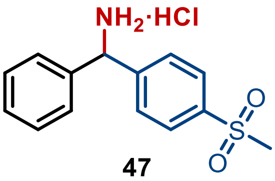

47

(47) (4-(methylsulfonyl)phenyl)(phenyl)methanamine hydrochloride

Prepared according to **General Procedure D** using *N'*-((4-(methylsulfonyl)phenyl)(phenyl) methylene)-2-(trifluoromethyl) benzenesulfonohydrazide **S47'** (0.3 mmol, 144.7 mg) and $NH_3 \cdot H_2O$ (308 μL, 2.4 mmol) afforded compound **47** (78.6 mg, 88% yield) as a white solid (mp: 171-173 °C). ^{1}**H NMR** (500 MHz, DMSO) δ 9.50 (s, 3H), 7.97 (d, J = 8.0 Hz, 2H), 7.87 (d, J = 8.0 Hz, 2H), 7.61 (d, J = 7.5 Hz, 2H), 7.42 (t, J = 7.5 Hz, 2H), 7.35 (t, J = 7.0 Hz, 1H), 5.78 (q, J = 3.0 Hz, 1H), 3.22 (s, 3H). ^{13}**C NMR** (126 MHz, DMSO) δ 144.9, 141.5, 138.6, 129.8, 129.5, 129.3, 128.4, 128.3, 57.5, 44.3. **HRMS** (ESI) m/z calcd for $C_{14}H_{16}NO_2S$ $[M+H]^+$ 262.0896, Found: 262.0900.

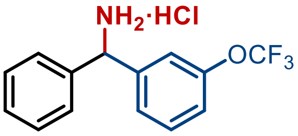

48

(48) phenyl(3-(trifluoromethoxy)phenyl)methanamine hydrochloride

Prepared according to **General Procedure D** using *N*'-(phenyl(3-(trifluoromethoxy)phenyl) methylene)-2-(trifluoromethyl) benzenesulfonohydrazide **S48'** (0.3 mmol, 146.5 mg) and $NH_3 \cdot H_2O$ (308 μL, 2.4 mmol) afforded compound **48** (74.6 mg, 93% yield) as a white solid (mp: 137-139 °C). **^{1}H NMR** (500 MHz, DMSO) δ 9.45 (s, 3H), 7.70-7.64 (m, 2H), 7.62 (d, *J* = 7.5 Hz, 2H), 7.55 (t, *J* = 8.0 Hz, 1H), 7.41 (t, *J* = 7.0 Hz, 2H), 7.37-7.32 (m, 2H), 5.74 (s, 1H). **^{13}C NMR** (126 MHz, DMSO) δ 149.4, 142.1, 138.9, 131.8, 129.8, 129.4, 128.4, 127.6, 121.6, 121.2, 121.0 (q, *J* = 257.0 Hz), 57.4. **^{19}F NMR** (470 MHz, DMSO) δ -56.71. **HRMS** (ESI) m/z calcd for $C_{14}H_{13}F_3NO$ $[M+H]^+$ 268.0944, Found: 268.0953.

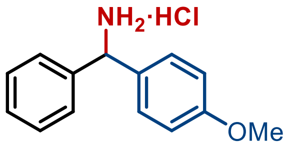

49

(49) (4-methoxyphenyl)(phenyl)methanamine hydrochloride

Prepared according to **General Procedure D** using *N*'-((4-methoxyphenyl)(phenyl) methylene)-2-(trifluoromethyl) benzenesulfonohydrazide **S49'** (0.3 mmol, 130.3 mg) and $NH_3 \cdot H_2O$ (308 μL, 2.4 mmol) afforded compound **49** (59.9 mg, 80% yield) as a white solid (mp: 155-157 °C). **^{1}H NMR** (500 MHz, DMSO) δ 9.21 (s, 3H), 7.55 (d, *J* = 7.5 Hz, 2H), 7.47 (d, *J* = 8.5 Hz, 2H), 7.40 (t, *J* = 7.5 Hz, 2H), 7.33 (t, *J* = 7.0 Hz, 1H), 6.96 (d, *J* = 8.5 Hz, 2H), 5.55 (s, 1H), 3.73 (s, 3H). **^{13}C NMR** (151 MHz, DMSO) δ 160.0, 139.7, 131.3, 129.8, 129.6, 129.0, 128.2, 115.0, 57.5, 56.2. **IR** (Film): 3477, 3251, 2248, 1052, 1024, 1005, 820, 757 cm^{-1}.

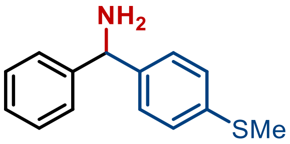

50

(50) (4-(methylthio)phenyl)(phenyl)methanamine

Prepared according to **General Procedure C** using *N*'-((4-(methylthio)phenyl)(phenyl)methylene)-2-(trifluoromethyl) benzenesulfonohydrazide **S50'** (0.3 mmol, 135.1 mg) and $NH_3 \cdot H_2O$ (308 μL, 2.4 mmol) afforded compound **50** (46.8 mg, 68% yield) as a colourless oil. **^{1}H NMR** (500 MHz, $CDCl_3$) δ 7.36 (d, *J* = 7.5 Hz, 2H), 7.34-7.28 (m, 4H), 7.25-7.19 (m, 3H), 5.18 (s, 1H), 2.46 (s, 3H), 1.92 (s, 2H). **^{13}C NMR** (151 MHz, $CDCl_3$) δ 145.3, 142.5, 136.9, 128.5, 127.4, 127.0, 126.82, 126.80, 59.3, 16.0. **IR** (Film): 3517, 3002, 2943, 2252, 1375, 1038, 917, 749 cm^{-1}.

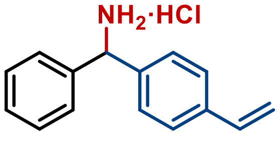

51

(51) phenyl(4-vinylphenyl)methanamine hydrochloride

Prepared according to **General Procedure D** using *N'*-(phenyl(4-vinylphenyl) methylene)-2-(trifluoromethyl) benzenesulfonohydrazide **S51'** (0.3 mmol, 129.1 mg) and $NH_3{\cdot}H_2O$ (308 μL, 2.4 mmol) afforded compound **51** (60.5 mg, 82% yield) as a white solid (mp: 144-146 °C). **^{1}H NMR** (500 MHz, DMSO) δ 9.37 (s, 3H), 7.61-7.55 (m, 4H), 7.50 (d, *J* = 8.0 Hz, 2H), 7.39 (t, *J* = 7.5 Hz, 2H), 7.33 (t, *J* = 7.5 Hz, 1H), 6.72 (dd, *J* = 17.5, 11.0 Hz, 1H), 5.85 (d, *J* = 17.5 Hz, 1H), 5.61 (q, *J* = 5.0 Hz, 1H), 5.27 (d, *J* = 11.0 Hz, 1H). **^{13}C NMR** (126 MHz, DMSO) δ 139.4, 138.9, 137.9, 136.9, 129.7, 129.2, 128.7, 128.4, 127.3, 116.1, 57.8. **HRMS** (ESI) m/z calcd for $C_{15}H_{16}N$ $[M+H]^+$ 210.1277, Found: 210.1273.

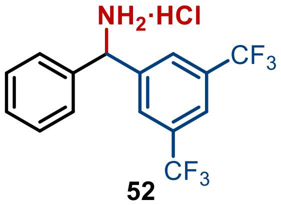

52

(52) (3,5-bis(trifluoromethyl)phenyl)(phenyl)methanamine hydrochloride

Prepared according to **General Procedure D** using *N'*-((3,5-*bis*(trifluoromethyl)phenyl)(phenyl) methylene)-2-(trifluoromethyl) benzenesulfonohydrazide **S52'** (0.3 mmol, 162.1 mg) and $NH_3{\cdot}H_2O$ (308 μL, 2.4 mmol) afforded compound **52** (102.4 mg, 96% yield) as a white solid (mp: 159-161 °C). **^{1}H NMR** (500 MHz, DMSO) δ 9.53 (s, 3H), 8.39 (s, 2H), 8.15 (s, 1H), 7.64-7.60 (m, 2H), 7.48-7.43 (m, 2H), 7.41-7.36 (m, 1H), 5.96 (s, 1H). **^{13}C NMR** (126 MHz, DMSO) δ 142.6, 138.1, 131.6 (q, *J* = 32.8 Hz), 130.0, 129.7, 129.6 (q, *J* = 2.5 Hz), 128.4, 124.1 (q, *J* = 273.4 Hz), 123.3-123.0 (m), 57.0. **^{19}F NMR** (564 MHz, DMSO) δ -61.29. **HRMS** (ESI) m/z calcd for $C_{15}H_{12}F_6N$ $[M+H]^+$ 320.0868, Found: 320.0867.

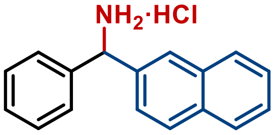

53

(53) naphthalen-2-yl(phenyl)methanamine hydrochloride

Prepared according to **General Procedure D** using *N'*-(naphthalen-2-yl(phenyl) methylene)-2-(trifluor-omethyl) benzenesulfonohydrazide **S53'** (0.3 mmol, 136.3 mg) and $NH_3{\cdot}H_2O$ (308 μL, 2.4 mmol) afforded compound **53** (63.1 mg, 78% yield) as a white solid (mp: 162-164 °C). **^{1}H NMR** (600 MHz, DMSO) δ 9.39 (d, *J* = 3.6 Hz, 3H), 8.14 (s, 1H), 7.94-7.87 (m, 3H), 7.65 (dd, *J* = 8.4, 1.2 Hz, 1H), 7.61 (d, *J* = 7.8 Hz, 2H), 7.57-7.51 (m, 2H), 7.40 (t, *J* = 7.2 Hz, 2H), 7.33 (t, *J* = 7.2 Hz, 1H), 5.77 (q, *J* = 5.4 Hz, 1H). **^{13}C NMR** (151 MHz, DMSO) δ 139.3, 136.9, 133.6, 133.4, 129.8, 129.5, 129.3, 128.9, 128.7, 128.5, 127.8, 127.7, 127.1, 126.1, 58.3. **IR** (Film): 3472, 2368, 1200, 732 cm^{-1}.

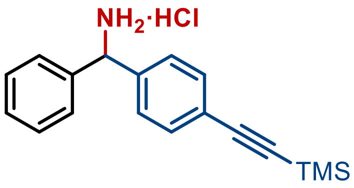

54

(54) phenyl(4-((trimethylsilyl)ethynyl)phenyl)methanamine hydrochloride

Prepared according to **General Procedure D** using *N'*-(phenyl(4-((trimethylsilyl)ethynyl)phenyl) methylene)-2-(trifluoromethyl) benzenesulfonohydrazide **S54'** (0.3 mmol, 150.2 mg) and $NH_3{\cdot}H_2O$ (308 μL, 2.4 mmol) afforded compound **54** (84.3 mg, 89% yield) as a white solid (mp: 147-149 °C). **^{1}H NMR** (500 MHz, DMSO) δ 9.44 (s, 3H), 7.62-7.56 (m, 4H), 7.48 (d, *J* = 8.5 Hz, 2H), 7.39 (t, *J* = 7.5 Hz, 2H), 7.33 (t, *J* = 7.5 Hz, 1H), 5.66 (s, 1H), 0.21 (s, 9H). **^{13}C NMR** (151 MHz, DMSO) δ 140.1, 139.0, 132.8, 129.7, 129.3, 128.8, 128.5, 122.9, 105.6, 95.8, 57.7, 0.8. **IR** (Film): 3485, 2248, 2123, 1621,1052, 1024, 1005, 820, 757 cm^{-1}.

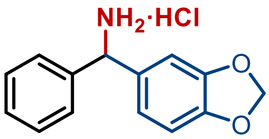

55

(55) benzo[*d*][1,3]dioxol-5-yl(phenyl)methanamine hydrochloride

Prepared according to **General Procedure D** using *N'*-(benzo[*d*][1,3]dioxol-5-yl(phenyl) methylene)-2-(trifluoromethyl) benzenesulfonohydrazide **S55'** (0.3 mmol, 134.5 mg) and $NH_3{\cdot}H_2O$ (308 μL, 2.4 mmol) afforded **55** (56.6 mg, 83% yield) as a white solid (mp: 160-162 °C). **^{1}H NMR** (500 MHz, DMSO) δ 9.16 (s, 3H), 7.63-7.27 (m, 5H), 7.18 (s, 1H), 6.97 (d, *J* = 28.5 Hz, 2H), 6.02 (s, 2H), 5.54 (s, 1H). **^{13}C NMR** (126 MHz, DMSO) δ 148.4, 148.0, 139.5, 133.1, 129.6, 129.1, 128.1, 122.1, 109.3, 108.8, 102.3, 57.8. **IR** (Film): 3465, 2984, 1736, 1372, 1233, 1043, 937, 846, 785 cm^{-1}.

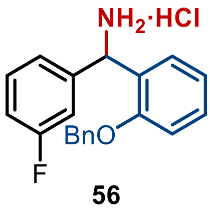

56

(56) (2-(benzyloxy)phenyl)(3-fluorophenyl)methanamine hydrochloride

Prepared according to **General Procedure D** using(*Z*)-*N'*-((2-(benzyloxy)phenyl)(3-fluorophenyl) methylene)-2-(trifluoromethyl) benzenesulfonohydrazide **S56'** (0.3 mmol, 158.4 mg) and $NH_3{\cdot}H_2O$ (308 μL, 2.4 mmol) afforded compound **56** (66.1 mg, 68% yield) as a white solid (mp: 153-165 °C). **^{1}H NMR** (500 MHz, DMSO) δ 9.34 (s, 3H), 7.77 (d, *J* = 7.5 Hz, 1H), 7.43-7.38 (m, 1H), 7.38-7.29 (m, 7H), 7.27 (d, *J* = 7.5 Hz, 1H), 7.20-7.15 (m, 1H), 7.12 (d, *J* = 8.5 Hz, 1H), 7.05 (t, *J* = 8.0 Hz, 1H), 5.77 (s, 1H), 5.15 (s, 2H). **^{13}C NMR** (126 MHz, DMSO) δ 162.8 (d, *J* = 244.0 Hz), 155.6, 141.3 (d, *J* = 7.4 Hz), 137.6, 131.5 (d, *J* = 8.2 Hz), 130.7, 129.3, 128.8, 128.5, 128.3, 126.7, 124.9 (d, *J* = 2.1 Hz), 121.7,

116.0 (d, J = 21.0 Hz), 115.7 (d, J = 22.5 Hz), 70.4, 52.4. **^{19}F NMR** (470 MHz, DMSO) δ (-112.21)-(-112.33) (m).

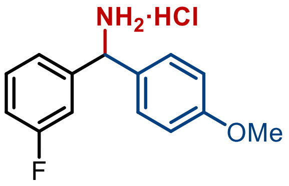

57

(57) (3-fluorophenyl)(4-methoxyphenyl)methanamine hydrochloride

Prepared according to **General Procedure D** using *N'*-((3-fluorophenyl)(4-methoxyphenyl) methylene)-2-(trifluoromethyl) benzenesulfonohydrazide **S57'** (0.3 mmol, 135.6 mg) and $NH_3{\cdot}H_2O$ (308 μL, 2.4 mmol) afforded compound **57** (53.0 mg, 70% yield) as a white solid (mp: 154-156 °C). **^{1}H NMR** (500 MHz, DMSO) δ 9.28 (s, 3H), 7.54-7.42 (m, 4H), 7.38 (d, J = 6.5 Hz, 1H), 7.38 (d, J = 7.0 Hz, 1H), 7.17 (t, J = 7.0 Hz, 1H), 6.96 (d, J = 8.0 Hz, 2H), 5.61 (s, 1H), 3.74 (s, 3H). **^{13}C NMR** (126 MHz, DMSO) δ 162.9 (d, J = 244.0 Hz), 160.1, 142.3 (d, J = 6.3 Hz), 131.7 (d, J = 7.6 Hz), 130.8, 129.8, 124.3 (d, J = 1.3 Hz), 115.8 (d, J = 21.4 Hz), 115.1 (d, J = 22.4 Hz), 115.0, 56.9, 56.1. **^{19}F NMR** (470 MHz, DMSO) δ (-112.21)-(-112.33) (m).

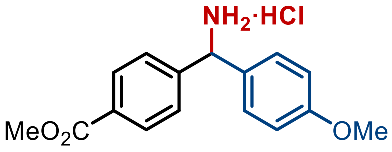

58

(58) methyl 4-(amino(4-methoxyphenyl)methyl)benzoate hydrochloride

Prepared according to **General Procedure D** using methyl 4-((4-methoxyphenyl) (2-((2-(trifluoromethyl)phenyl)sulfonyl)hydrazono)methyl) benzoate **S58'** (0.3 mmol, 147.7 mg) and $NH_3{\cdot}H_2O$ (308 μL, 2.4 mmol) afforded compound **58** (80.3 mg, 87% yield) as a white solid (mp: 173-175 °C). **^{1}H NMR** (500 MHz, DMSO) δ 9.32 (s, 3H), 7.97 (d, J = 8.0 Hz, 2H), 7.70 (d, J = 8.0 Hz, 2H), 7.47 (d, J = 8.5 Hz, 2H), 6.96 (d, J = 8.5 Hz, 2H), 5.69 (q, J = 5.0 Hz, 1H), 3.84 (s, 3H), 3.73 (s, 3H). **^{13}C NMR** (126 MHz, DMSO) δ 166.8, 160.2, 144.7, 130.7, 130.4, 130.2, 130.0, 128.5, 115.1, 57.2, 56.2, 53.2. **IR** (Film): 3472, 3002, 2970, 2657, 2249, 2124, 1672, 1281, 1053, 1025, 1006, 823, 775, 726 cm^{-1}.

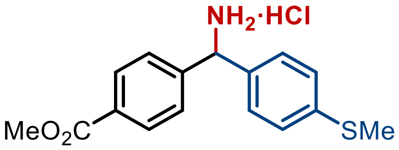

59

(59) methyl 4-(amino(4-(methylthio)phenyl)methyl)benzoate hydrochloride

Prepared according to **General Procedure D** using 4-((4-(methylthio)phenyl)(2-((2-(trifluoromethyl) phenyl)sulfonyl)hydrazono)methyl) benzoate **S59'** (0.3 mmol, 152.6 mg) and $NH_3{\cdot}H_2O$ (308 μL, 2.4

mmol) afforded compound **59** (66.1 mg, 68% yield) as a white solid (mp: 171-173 ºC). **^{1}H NMR** (500 MHz, DMSO) δ 9.41 (s, 3H), 7.98 (d, *J* = 8.5 Hz, 2H), 7.72 (d, *J* = 8.0 Hz, 2H), 7.50 (d, *J* = 8.0 Hz, 2H), 7.29 (d, *J* = 8.5 Hz, 2H), 5.72 (s, 1H), 3.84 (s, 3H), 2.45 (s, 3H). **^{13}C NMR** (126 MHz, DMSO) δ 166.7, 144.4, 139.8, 135.1, 130.4, 130.3, 129.1, 128.7, 126.9, 57.2, 53.2, 15.4. **IR** (Film): 3441, 3043, 2976, 2657, 2249, 2124, 1720, 1281, 1053, 1025, 1006, 819, 775, 726 cm^{-1}.

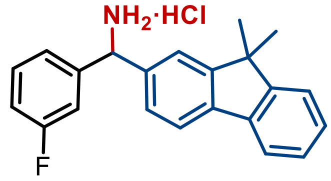

60

(60) (9,9-dimethyl-9*H*-fluoren-2-yl)(3-fluorophenyl)methanamine hydrochloride

Prepared according to **General Procedure D** using *N*'-((3-fluorophenyl)(4-methoxyphenyl) methylene)-2-(trifluoromethyl) benzenesulfonohydrazide **S60'** (0.3 mmol, 161.6 mg) and $NH_3{\cdot}H_2O$ (308 μL, 2.4 mmol) afforded compound **60** (98.7 mg, 93% yield) as a white solid (mp: 161-163 ºC). **^{1}H NMR** (500 MHz, DMSO) δ 9.34 (s, 3H), 7.93 (s, 1H), 7.87 (d, *J* = 8.0 Hz, 1H), 7.85-7.82 (m, 1H), 7.59-7.51 (m, 3H), 7.50-7.44 (m, 2H), 7.38-7.30 (m, 2H), 7.20 (t, *J* = 8.0 Hz, 1H), 5.73 (s, 1H), 1.434 (s, 3H), 1.427 (s, 3H). **^{13}C NMR** (126 MHz, DMSO) δ 163.0 (d, *J* = 244.0 Hz), 154.8, 154.4, 142.2 (d, *J* = 7.0 Hz), 139.7, 138.7, 137.9, 131.9 (d, *J* = 8.2 Hz), 128.7, 128.1, 127.3, 124.5 (d, *J* = 2.6 Hz), 123.8, 123.0, 121.4, 116.0 (d, *J* = 21.4 Hz), 115.2 (d, *J* = 22.7 Hz), 57.9, 47.5, 27.79, 27.76. **^{19}F NMR** (470 MHz, DMSO) δ (-112.21)-(-112.33) (m). **IR** (Film): 3481, 2248, 1053, 2014, 1005, 820, 757 cm^{-1}.

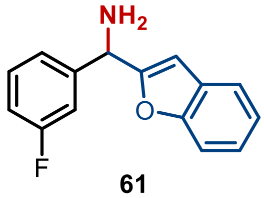

61

(61) benzofuran-2-yl(3-fluorophenyl)methanamine

Prepared according to **General Procedure C** using *N*'-(benzofuran-2-yl(3-fluorophenyl) methylene)-2-(trifluoromethyl) benzenesulfonohydrazide **S61'** (0.3 mmol, 138.7 mg) and $NH_3{\cdot}H_2O$ (308 μL, 2.4 mmol) afforded compound **61** (49.2 mg, 68% yield) as a colourless oil. **^{1}H NMR** (500 MHz, $CDCl_3$) δ 7.52 (d, *J* = 7.5 Hz, 1H), 7.43 (d, *J* = 8.0 Hz, 1H), 7.37-7.30 (m, 1H), 7.26-7.17 (m, 4H), 7.01 (td, *J* = 8.5, 2.5 Hz, 1H), 6.55 (s, 1H), 5.34 (s, 1H), 2.14 (s, 2H). **^{13}C NMR** (126 MHz, $CDCl_3$) δ 163.0 (d, *J* = 246.5 Hz), 159.6, 155.0, 144.2 (d, *J* = 6.8 Hz), 131.2, 130.2 (d, *J* = 8.1 Hz), 128.1, 124.1, 122.83, 122.76 (d, *J* = 3.0 Hz), 121.0, 114.8 (d, *J* = 21.2 Hz), 114.1 (d, *J* = 22.2 Hz), 111.2, 103.0, 54.0. **^{19}F NMR** (564 MHz, $CDCl_3$) δ (-112.46)-(-112.58) (m). **IR** (Film): 3427, 2986, 2253, 1264, 904, 725 cm^{-1}.

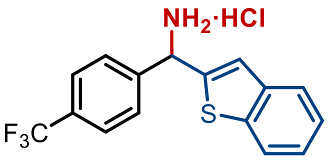

62

(62) benzo[*b*]thiophen-2-yl(3-(trifluoromethyl)phenyl)methanamine hydrochloride

Prepared according to **General Procedure D** using *N'*-(benzo[*b*]thiophen-2-yl(4-(trifluoromethyl) phen-yl)methylene)-2-(trifluoromethyl) benzenesulfonohydrazide **S62'** (0.3 mmol, 158.5 mg) and $NH_3{\cdot}H_2O$ (308 μL, 2.4 mmol) afforded compound **62** (83.5 mg, 81% yield) as a white solid (mp: 148-150 °C). **^{1}H NMR** (500 MHz, DMSO) δ 9.69 (s, 3H), 7.97-7.83 (m, 6H), 7.72 (s, 1H), 7.44-7.32 (m, 2H), 6.22 (s, 1H). **^{13}C NMR** (126 MHz, DMSO) δ 142.8, 142.0, 140.3, 139.7, 130.2 (q, *J* = 31.5 Hz), 129.5, 126.7 (q, *J* = 3.8 Hz), 126.0, 125.8, 125.01, 124.96, 124.95 (q, *J* = 272.2 Hz), 123.5, 53.6. **^{19}F NMR** (470 MHz, DMSO) δ -61.14. **IR** (Film): 3477, 2249, 2123, 1052, 1024, 1005, 820, 757 cm^{-1}.

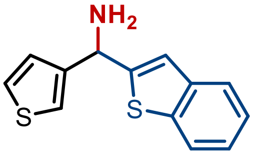

63

(63) benzo[*b*]thiophen-2-yl(thiophen-3-yl)methanamine

Prepared according to **General Procedure C** using (*Z*)-*N'*-(benzo[*b*]thiophen-2-yl(thiophen-3-yl) methylene)-2-(trifluoromethyl) benzenesulfonohydrazide **S63'** (0.3 mmol, 140.0 mg) and $NH_3{\cdot}H_2O$ (308 μL, 2.4 mmol) afforded compound **63** (46.4 mg, 63% yield) as a colourless oil. **^{1}H NMR** (500 MHz, $CDCl_3$) δ 7.78 (d, *J* = 7.5 Hz, 1H), 7.67 (d, *J* = 7.5 Hz, 1H), 7.33-7.28 (m, 3H), 7.26 (s, 1H), 7.13 (s, 1H), 7.11 (dd, *J* = 4.5, 1.0 Hz, 1H), 5.57 (s, 1H), 1.94 (s, 2H). **^{13}C NMR** (126 MHz, $CDCl_3$) δ 150.9, 145.6, 139.6, 139.5, 126.5, 126.2, 124.2, 124.0, 123.3, 122.4, 121.2, 120.3, 52.6. **IR** (Film): 3402, 3006, 2952, 2253, 902, 723 cm^{-1}.

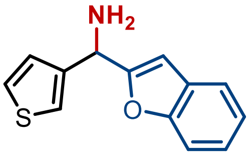

64

(64) benzofuran-2-yl(thiophen-3-yl)methanamine

Prepared according to **General Procedure C** using *N'*-(benzofuran-2-yl(thiophen-3-yl) methylene)-2-(trifluoromethyl) benzenesulfonohydrazide **S64'** (0.3 mmol, 135.1 mg) and $NH_3{\cdot}H_2O$ (308 μL, 2.4 mmol) afforded compound **64** (47.5 mg, 69% yield) as a colourless oil. **^{1}H NMR** (600 MHz, $CDCl_3$) δ 7.51 (d, *J* = 7.8 Hz, 1H), 7.43 (d, *J* = 7.8 Hz, 1H), 7.33-7.28 (m, 2H), 7.24 (d, *J* = 7.2 Hz, 1H), 7.20 (t, *J* = 7.2 Hz, 1H), 7.13 (d, *J* = 4.8 Hz, 1H), 6.51 (s, 1H), 5.38 (s, 1H), 2.15 (s, 2H). **^{13}C NMR** (126 MHz, $CDCl_3$) δ 160.2, 154.9, 143.0, 128.2, 126.7, 126.1, 124.0, 122.7, 121.7, 120.9, 111.2, 102.4, 50.4. **IR** (Film): 3372, 3106, 2922, 1454, 906, 730 cm^{-1}.

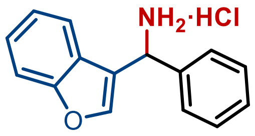

65

(65) benzofuran-3-yl(phenyl)methanamine hydrochloride

Prepared according to **General Procedure D** using *N*'-(benzofuran-3-yl(phenyl) methylene)-2-(*tri*-fluoromethyl) benzenesulfonohydrazide **S65'** (0.3 mmol, 133.3 mg) and $NH_3 \cdot H_2O$ (308 μL, 2.4 mmol) afforded compound **65** (51.4 mg, 66% yield) as a white solid (mp: 162-164 °C). **^{1}H NMR** (600 MHz, DMSO) δ 9.57 (s, 3H), 7.70-7.63 (m, 3H), 7.55 (d, J = 8.4 Hz, 1H), 7.49-7.39 (m, 3H), 7.31 (t, *J* = 7.8 Hz, 1H), 7.26 (t, *J* = 7.8 Hz, 1H), 7.07 (s, 1H), 5.90 (s, 1H). **^{13}C NMR** (151 MHz, DMSO) δ 155.2, 154.4, 136.2, 130.0, 129.8, 129.0, 128.3, 125.9, 124.3, 122.6, 112.1, 106.6, 52.4. **IR** (Film): 3442, 3052, 2361, 2253, 1264, 904, 725 cm^{-1}.

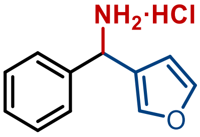

66

(66) furan-3-yl(phenyl)methanamine hydrochloride

Prepared according to **General Procedure D** using *N*'-(furan-3-yl(phenyl) methylene)-2-(trifluoromethyl) benzenesulfonohydrazide **S66'** (0.3 mmol, 118.3 mg) and $NH_3 \cdot H_2O$ (308 μL, 2.4 mmol) afforded compound **66** (48.4 mg, 77% yield) as a white solid (mp: 166-168 °C). **^{1}H NMR** (500 MHz, DMSO) δ 9.20 (s, 3H), 7.64 (s, 1H), 7.61-7.54 (m, 3H), 7.42 (t, *J* = 6.5 Hz, 2H), 7.38-7.33 (m, 1H), 7.26 (d, *J* = 3.5 Hz, 1H), 5.67 (s, 1H). **^{13}C NMR** (151 MHz, DMSO) δ 140.2, 138.9, 129.6, 129.2, 128.3, 128.2, 127.8, 124.6, 54.2. **IR** (Film): 3445, 2248, 1052, 1024, 1005, 820, 757 cm^{-1}.

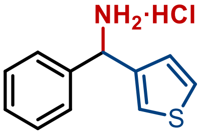

67

(67) phenyl(thiophen-3-yl)methanamine hydrochloride

Prepared according to **General Procedure D** using *N'*-(phenyl(thiophen-3-yl) methylene)-2-(trifluoromethyl) benzenesulfonohydrazide **S67'** (0.3 mmol, 123.1 mg) and $NH_3 \cdot H_2O$ (308 μL, 2.4 mmol) afforded compound **67** (42.7 mg, 63% yield) as a white solid (mp: 159-161 °C). **^{1}H NMR** (500 MHz, DMSO) δ 9.13 (s, 3H), 7.69 (d, *J* = 0.5 Hz, 2H), 7.58 (d, *J* = 7.5 Hz, 2H), 7.43 (t, *J* = 7.0 Hz, 2H), 7.37 (t, *J* = 7.0 Hz, 1H), 6.70 (s, 1H), 5.52 (q, *J* = 4.5 Hz, 1H). **^{13}C NMR** (151 MHz, DMSO) δ 145.0, 141.8, 138.6, 129.7, 129.4, 128.4, 124.6, 110.8, 50.7. **IR** (Film): 3439, 2249, 2124, 1052, 1024, 1005, 820, 758 cm^{-1}.

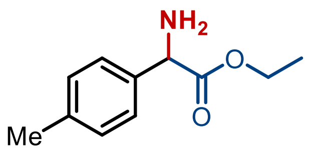

68

(68) ethyl 2-amino-2-(p-tolyl)acetate

Prepared according to **General Procedure C** using ethyl (*Z*)-2-(*p*-tolyl)-2-(2-((2-(trifluoromethyl) phenyl)sulfonyl)hydrazineylidene) acetate **S68'** (0.3 mmol, 124.2 mg) and $NH_3{\cdot}H_2O$ (308 μL, 2.4 mmol) afforded compound **68** (46.9 mg, 81% yield) as a colourless oil. **^{1}H NMR** (500 MHz, $CDCl_3$) δ 7.26 (d, *J* = 8.0 Hz, 2H), 7.16 (d, *J* = 8.0 Hz, 2H), 4.55 (s, 1H), 4.24-4.07 (m, 2H), 2.34 (s, 3H), 1.93 (s, 2H), 1.21 (t, *J* = 7.5 Hz, 3H). **^{13}C NMR** (126 MHz, $CDCl_3$) δ 174.1, 137.6, 137.5 129.4, 126.6, 61.2, 58.5, 21.1, 14.0.

Spectroscopic data are in agreement with those reported in the literature.[5]

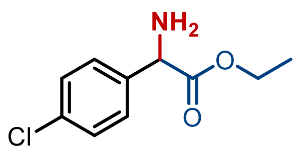

69

(69) ethyl 2-amino-2-(4-chlorophenyl)acetate

Prepared according to **General Procedure C** using ethyl 2-(4-chlorophenyl)-2-(2-((2-(trifluoromethyl) phenyl)sulfonyl)hydrazono) acetate **S69'** (0.3 mmol, 130.4 mg) and $NH_3{\cdot}H_2O$ (308 μL, 2.4 mmol) afforded compound **69** (50.0 mg, 78% yield) as a colourless oil. **^{1}H NMR** (500 MHz, $CDCl_3$) δ 7.33-7.30 (s, 4H), 4.58 (s, 1H), 4.24-4.09 (m, 2H), 1.86 (s, 2H), 1.21 (t, *J* = 7.0 Hz, 3H). **^{13}C NMR** (126 MHz, $CDCl_3$) δ 173.6, 138.8, 133.8, 128.8, 128.2, 61.5, 58.1, 14.0. **HRMS** (ESI) m/z calcd for $C_{10}H_{12}ClNNaO_2$ $[M+Na]^+$ 236.0449, Found: 236.0438.
Spectroscopic data are in agreement with those reported in the literature.[5]

---

4. Preparation of Starting Materials

4.1 Synthesis and analytical data of new diazo compounds

<u>Route A</u>

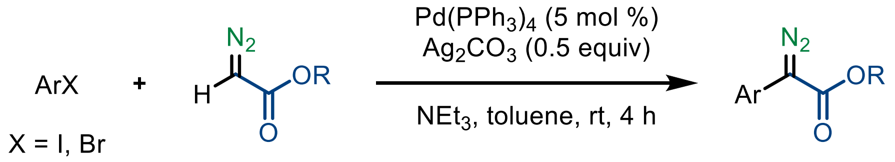

According to the literature,[8] tetrakis(triphenylphosphine)palladium (0.25 mmol, 0.3 g), iodobenzene (5 mmol), silver carbonate (2.5 mmol, 0.7 g) and triethylamine (6.5 mmol, 0.9 mL) suspended in toluene (20 mL) in a 50 mL Schlenk tube under nitrogen. ethyl diazoacetate (6.5 mmol) was then added. The resulting solution was stirred at room temperature for 4 hours, then filtered through a short path of silica gel and eluted with ethyl acetate. The volatile compounds were removed in vacuo and the residue was purified by column chromatography (petroleum ether: ethyl acetate = 10:1) to obtain the product α-aryl diazoester.

Route B

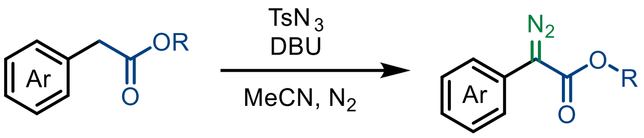

According to the literature,[9] the ester (5.0 mmol) and tosyl azide (15.0 mmol) were added to a mixture in anhydrous MeCN (15.0 mL) followed by slow addition of 1,8-diazabicyclo[5.4.0]undecane yl-7-ene (DBU) (1.1 mL, 1.1 g, 7.5 mmol). The reaction mixture was stirred at room temperature overnight. After complete consumption of starting material, the reaction mixture was quenched with saturated aqueous NH_4Cl, extracted with CH_2Cl_2, washed with brine, dried over $MgSO_4$, and concentrated under reduced pressure to give the product. The residue was purified by flash chromatography (hexane/EtOAc, 9:1) to give the α-diazo ester.

Route C

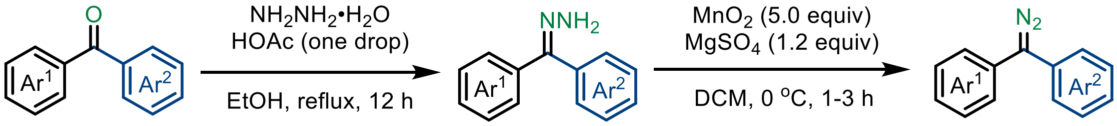

Diaryl ketones were synthesized were prepared according to reported literature.[10]

A 100 mL round-bottom flask containing diaryl ketone (5.0 mmol, 1.0 Equiv), hydrazine hydrate (50.0 mmol, 10.0 Equiv), HOAc (one drop) and ethanol (10 mL) was refluted for 12 h. After cooling to room temperature, the solvent EtOH was removed in vacuum to obtain the crude product. The crude product was purified by silica gel column chromatography to obtain the desired diaryl hydrazone.

Diaryl hydrazone (2.0 mmol, 1.0 equiv) was dissolved in 15 mL DCM in a 50 mL round-bottom flask. After cooling to 0 °C, anhydrous $MgSO_4$ (2.4 mmol, 1.2 equiv) and activated MnO_2 (10.0 mmol, 5.0 equiv) were added to the stirring mixture. The reaction mixture was maintained at 0 °C for another hour. After the reaction is complete, the reaction mixture is leached out as a solid with diatomite. The solvent was removed under reduced pressure and the crude product was obtained. The crude product was purified by silica gel column chromatography to obtain the desired diaryl diazomethane.

Route D

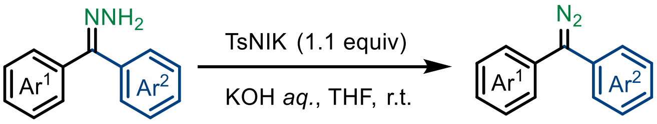

According to the literature,[11] a suspension of potassium *N*-iodo *p*-toluenesulfonamide[12] (TSNIK, 369.0 mg, 1.1 mmol) in a solution of hydrazone in THF (1.0 mmol in 4.0 mL) was prepared. THF was added to a mixture of the hydrazone and potassium *N*-iodo *p*-toluenesulfonamide. Aqueous potassium hydroxide was slowly added to the THF suspension (so that the final volume ratio KOH: THF was equal to 1:4). the reaction was complete after stirring for 1 h at room temperature. The mixture was poured into aqueous potassium hydroxide and extracted with ether. The ethereal phase was washed with aqueous potassium hydroxide, saturated brine and dried over $MgSO_4$. Removal of the solvent under reduced pressure and purified by column chromatography to obtain the desired diaryl diazomethane.

Analytical data of new diazo compounds

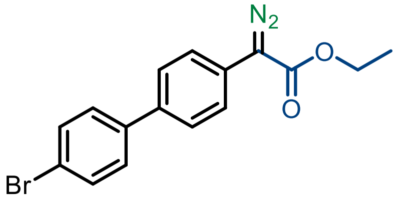

S16

Orange yellow oil, yield: 85%. **^{1}H NMR** (500 MHz, $CDCl_3$) δ 7.59-7.53 (m, 6H), 7.47–7.42 (m, 2H), 4.36 (q, *J* = 7.0 Hz, 2H), 1.36 (t, *J* = 7.0 Hz, 3H). **^{13}C NMR** (126 MHz, $CDCl_3$) δ 165.1, 139.2, 137.2, 132.0, 128.4, 127.3, 125.1, 124.3, 121.6, 61.1, 14.5. **HRMS** (ESI) m/z calculated for $C_{16}H_{13}BrN_2NaO_2$ $[M+Na]^+$ 367.0058, found 367.0060.

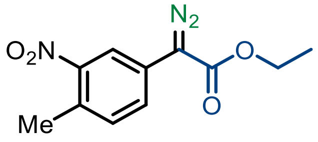

S20

Orange yellow oil, yield: 84%. **^{1}H NMR** (600 MHz, $CDCl_3$) δ 8.12 (d, *J* = 1.8 Hz, 1H), 7.60 (dd, *J* = 8.4 Hz, 1.8 Hz, 1H), 7.34 (d, *J* = 8.4 Hz, 1H), 4.36 (q, *J* = 7.2 Hz, 2H), 2.57 (s, 3H), 1.36 (t, *J* = 7.2 Hz, 3H). **^{13}C NMR** (151 MHz, $CDCl_3$) δ 164.3, 149.5, 133.2, 130.3, 127.4, 125.5, 119.3, 61.3, 19.9, 14.4.

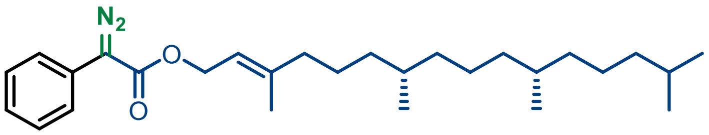

S70

(S70) α-Aryl diazoester were synthesized were prepared according to **route B**. **^{1}H NMR** (500 MHz, $CDCl_3$) δ 7.48 (d, *J* = 7.5 Hz, 2H), 7.37 (t, *J* = 8.0 Hz, 2H), 7.17 (t, *J* = 7.0 Hz, 1H), 5.39 (t, *J* = 7.0 Hz, 1H), 4.79 (d, *J* = 7.0 Hz, 2H), 2.06-1.98 (m, 2H), 1.73 (s, 3H), 1.55-1.48 (m, 1H), 1.44-1.33 (m, 4H), 1.30-1.20 (m, 8H), 1.18-1.09 (m, 3H), 1.09-1.01 (m, 3H), 0.88-0.82 (m, 12H). **^{13}C NMR** (126 MHz, $CDCl_3$) δ 165.3, 143.1, 128.9, 125.7, 124.0, 118.1, 61.9, 39.9, 39.4, 37.44, 37.38, 37.3, 36.6, 32.8, 32.7, 28.0, 25.0, 24.8, 24.5, 22.7, 22.6, 19.8, 19.7, 16.5. **HRMS** (ESI) m/z calculated for $C_{28}H_{44}N_2O_2Na$ $[M+Na]^+$ 463.3300, found 463.3322.

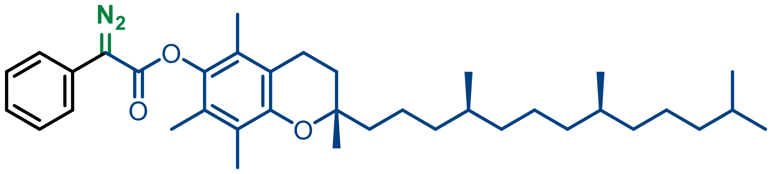

S71

(S71) α-Aryl diazoester were synthesized were prepared according to **route B**. **^{1}H NMR** (500 MHz, $CDCl_3$) δ 7.59-7.55 (m, 2H), 7.41 (t, *J* = 8.0 Hz, 2H), 7.21 (t, *J* = 7.5 Hz, 1H), 2.61 (t, *J* = 7.0 Hz, 2H), 2.11 (s, 3H), 2.09 (s, 3H), 2.05 (s, 3H), 1.87-1.72 (m, 2H), 1.59-1.50 (m, 3H), 1.46-1.21 (m, 15H), 1.17-1.02 (m, 6H), 0.89-0.82 (m, 12H). **^{13}C NMR** (126 MHz, $CDCl_3$) δ 163.7, 149.6, 140.0, 129.0, 127.1, 125.9, 125.4, 125.3, 123.9, 123.2, 117.5, 75.1, 39.4, 37.4, 37.3, 32.8, 32.7, 31.2, 28.0, 24.8, 24.4, 22.7, 22.6, 21.0, 20.6, 19.74, 19.65, 13.1, 12.2, 11.8. **HRMS** (ESI) m/z calculated for $C_{37}H_{54}N_2NaO_3$ $[M+Na]^+$ 597.4032, found 597.4036.

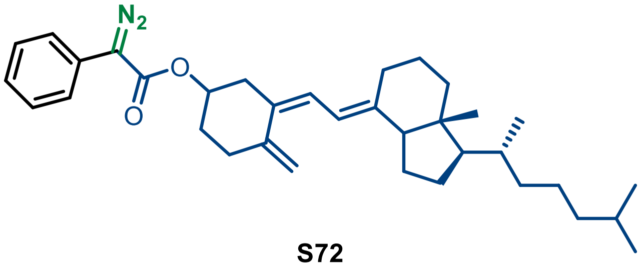

S72

(S72) α-Aryl diazoester were synthesized were prepared according to **route B**. **^{1}H NMR** (500 MHz, $CDCl_3$) δ 7.39 (d, *J* = 7.5 Hz, 2H), 7.29 (t, *J* = 8.0 Hz, 2H), 7.09 (t, *J* = 7.5 Hz, 1H), 6.18 (d, *J* = 11.0 Hz, 1H), 5.97 (d, *J* = 11.0 Hz, 1H), 5.13-5.07 (m, 1H), 5.01 (d, *J* = 2.1 Hz, 1H), 4.79 (d, *J* = 2.1 Hz, 1H), 2.77-2.71 (m, 1H), 2.59 (dd, *J* = 13.4, 3.5 Hz, 1H), 2.44-2.30 (m, 2H), 2.18 (ddd, *J* = 13.5, 8.5, 4.5 Hz, 1H), 2.00-1.88 (m, 3H), 1.81-1.75 (m, 2H), 1.66-1.55 (m, 2H), 1.47-1.39 (m, 3H), 1.34-1.16 (m, 7H), 1.13-1.00 (m, 3H), 0.97-0.90 (m, 1H), 0.85 (d, *J* = 6.4 Hz, 3H), 0.82-0.75 (m, 6H), 0.48 (s, 3H). **^{13}C NMR** (126 MHz, $CDCl_3$) δ 164.8, 144.6, 142.6, 134.1, 128.96, 128.91, 125.7, 124.0, 122.8, 117.5, 112.8, 72.7, 56.6, 56.4, 45.9, 42.4, 40.6, 39.5, 36.2, 32.06, 32.05, 29.1, 28.0, 27.7, 23.9, 23.6, 22.8, 22.6, 22.3, 18.9, 12.0.

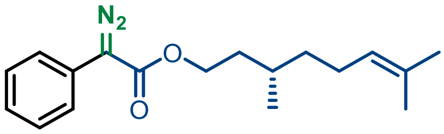

S73

(S73) α-Aryl Diazoester were synthesized were prepared according to **route B**. **^{1}H NMR** (500 MHz, $CDCl_3$) δ 7.51-7.47 (m, 2H), 7.41-7.36 (m, 2H), 7.21-7.16 (m, 1H), 5.12-5.07 (m, 1H), 4.36-4.27 (m, 2H), 2.08-1.91 (m, 2H), 1.79-1.71 (m, 1H), 1.68 (d, *J* = 0.8 Hz, 3H), 1.64-1.56 (m, 4H), 1.55-1.47 (m, 1H), 1.42-1.33 (m, 1H), 1.26-1.17 (m, 1H), 0.94 (d, *J* = 6.5 Hz, 3H). **^{13}C NMR** (126 MHz, $CDCl_3$) δ 165.3, 131.4, 128.9, 125.7, 125.6, 124.5, 123.9, 63.5, 37.0, 35.6, 29.5, 25.7, 25.4, 19.4, 17.7.

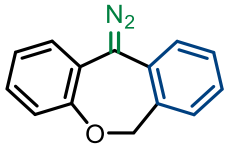

S74

(S74) Diazoester were synthesized were prepared according to the literature[13], Orange yellow oil, yield: 45%. **^{1}H NMR** (500 MHz, $CDCl_3$) δ 7.32 (t, *J* = 8.0 Hz, 1H), 7.17 (d, *J* = 7.5 Hz, 1H), 7.09-7.01 (m, 3H), 6.99-6.96 (m, 2H), 6.94-6.91 (m, 1H), 5.00 (s, 2H). **^{13}C NMR** (126 MHz, $CDCl_3$) δ 157.2, 134.9, 129.6, 128.9, 128.7, 125.1, 124.8, 123.5, 123.3, 123.0, 121.2, 117.8, 74.8.

4.2 Synthesis and analytical data of *N*-triftosylhydrazones

Route E.

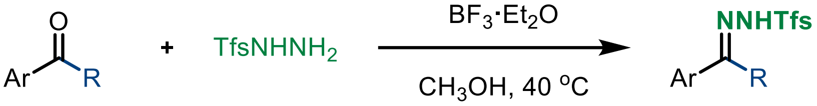

According to the literature,[14] to a stirred solution of $TfsNHNH_2$ (2.0 mmol, 1.2 equiv) in CH_3OH (2 mL) were added aryl ketone (2.2 mmol, 1.0 equiv) and boron trifluoride diethyl etherate, then the mixture was stirred at 40 °C. The progress of the reaction was monitored by TLC. Upon consumption of the starting material, the solvent was removed in vacuo and the residue was purified by flash chromatography on silica gel to afford the *N*-triftosylhydrazones. The yields were around 50-90% in general.

Note: Part of the N-triftosylhydrazone was obtained as two isomers, which was used in the next step without further purification. Both isomers can be used as donor/donore carbene precursor under standard conditions.

Analytical data of *N*-triftosylhydrazones

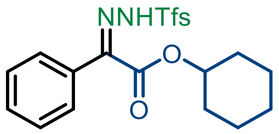

S32'

(S32') Light yellow oil, m.p. 148-150 °C, yield: 80%. **^{1}H NMR** (600 MHz, $CDCl_3$) δ 11.81 (s, 1H), 8.40-8.33 (m, 1H), 7.84-7.82 (m, 1H), 7.70-7.66 (m, 2H), 7.50-7.47 (m, 2H), 7.33-7.28 (m, 3H), 5.08-5.04 (m, 1H), 1.93-1.86 (m, 2H), 1.69-1.63 (m, 2H), 1.54-1.48 (m, 3H), 1.42-1.34 (m, 2H), 1.30-1.22(m, 1H). **^{13}C NMR** (151 MHz, $CDCl_3$) δ 161.2, 134.0, 136.6, 133.5, 132.8, 132.3, 129.4, 128.3, 128.2 (q, *J* = 6.3 Hz), 127.8, 127.6 (q, *J* = 33.3 Hz), 122.6 (q, *J* = 274.0 Hz), 75.7, 31.0, 24.8, 23.2. **^{19}F NMR** (470 MHz, $CDCl_3$) δ -58.00. **HRMS** (ESI) m/z calculated for $C_{21}H_{21}F_3N_2O_4SNa$ $[M+Na]^+$ 477.1083, found 477.1085.

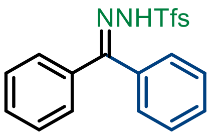

S33'

(**S33'**) White solid, m.p. 180-184 °C, yield: 90%. **^{1}H NMR** (500 MHz, DMSO) δ 10.92 (s, 1H), 8.10 (d, *J* = 7.6 Hz, 1H), 8.04 (d, *J* = 7.6 Hz, 1H), 7.95 (t, *J* = 7.6 Hz, 1H), 7.88 (t, *J* = 7.6 Hz, 1H), 7.60-7.53 (m, 3H), 7.40-7.35 (m, 1H), 7.34-7.27 (m, 4H), 7.25-7.20 (m, 2H). **^{13}C NMR** (126 MHz, DMSO) δ 154.9, 138.3, 136.9, 133.3, 133.2, 132.4, 130.9, 129.9, 129.6, 129.1, 128.7, 128.4, 128.3 (d, *J* = 6.3 Hz), 127.2, 126.7 (d, *J* = 32.8 Hz), 122.8 (d, *J* = 274.8 Hz). **^{19}F NMR** (470 MHz, DMSO) δ -58.21. Spectroscopic data in agreement with those reported in literature (*31*).

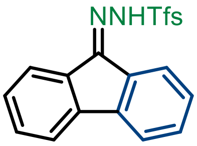

S37'

(S37') White solid, m.p. 203-207 °C, yield: 87%. **^{1}H NMR** (500 MHz, DMSO) δ 12.04 (s, 1H), 8.35 (d, *J* = 8.0 Hz, 1H), 8.27 (d, *J* = 7.5 Hz, 1H), 8.09 (d, *J* = 8.0 Hz, 1H), 7.99 (t, *J* = 7.8 Hz, 1H), 7.95-7.86 (m, 2H), 7.81 (d, *J* = 7.5 Hz, 1H), 7.60-7.55 (m, 1H), 7.53-7.48 (m, 2H), 7.46-7.42 (m, 1H), 7.33-7.28 (m, 1H). **^{13}C NMR** (126 MHz, DMSO) δ 152.9, 142.2, 140.2, 138.1, 136.3, 134.0, 133.6, 132.5, 131.8, 131.4, 129.5, 128.8, 128.71 (q, *J* = 6.6 Hz), 128.70, 128.5, 127.7 (q, *J* = 32.9 Hz), 123.3 (q, *J* = 274.7 Hz), 122.0, 121.1, 120.9. **^{19}F NMR** (470 MHz, DMSO) δ -55.72. **HRMS** (ESI) m/z calculated for $C_{20}H_{13}F_{3}N_{2}O_{2}SNa$ [M+Na]$^{+}$ 425.0655, found 425.0658.

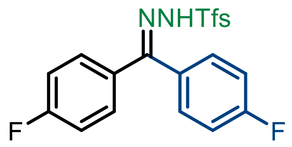

S40'

(S40') White solid, m.p. 160-162 °C, yield: 82%; **^{1}H NMR** (500 MHz, $CDCl_3$) δ 8.50 (d, *J* = 8.0 Hz, 1H), 7.89 (d, *J* = 7.5 Hz, 1H), 7.82 (d, *J* = 7.5 Hz, 1H), 7.79-7.72 (m, 2H), 7.34-7.30 (m, 2H), 7.29-7.23 (m, 2H), 7.19-7.13 (m, 2H), 6.94 (t, *J* = 8.7 Hz, 2H). **^{13}C NMR** (151 MHz, $CDCl_3$) δ 163.9 (d, *J* = 251.4 Hz), 163.6 (d, *J* = 251.4 Hz), 152.9, 136.8, 133.7, 133.5, 132.4, 132.3 (d, *J* = 3.0 Hz), 130.4 (d, *J* = 9.1 Hz), 129.4 (d, *J* = 9.1 Hz), 128.4 (q, *J* = 6.0 Hz), 127.7 (q, *J* = 31.7 Hz), 126.5 (d, *J* = 4.5 Hz), 122.7 (q, *J* = 273.3 Hz), 117.2 (d, *J* = 22.7 Hz), 115.4 (d, *J* = 22.7 Hz). **^{19}F NMR** (564 MHz, $CDCl_3$) δ -58.31 (s), (-108.64)-(-108.84) (m), (-109.97)-(-110.17) (m). **HRMS** (ESI) m/z calculated for $C_{20}H_{13}F_{5}N_{2}O_{2}SNa$ [M+Na]$^{+}$ 463.1514, found 463.1514.

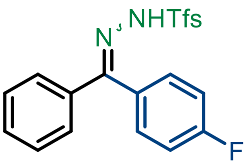

S45'

(S45') White solid, m.p. 158-160 ºC, yield: 85%. major:minor = 6.7:1; **^{1}H NMR** (600 MHz, DMSO) δ 11.22 (s, minor, 1H), 11.10 (s, major, 1H), 8.07 (d, *J* = 7.8 Hz, major, 1H, minor, 1H), 8.05-8.02 (m, major, 1H, minor, 1H), 7.94 (t, *J* = 7.8 Hz, major, 1H, minor, 1H), 7.88 (t, *J* = 7.8 Hz, major, 1H, minor, 1H), 7.65-7.62 (m, minor, 2H), 7.60-7.54 (m, major, 3H), 7.42-7.39 (m, major, 2H), 7.38-7.36 (m, minor, 1H), 7.34-7.32 (m, minor, 4H), 7.32-7.28 (m, major, 2H), 7.25-7.21 (m, major, 2H, minor, 2H). **^{13}C NMR** (126 MHz, DMSO) δ 199.9, 163.0 (d, *J* = 249.5 Hz), 153.9, 153.6, 138.2, 136.8, 133.5 (d, *J* = 3.8 Hz), 133.3, 133.2, 132.2, 131.2 (d, *J* = 8.8 Hz), 130.8, 129.9, 129.7, 129.5, 129.4, 129.1, 128.6, 128.4, 128.3 (q, *J* = 6.3 Hz), 127.2, 126.7 (q, *J* = 34.0 Hz), 122.8 (q, *J* = 274.7 Hz), 116.1 (d, *J* = 22.1 Hz), 115.4 (d, *J* = 22.1 Hz). **^{19}F NMR** (470 MHz, DMSO) δ (-56.01)-(-56.04) (m, major + minor), (-111.15)-(-111.30) (m, major), (-111.55)-(-111.70) (m, minor). **HRMS** (ESI) m/z calculated for $C_{20}H_{14}F_4N_2O_2SNa$ $[M+Na]^+$ 445.3814, found 445.3818.

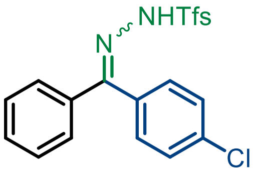

S46'

(**S46'**) White solid, m.p. 170-171 ºC, yield: 85%. major:minor = 2:1; **^{1}H NMR** (500 MHz, DMSO) δ 11.11 (s, minor, 1H), 10.99 (s, major, 1H), 8.10-8.05 (m, major, 1H, minor, 1H), 8.03 (d, *J* = 7.6 Hz, major, 1H, minor, 1H), 7.94 (t, *J* = 7.6 Hz, major, 1H, minor, 1H), 7.88 (t, *J* = 7.6 Hz, major, 1H, minor, 1H), 7.61-7.54 (m, major, 2H, minor, 2H), 7.44-7.21 (m, major, 5H, minor, 7H), 7.20-7.14 (m, major, 2H). **^{13}C NMR** (126 MHz, DMSO) δ 154.0, 138.7, 137.1, 136.3, 135.1, 133.8, 133.7, 131.8, 132.34, 131.30, 131.2, 130.4, 130.2, 129.7, 129.6, 129.3, 129.1, 129.0, 128.9, 128.8 (q, *J* = 6.3 Hz), 127.6, 127.2 (q, *J* = 32.8 Hz), 123.3 (d, *J* = 274.7 Hz). **^{19}F NMR** (470 MHz, DMSO) δ -56.01 (minor), -56.03 (major). **HRMS** (ESI) m/z calculated for $C_{20}H_{14}ClF_3N_2O_2SNa$ $[M+Na]^+$ 461.2753, found 461.2755.

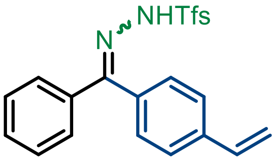

S51'

(S51') White solid, m.p. 160-162 ºC, yield: 81%. major:minor = 1:1; **^{1}H NMR** (500 MHz, $CDCl_3$) δ 8.53-8.49 (m, 2H), 7.87 (d, *J* = 7.5 Hz, 2H), 7.84-7.78 (m, 4H), 7.74 (t, *J* = 7.5 Hz, 2H), 7.59-7.53 (m, 5H), 7.38-7.31 (m, 5H), 7.30-7.23 (m, 4H), 7.15-7.11 (m, 4H), 6.78 (dd, *J* = 17.5, 11.0 Hz, 1H), 6.66 (dd, *J* = 17.5, 11.0 Hz, 1H), 5.88 (d, *J* = 17.5, 1H), 5.74 (d, *J* = 17.5 Hz, 1H), 5.41 (d, *J* = 11.0 Hz, 1H), 5.27 (d, *J* = 11.0 Hz, 1H). **^{13}C NMR** (126 MHz, $CDCl_3$) δ 154.8, 154.7, 139.5, 139.2, 137.0, 136.1,

136.0, 135.7, 135.4, 133.8, 133.7, 133.3, 132.3, 130.8, 130.2, 130.0, 129.9, 129.8, 128.4, 128.3 (q, *J* = 6.3 Hz), 128.2, 128.1, 127.8 (q, *J* = 32.9 Hz), 127.7, 127.6, 127.4, 126.0, 122.70 (d, *J* = 274.7 Hz), 122.69 (d, *J* = 274.7 Hz), 116.1, 115.2. **^{19}F NMR** (470 MHz, $CDCl_3$) δ -58.28 (s), -58.31 (s). **HRMS** (ESI) m/z calculated for $C_{22}H_{17}F_3N_2O_2SNa$ $[M+Na]^+$ 453.2846, found 453.2848.

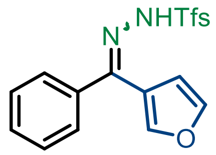

S66'

(S66') White solid, m.p. 147-149 °C, yield: 84%. *Z*: *E* = 1:1; **^{1}H NMR** (500 MHz, $CDCl_3$) δ 8.50 (d, *J* = 7.6 Hz, 1H), 8.47 (d, *J* = 7.6 Hz, 1H), 8.02 (s, 1H), 7.86 (d, *J* = 7.6 Hz, 2H), 7.83-7.77 (m, 2H), 7.76-7.72 (m, 2H), 7.70 (s, 1H), 7.59-7.51 (m, 4H), 7.39-7.30 (m, 5H), 7.28-7.22 (m, 3H), 7.19-7.16 (m, 2H), 6.98-6.96 (m, 1H), 6.95-6.93 (m, 1H). **^{13}C NMR** (151 MHz, $CDCl_3$) δ 151.4, 150.7, 139.7, 136.8, 136.2, 133.7, 133.4, 133.3, 132.4, 132.3, 131.0, 130.6, 130.3, 130.1, 129.7, 128.30 (q, *J* = 6.0 Hz), 128.27, 128.24, 128.19, 127.72, 127.68 (q, *J* = 33.4 Hz), 127.66 (q, *J* = 33.4 Hz), 127.64, 127.4, 127.1, 126.8, 126.1, 125.6, 122.69 (q, *J* = 274.1 Hz), 122.67 (q, *J* = 274.1 Hz). **^{19}F NMR** (564 MHz, $CDCl_3$) δ -58.30 (s), -58.32 (s). **HRMS** (ESI) m/z calculated for $C_{18}H_{13}F_3N_2O_3SNa$ $[M+Na]^+$ 417.1497, found 417.1450.

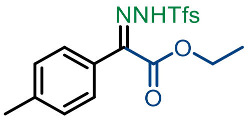

S68'

(S68') White solid, m.p. 160-163 °C, yield: 87%. **^{1}H NMR** (600 MHz, $CDCl_3$) δ 11.64 (s, 1H), 8.41-8.36 (m, 1H), 7.90-7.85 (m, 1H), 7.74-7.70 (m, 2H), 7.37 (d, *J* = 8.4 Hz, 2H), 7.13 (d, *J* = 7.8 Hz, 2H), 4.39 (q, *J* = 7.2 Hz, 2H), 2.34 (s, 3H), 1.35 (t, *J* = 7.2 Hz, 3H). **^{13}C NMR** (126 MHz, $CDCl_3$) δ 162.0, 139.84, 139.75, 136.9, 133.4, 133.1, 132.3, 130.8, 128.8, 128.34 (q, *J* = 6.6 Hz), 128.33, 128.0 (q, *J* = 33.3 Hz), 122.7 (q, *J* = 274.3 Hz), 62.6, 21.3, 14.0. **^{19}F NMR** (564 MHz, $CDCl_3$) δ -58.22. **HRMS** (ESI) m/z calculated for $C_{18}H_{17}F_3N_2O_4SNa$ $[M+Na]^+$ 437.1759, found 427.1760.

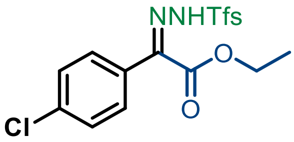

S69'

(S69') White solid, m.p. 123-125 °C, yield: 85%. **^{1}H NMR** (600 MHz, $CDCl_3$) δ 11.87 (s, 1H), 8.39-8.37 (m, 1H), 7.91-7.86 (m, 1H), 7.76-7.71 (m, 2H), 7.42 (d, *J* = 8.4 Hz, 2H), 7.29 (d, *J* = 8.4 Hz, 2H), 4.39 (q, *J* = 7.2 Hz, 2H), 1.35 (t, *J* = 7.2 Hz, 3H). **^{13}C NMR** (126 MHz, $CDCl_3$) δ 161.6, 137.9, 136.8, 135.7, 133.6, 133.0, 132.4, 132.2, 129.8, 128.4 (q, *J* = 6.2 Hz), 128.3, 128.0 (q, *J* = 33.0 Hz), 122.7 (q, *J* = 274.7 Hz), 62.8, 13.9. **^{19}F NMR** (470 MHz, $CDCl_3$) δ -58.21. **HRMS** (ESI) m/z calculated for $C_{17}H_{14}ClF_3N_2O_4SNa$ $[M+Na]^+$ 457.0218, found 457.0214.

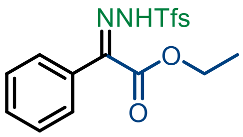

S75'

(S75') White solid, m.p. 148-150 °C, yield: 88%. **^{1}H NMR** (600 MHz, $CDCl_3$) δ 11.76 (s, 1H), 8.40-8.38 (m, 1H), 7.89-7.87 (m, 1H), 7.75-7.71 (m, 2H), 7.49-7.45 (m, 2H), 7.37-7.31 (m, 3H), 4.39 (q, J = 7.2 Hz, 2H), 1.35 (t, J = 7.2 Hz, 3H). **^{13}C NMR** (126 MHz, $CDCl_3$) δ 161.9, 139.5, 136.9, 133.7, 133.5, 133.1, 132.4, 129.5, 128.5, 128.4 (q, J = 6.3 Hz), 128.05, 128.02 (q, J = 33.1 Hz), 122.7 (q, J = 274.7 Hz), 62.6, 13.9. **^{19}F NMR** (470 MHz, $CDCl_3$) δ -58.22. **HRMS** (ESI) m/z calculated for $C_{17}H_{15}F_3N_2O_4SNa$ $[M+Na]^+$ 423.0623, found 423.0622.

---

5. Gram-Scale Reaction and Synthetic Applications

5.1 Gram-scale experiments

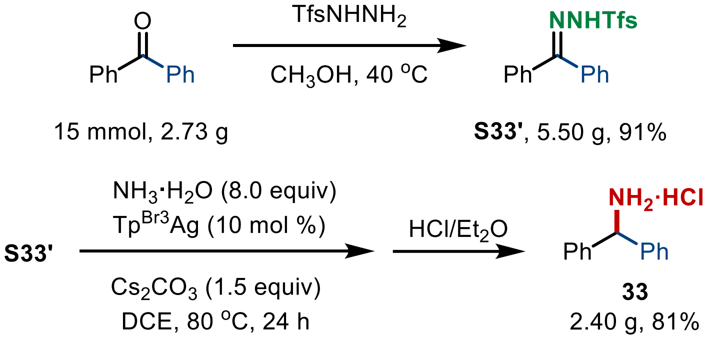

Prpcedure: According to **route E**, to a solution of benzophenone (2.73 g, 15 mmol, 1.0 equiv) in methanol (20 mL) was added *o*-trifluoromethylbenzenesulfonyl hydrazide (3.60 g, 15 mmol, 1.0 equiv). The resulting reaction mixture was stirred at 40 °C for 8 h. After the reaction is complete, the solvent was removed in vacuo. Et_2O (10 mL) and petroleum ether (10 mL) were added inot the residue to precipitate white solid, which was filtered and washed with ice cold diethyl ether and dried under reduced pressure to give *N*-triftosylhydrazone **S33'**.

To a dry Schlenk reaction tube, $Tp^{Br3}Ag(thf)$ (150 mg, 10 mol%) and Cs_2CO_3 (6.65 g, 1.5 equiv) were charged. After sealed the tube was evacuated and backfilled with N_2 for three times, followed by addition of DCE (20 mL) solution of $NH_3{\cdot}H_2O$ (14 mL, 28%-30% wt%, 0.6 mmol, 8.0 equiv) and DCE (20 mL) solution of *N*-triftosylhydrazone **S33'** (13.6 mmol, 1.0 equiv) via syringe. The reaction mixture was tranfered on to preheated oil bath at 80 °C in the dark for 12 h. When the reaction was completed, the reaction was allowed to cool to room temperature, and filtered through a short pad of basic alumina with EtOAc as an eluent. After the solvent was removed under vacuum, the residue was added the ether solution of HCl and stirred for 1 h at room temperature to precipitate a white solid, which was filtered, washed with cold ether and dried under vacuo to yield hydrochloride of N–H insertion product **33**.

5.2 Late-stage modification of bioactive and drugs molecules

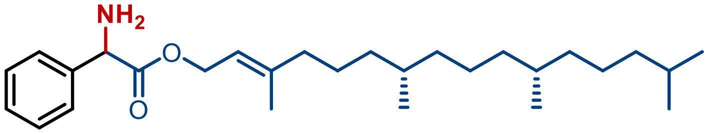

70

(70) (7S,11S,E)-3,7,11,15-tetramethylhexadec-2-en-1-yl (R)-2-amino-2-phenylacetate

Prepared according to **General Procedure A** using (7S,11S,*E*)-3,7,11,15-tetramethylhexadec-2-en-1-yl 2-diazo-2-phenylacetate **S70** (0.3 mmol, 132.2 mg) and $NH_3 \cdot H_2O$ (308 μL, 2.4 mmol) afforded compound **70** (78.6 mg, 61%) as a colourless oil. **^{1}H NMR** (500 MHz, $CDCl_3$) δ 7.39-7.32 (m, 4H), 7.31-7.27 (m, 1H), 5.29-5.24 (m, 1H), 4.66 (dd, *J* = 12.0, 7.0 Hz, 1H), 4.60 (s, 1H), 4.57 (dd, *J* = 12.0, 7.0 Hz, 1H), 1.99-1.91 (m, 2H), 1.85 (s, 2H), 1.62 (s, 3H), 1.57-1.47 (m, 1H), 1.40-1.10 (m, 14H), 1.09-1.00 (m, 4H), 0.88-0.82 (m, 12H) **^{13}C NMR** (126 MHz, $CDCl_3$) δ 174.0, 143.3, 140.4, 128.7, 127.9, 126.8, 117.6, 62.2, 58.8, 39.8, 39.4, 37.4, 37.33, 37.28, 36.6, 32.8, 32.7, 28.0, 25.0, 24.8, 24.5, 22.7, 22.6, 19.74, 19.69, 16.3. **HRMS** (ESI) m/z calcd for $C_{28}H_{47}NNaO_2$ $[M+Na]^+$ 452.3499, Found: 452.3506.

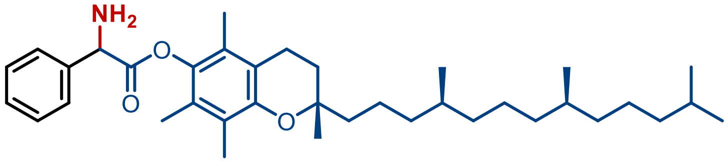

71

(71) Prepared according to **General Procedure A** using diazo compound **S71** (0.3 mmol, 172.5 mg) and $NH_3 \cdot H_2O$ (308 μL, 2.4 mmol) afforded compound **71** (88.0 mg, 52%) as a colourless oil. **^{1}H NMR** (500 MHz, $CDCl_3$) δ 7.55-7.52 (m, 2H), 7.42-7.38 (m, 2H), 7.37-7.32 (m, 1H), 4.93 (s, 1H), 2.60-2.44 (m, 2H), 2.07-1.91 (m, 8H), 1.82-1.72 (m, 2H), 1.59-1.46 (m, 5H), 1.44-1.04 (m, 22H), 0.91-0.82 (m, 12H). **^{13}C NMR** (126 MHz, $CDCl_3$) δ 172.4, 149.4, 140.2, 140.1, 128.7, 128.2, 127.1, 126.7, 124.9, 123.0, 117.3, 75.0, 58.8, 39.3, 37.40, 37.39, 37.3, 32.8, 32.7, 31.1, 27.9, 24.8, 24.4, 22.7, 22.6, 21.0, 20.5, 19.7, 19.6, 11.7. **HRMS** (ESI) m/z calcd for $C_{37}H_{57}NNaO_3$ $[M+Na]^+$ 586.4231, Found: 586.4226.

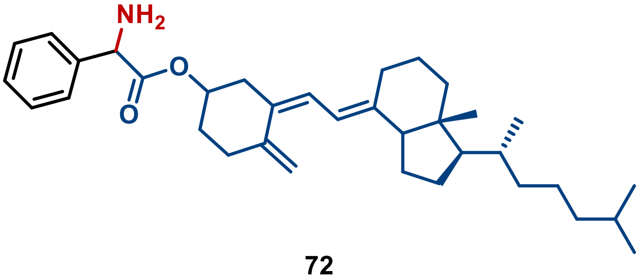

72

(72) Prepared according to **General Procedure A** using diazo compound **S72** (0.3 mmol, 158.6 mg) and $NH_3 \cdot H_2O$ (308 μL, 2.4 mmol) afforded compound **72** (90.1 mg, 58% yield) as a colourless oil. **^{1}H NMR** (500 MHz, $CDCl_3$) δ 7.36 (d, *J* = 7.0 Hz, 2H), 7.30 (t, *J* = 7.0 Hz, 2H), 7.26-7.23 (m, 1H), 5.98 (ABq, *J* = 11.0 Hz, 2H), 5.06-4.95 (m, 2H), 4.79 (d, *J* = 2.0 Hz, 1H), 4.59 (s, 1H), 2.76-2.69 (m, 1H),

2.47-2.33 (m, 2H), 2.23-2.17 (m, 3H), 2.02-1.92 (m, 3H), 1.91-1.83 (m, 1H), 1.81-1.72 (m, 1H), 1.69-1.59 (m, 2H), 1.55-1.44 (m, 4H), 1.37-1.24 (m, 7H), 1.18-1.08 (m, 3H), 1.05-0.97 (m, 1H), 0.92 (d, J = 6.5 Hz, 3H), 0.88-0.85 (m, 6H), 0.52 (s, 3H). **^{13}C NMR** (151 MHz, $CDCl_3$) δ173.3, 144.5, 142.3, 140.1, 133.7, 128.6, 127.8, 126.7, 122.7, 117.5, 112.7, 72.7, 58.7, 56.6, 56.4, 45.9, 41.6, 40.5, 39.5, 36.1, 32.0, 31.8, 29.1, 28.0, 27.7, 23.8, 23.6, 22.8, 22.5, 22.2, 18.8, 11.9. **IR**(Film): 3464, 3003, 2944, 2628, 2253, 1375, 1039, 918 cm^{-1}.

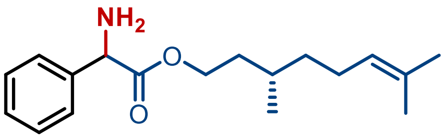

73

(73) Prepared according to **General Procedure A** using diazo compound **S73** (0.3 mmol, 94.3 mg) and $NH_3{\cdot}H_2O$ (308 μL, 2.4 mmol) afforded compound **73** (65.6 mg, 72% yield) as a colourless oil. **^{1}H NMR** (500 MHz, $CDCl_3$) δ 7.40-7.31 (m, 4H), 7.31-7.27 (m, 1H), 5.08-5.00 (m, 1H), 4.59 (s, 1H), 4.18-4.09 (m, 2H), 2.00-1.90 (m, 3H), 1.90-1.79 (m, 1H), 1.67 (s, 3H), 1.64-1.54 (m, 4H), 1.42-1.33 (m, 2H), 1.30-1.21 (m, 1H), 1.15-1.06 (m, 1H), 0.85-0.79 (m, 3H). **^{13}C NMR** (126 MHz, $CDCl_3$) δ 174.0, 140.4, 131.23, 131.21, 128.7, 127.9, 126.7, 124.5, 63.8, 58.8, 36.82, 36.79, 35.2, 29.30, 29.28, 25.6, 25.3, 19.2, 19.1, 17.6. **IR** (Film): 3474, 3001, 2943, 2628, 2253, 1753, 1375, 1038, 918 cm^{-1}.

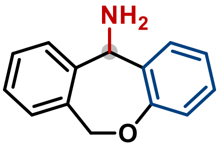

74

(74) 6,11-dihydrodibenzo[*b*,*e*]oxepin-11-amine

Prepared according to **General Procedure A** using 11-diazo-6,11-dihydrodibenzo[*b*,*e*]oxepine **S74** (0.3 mmol, 66.7 mg) and $NH_3{\cdot}H_2O$ (308 μL, 2.4 mmol) afforded compound **74** (36.1 mg, 57% yield) as a colourless oil. **^{1}H NMR** (500 MHz, $CDCl_3$) δ 7.36-7.31 (m, 1H), 7.29-7.23 (m, 3H), 7.21-7.15 (m, 2H), 6.99-6.95 (m, 2H), 5.92 (d, J = 13.5 Hz, 1H), 5.05-5.00 (m, 2H), 2.04 (s, 2H). **^{13}C NMR** (126 MHz, $CDCl_3$) δ 157.1, 141.8, 135.6, 132.3, 129.8, 129.1, 128.3, 128.2, 128.0, 127.8, 122.5, 120.8, 71.9, 60.4. **HRMS** (ESI) m/z calcd for $C_{14}H_{13}NNaO$ $[M+Na]^+$ 234.0889, Found: 234.0890.

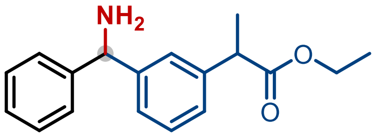

75

(75) ethyl 2-(3-(amino (phenyl) methyl) phenyl) propanoate-methane

Prepared according to **General Procedure C** using ethyl (*Z*)-2-(3-(phenyl(2-((2-(trifluoromethyl) phenyl) sulfonyl) hydrazineylidene) methyl) phenyl) propanoate **S75'** (0.3 mmol, 151.4 mg) and $NH_3 \cdot H_2O$ (308 μL, 2.4 mmol) afforded compound **75** (85.3 mg, 95% yield) as a colourless oil. **^{1}H NMR** (600 MHz, $CDCl_3$) δ 7.36-7.29 (m, 5H), 7.29-7.22 (m, 3H), 7.20 (d, *J* = 7.2 Hz, 1H), 5.31 (s, 1H), 4.16-4.02 (m, 2H), 3.68 (q, *J* = 7.2 Hz, 1H), 3.17 (s, 2H), 1.46 (d, *J* = 7.2 Hz, 3H), 1.16 (td, *J* = 7.2, 1.8 Hz, 3H). **^{13}C NMR** (126 MHz, $CDCl_3$) δ 174.4, 144.23, 144.18, 143.8, 141.02, 140.98, 128.9, 128.6, 127.3, 126.9, 126.40, 126.35, 126.31, 126.2, 125.7, 125.6, 60.7, 59.46, 59.44, 45.50, 45.47, 18.58, 18.50, 14.04. **HRMS** (ESI) m/z calcd for $C_{18}H_{21}NNaO_2$ $[M+Na]^+$ 306.1464, Found: 306.1469.

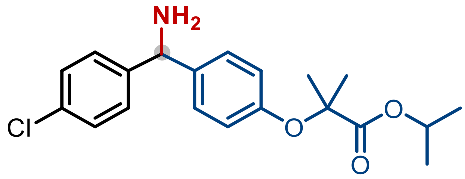

76

(76) isopropyl 2-(4-(amino (4-chlorophenyl) methyl) phenoxy)-2-methylpropanoate-methane

Prepared according to **General Procedure C** using isopropyl (*E*)-2-(4-((4-chlorophenyl) (2-((2-(trifluoromethyl) phenyl) sulfonyl)-2l2-diazaneylidene) methyl) phenoxy)-2-methylpropanoate **S76'** (0.3 mmol, 174.6 mg) and $NH_3 \cdot H_2O$ (308 μL, 2.4 mmol) afforded compound **76** (95.2 mg, 84% yield) as a colourless oil. **^{1}H NMR** (600 MHz, $CDCl_3$) δ 7.30-7.25 (m, 4H), 7.18 (d, *J* = 9.0 Hz, 2H), 6.81-6.76 (m, 2H), 5.12 (s, 1H), 5.10-5.03 (m, 1H), 1.91 (s, 2H), 1.56 (s, 6H), 1.20 (d, *J* = 6.0 Hz, 6H). **^{13}C NMR** (126 MHz, $CDCl_3$) δ 173.6, 154.6, 144.1, 138.5, 132.5, 128.4, 128.2, 127.4, 118.9, 79.0, 68.8, 58.5, 25.3, 21.5.

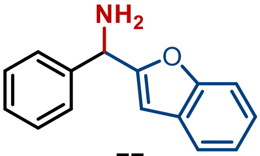

77

(77) benzofuran-2-yl (phenyl) methanamine

Prepared according to **General Procedure C** using *N'*-(benzofuran-2-yl(phenyl) methylene)-2-(trifluoromethyl) benzenesulfonohydrazide **S77'** (0.3 mmol, 94.3 mg) and $NH_3 \cdot H_2O$ (308 μL, 2.4 mmol) afforded compound **77** (65.6 mg, 72% yield) as a colourless oil. **^{1}H NMR** (500 MHz, $CDCl_3$) δ 7.43-7.41 (m, 1H), 7.38-7.35 (m, 2H), 7.35-7.32 (m, 1H), 7.31-7.26 (m, 2H), 7.24-7.21 (m, 1H), 7.18-7.08 (m, 2H), 6.43 (s, 1H), 5.21 (s, 1H), 1.89 (s, 2H). **^{13}C NMR** (126 MHz, $CDCl_3$) δ 160.8, 154.9, 142.1, 128.6, 128.3, 127.7, 127.1, 123.8, 122.6, 120.8, 111.1, 102.6, 54.5.

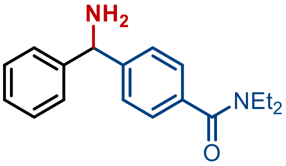

78

(78) 4-(amino (phenyl) methyl)-*N*, *N*-diethylbenzamide

Prepared according to **General Procedure C** using (*Z*)-*N, N*-diethyl-4-(phenyl (2-((2-(trifluoromethyl) phenyl) sulfonyl) hydrazineylidene) methyl) benzamide **S78'** (0.3 mmol, 151.1 mg) and $NH_3{\cdot}H_2O$ (308 μL, 2.4 mmol) afforded compound **78** (51.7 mg, 61% yield) as a colourless oil. **^{1}H NMR** (500 MHz, $CDCl_3$) δ 7.40 (d, *J* = 8.5 Hz, 2H), 7.36 (d, *J* = 7.0 Hz, 2H), 7.34-7.29 (m, 4H), 7.24 (t, *J* = 7.0 Hz, 1H), 5.22 (s, 1H), 3.59-3.47 (m, 2H), 3.30-3.18 (m, 2H), 1.89 (s, 2H), 1.32-1.16 (m, 3H), 1.16-1.03 (m, 3H). **^{13}C NMR** (151 MHz, $CDCl_3$) δ 171.1, 146.5, 145.1, 135.8, 128.5, 127.1, 126.91, 126.87, 126.5, 59.5, 43.2, 39.2, 14.2, 12.8. **HRMS** (ESI) m/z calcd for $C_{18}H_{22}N_2NaO$ $[M+Na]^+$ 305.1624, Found: 305.1616.

---

6. Mechanistic Studies

6.1 Control experiments

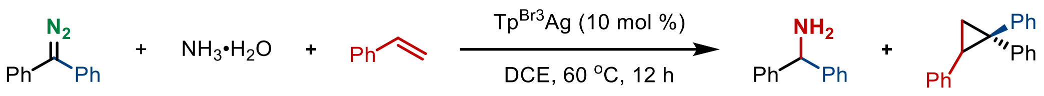

33, 30% **79**, 30%

Experimental Procedures: The Tp^{Br3}Ag(thf) (33.0 mg, 0.03 mmol, 10 mol %) was introduced into a Schlenk Sealing pipe. After sealed the tube was evacuated and backfilled with N_2 for three times. A mixture of $NH_3{\cdot}H_2O$ (308 μL, 28%-30% wt%, 0.6 mmol, 8.0 equiv), Styrene (62.5 mg, 0.6 mmol) and DCE (2 mL) was injected into the Sealing pipe, followed by DCE (2 mL) solution of diphenyldiazomethane (58.3 mg, 0.3 mmol, 1.0 equiv). The resulting mixture was stirred at 60 °C for 12 h in the dark.

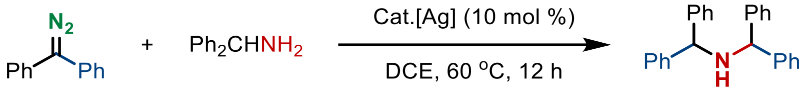

Tp^{Br3}Ag with **$NH_3{\cdot}H_2O$**: **33'**, 0%
Tp^{Br3}Ag without **$NH_3{\cdot}H_2O$**: **33'**, 60%
AgOTf without **$NH_3{\cdot}H_2O$**: **33'**, 98%

Experimental Procedures: The Cat.[Ag] (0.03 mmol, 10 mol %) was introduced into a Schlenk Sealing pipe. After sealed the tube was evacuated and backfilled with N_2 for three times. A mixture of Aminodiphenylmethane (110 mg, 0.6 mmol), $NH_3{\cdot}H_2O$ (308 μL, 28%-30% wt%, 0.6 mmol, 8.0 equiv) and DCE (2 mL) was injected into the Sealing pipe, followed by DCE (2 mL) solution of diphenyldiazomethane (58.3 mg, 0.3 mmol, 1.0 equiv). The resulting mixture was stirred at 60 °C for 12 h in the dark.

Characterization of silver complex [Tp^{Br3}Ag]$_2$(DCE)

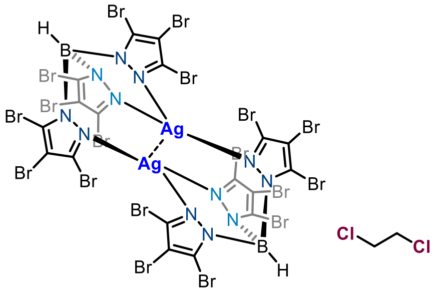

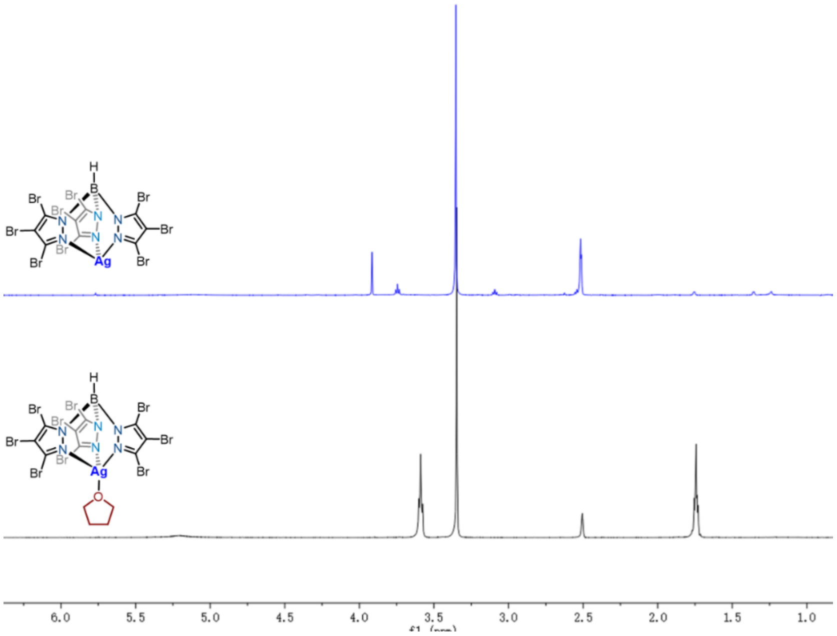

Supplementary Fig. 1 ^{1}H NMR spectrum Comparison of Catalysts Tp^{Br3}Ag(thf) and [Tp^{Br3}Ag]$_2$(DCE)

Supplementary Table 3. X-ray Crystal Structure of [Tp^{Br3}Ag]$_2$(DCE).

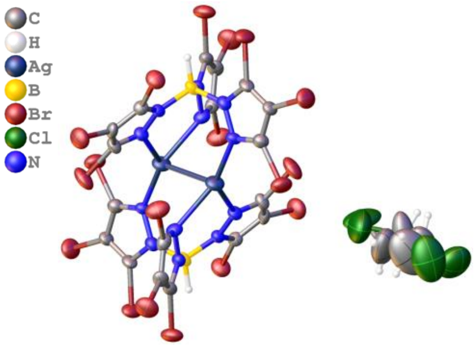

Empirical formula	$C_{11}H_5AgBBr_9Cl_2N_6$
CCDC No.	2166126

Temperature	297 K
Wavelength	0.71073 Å
Unit cell dimensions	a = 10.797 (2) Å<br>b = 11.574 (2) Å<br>c = 11.7947 (19) Å<br>alpha = 82.136 (15) deg.<br>beta = 76.706 (15) deg.<br>gamma = 67.752 (19) deg.
Volume	1325.5 (5) $Å^3$
Z	2
Calculated density	2.831 Mg/m^3
Absorption coefficient	14.541 mm^{-1}
F(000)	1028.0
Crystal size	0.35 x 0.20 x 0.18 mm
Theta range for data collection	2.078 to 24.994
Reflections collected / unique	8643/4634 [R_{int} = 0.0383]
Data / restraints / parameters	4634/20/311
Goodness-of-fit on F^2	1.029
Final R indices [I>2sigma(I)]	R_1 = 0.0442, wR_2 = 0.0768
Rindices (all data)	R_1 = 0.0769, wR_2 = 0.0906

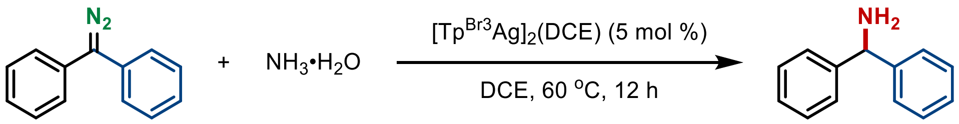

Supplementary Fig. 2 $[Tp^{Br3}Ag]_2$(DCE)-catalyzed N–H insertion of diphenyldiazomethane.

Experimental Procedures: The $[Tp^{Br3}Ag]_2$(DCE) (32.4 mg, 0.03 mmol, 5 mol %) was introduced into a Schlenk Sealing pipe. After sealed the tube was evacuated and backfilled with N_2 for three times. A mixture of $NH_3{\cdot}H_2O$ (308 μL, 28%-30% wt%, 0.6 mmol, 8.0 equiv) and DCE (2 mL) was injected into the Sealing pipe, followed by DCE (2 mL) solution of diphenyldiazomethane (58.3 mg, 0.3 mmol, 1.0 equiv). The resulting mixture was stirred at 60 °C for 12 h in the dark. When the reaction was completed, the crude reaction mixture was allowed to reach room temperature and concentrated in vacuo and purified by column chromatography on silica gel (petroleum ether/EtOAc) to afford the corresponding N–H insertion product Aminodiphenylmethane (44.5 mg, 81%).

6.2 Computational studies

Theoretical methodology: The quantum chemical calculations described in this work were carried out with Gaussian16 package.[15] Geometry optimizations and frequency analysis were performed using the B3LYP functional [16,17] and GD3BJ empirical dispersion.[18] A mixed basis set of SDD [19,20]was used for Ag and Br atoms with 6-31G(d) [21,22] for other atoms. To confirm whether each optimized stationary point is an energy minimum or a saddle point, as well as evaluate the zero-point energy (ZPE) and thermal energy corrections, the vibrational frequencies were computed at the same level of theory. Intrinsic reaction coordinate (IRC) [23,24] calculations were carried out to ascertain the true nature of the transition states. Single-point solvation energies were calculated at the M06[25]/6-311+G(d,p) level (SDD for Ag and Br) with the SMD (solvation-model density) implicit continuum solvent model (DCE solvent)[26] on B3LYP-D3(BJ)-optimized geometries, because it is expected that this strategy will provide greater accuracy for Ag-containing reactions and could give reasonable agreement with higher-level calculations.[27] Computed structures were illustrated using CYLview visualization software.[28]

Silver catalyzed N–H insertion of NH_3

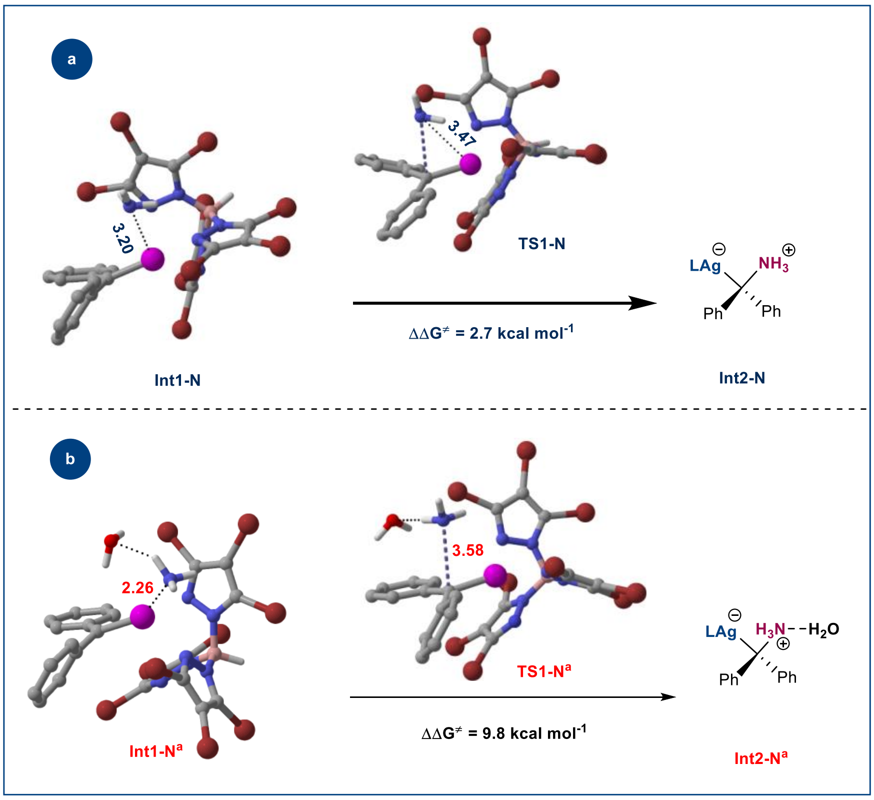

Supplementary Fig. 3 The comparison of the formation of N-ylides from NH_3 and $NH_3{\cdot}H_2O$. a. The formation of *N*-ylide Int2-N from NH_3. **b**. The formation of *N*-ylide Int2-N^a from $NH_3{\cdot}H_2O$.

For comparison, we located the transition state (**TS1-N^a**) for the formation of N-ylide **Int2-N^a** from $NH_3{\cdot}H_2O$ (Fig. S3). The activation barrier of **TS1-N^a** is 7.1 kcal/mol higher than that of

TS1-N from NH_3, thus ruling out the possibility of N−H insertion involving the real forms of $NH_3{\cdot}H_2O$ in solution. As shown in Fig. S3, there is a stronger coordination of N to Ag in **Int1-N**[a] (2.26 versus 3.20 Å). Therefore, the formation of N-ylide **Int2-N**[a] needs to overcome a higher activation barrier (9.8 kcal/mol) to break Ag-N bond in **Int1-N**[a]**.**

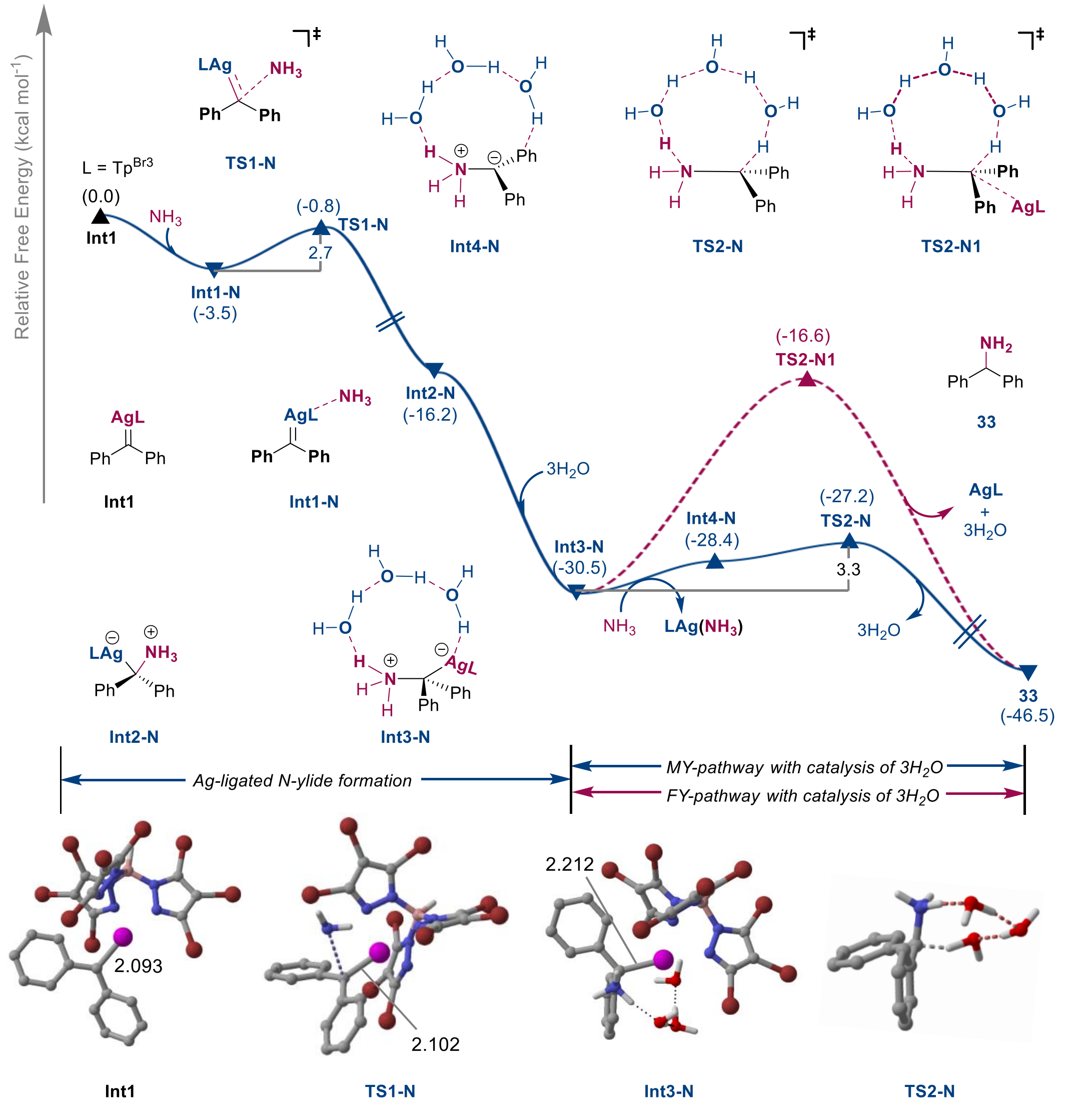

Supplementary Fig. 4 Free energy profiles for the N–H insertion of NH_3 in aqueous ammonia system. The bond distances are given in Å and the relative free energies are given in kcal mol^{-1} calculated at the SMD(DCE)-M06/[6-311+G(d,p)-SDD(Ag/Br)] level.

For N–H insertion of NH_3, three molecules of water assisted free-ylide pathway is the most favorable. As depicted in Fig. S2, the 1,2-proton transfer from free-ylide (FY) **Int4-N** (via **TS2-N**, red line) is more favored than that from the silver-ligated ylide (MY) **Int3-N** (via **TS2-N1**, blue line). The RDS activation free energies for these two paths are 3.3 kcal mol^{-1} and 13.9 kcal mol^{-1} respectively, indicating a water-assisted free-ylide intermediate mechanism.

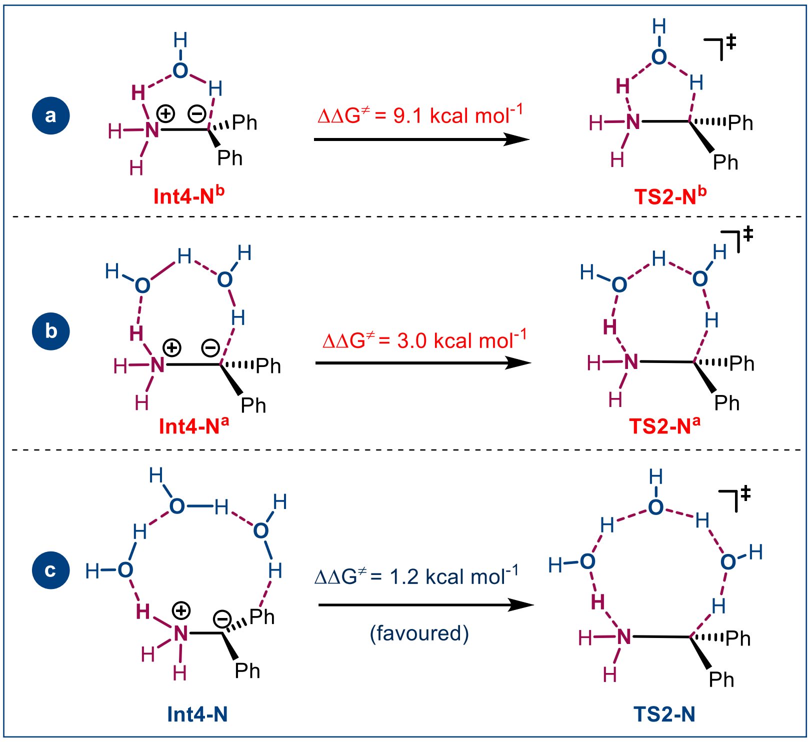

Supplementary Fig. 5 The comparison of one, two and three H_2O molecules-assisted 1,2-proton transfer of *N*-ylide. a. One H_2O molecule-assisted 1,2-proton transfer of *N*-ylide. **b.** Two H_2O molecule-assisted 1,2-proton transfer of *N*-ylide. **c.** Three H_2O molecule-assisted 1,2-proton transfer of *N*-ylide.

As shown in Supplementary Fig. 5, the energy barriers for one, two and three H_2O molecules-assisted 1,2-proton transfer For N−H insertion are 9.1, 3.0 and 1.2 kcal/mol, respectively.

Silver catalyzed N–H insertion of primary amine 33:

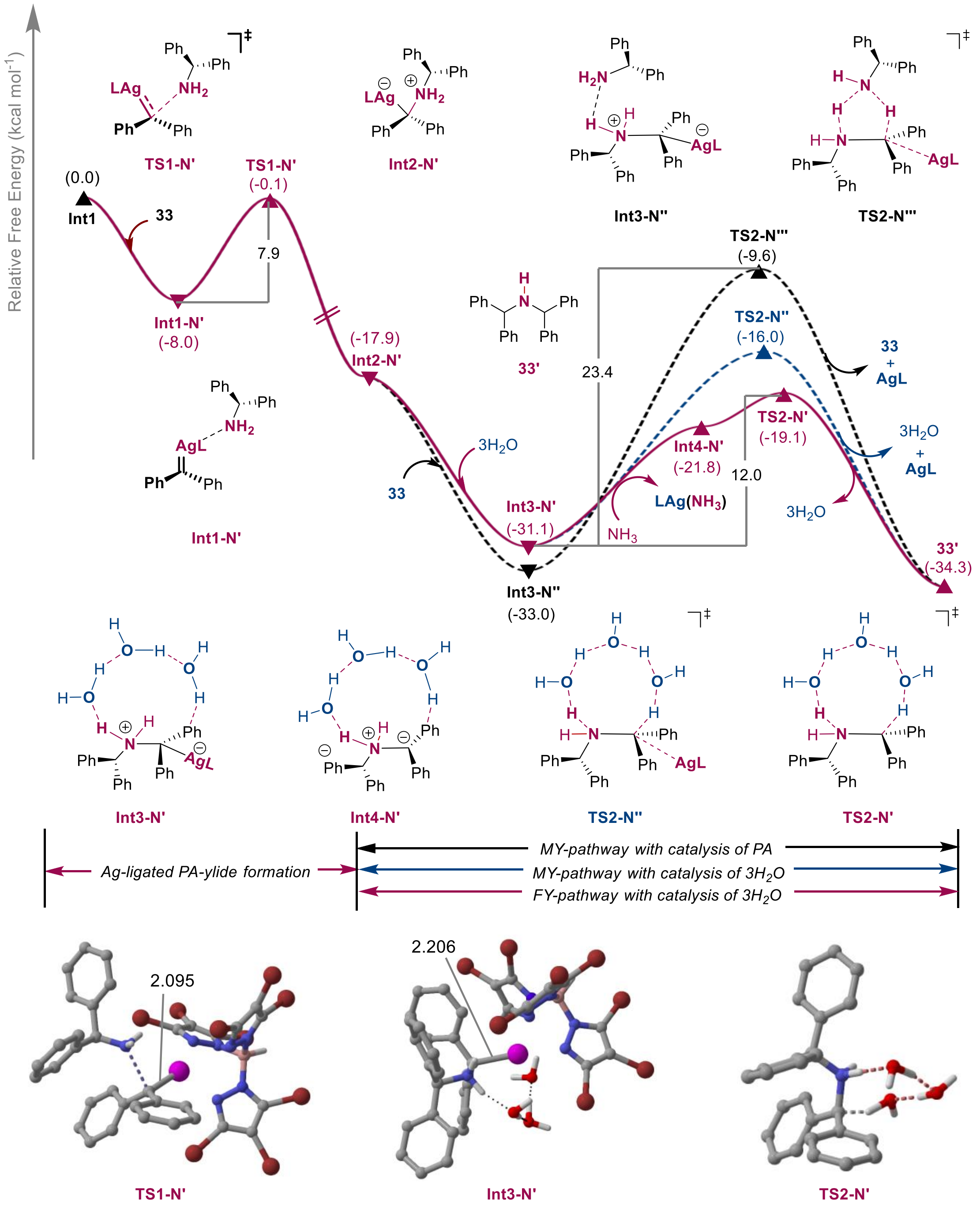

Supplementary Fig. 6 Free energy profiles for the N–H insertion of primary amine **33** in aqueous ammonia system (red and blue) and in DCE (black line). The bond distances are given in Å and the relative free energies are given in kcal mol^{-1} calculated at the SMD(DCE)-M06/[6-311+G(d,p)-SDD(Ag/Br)] level.

The N–H insertion of primary amine **33** in aqueous ammonia system possesses the same mechanism with N–H insertion of NH_3. The RDS activation free energies for three water-assisted 1,2-proton transfer process from free-ylide **Int4- N'** (via **TS2-N'**, red line) and from silver-ligated ylide **Int3-N'** (via **TS2-N''**, blue line) are 12.0 and 15.1 kcal mol^{-1}, respectively, which are much higher than that of N–H insertion of NH_3 (3.3 kcal mol^{-1} for RDS activation free energy). These results are in agreement with the experimental observations that no over N–H insertion product

33' was observed. Interestingly, the reaction of primary amine **33** with diphenyldiazomethane in the absence of aqueous ammonia gave N–H insertion product **33'** in 60% yield. We suspected that the 1,2-proton transfer process may be assisted by primary amine **33**. In fact, the activation free energy is 23.4 kcal mol^{-1} (**TS2-N'''**, black line), indicating this process can occur smoothly under the current reaction conditions (60 °C).

Silver catalyzed O–H insertion of H_2O

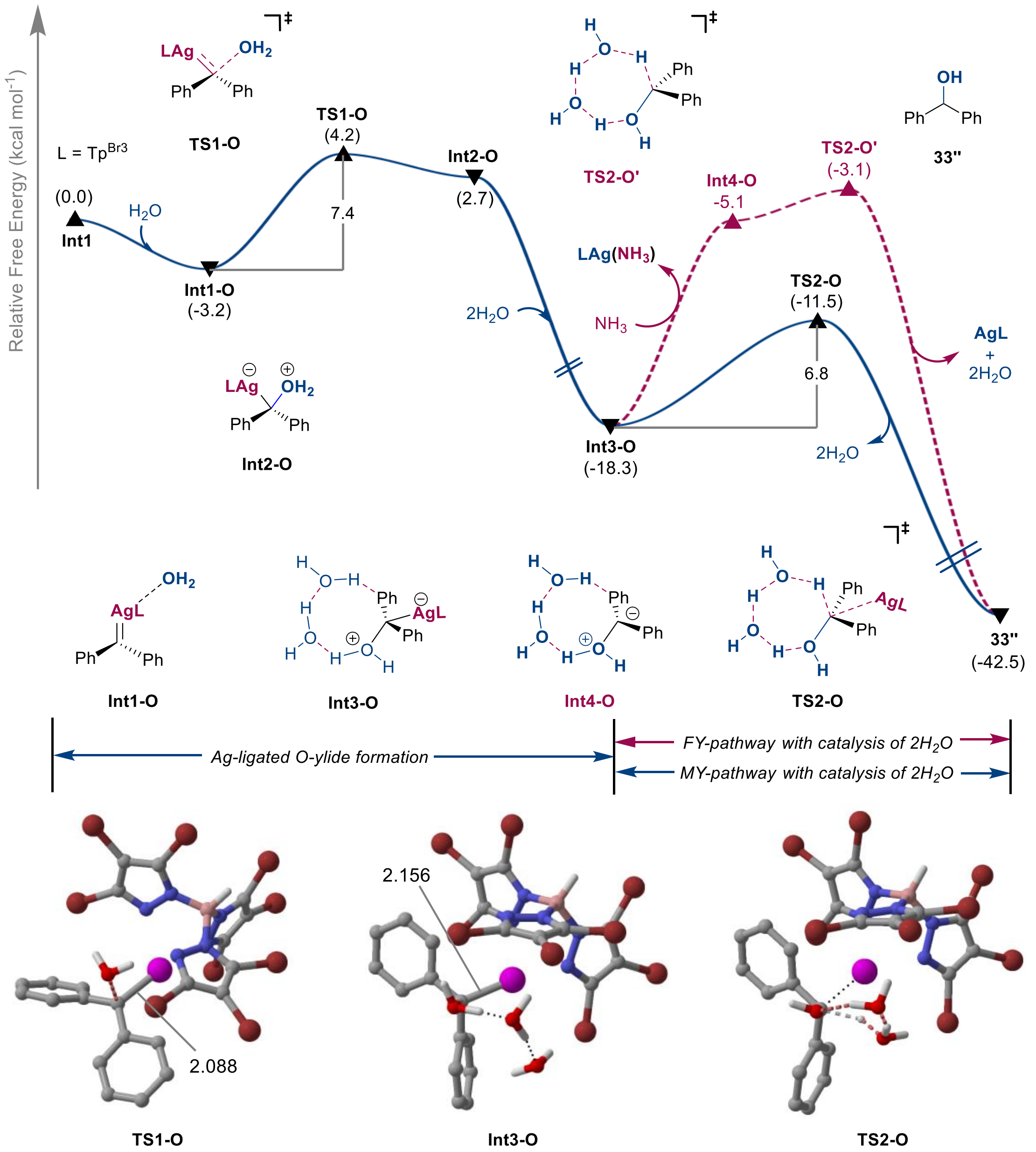

Supplementary Fig. 7 Free energy profiles for the O–H insertion of H_2O in aqueous ammonia system. The bond distances are given in Å and the relative free energies are given in kcal mol^{-1} calculated at the SMD(DCE)-M06/[6-311+G(d,p)-SDD(Ag/Br)] level.

Distinct from the N–H insertion, two H_2O molecules assisted 1,2-proton shift from silver-ligated ylide **Int3-O** (6.8 kcal mol^{-1}, via **TS2-O**, blue line) is more favored than that from the free-ylide **Int4-O** (15.2 kcal mol^{-1} via **TS2-O'**, red line), which may be ascribed to the considerable C-Ag bond strength in **Int3-O**.

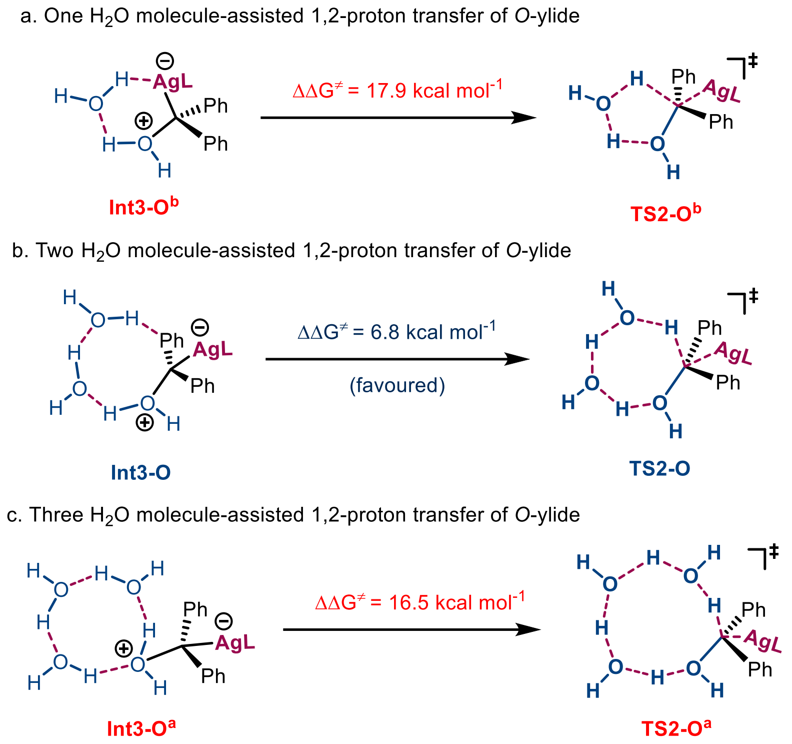

Supplementary Fig. 8 The comparison of one, two and three H_2O molecules-assisted 1,2-proton transfer of *O*-ylide

The origin for divergent silver-ligated ylide formation

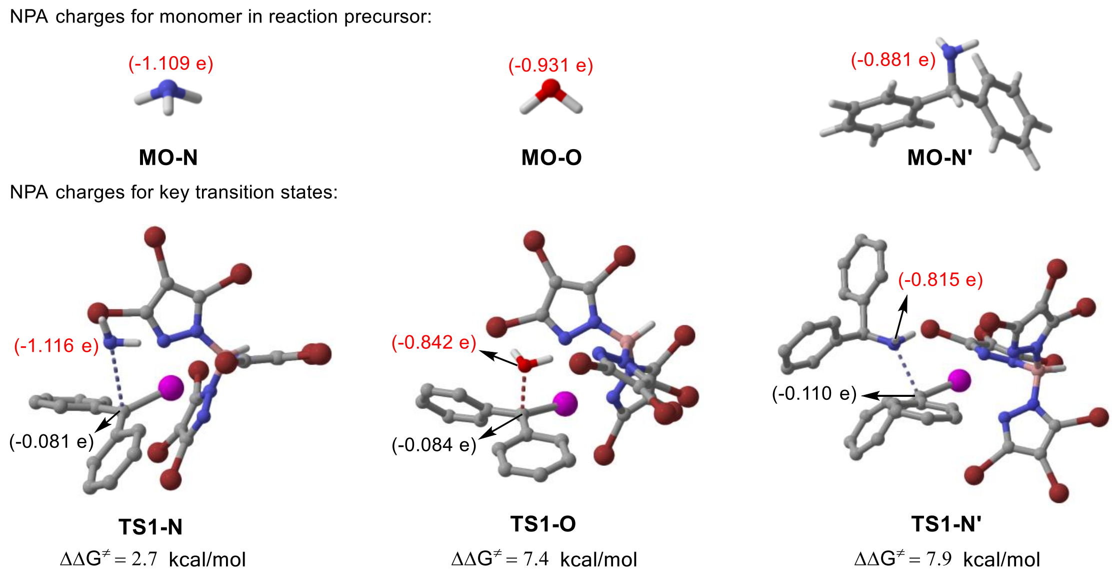

Supplementary Fig. 9 NPA charge analysis for the origin to form the different silver-ligated ylide species: The values in e represent the NPA charge of certain atoms.

To probe the origin of divergent ylide species formation, we have carried out NPA charge analysis for corresponding monomers as well as key transition states. Results show that the charge on N in **MO-N** is -1.109 e whereas the charges on O in **MO-O** and N in **MO-N'** are -0.931 e and -0.881 e respectively, confirming the order of nucleophilicity follows the trend **MO-N > MO-O > MO-N'**. Similarly, the charges in key transition states can also lead to the order of nucleophilicity as **TS1-N > TS1-O > TS1-N'**. These results are consistent with the ylide formation activation free energies of 2.7, 7.4 and 7.9 kcal mol^{-1}.

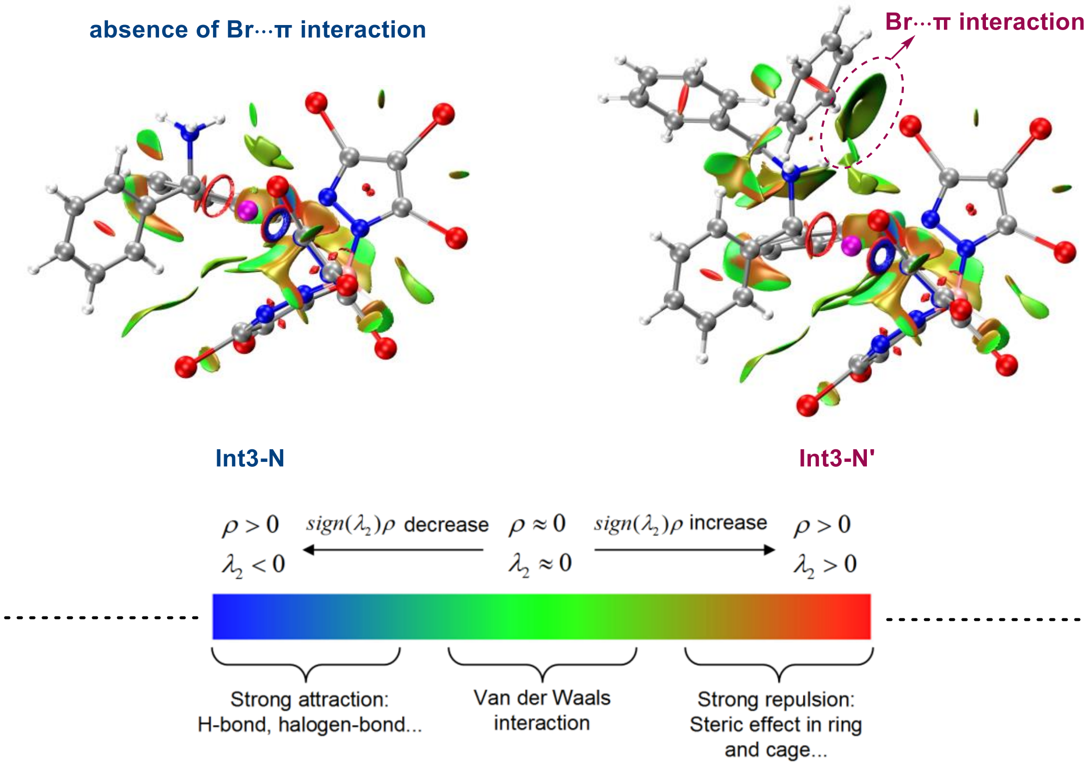

Supplementary Fig. 10 NCI analysis for the key intermediates **Int3-N** and **Int3-N'** in N–H insertion of NH_3 and primary amine **33**.

The reaction pathway for the N–H insertion of diphenylmethylamine **33** encounters a higher energy barrier to the cleavage of the Ag–C bond in ylide **Int3-N'** (9.3 vs 2.1 kcal mol^{-1}). To explore the origin of this phenomenon, we have performed NCI analysis of **Int3-N** and **Int3-N'** using Multiwfn.[29] There is a stronger Br···π weak interaction between phenyl and bulky Tp^{Br3} ligand in **Int3-N'**, which renders the silver catalyst part and ylide part binds more tightly and a higher dissociation energy is needed for **Int3-N'** to break the Ag–C bond in ylide **Int3-N'**. This Br···π weak interaction is absent from **Int3-N**, which results in the highest RDS activation free energy (3.3 kcal mol^{-1}) for N–H insertion of NH_3 is much lower than that of N–H insertion of diphenylmethylamine **33** (12.0 kcal mol^{-1}).

7. NMR Spectra of Product

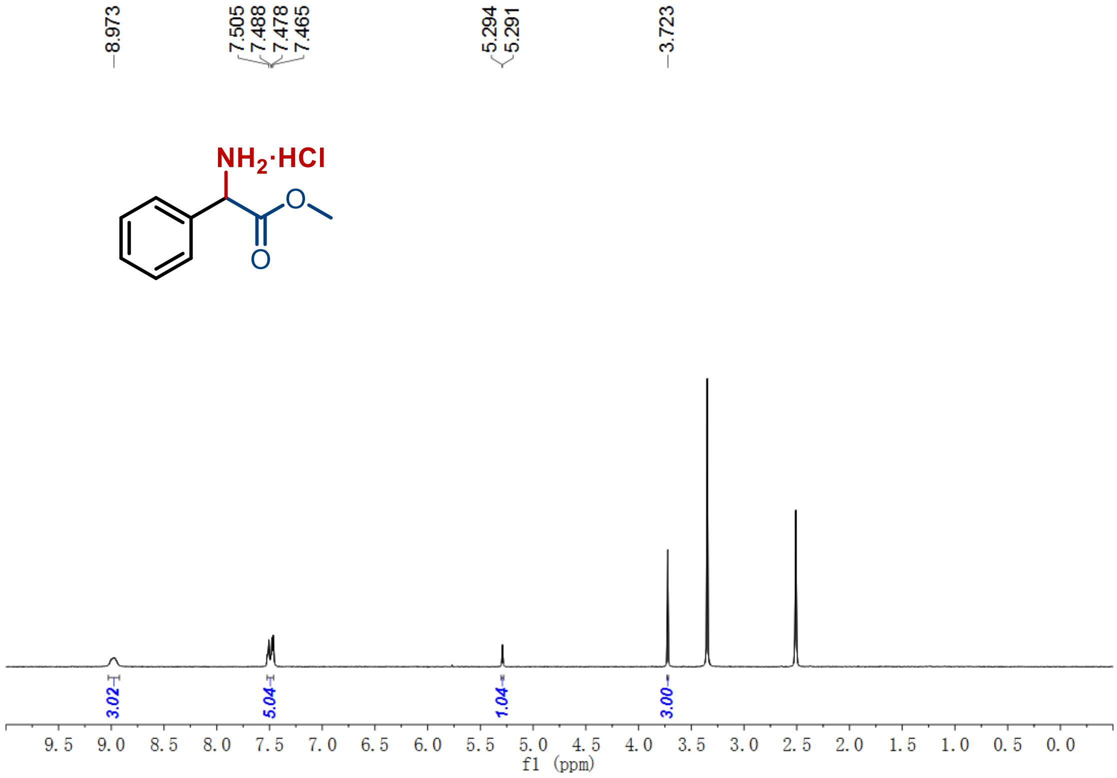

Supplementary Fig. 11 ^{1}H NMR (500 MHz, DMSO) spectrum of compound **2**

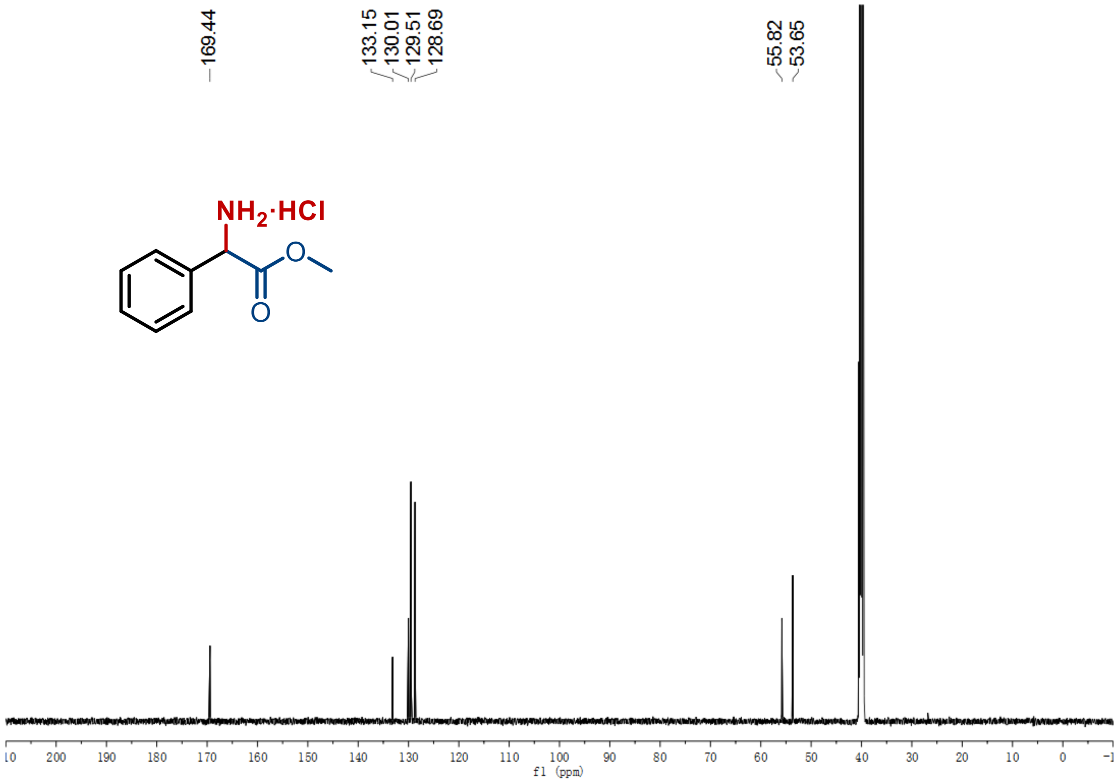

Supplementary Fig. 12 ^{13}C NMR (126 MHz, DMSO) spectrum of compound **2**

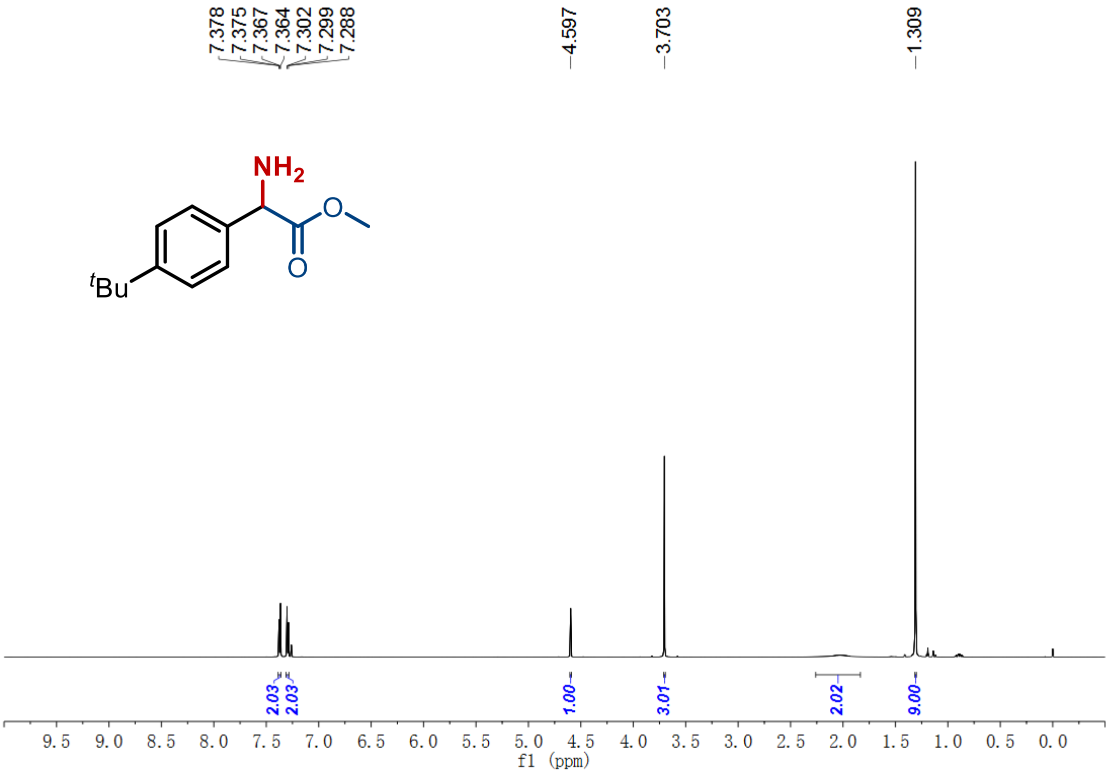

Supplementary Fig. 13 ^{1}H NMR (600 MHz, $CDCl_3$) spectrum of compound **4**.

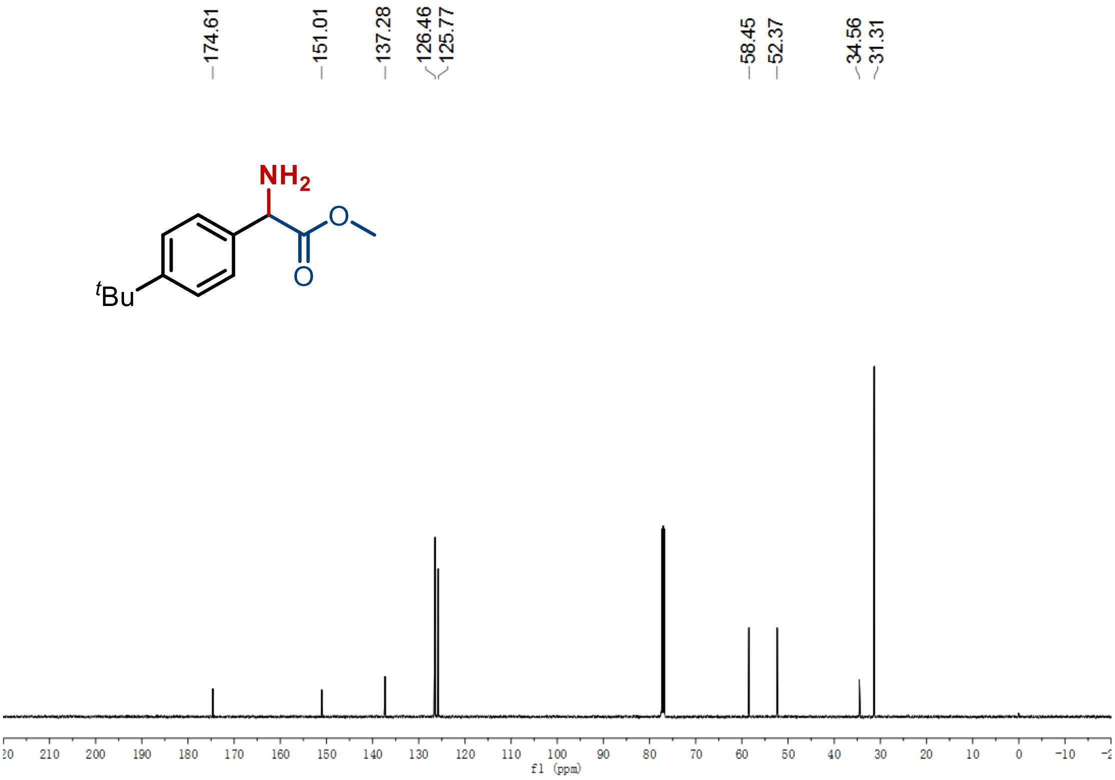

Supplementary Fig. 14 ^{13}C NMR (126 MHz, $CDCl_3$) spectrum of compound **4**.

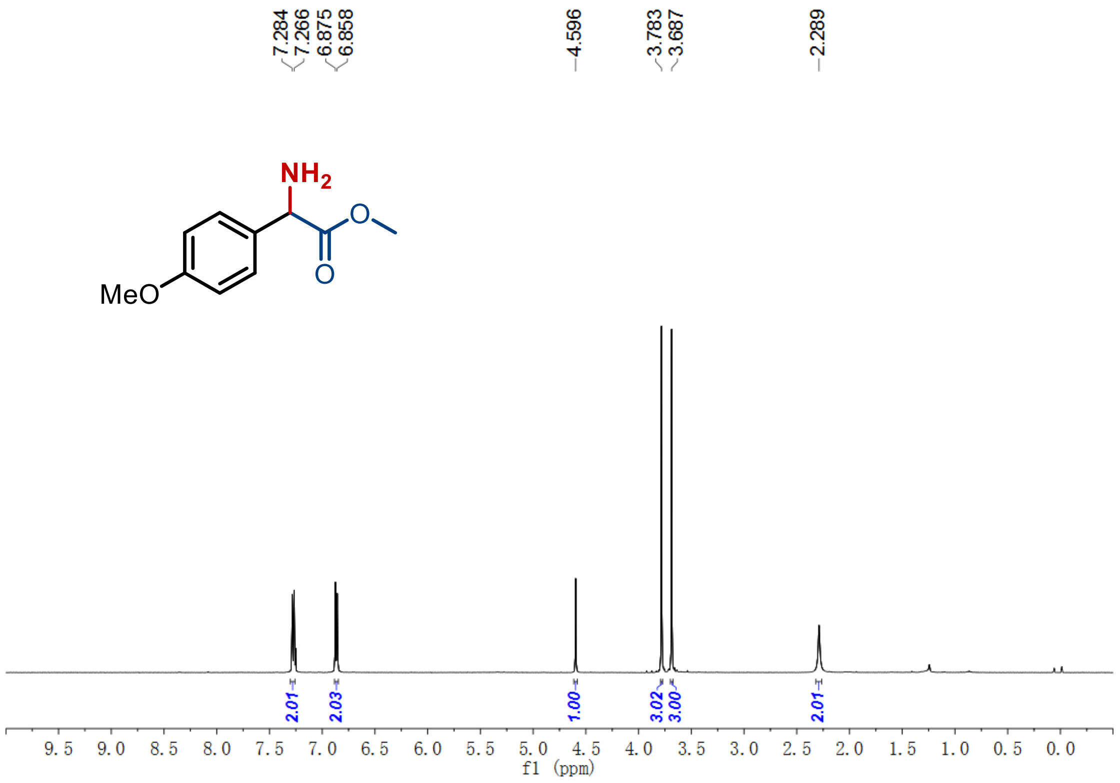

Supplementary Fig. 15 ^{1}H NMR (500 MHz, $CDCl_3$) spectrum of compound **5**.

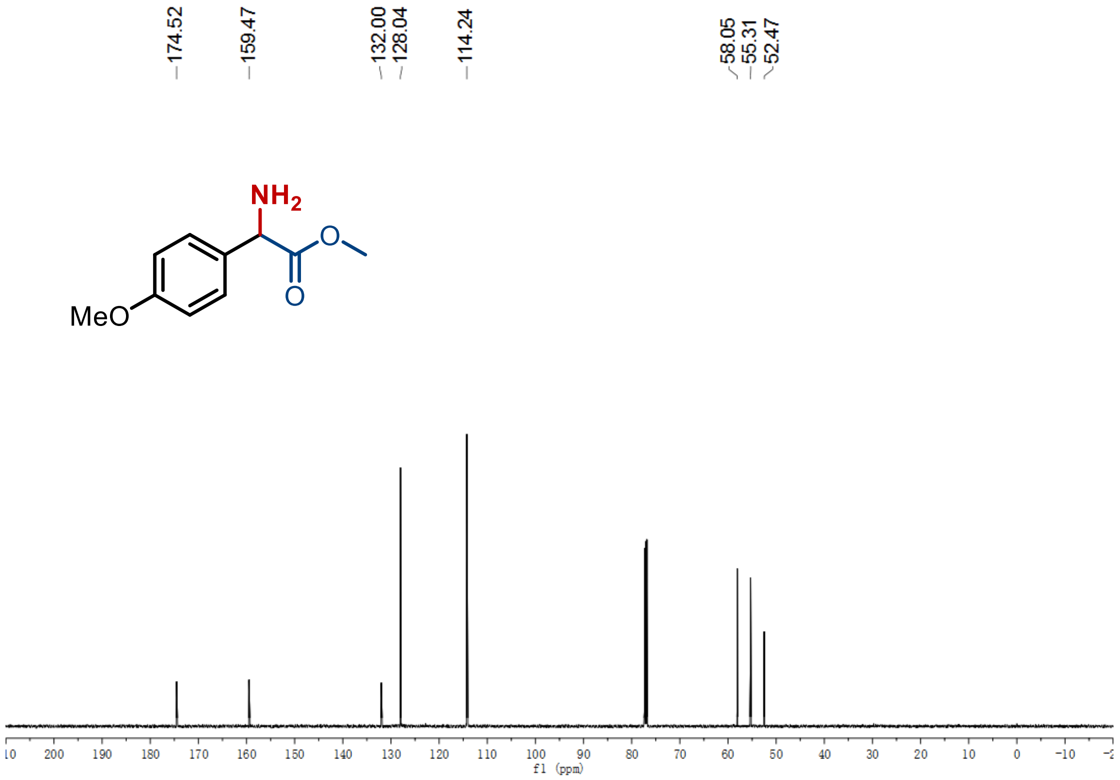

Supplementary Fig. 16 ^{13}C NMR (151 MHz, $CDCl_3$) spectrum of compound **5**.

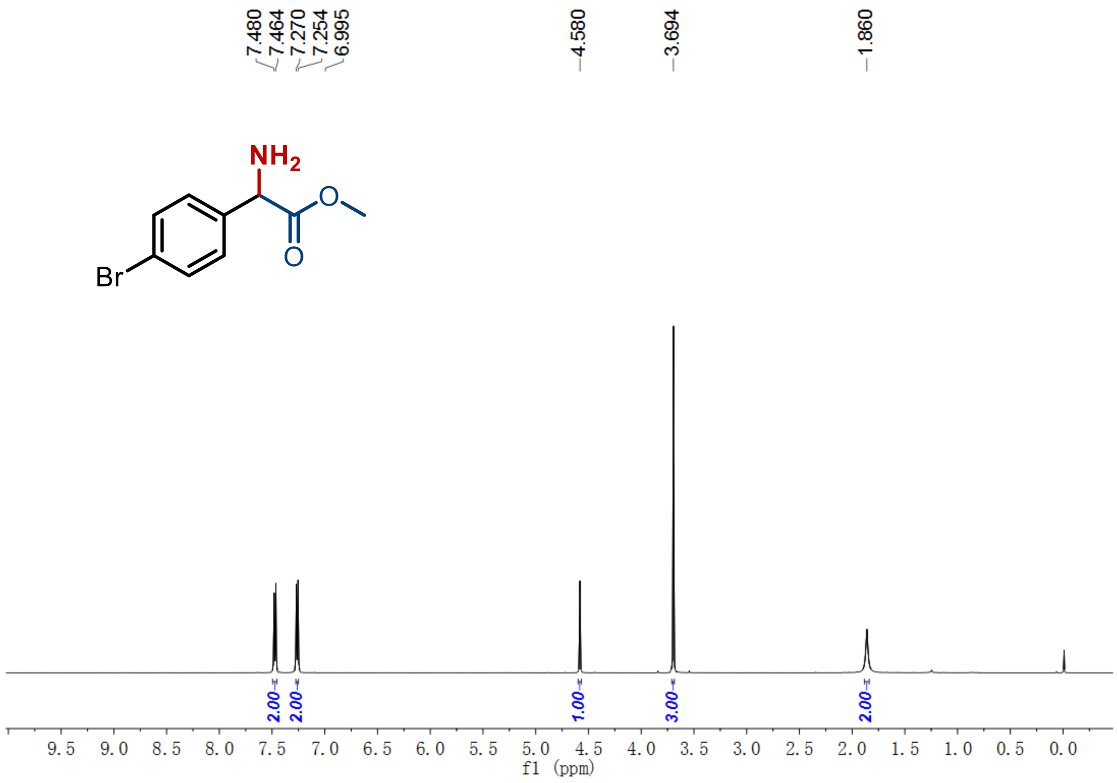

Supplementary Fig. 17 ^{1}H NMR (500 MHz, $CDCl_3$) spectrum of compound **6**.

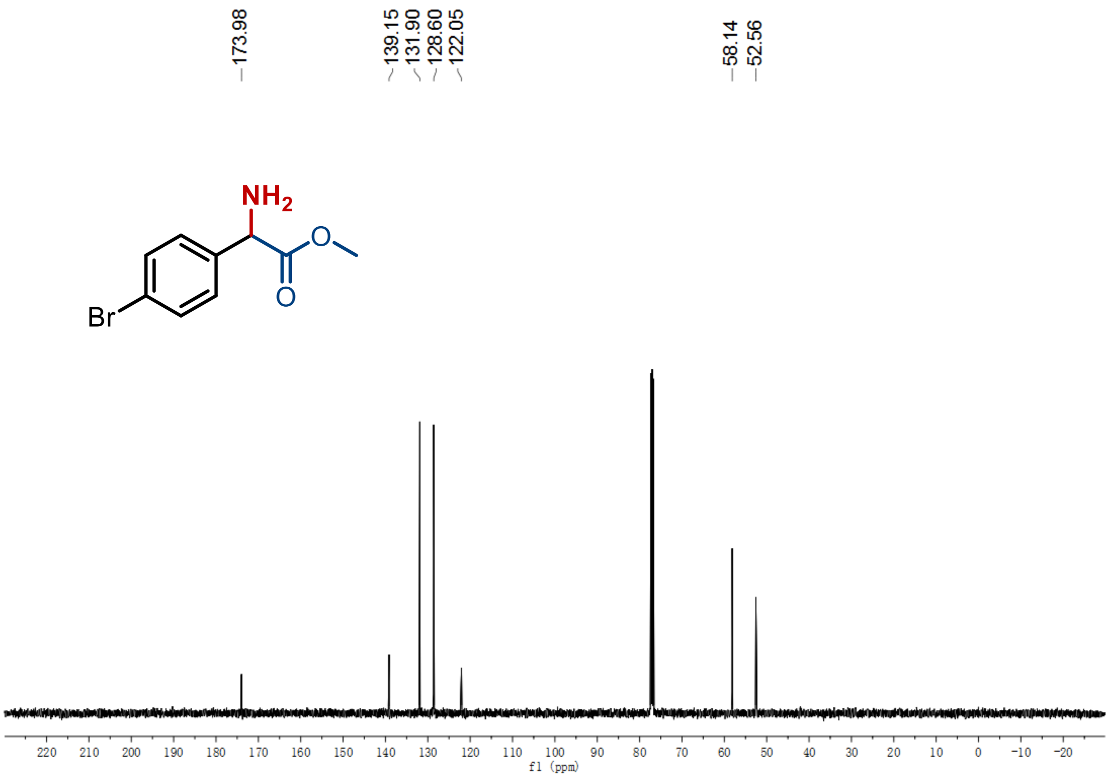

Supplementary Fig. 18 ^{13}C NMR (126 MHz, $CDCl_3$) spectrum of compound **6**.

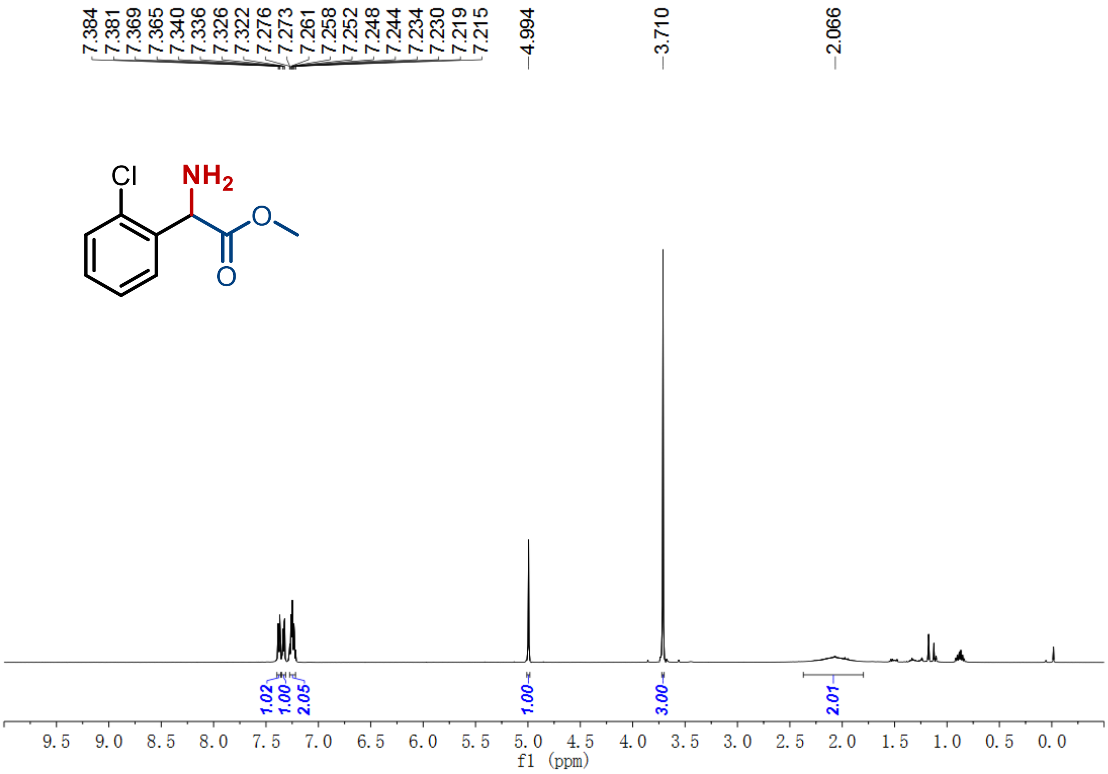

Supplementary Fig. 19 ^{1}H NMR (500 MHz, $CDCl_3$) spectrum of compound **7**.

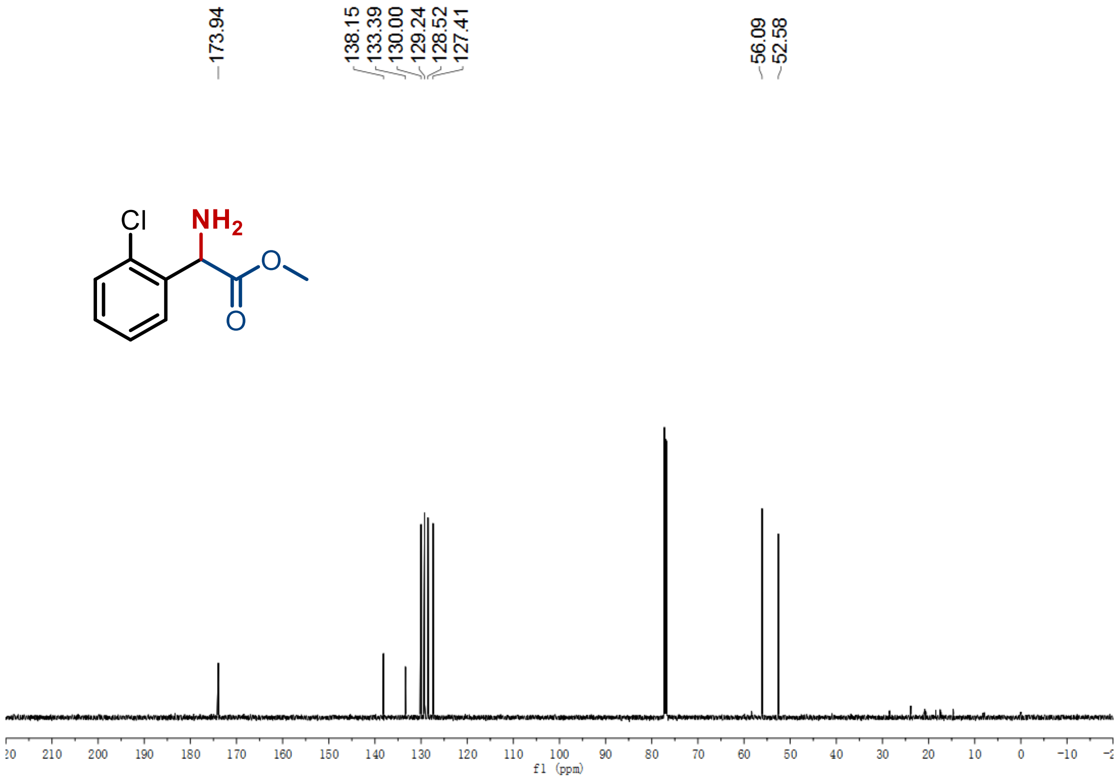

Supplementary Fig. 20 ^{13}C NMR (151 MHz, $CDCl_3$) spectrum of compound **7**.

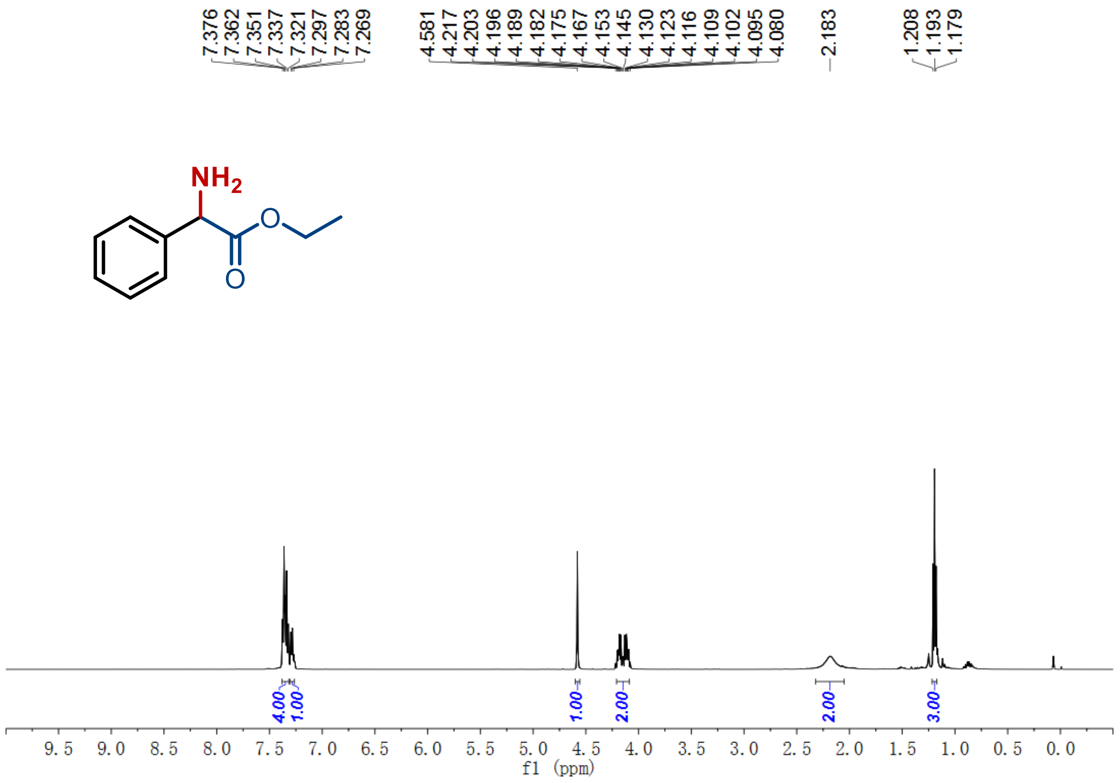

Supplementary Fig. 21 ^{1}H NMR (500 MHz, $CDCl_3$) spectrum of compound **8**.

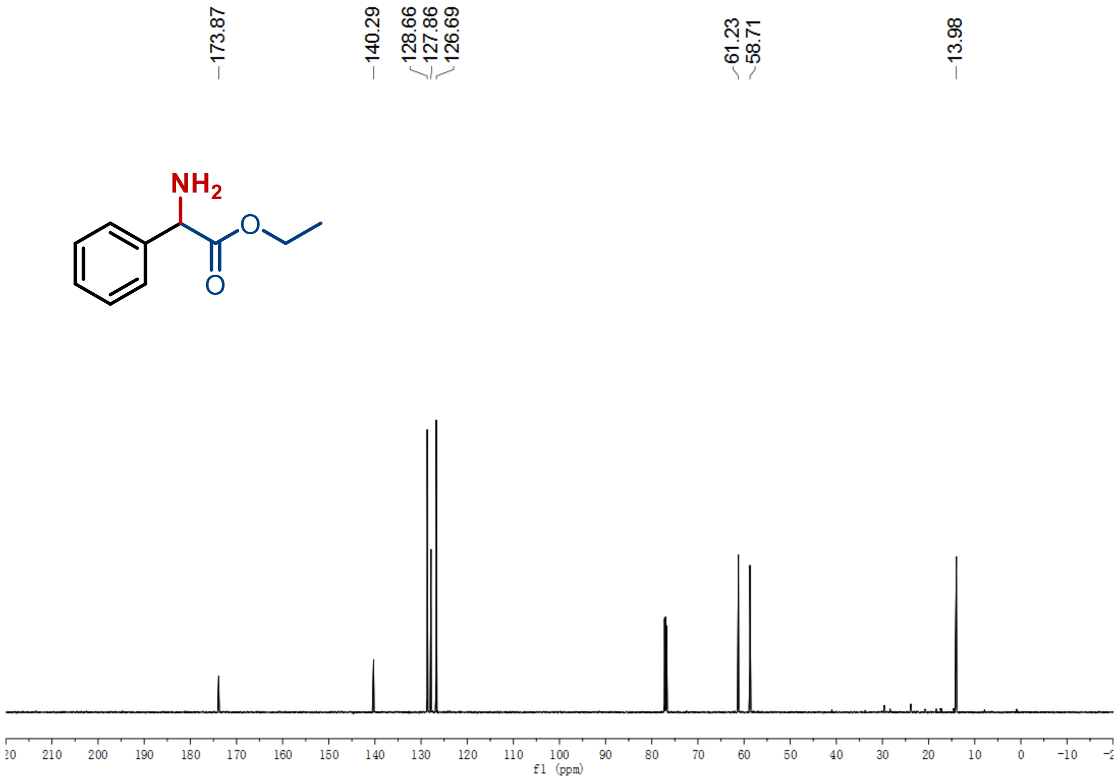

Supplementary Fig. 22 ^{13}C NMR (126 MHz, $CDCl_3$) spectrum of compound **8**.

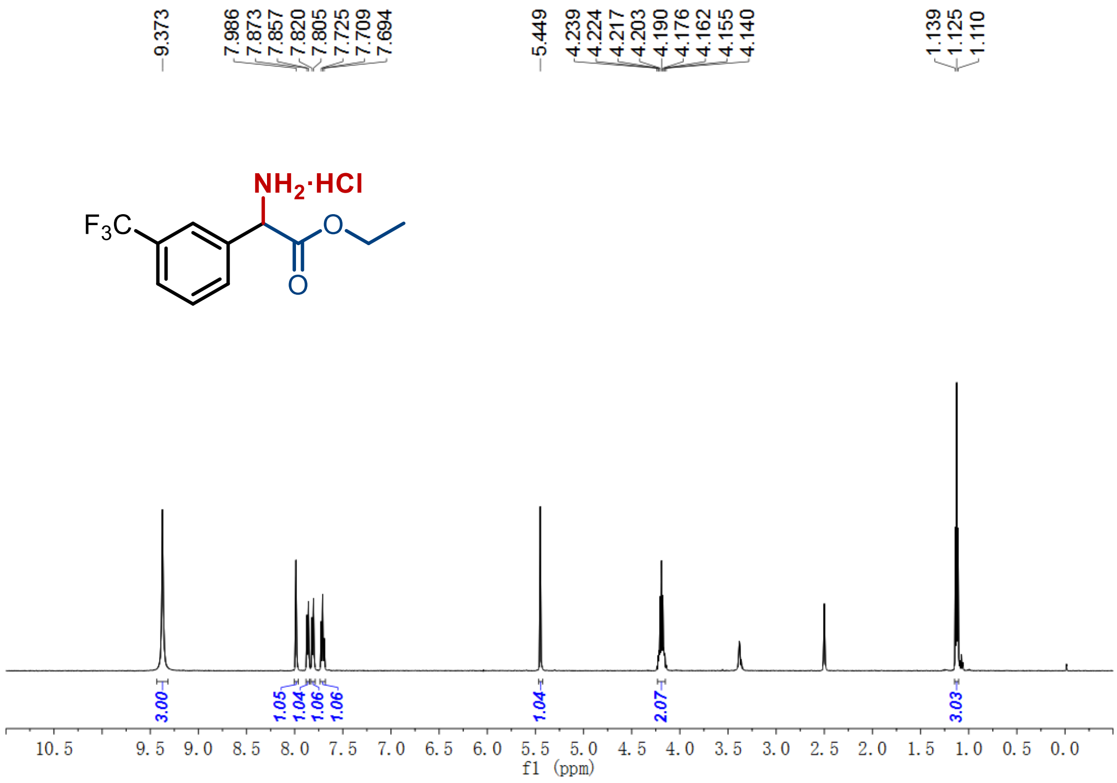

Supplementary Fig. 23 ^{1}H NMR (500 MHz, DMSO) spectrum of compound **9**.

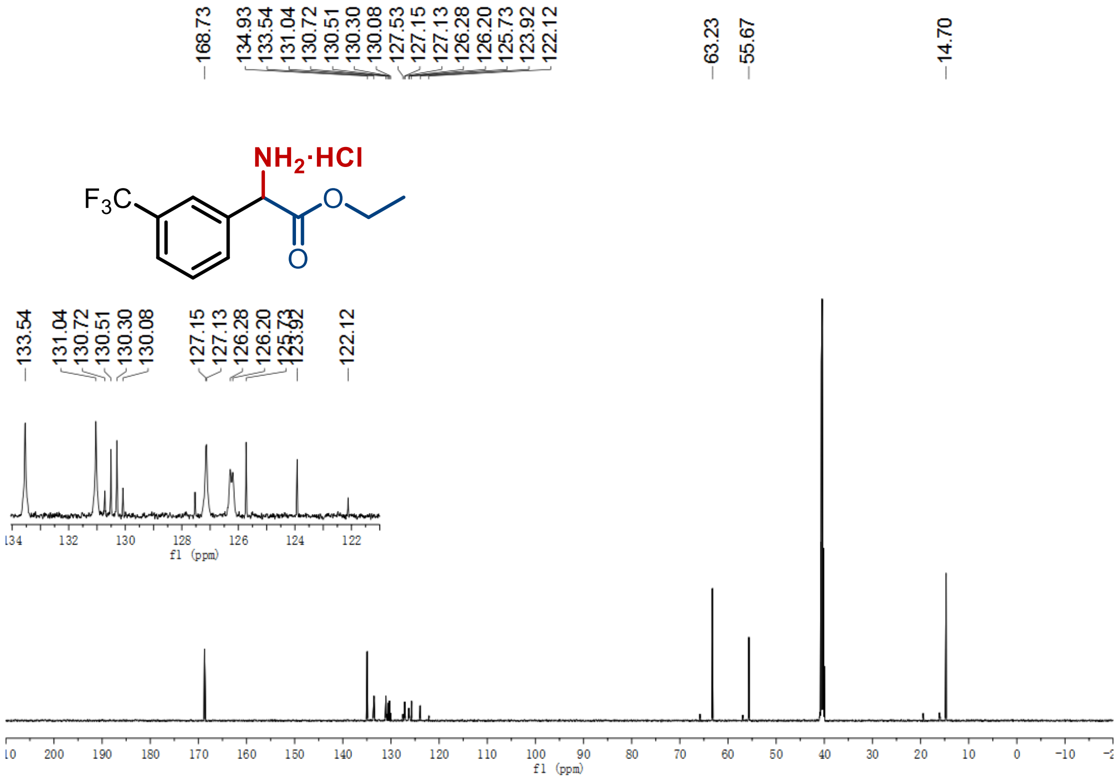

Supplementary Fig. 24 ^{13}C NMR (151 MHz, DMSO) spectrum of compound **9**.

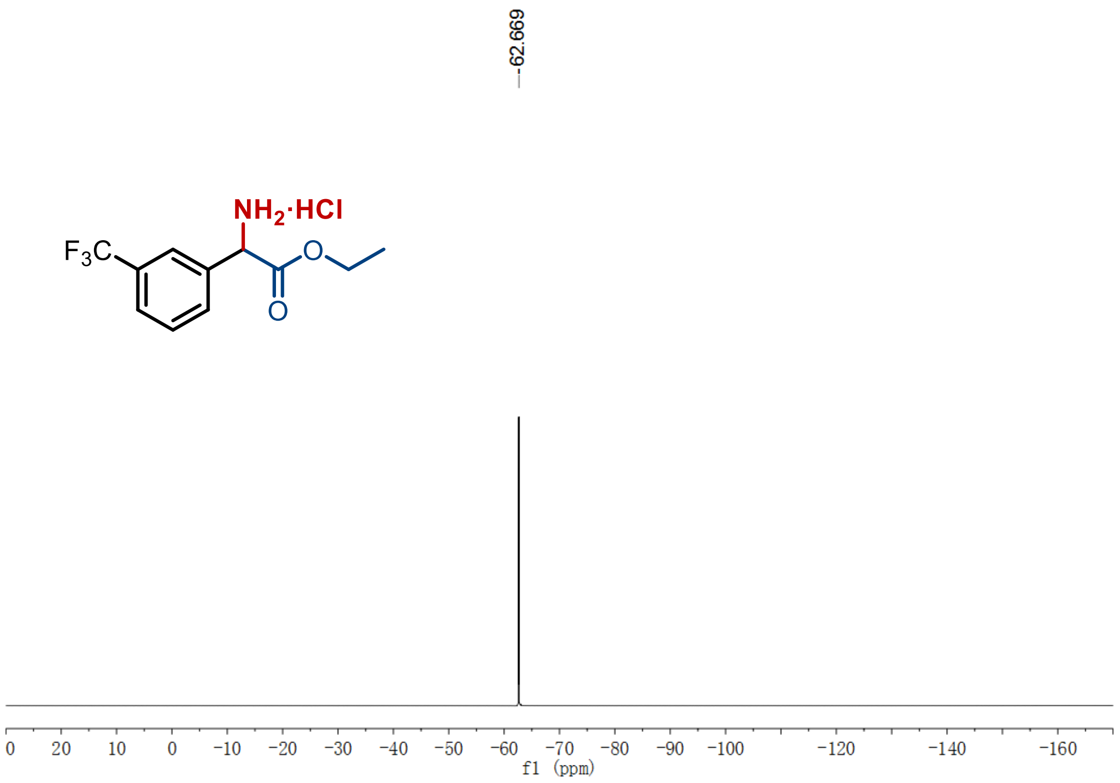

Supplementary Fig. 25 ^{19}F NMR (565 MHz, DMSO) spectrum of compound **9**.

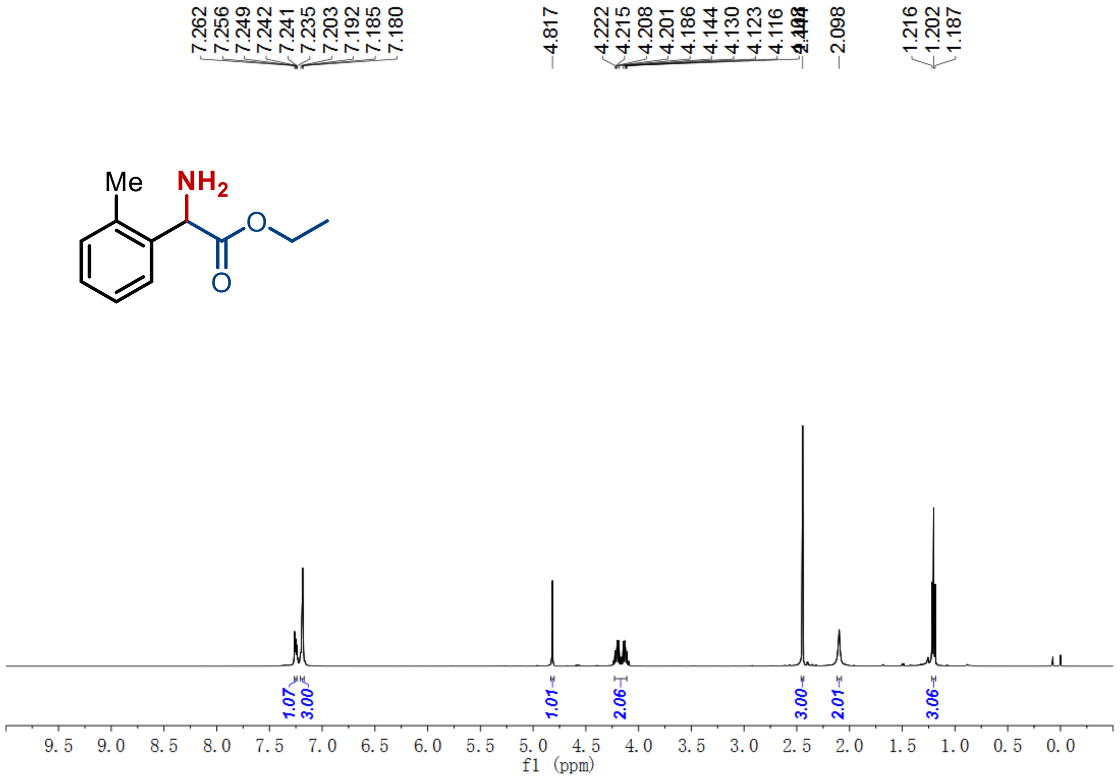

Supplementary Fig. 26 ^{1}H NMR (500 MHz, $CDCl_3$) spectrum of compound **10**.

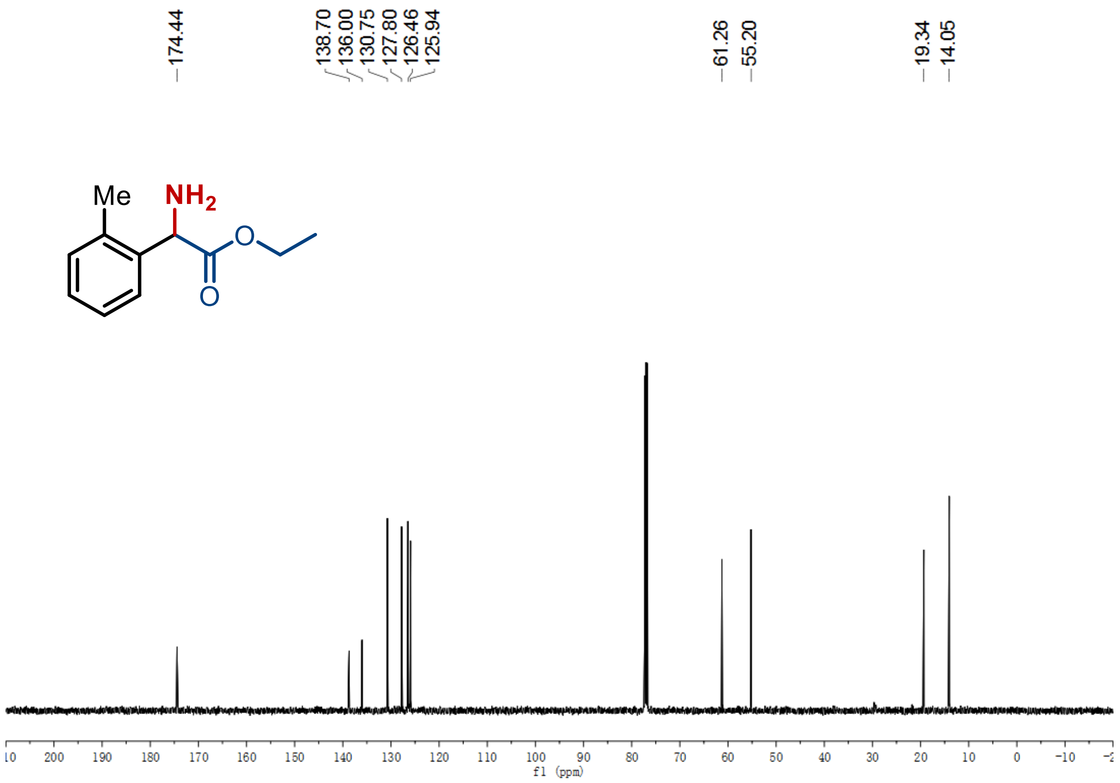

Supplementary Fig. 27 ^{13}C NMR (126 MHz, $CDCl_3$) spectrum of compound **10**.

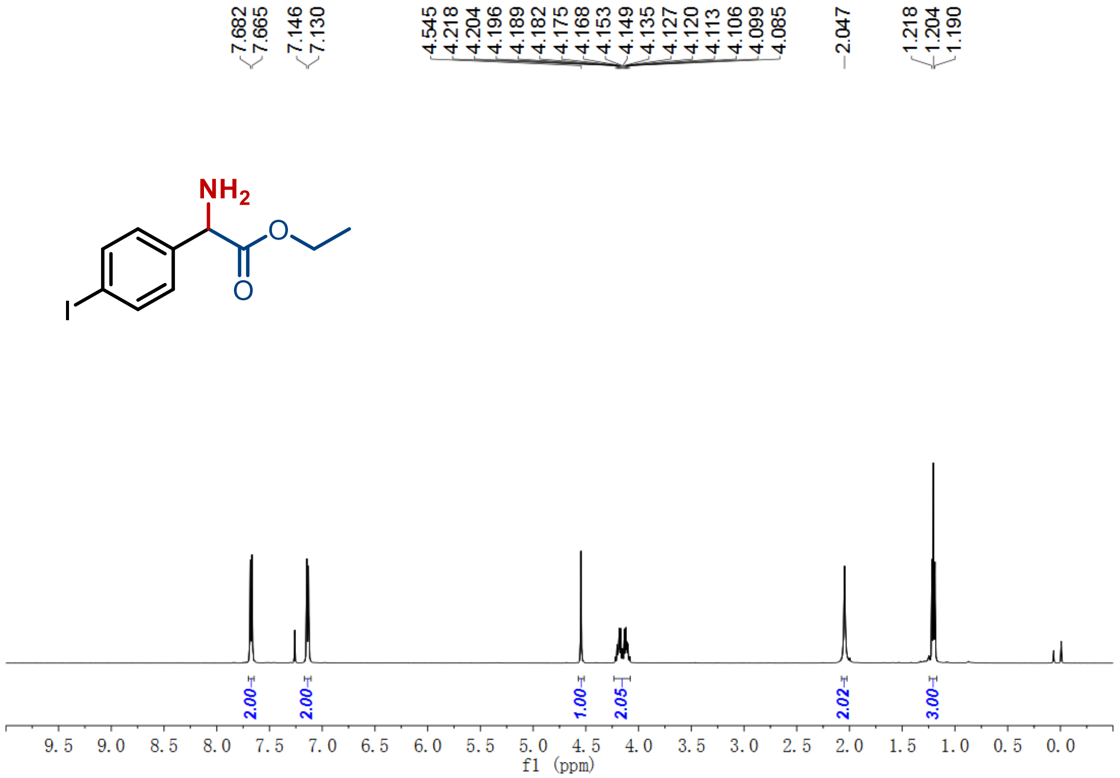

Supplementary Fig. 28 ^{1}H NMR (500 MHz, $CDCl_3$) spectrum of compound **11**.

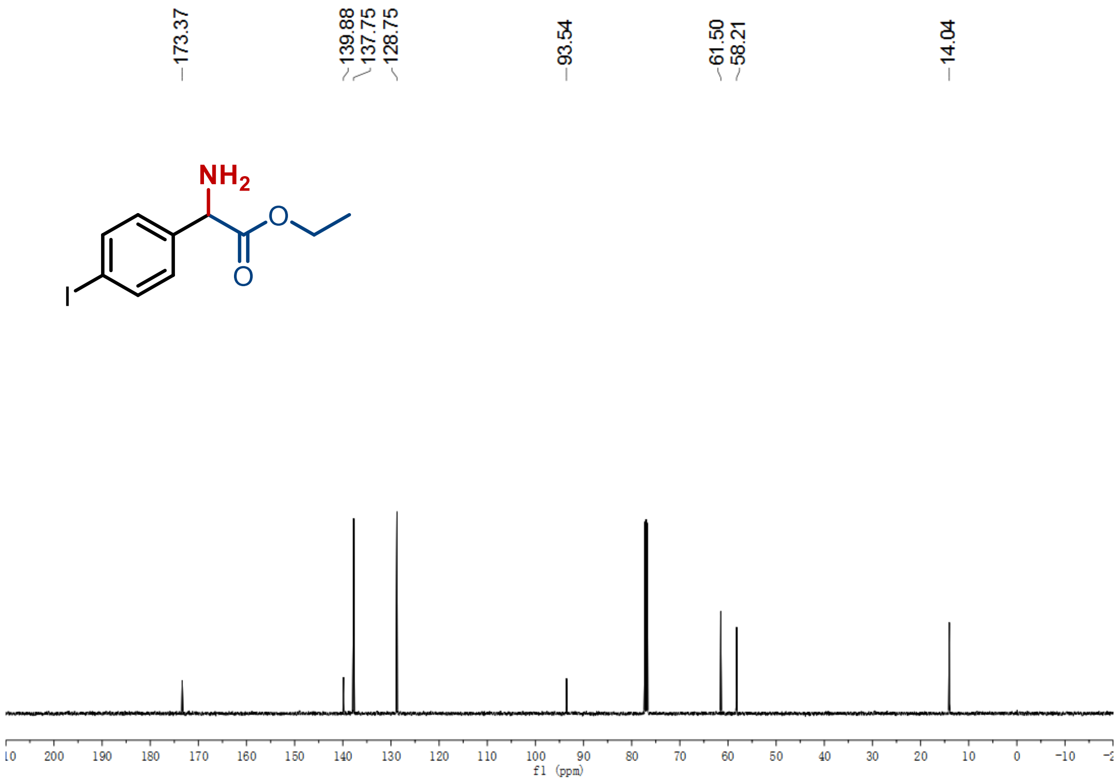

Supplementary Fig. 29 ^{13}C NMR (126 MHz, $CDCl_3$) spectrum of compound **11**.

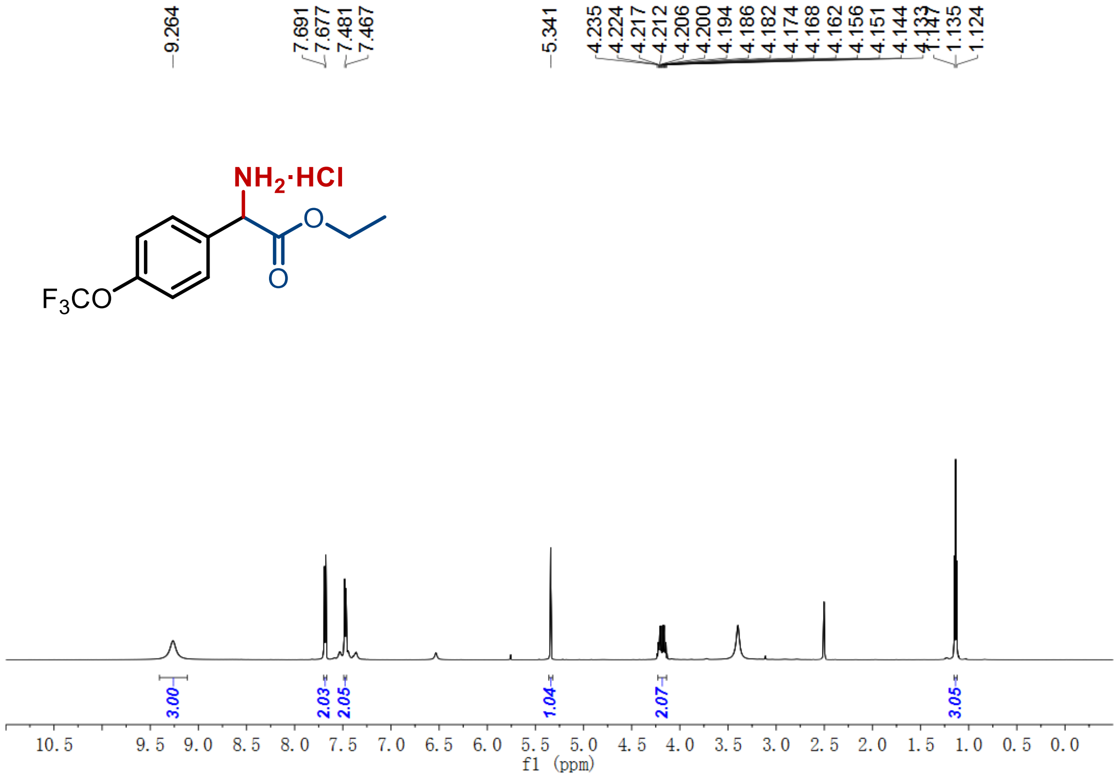

Supplementary Fig. 30 ^{1}H NMR (600 MHz, DMSO) spectrum of compound **12**.

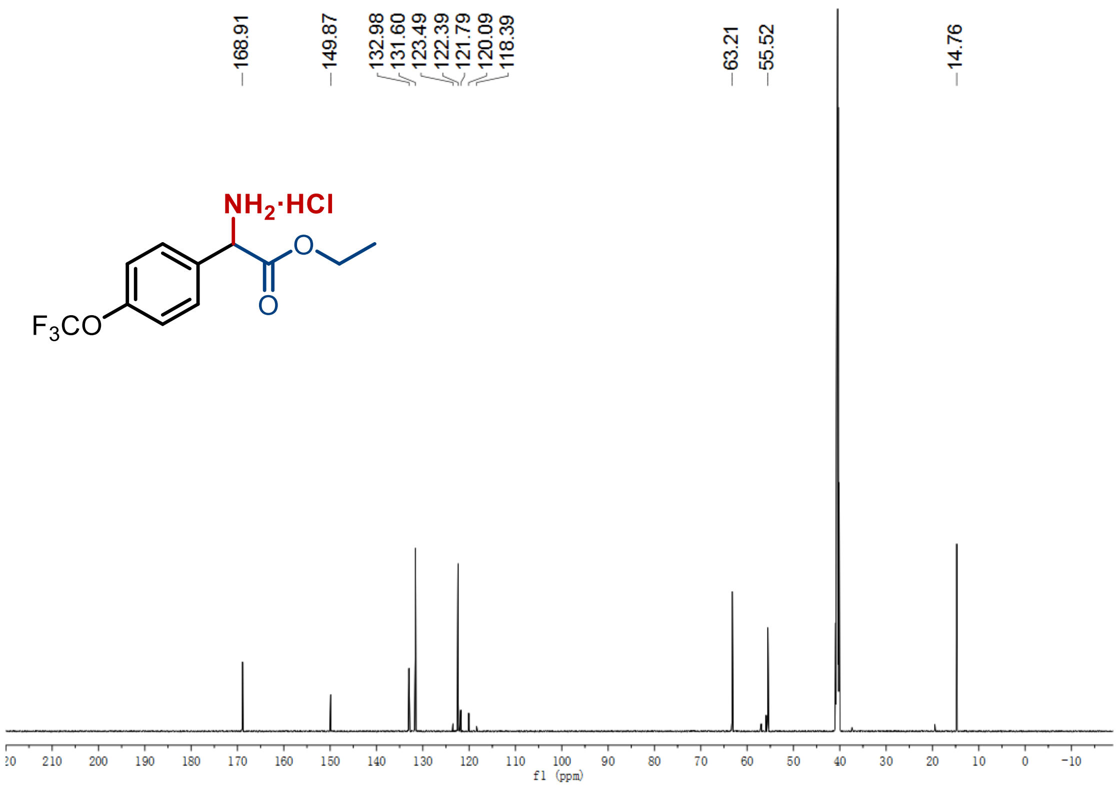

Supplementary Fig. 31 ^{13}C NMR (151 MHz, DMSO) spectrum of compound **12**.

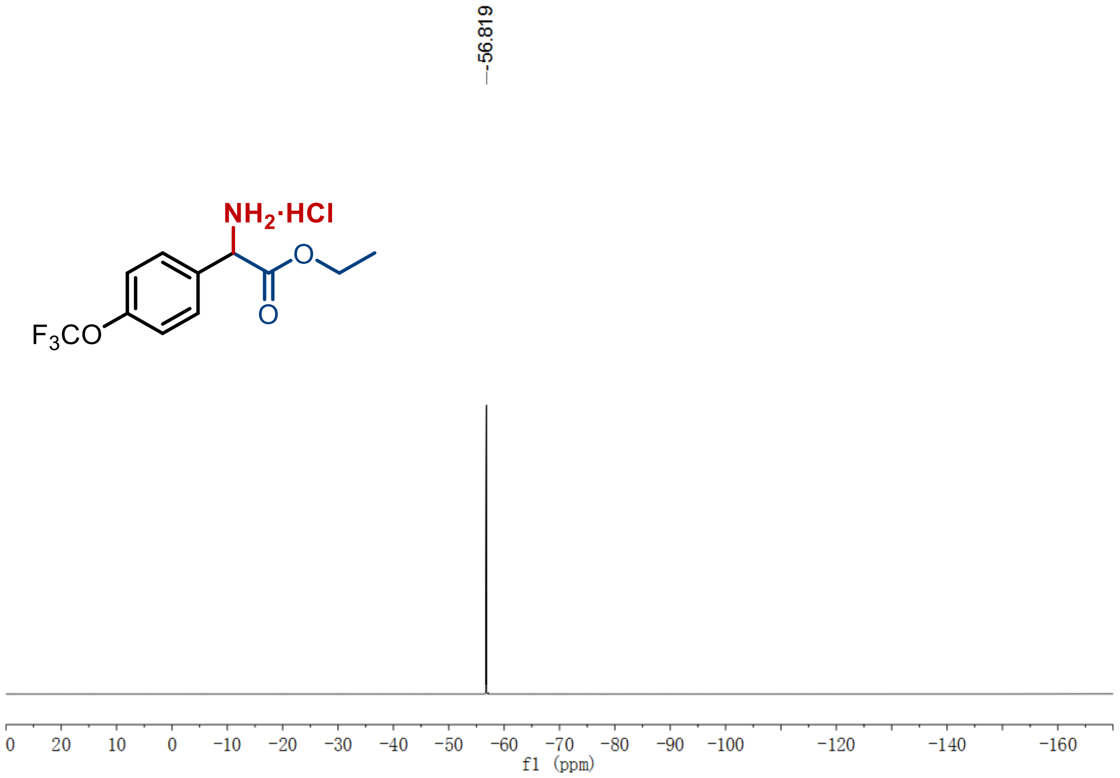

Supplementary Fig. 32 ^{19}F NMR (565 MHz, DMSO) spectrum of compound **12**.

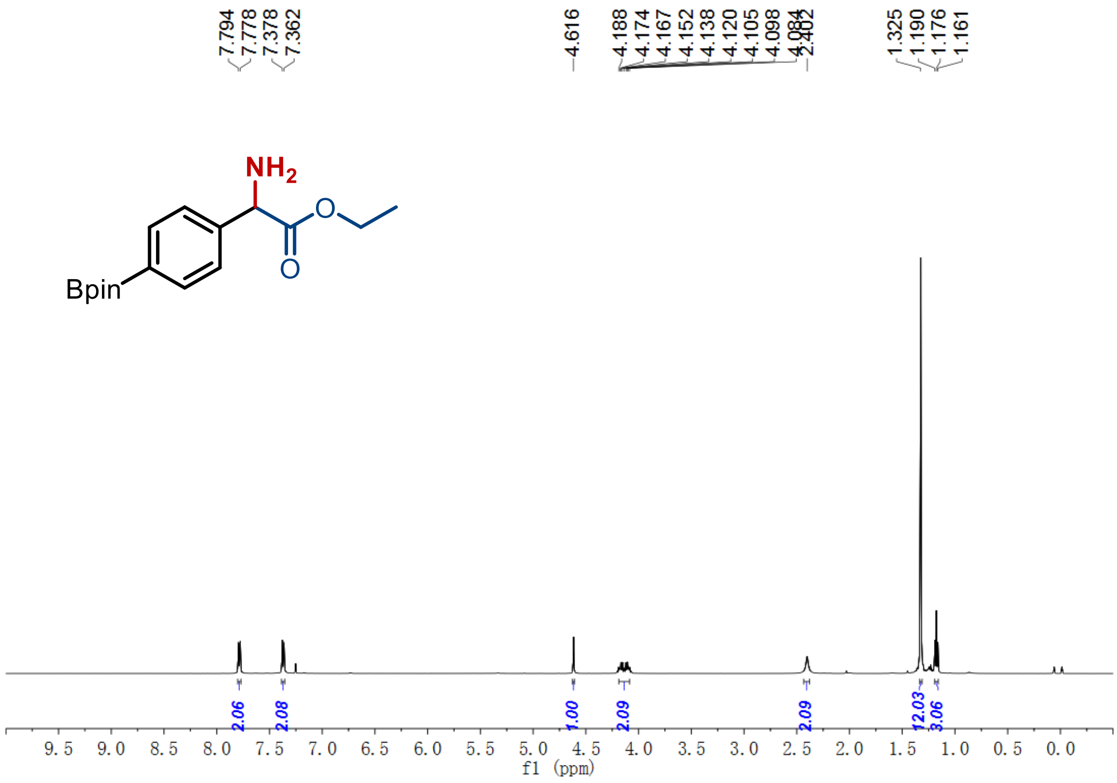

Supplementary Fig. 33 ^{1}H NMR (500 MHz, $CDCl_3$) spectrum of compound **13**.

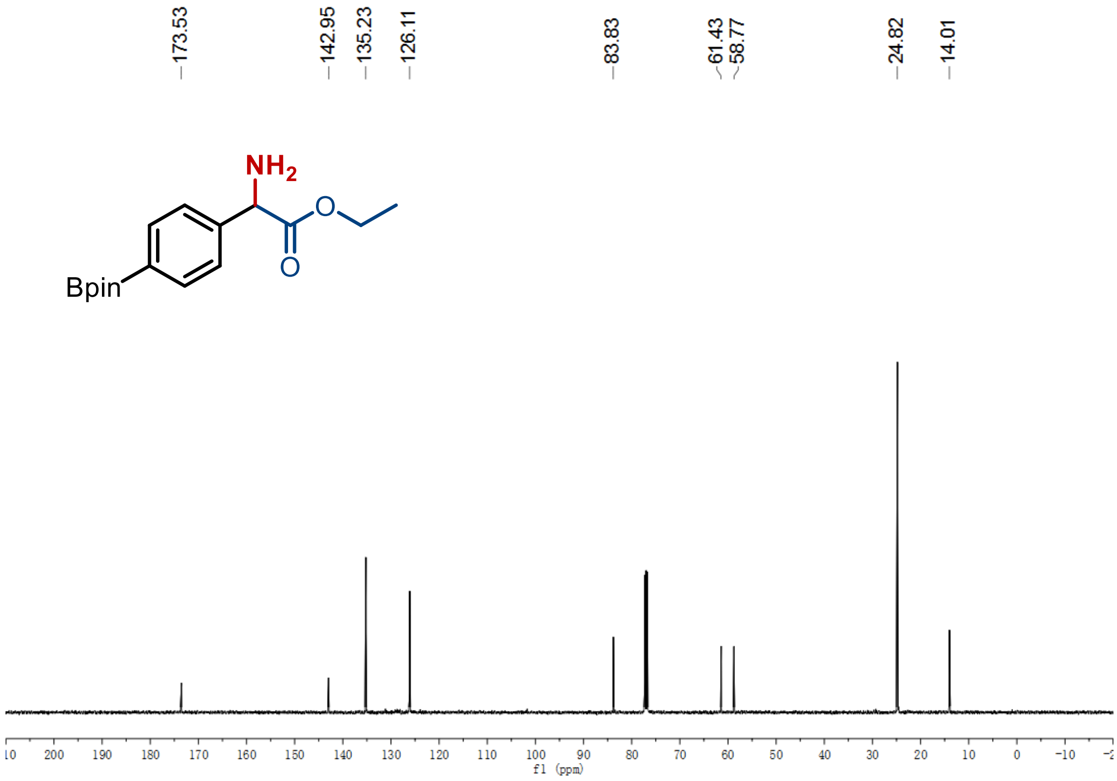

Supplementary Fig. 34 ^{13}C NMR (126 MHz, $CDCl_3$) spectrum of compound **13**.

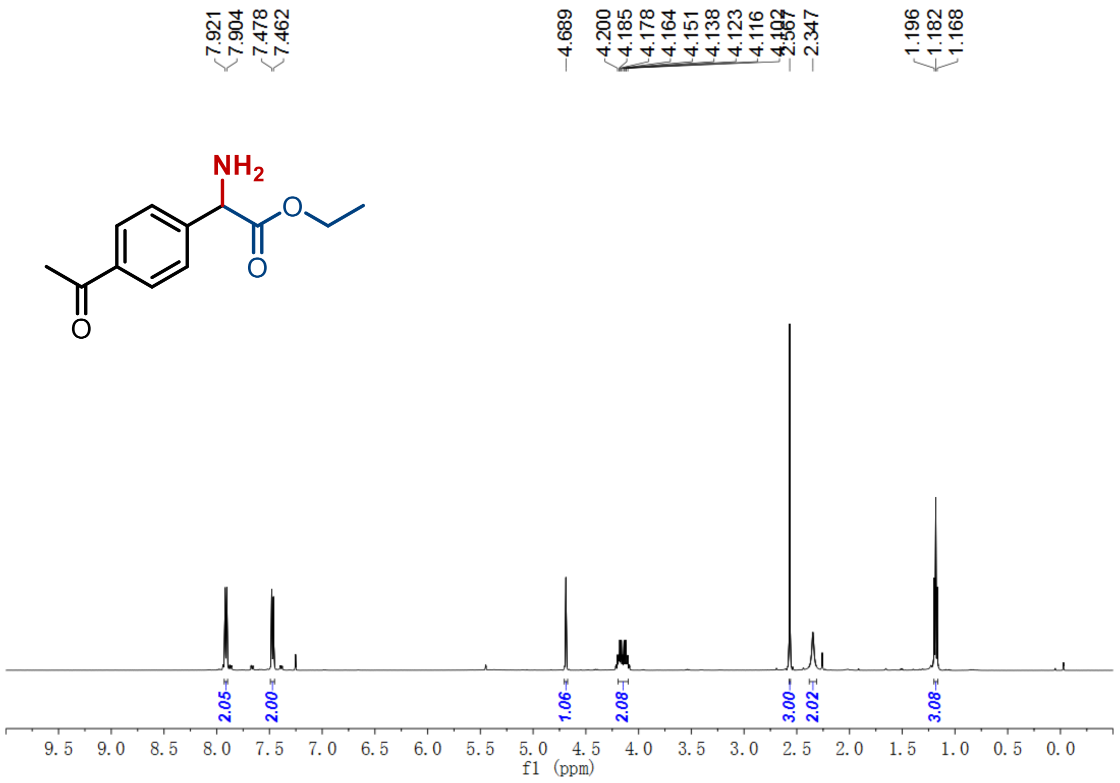

Supplementary Fig. 35 ^{1}H NMR (500 MHz, $CDCl_3$) spectrum of compound **14**.

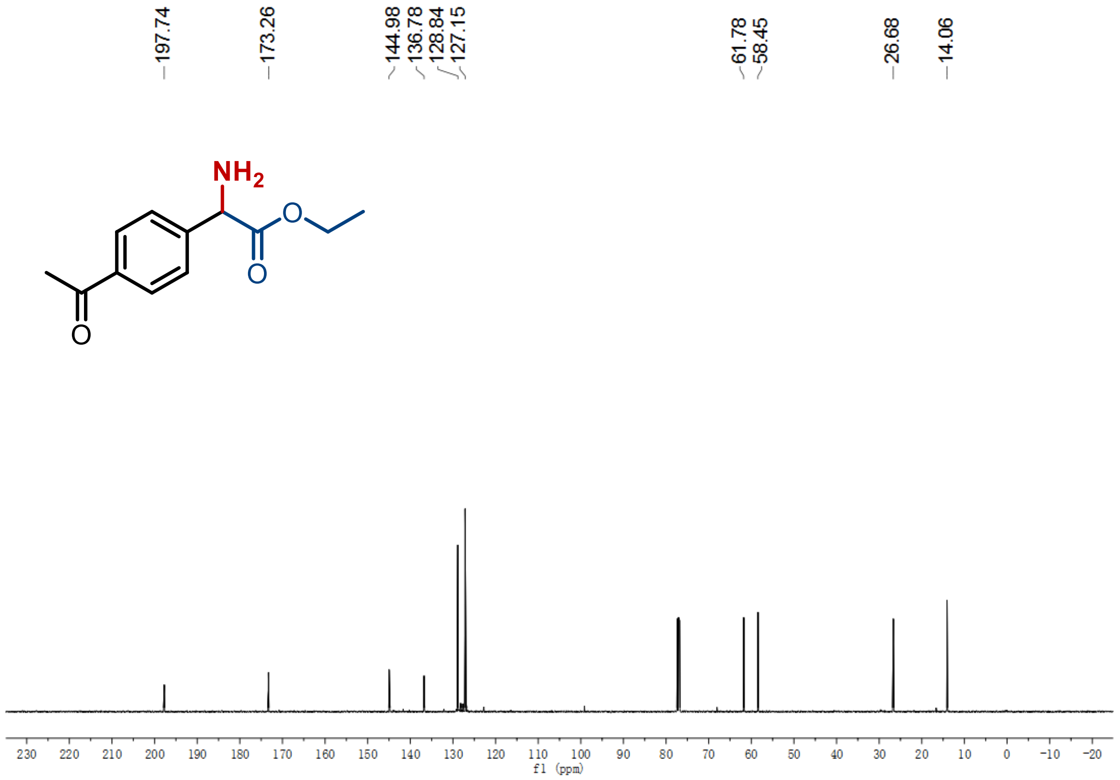

Supplementary Fig. 36 ^{13}C NMR (126 MHz, $CDCl_3$) spectrum of compound **14**.

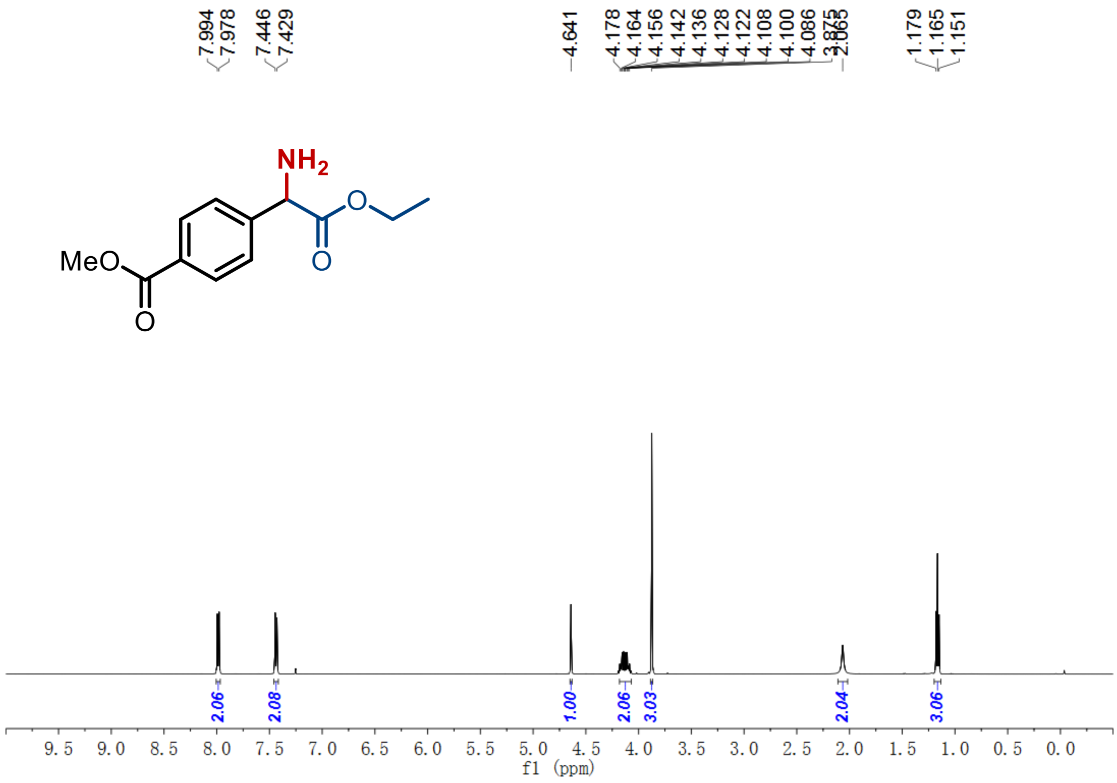

Supplementary Fig. 37 ^{1}H NMR (500 MHz, $CDCl_3$) spectrum of compound **15**.

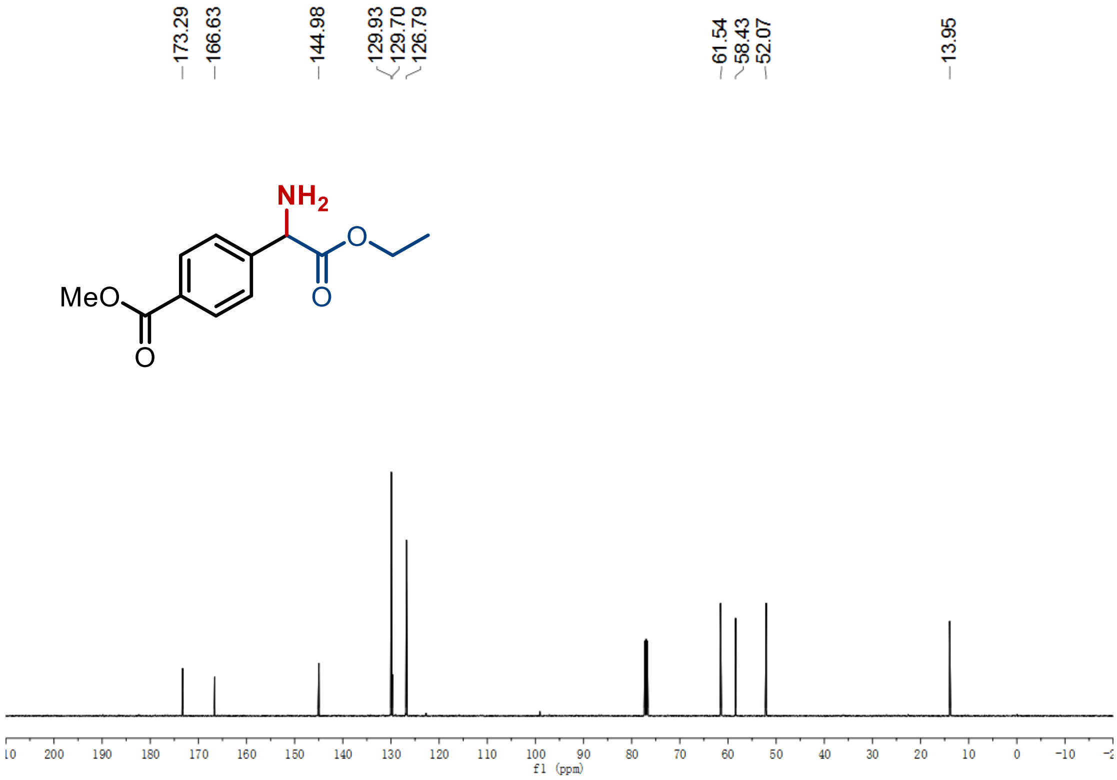

Supplementary Fig. 38 ^{13}C NMR (126 MHz, $CDCl_3$) spectrum of compound **15**.

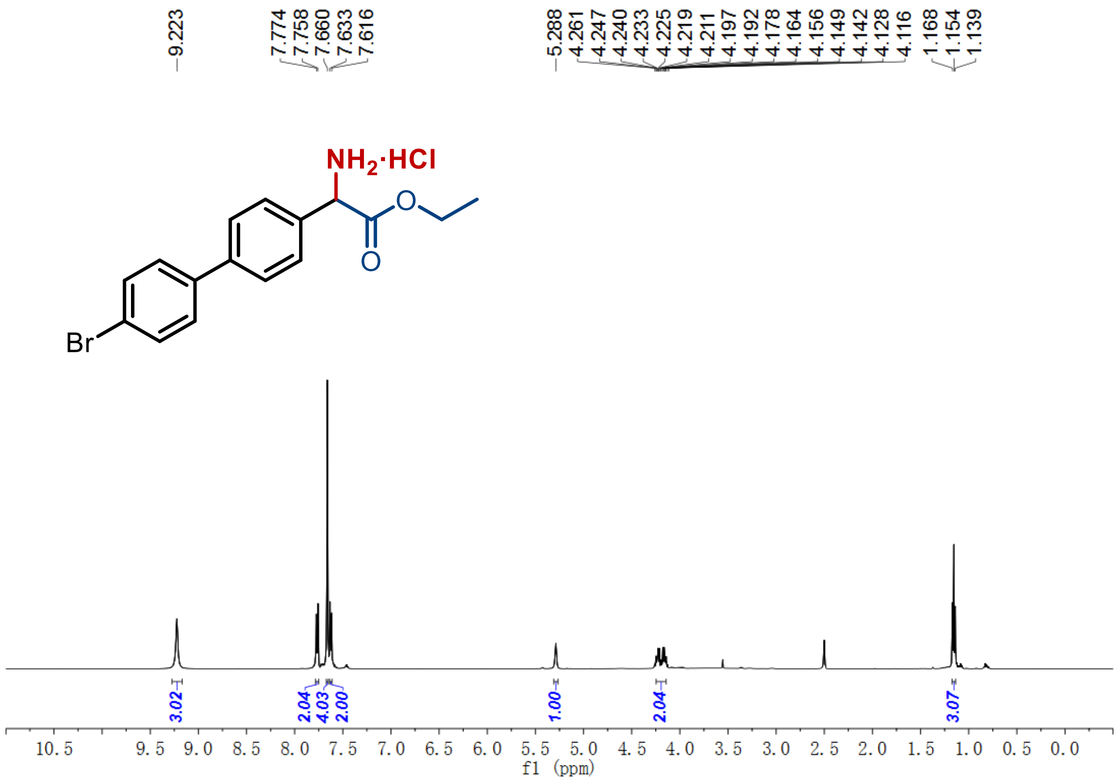

Supplementary Fig. 39 ^{1}H NMR (500 MHz, DMSO) spectrum of compound **16**.

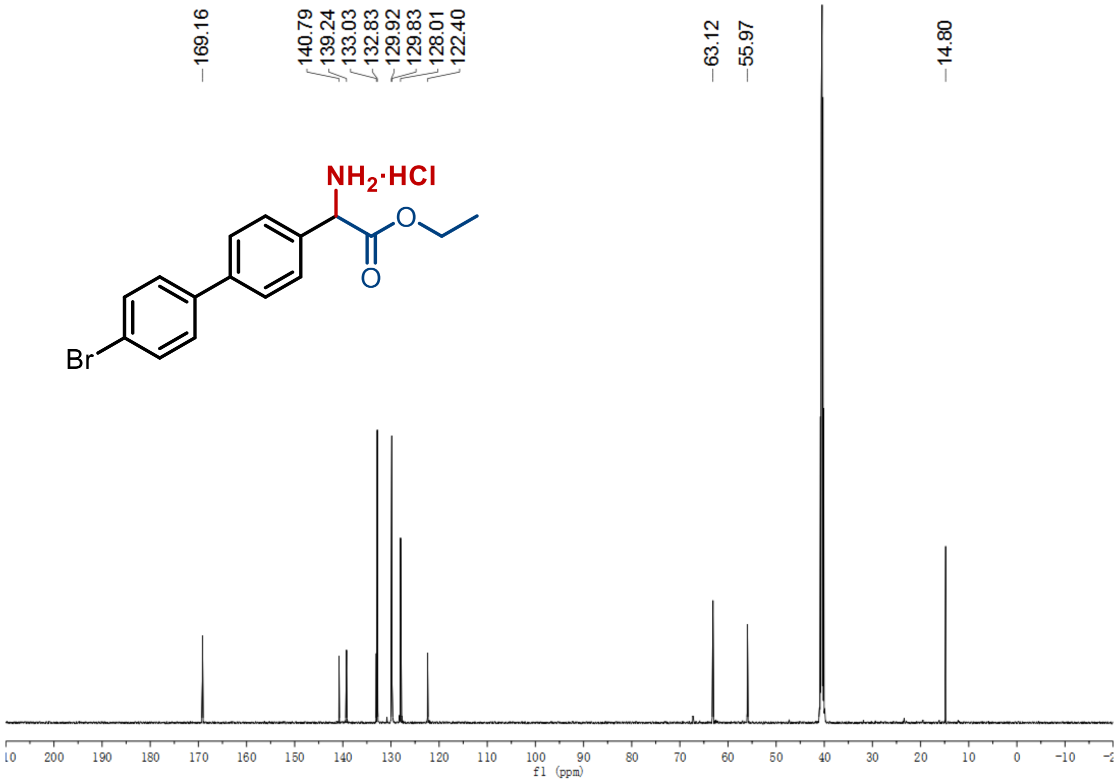

Supplementary Fig. 40 ^{13}C NMR (126 MHz, DMSO) spectrum of compound **16**.

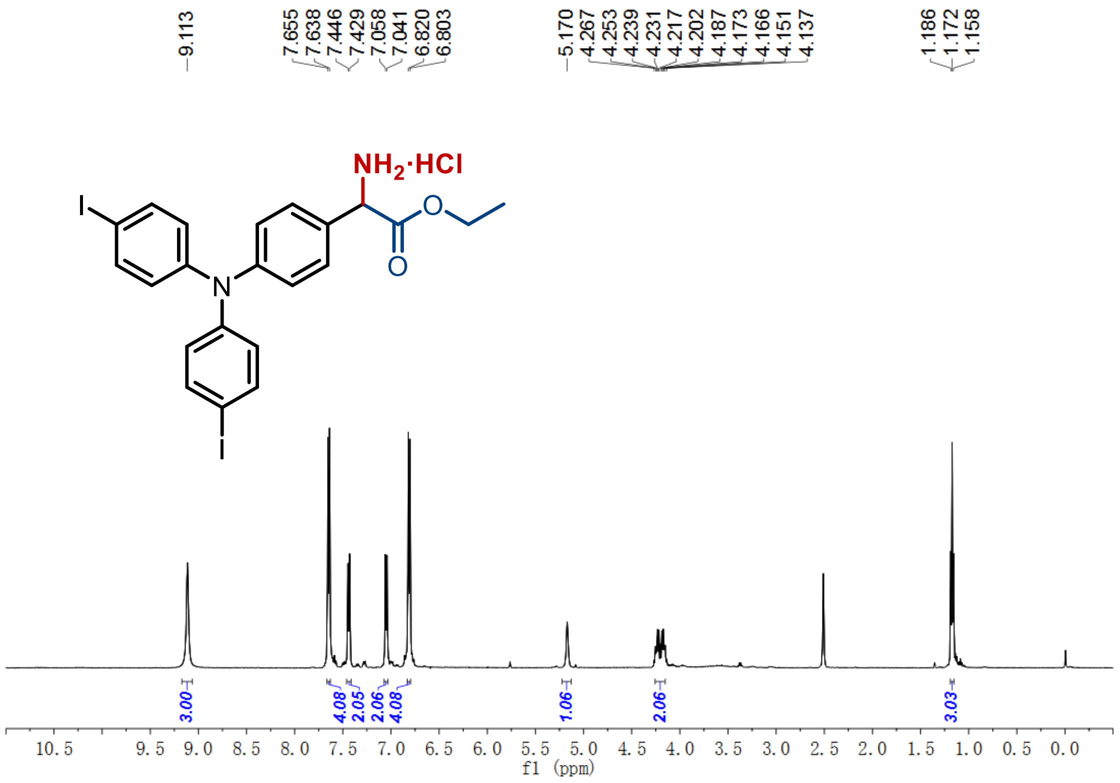

Supplementary Fig. 41 ^{1}H NMR (500 MHz, DMSO) spectrum of compound **17**.

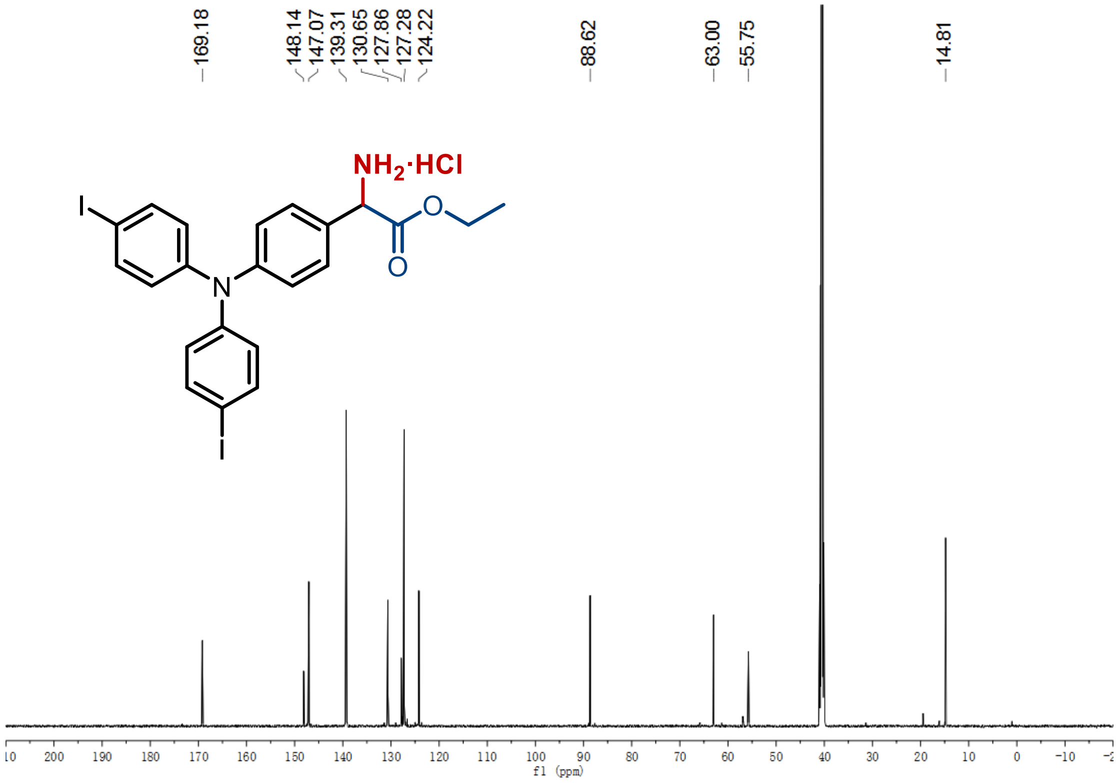

Supplementary Fig. 42 ^{13}C NMR (126 MHz, DMSO) spectrum of compound **17**.

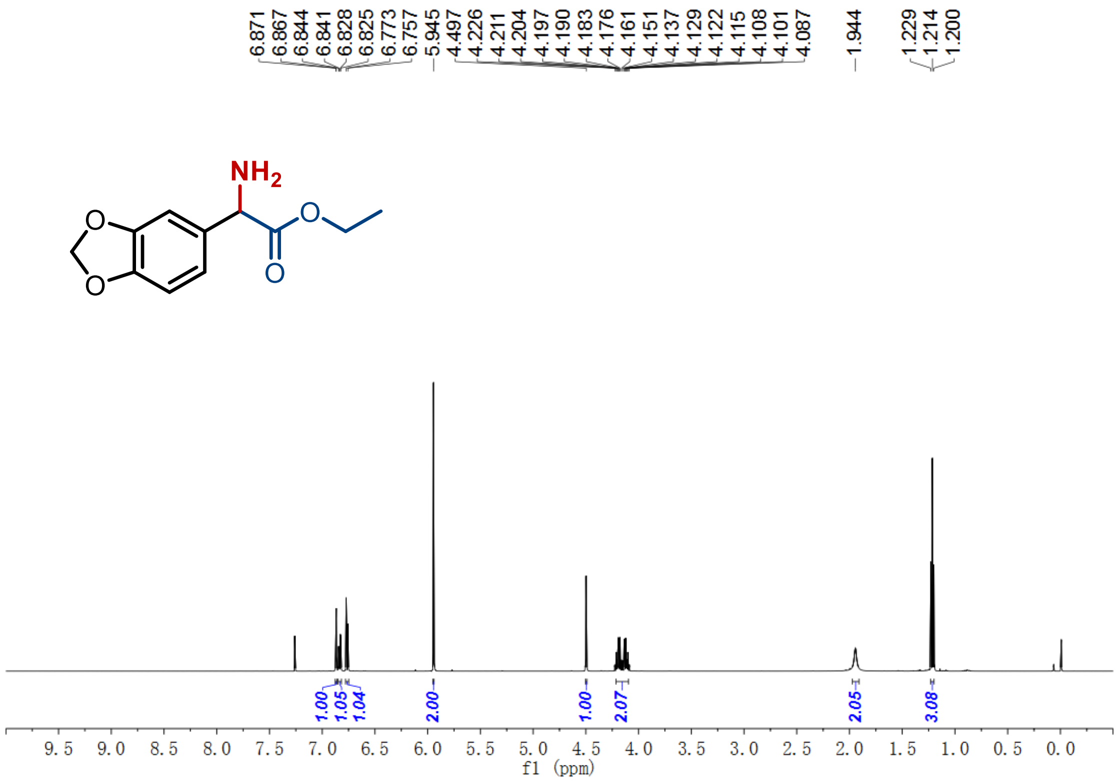

Supplementary Fig. 43 ^{1}H NMR (500 MHz, $CDCl_3$) spectrum of compound **18**.

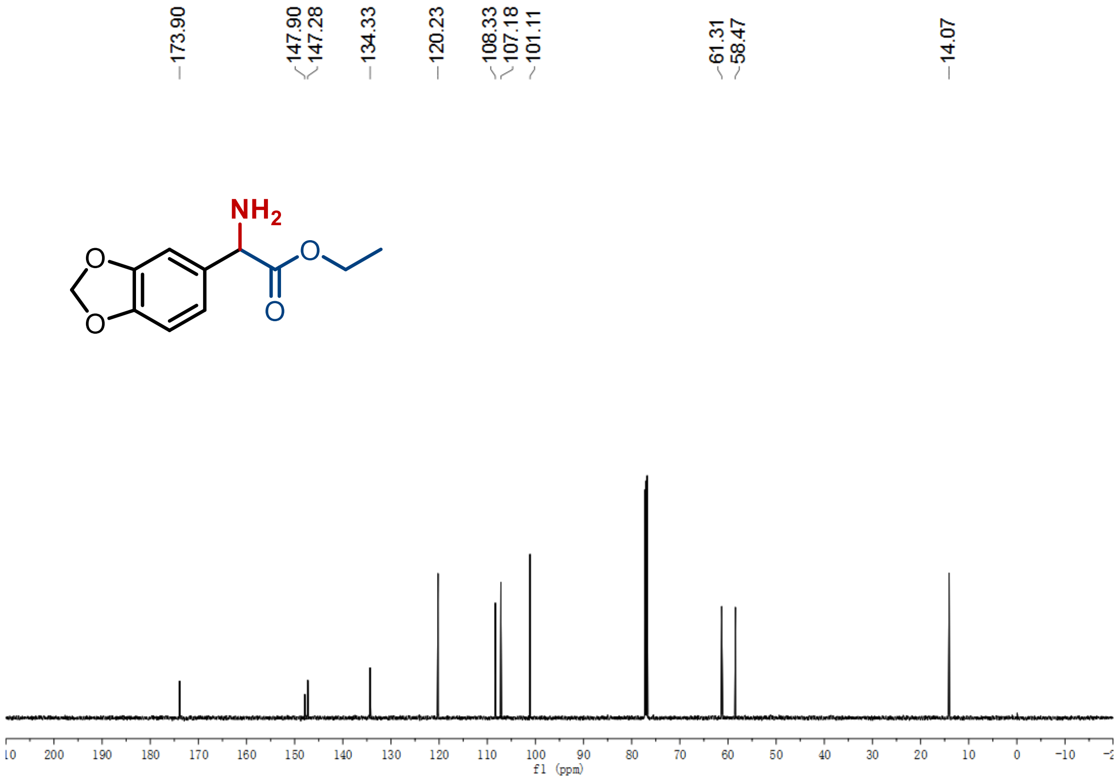

Supplementary Fig. 44 ^{13}C NMR (151 MHz, $CDCl_3$) spectrum of compound **18**.

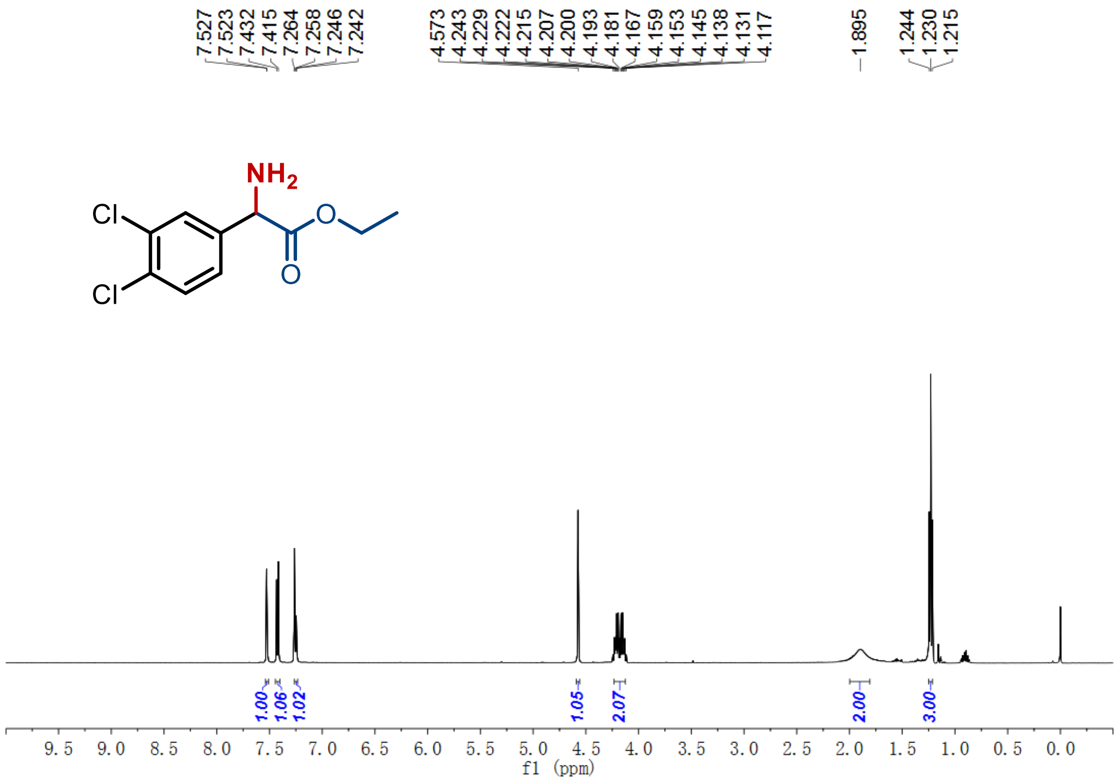

Supplementary Fig. 45 ^{1}H NMR (500 MHz, $CDCl_3$) spectrum of compound **19**.

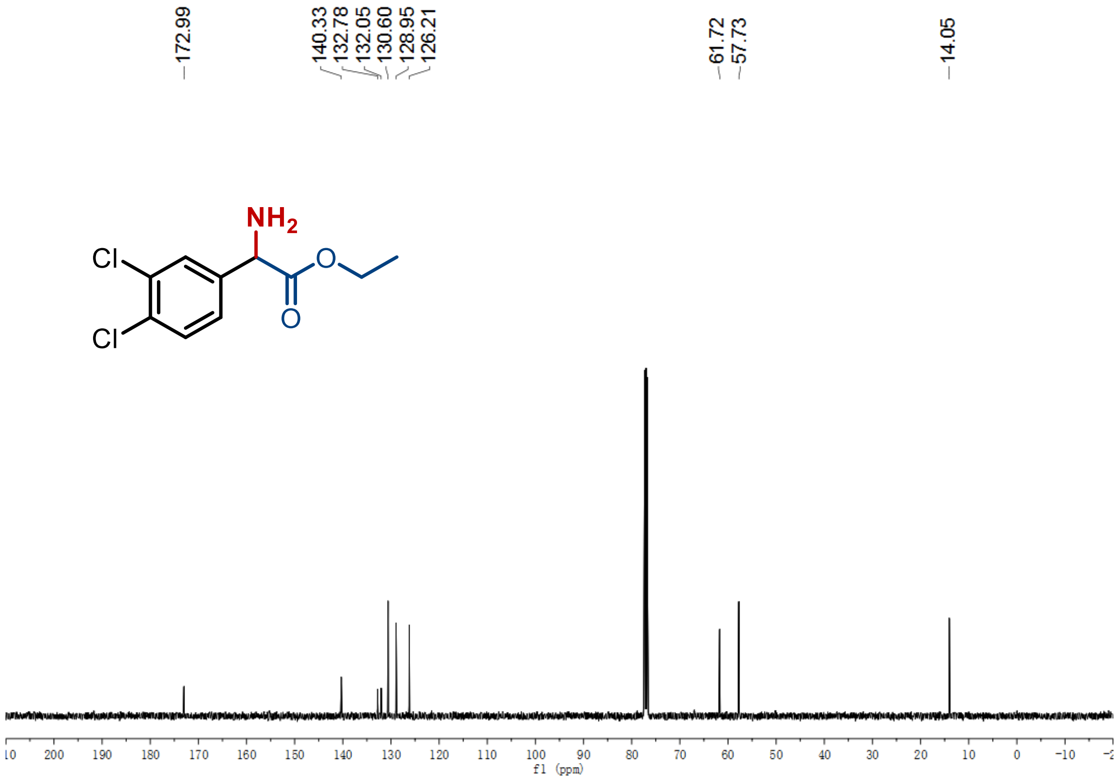

Supplementary Fig. 46 ^{13}C NMR (126 MHz, $CDCl_3$) spectrum of compound **19**.

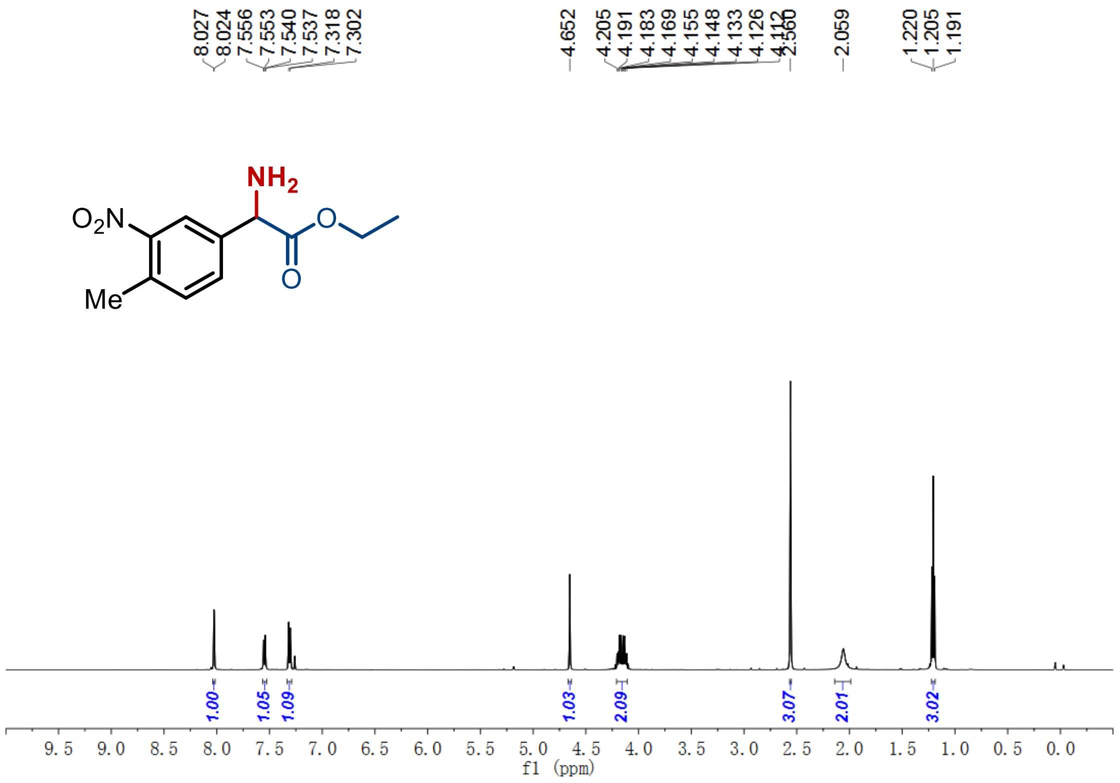

Supplementary Fig. 47 ^{1}H NMR (500 MHz, $CDCl_3$) spectrum of compound **20**.

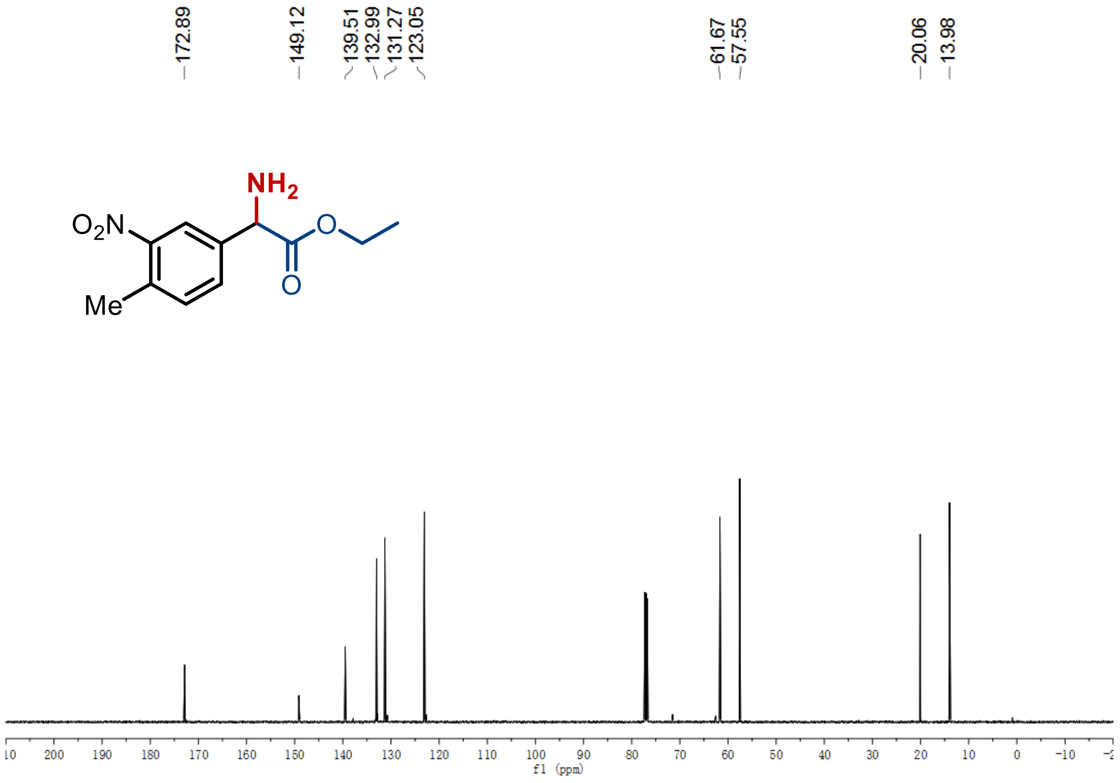

Supplementary Fig. 48 ^{13}C NMR (126 MHz, $CDCl_3$) spectrum of compound **20**.

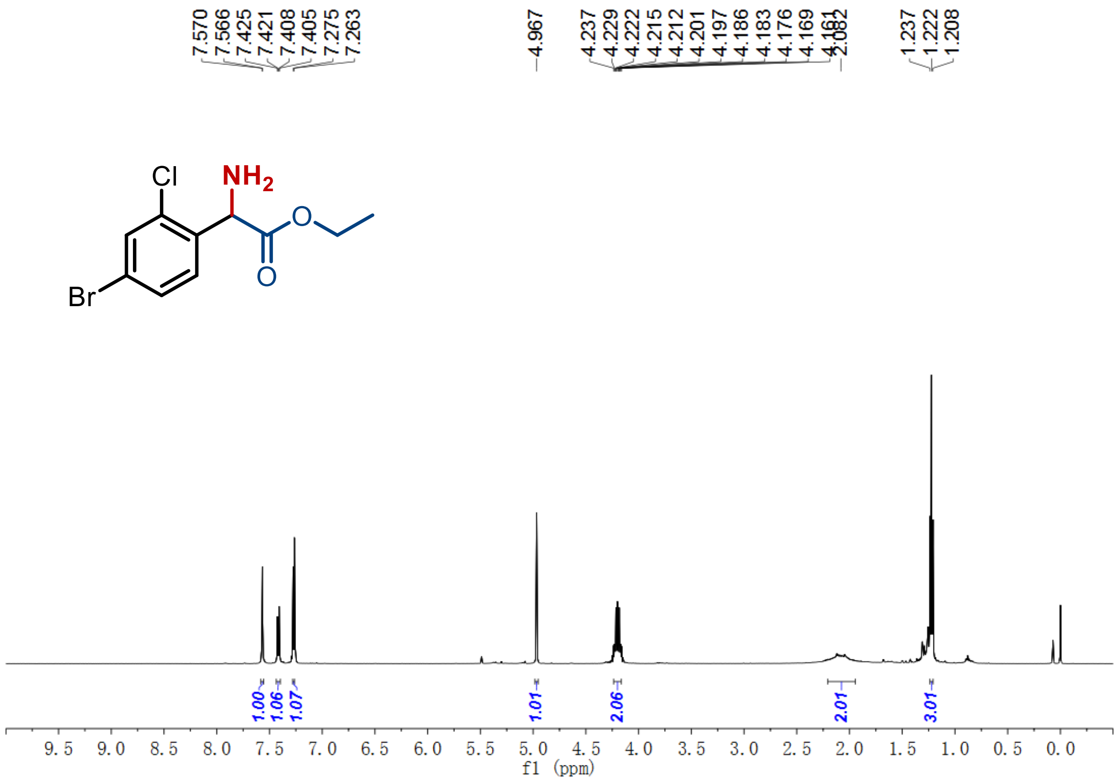

Supplementary Fig. 49 ^{1}H NMR (500 MHz, $CDCl_3$) spectrum of compound **21**.

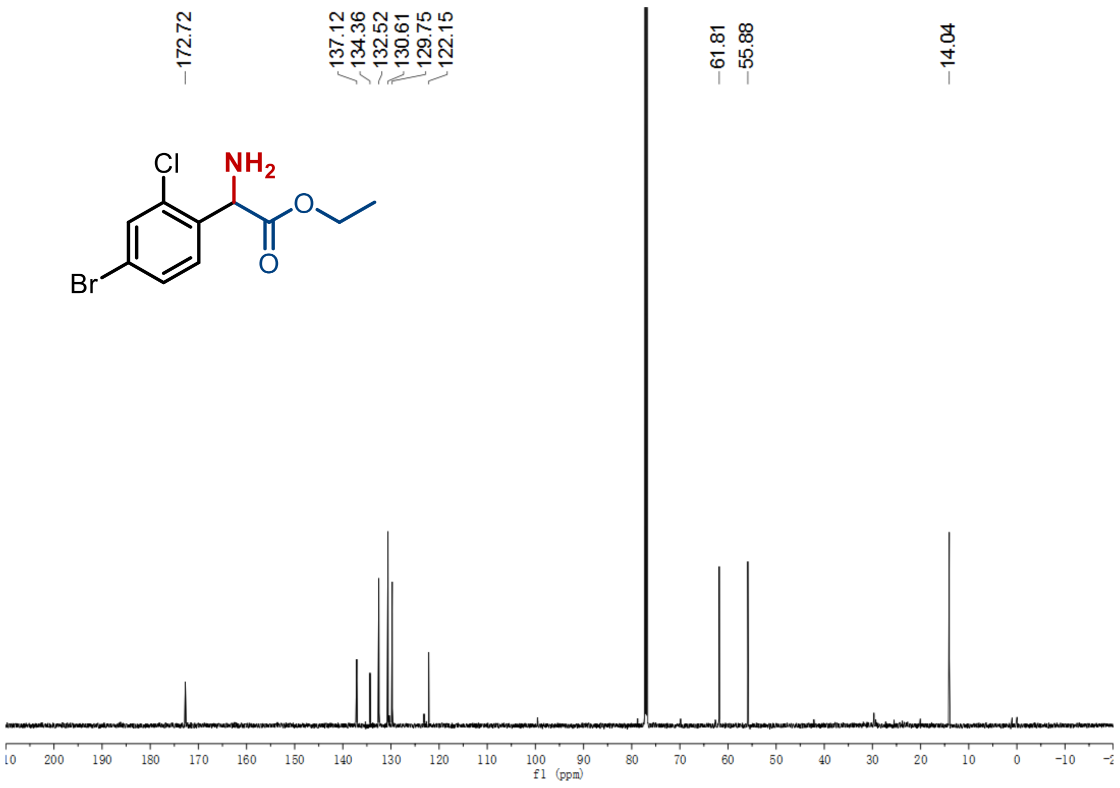

Supplementary Fig. 50 ^{13}C NMR (151 MHz, $CDCl_3$) spectrum of compound **21**.

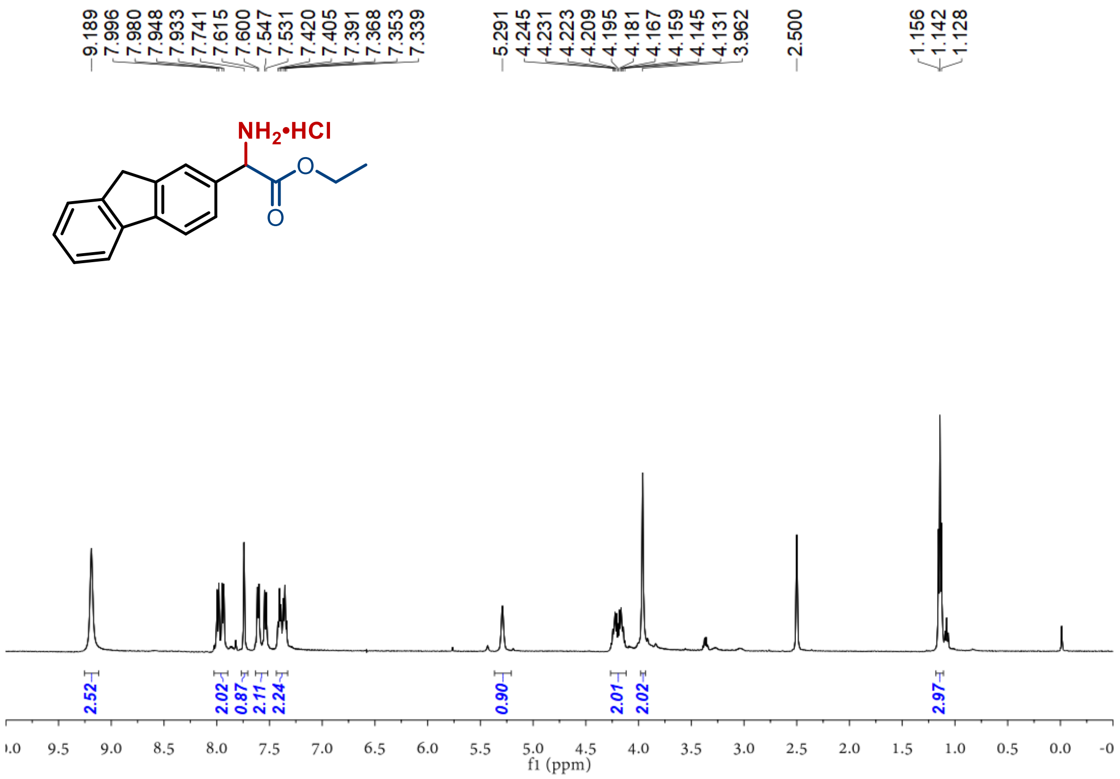

Supplementary Fig. 51 ^{1}H NMR (500 MHz, DMSO) spectrum of compound **22**.

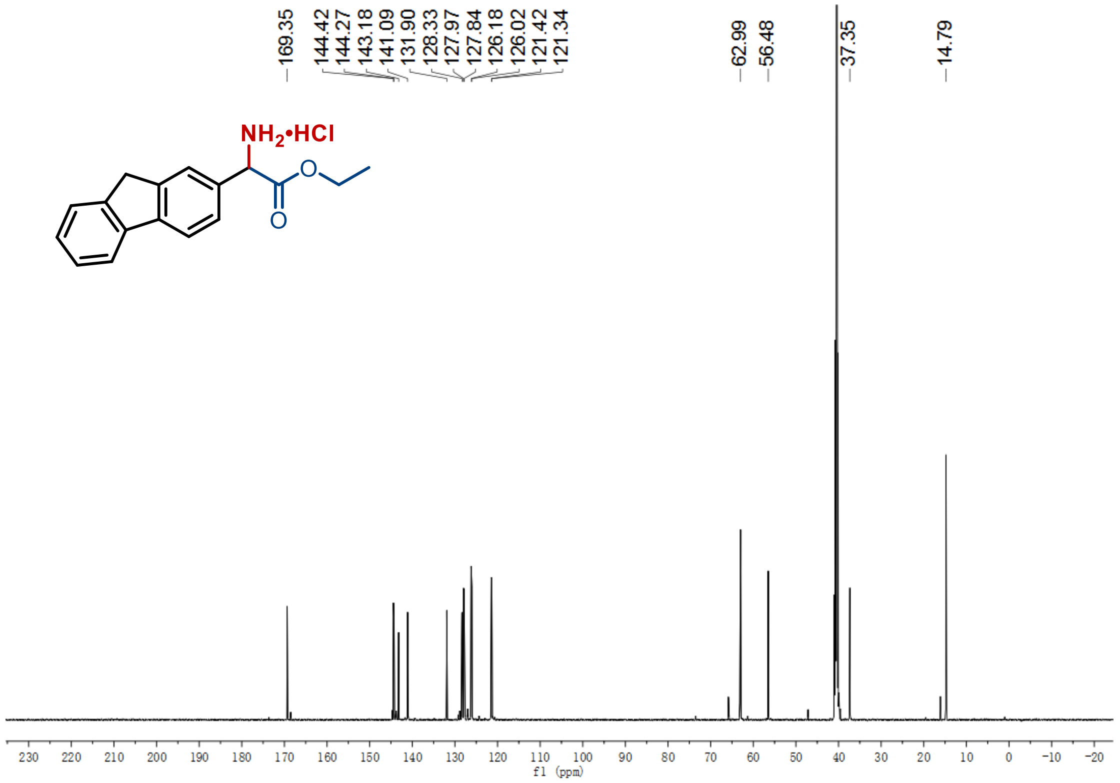

Supplementary Fig. 52 ^{13}C NMR (126 MHz, DMSO) spectrum of compound **22**.

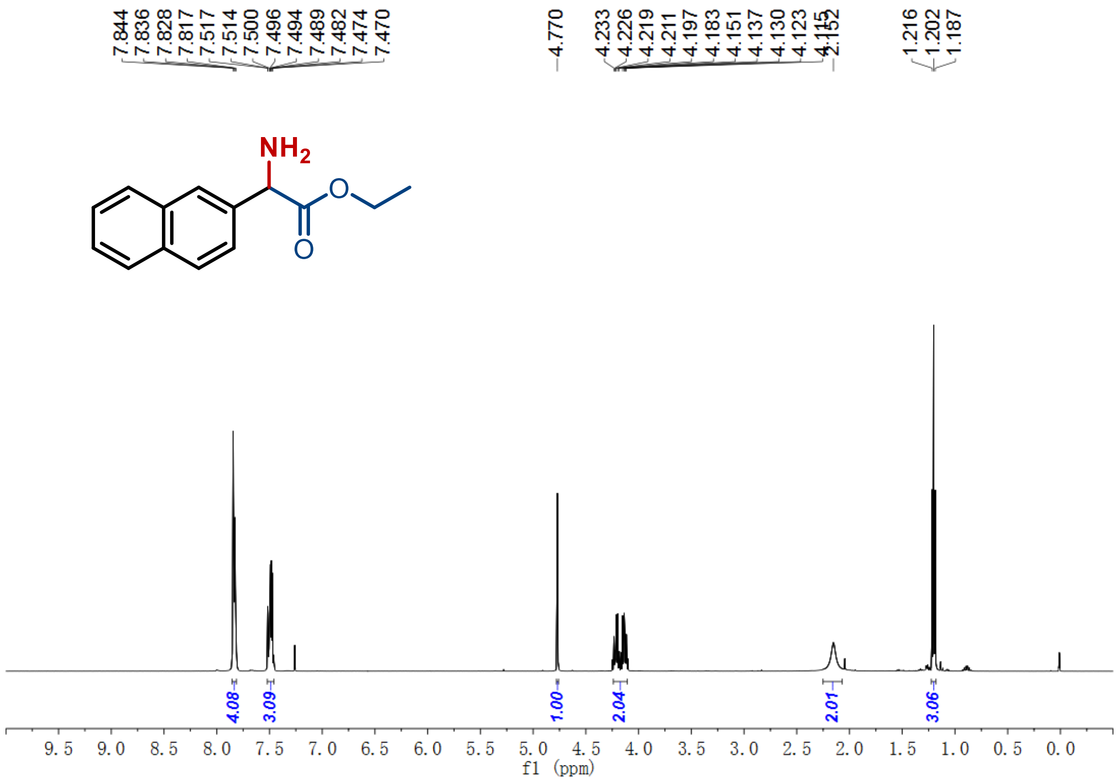

Supplementary Fig. 53 ^{1}H NMR (500 MHz, $CDCl_3$) spectrum of compound **23**.

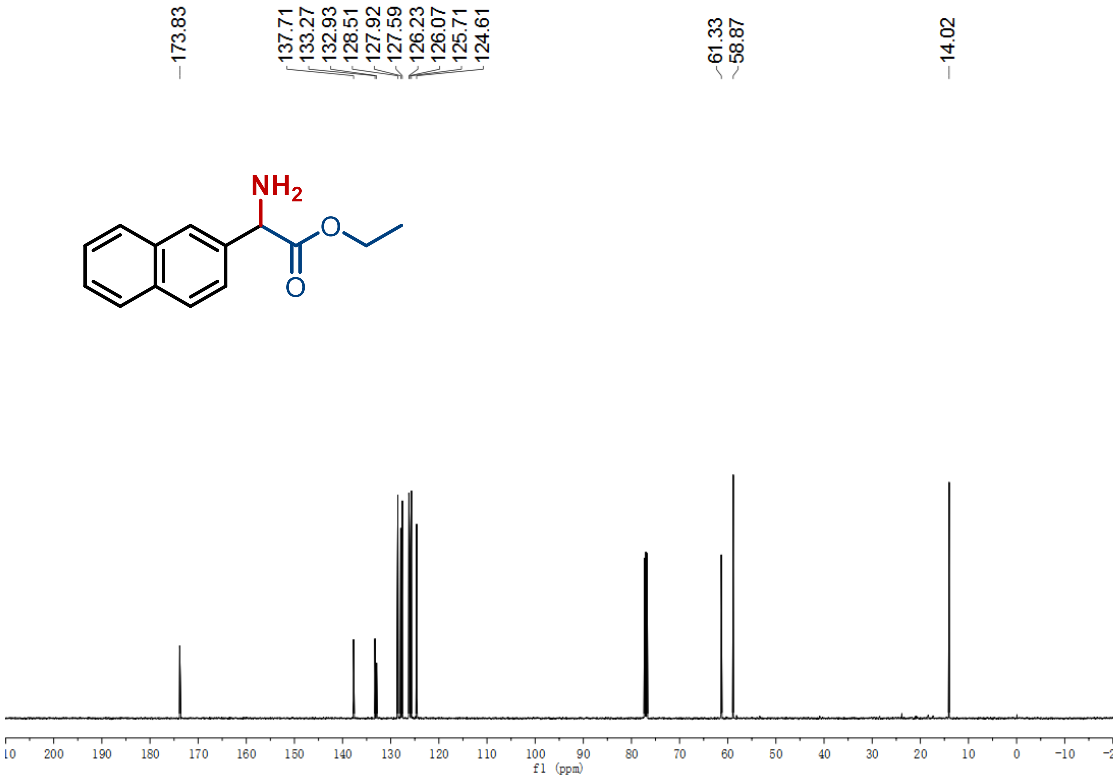

Supplementary Fig. 54 ^{13}C NMR (126 MHz, $CDCl_3$) spectrum of compound **23**.

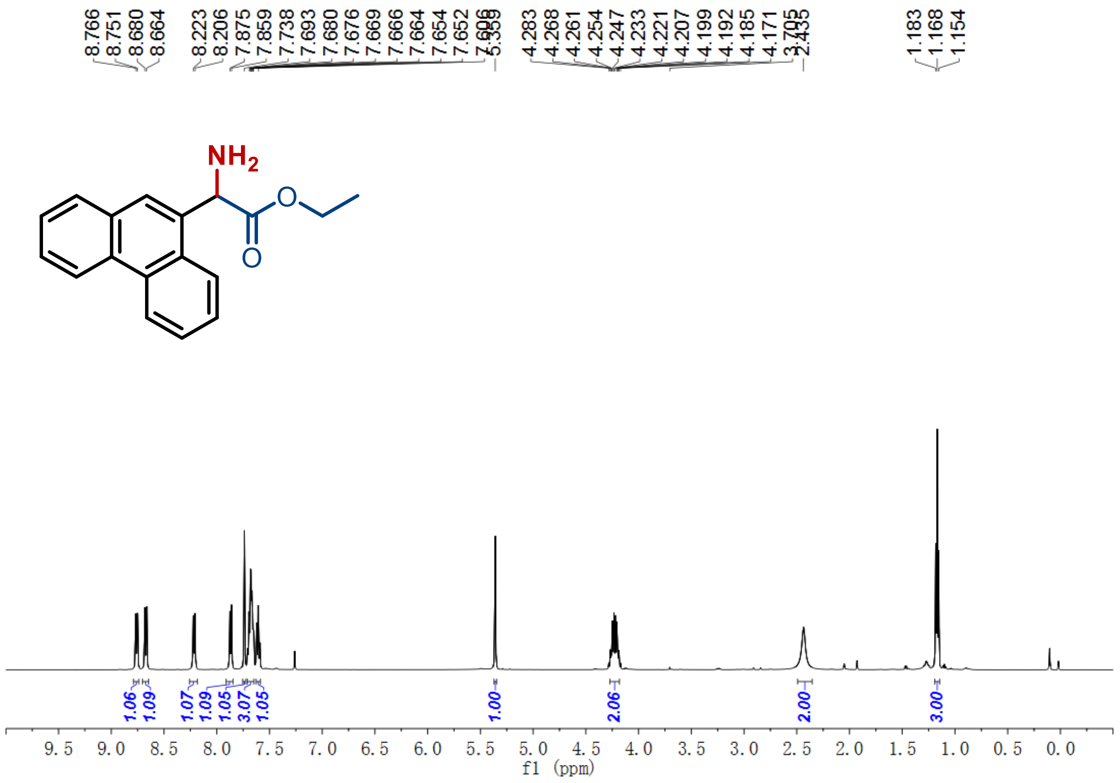

Supplementary Fig. 55 ^{1}H NMR (500 MHz, $CDCl_3$) spectrum of compound **24**.

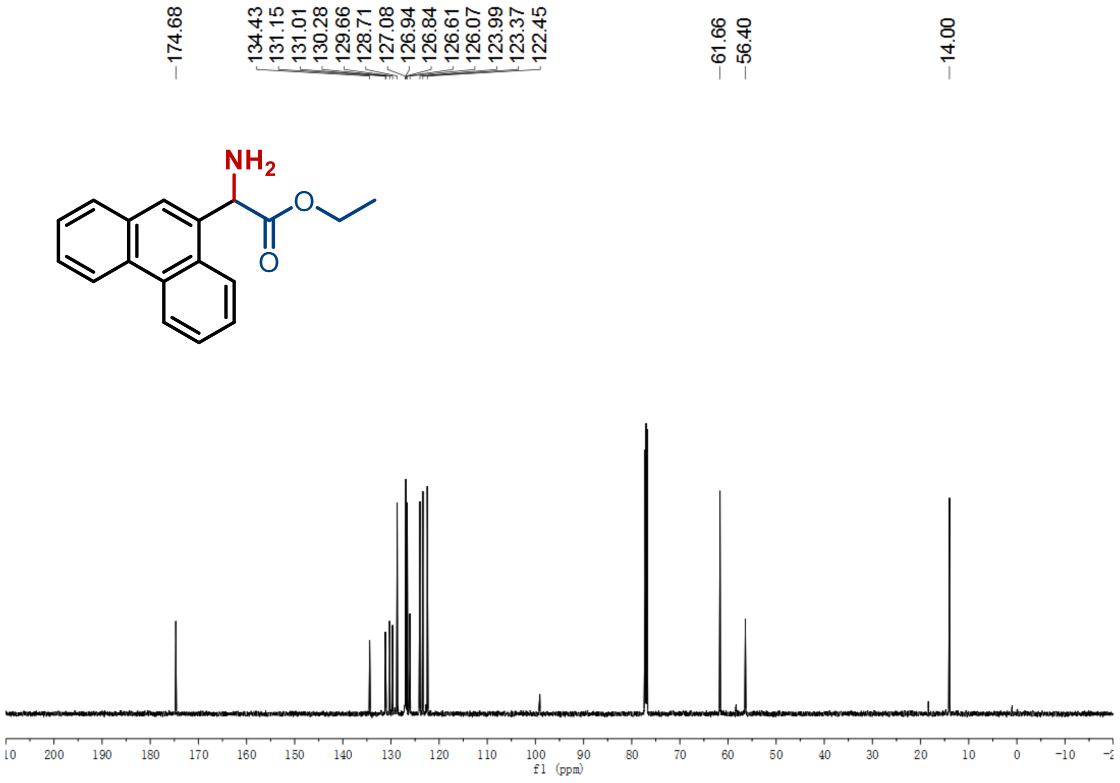

Supplementary Fig. 56 ^{13}C NMR (151 MHz, $CDCl_3$) spectrum of compound **24**.

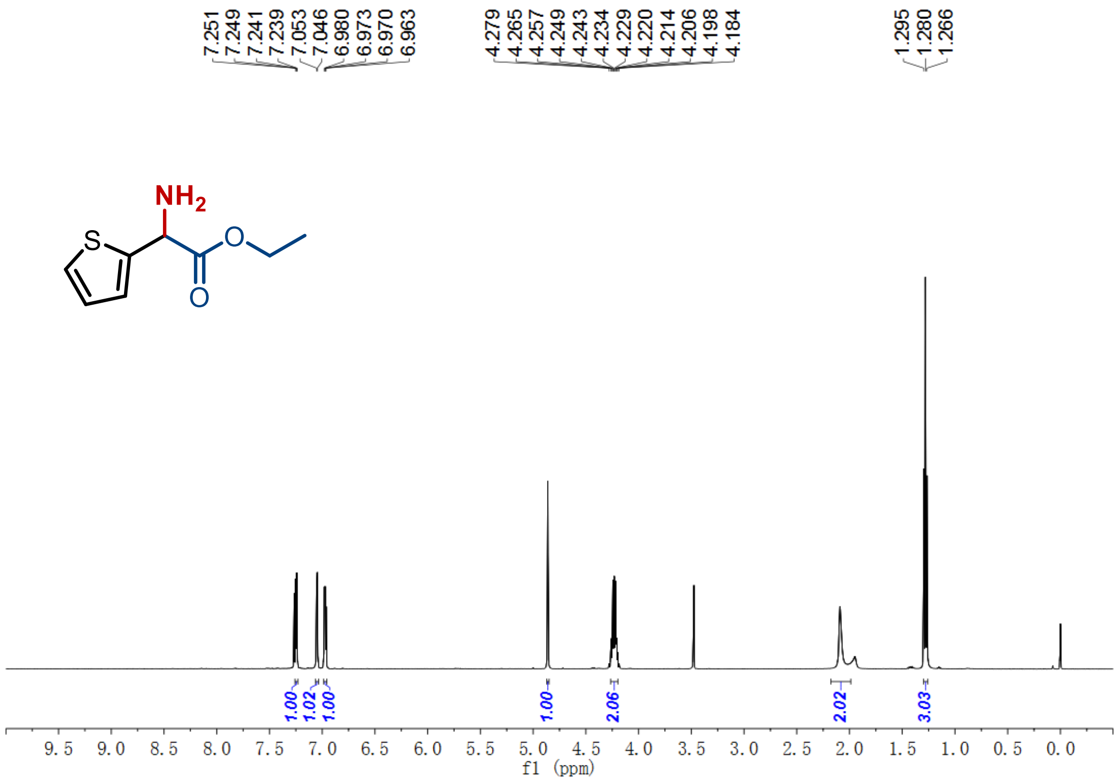

Supplementary Fig. 57 ^{1}H NMR (500 MHz, $CDCl_3$) spectrum of compound **25**.

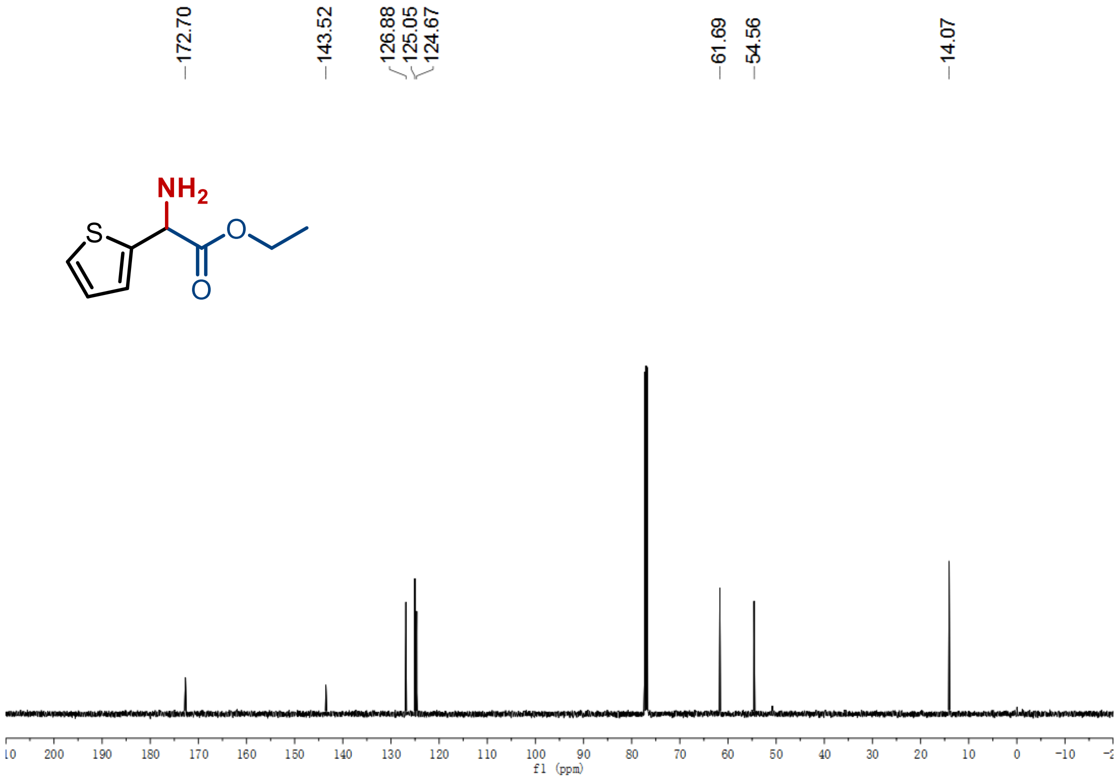

Supplementary Fig. 58 ^{13}C NMR (126 MHz, $CDCl_3$) spectrum of compound **25**.

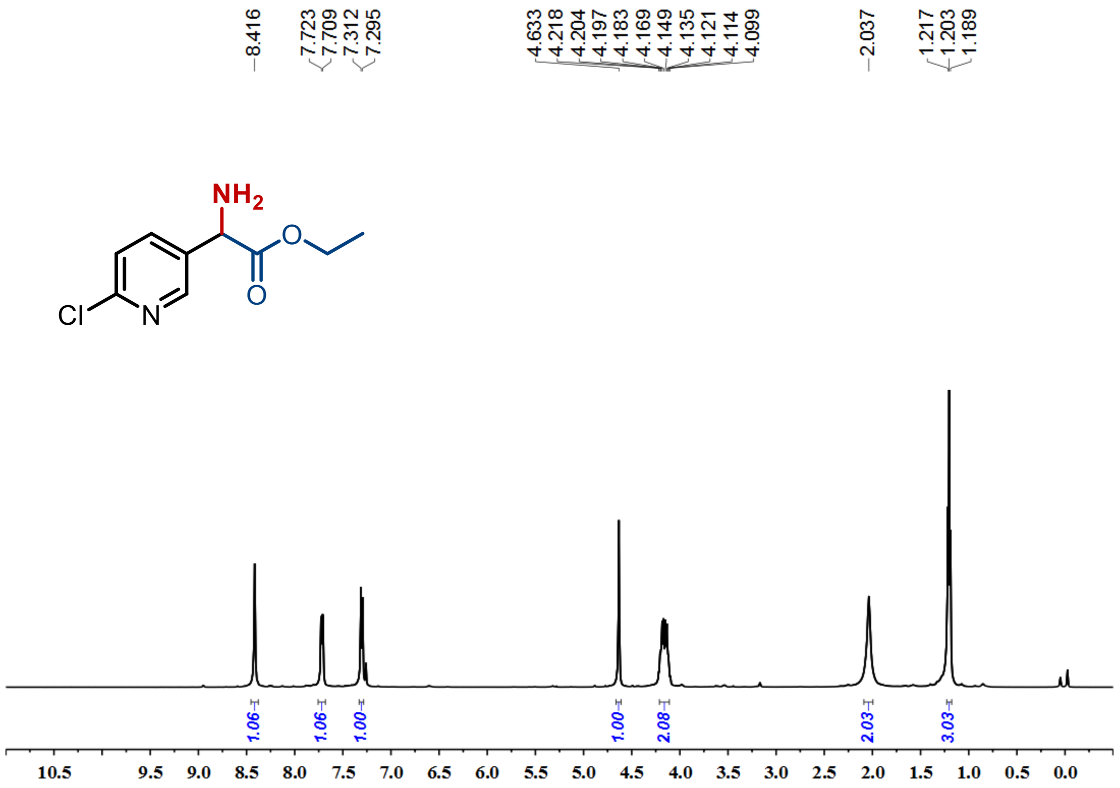

Supplementary Fig. 59 ^{1}H NMR (500 MHz, $CDCl_3$) spectrum of compound **26**.

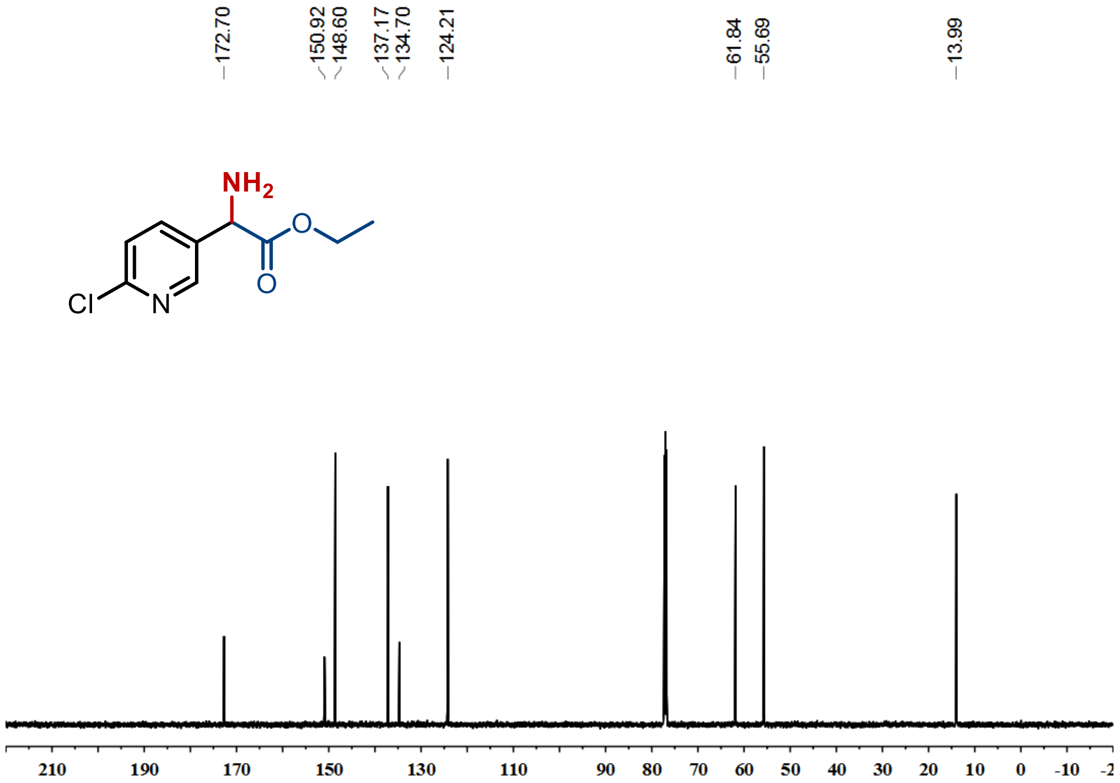

Supplementary Fig. 60 ^{13}C NMR (151 MHz, $CDCl_3$) spectrum of compound **26**.

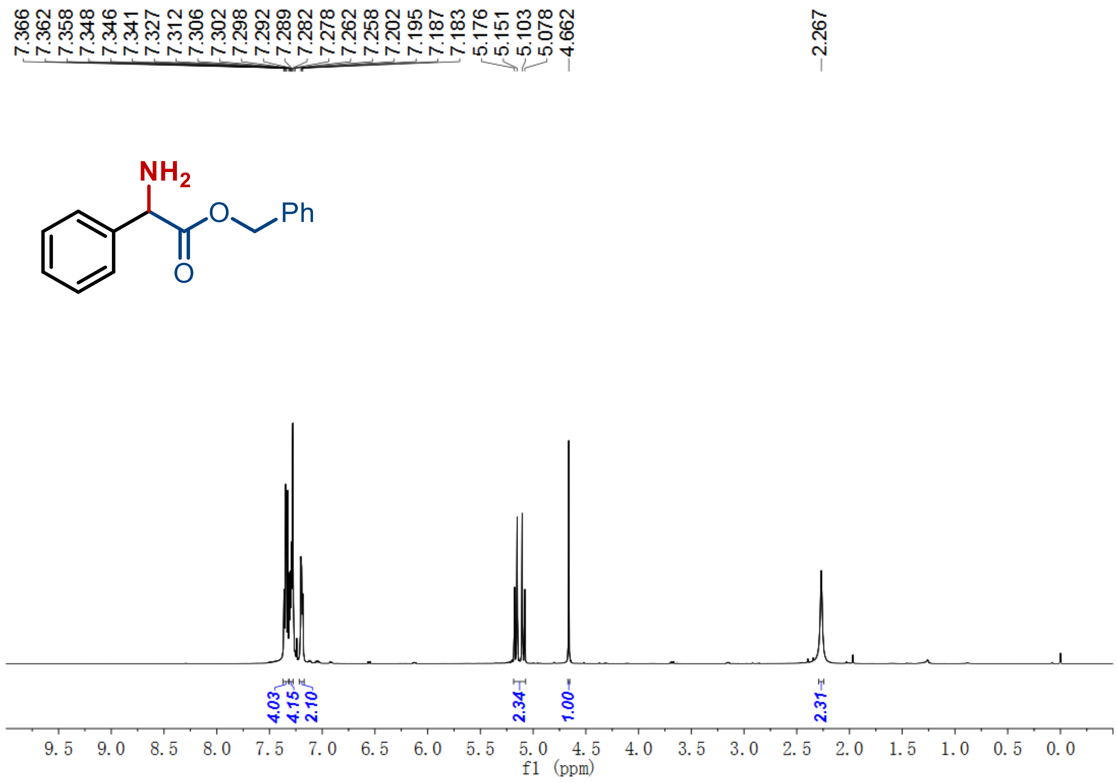

Supplementary Fig. 61 ^{1}H NMR (500 MHz, $CDCl_3$) spectrum of compound **27**.

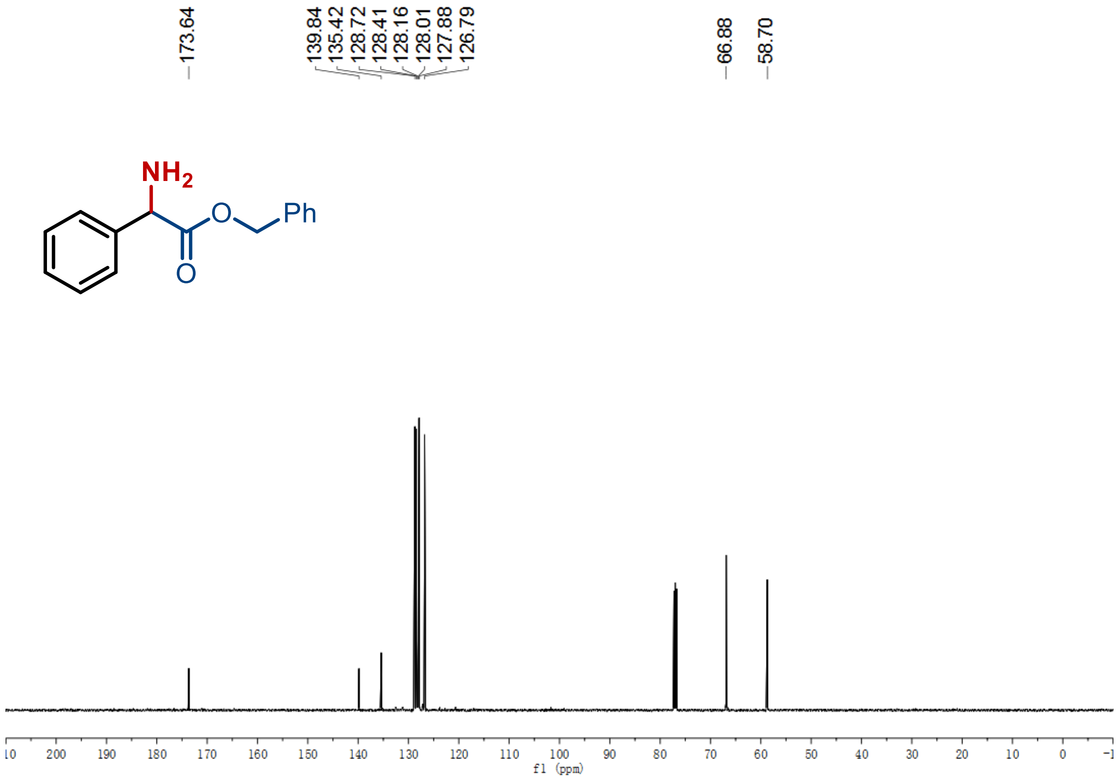

Supplementary Fig. 62 ^{13}C NMR (126 MHz, $CDCl_3$) spectrum of compound **27**.

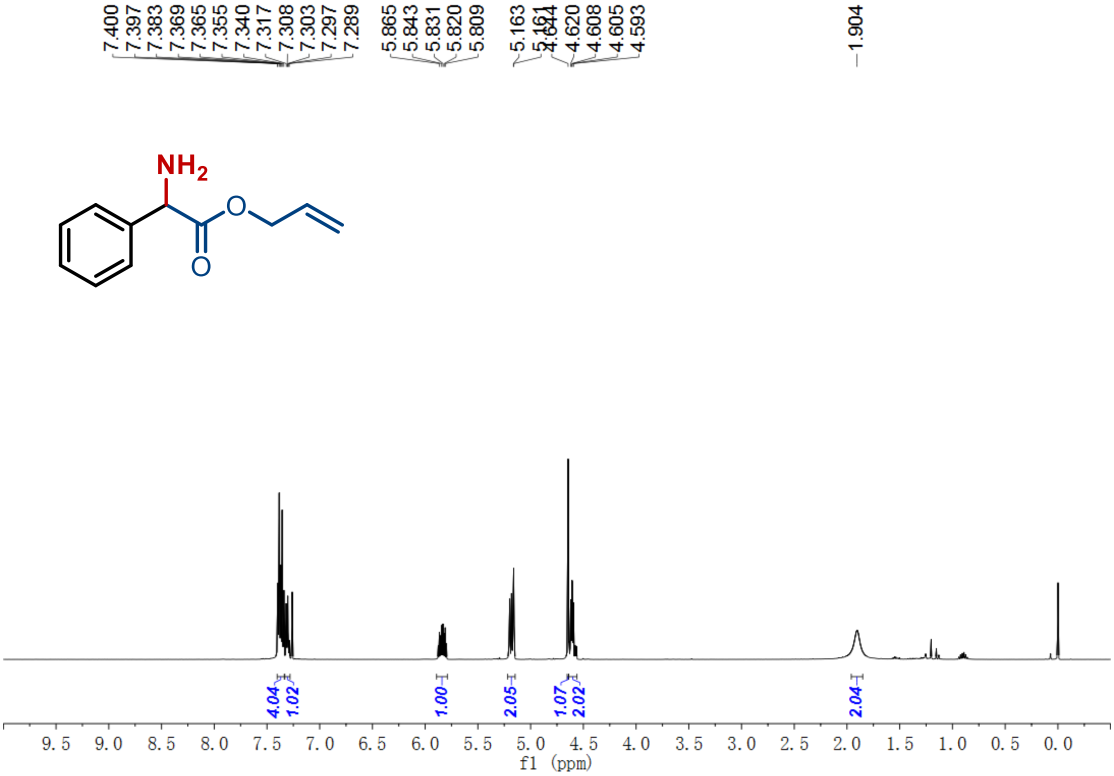

Supplementary Fig. 63 ^{1}H NMR (500 MHz, $CDCl_3$) spectrum of compound **28**.

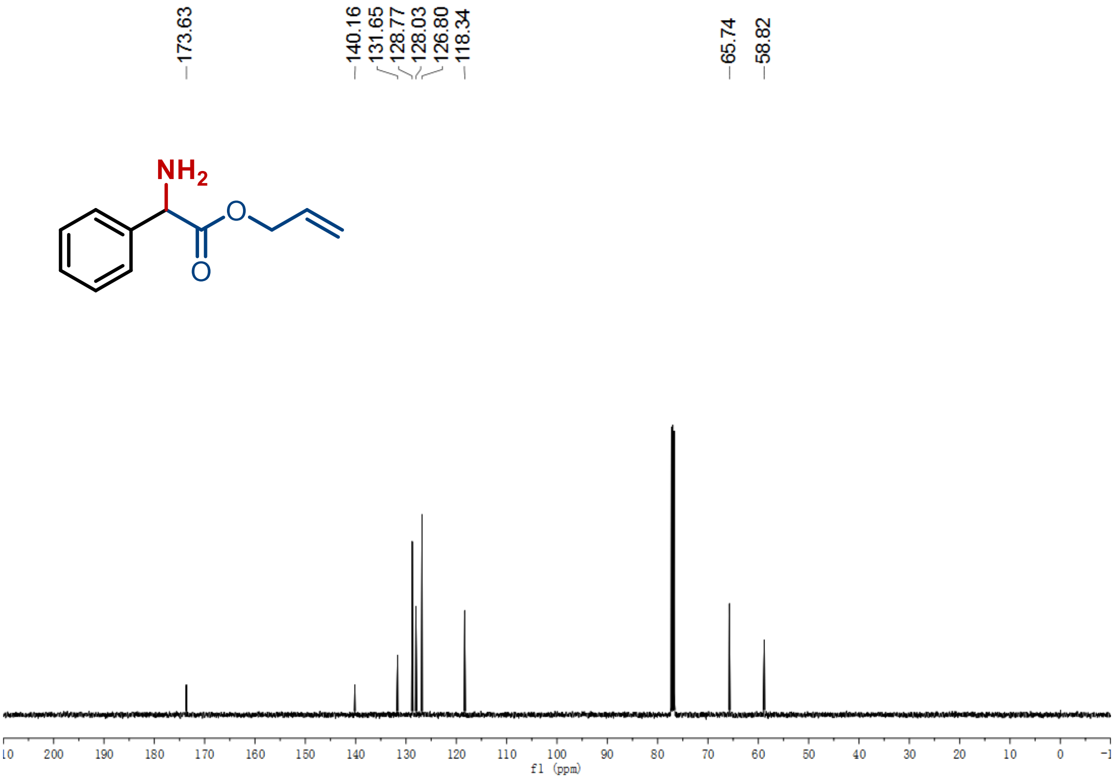

Supplementary Fig. 64 ^{13}C NMR (126 MHz, $CDCl_3$) spectrum of compound **28**.

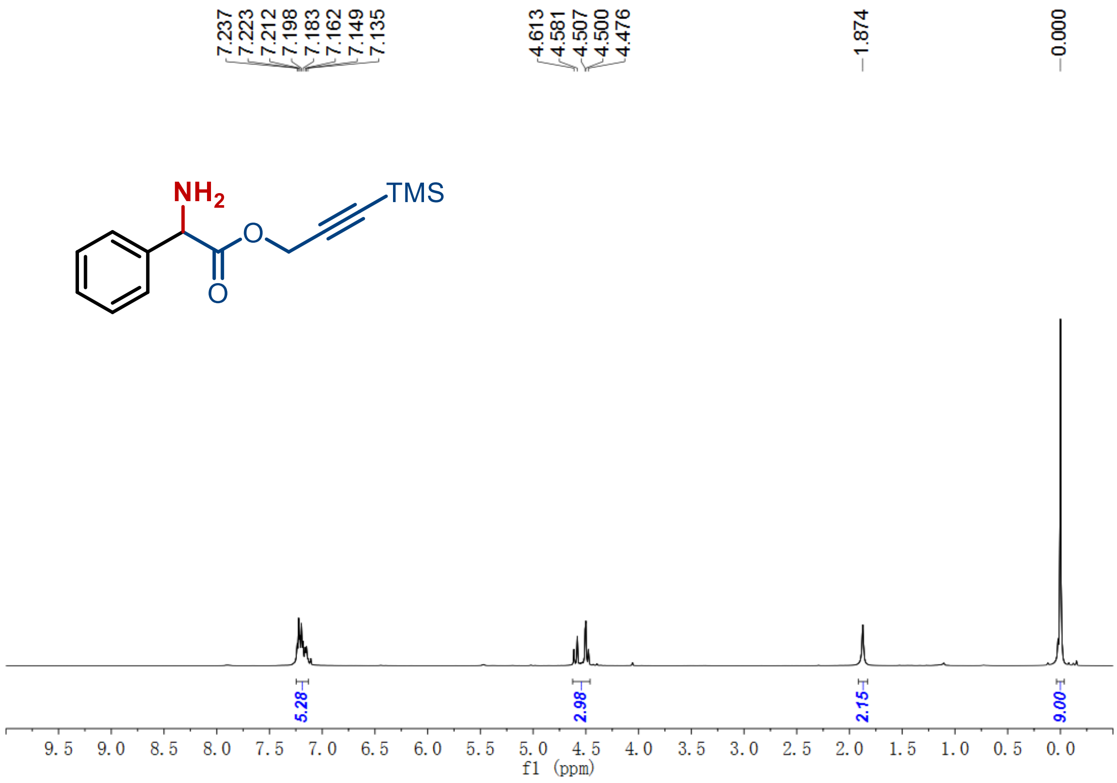

Supplementary Fig. 65 ^{1}H NMR (500 MHz, $CDCl_3$) spectrum of compound **29**.

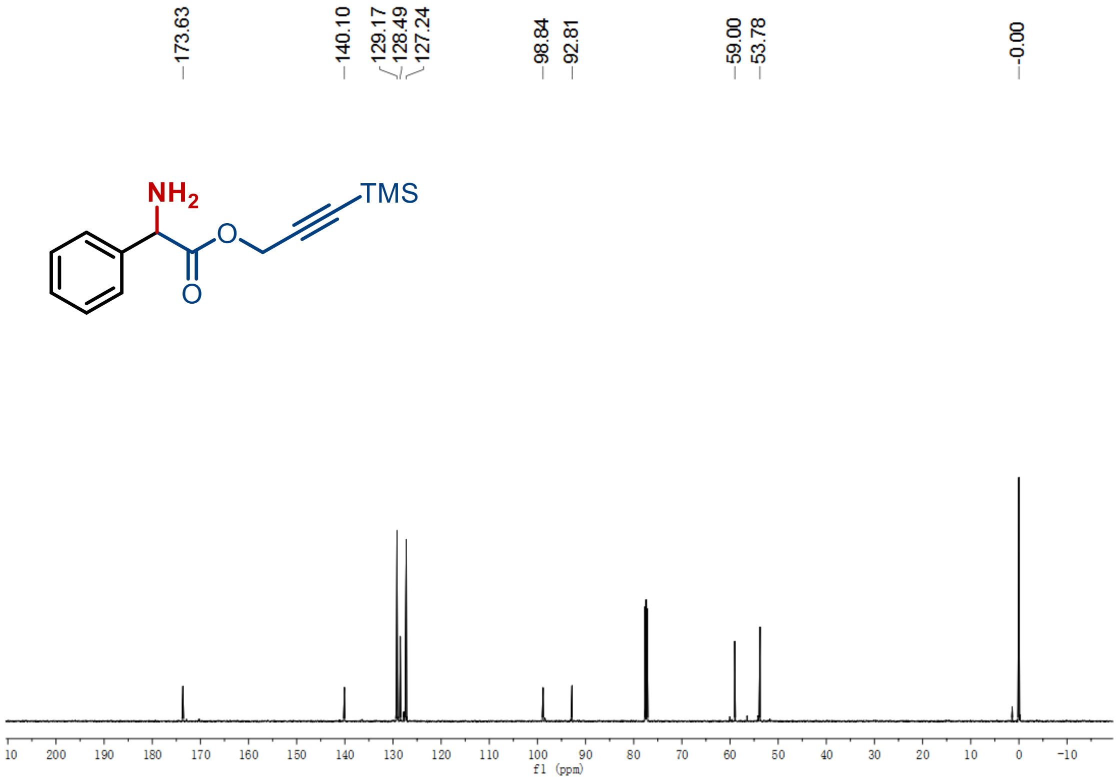

Supplementary Fig. 66 ^{13}C NMR (151 MHz, $CDCl_3$) spectrum of compound **29**.

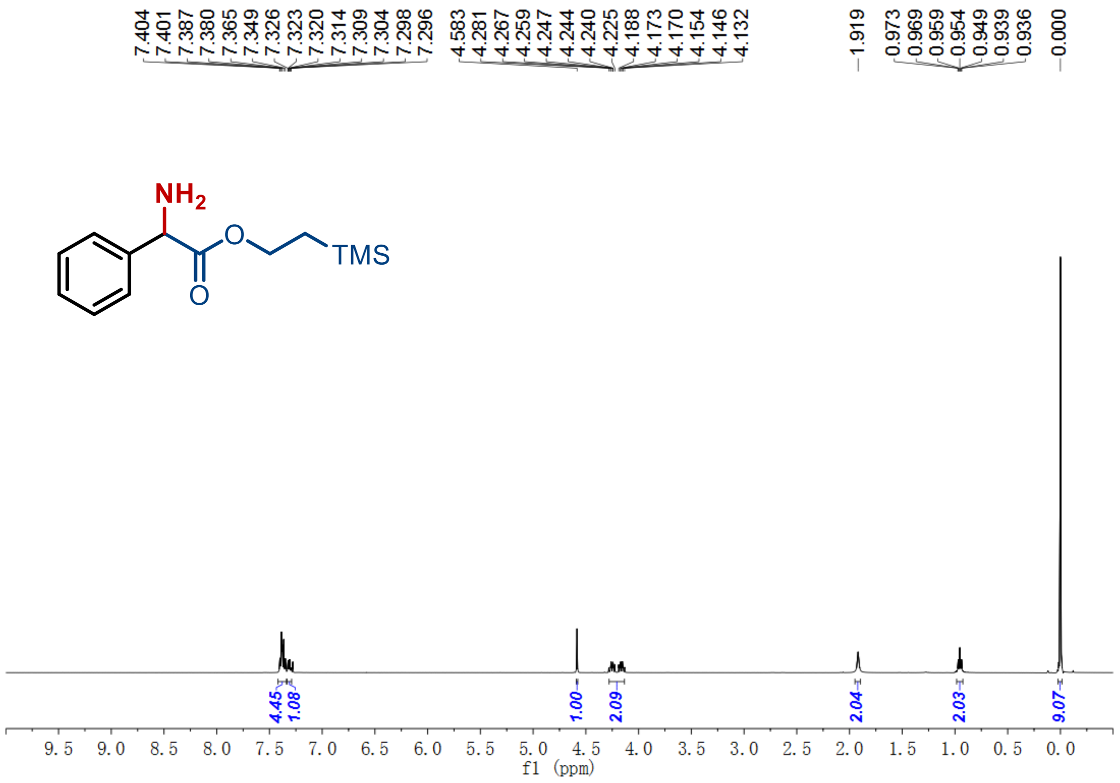

Supplementary Fig. 67 ^{1}H NMR (500 MHz, $CDCl_3$) spectrum of compound **30**.

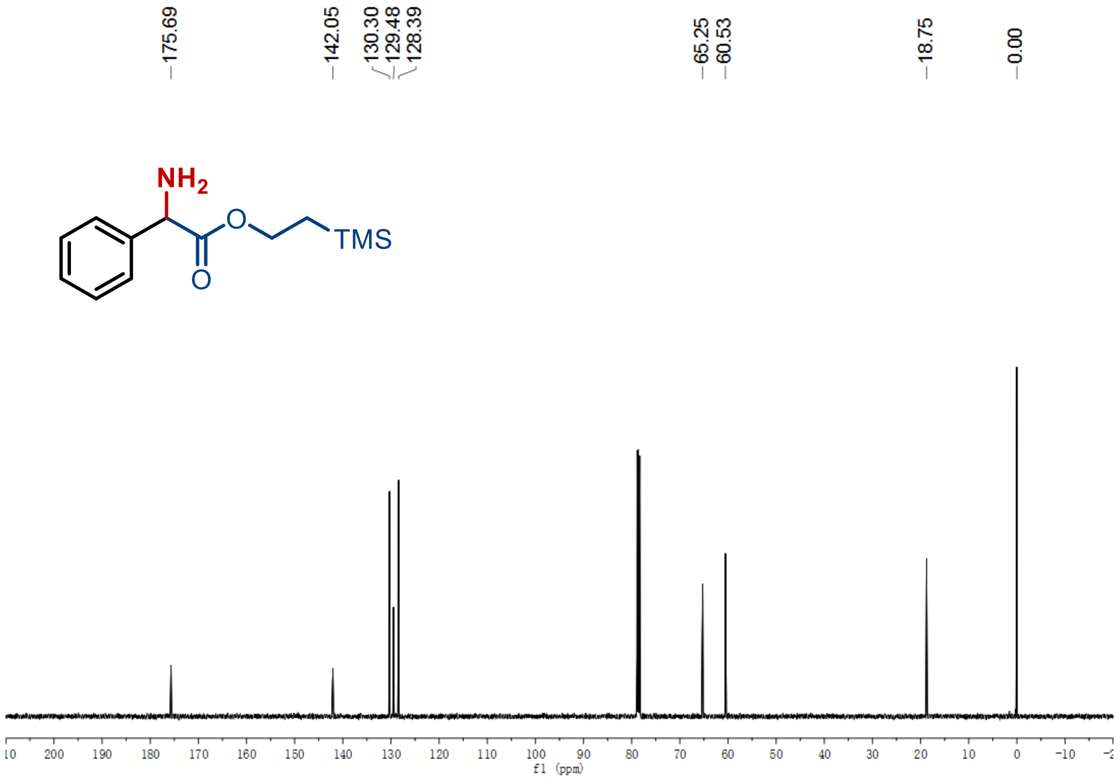

Supplementary Fig. 68 ^{13}C NMR (126 MHz, $CDCl_3$) spectrum of compound **30**.

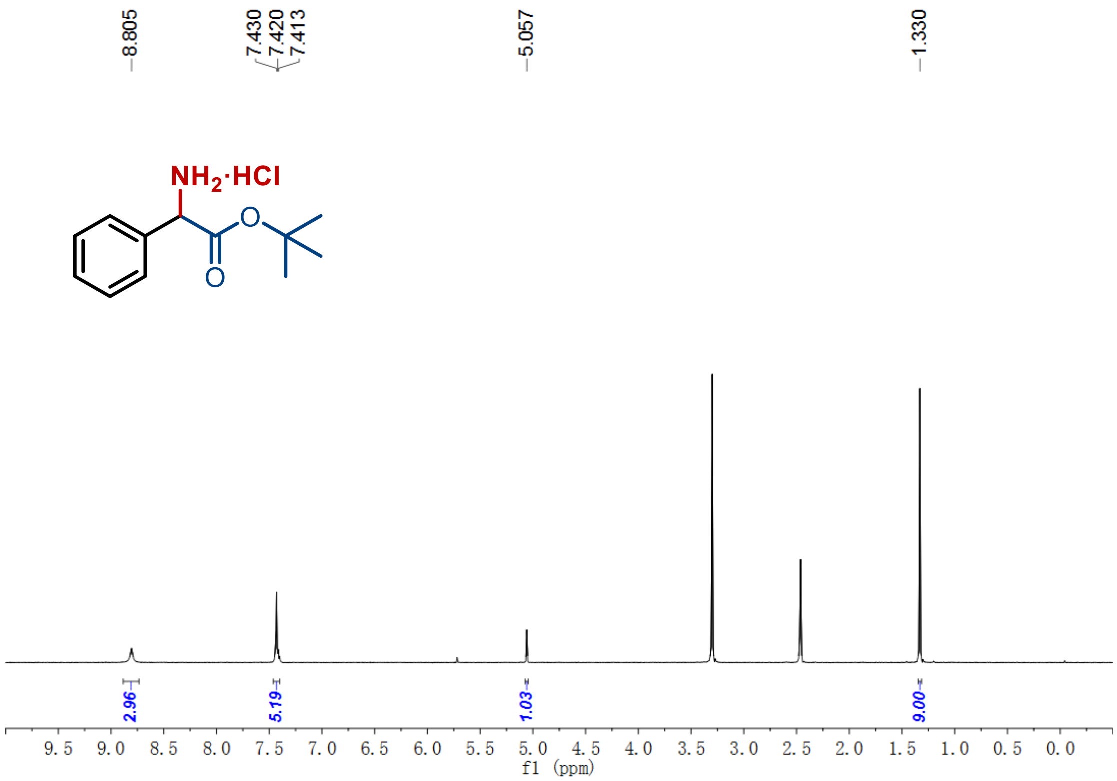

Supplementary Fig. 69 ^{1}H NMR (500 MHz, DMSO) spectrum of compound **31**.

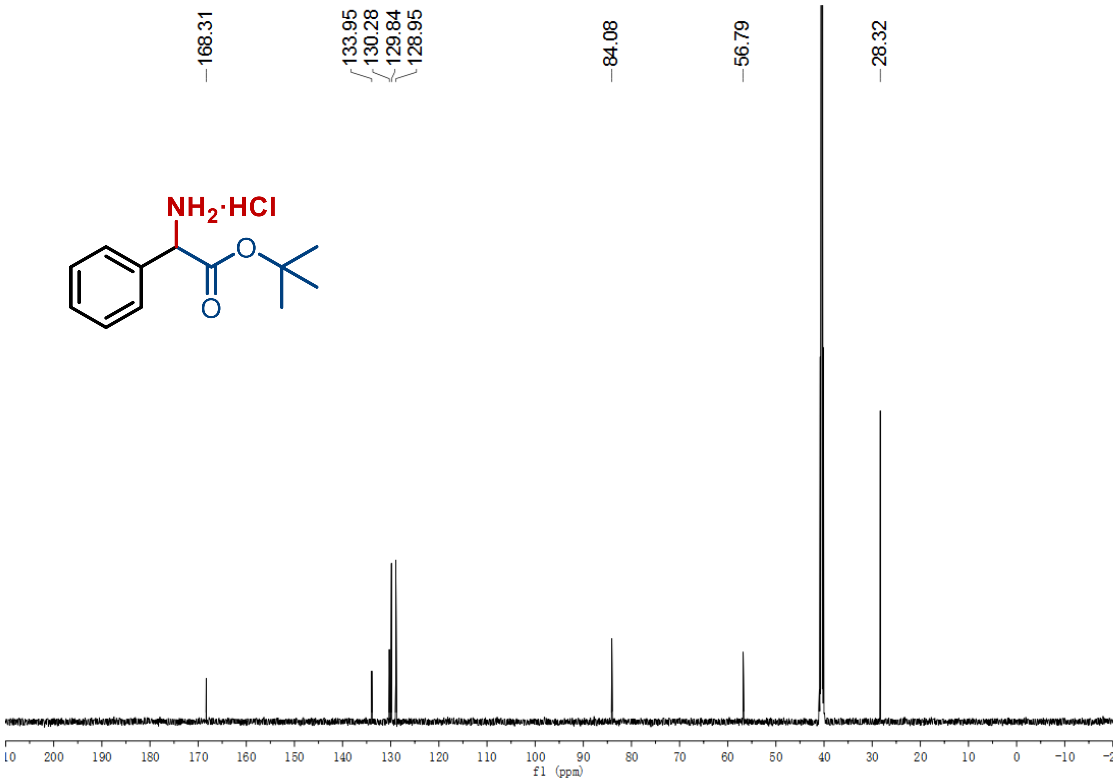

Supplementary Fig. 70 ^{13}C NMR (126 MHz, DMSO) spectrum of compound **31**.

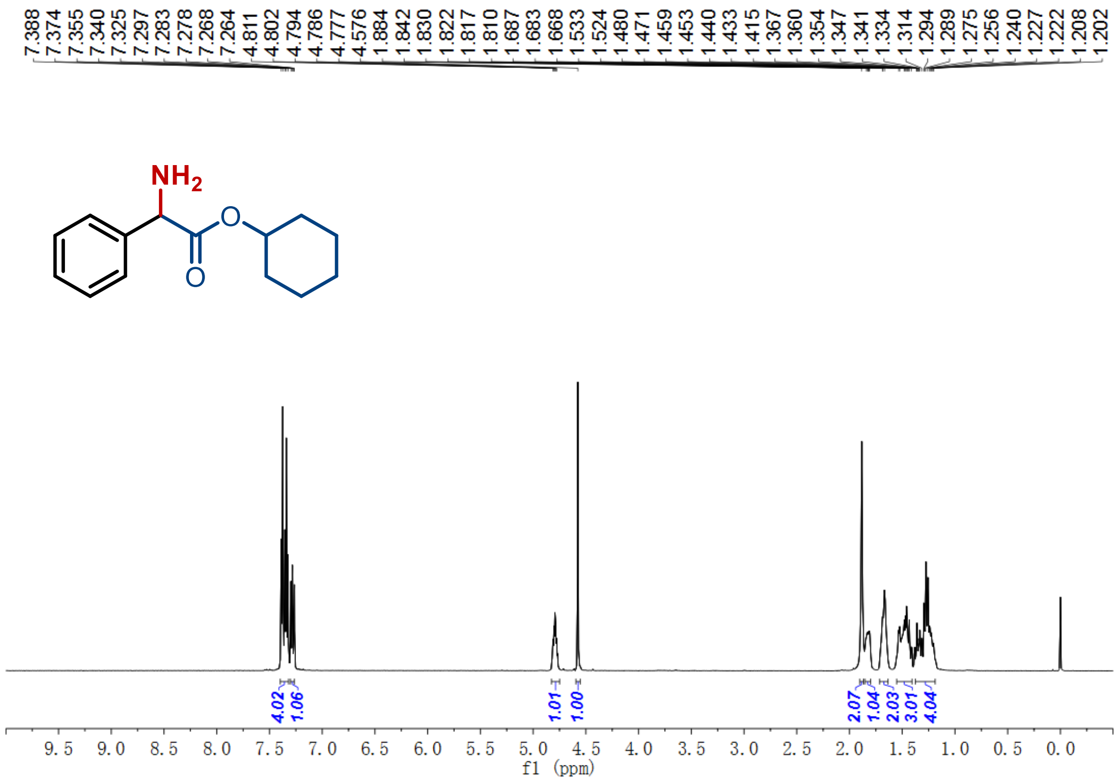

Supplementary Fig. 71 ^{1}H NMR (600 MHz, $CDCl_3$) spectrum of compound **32**.

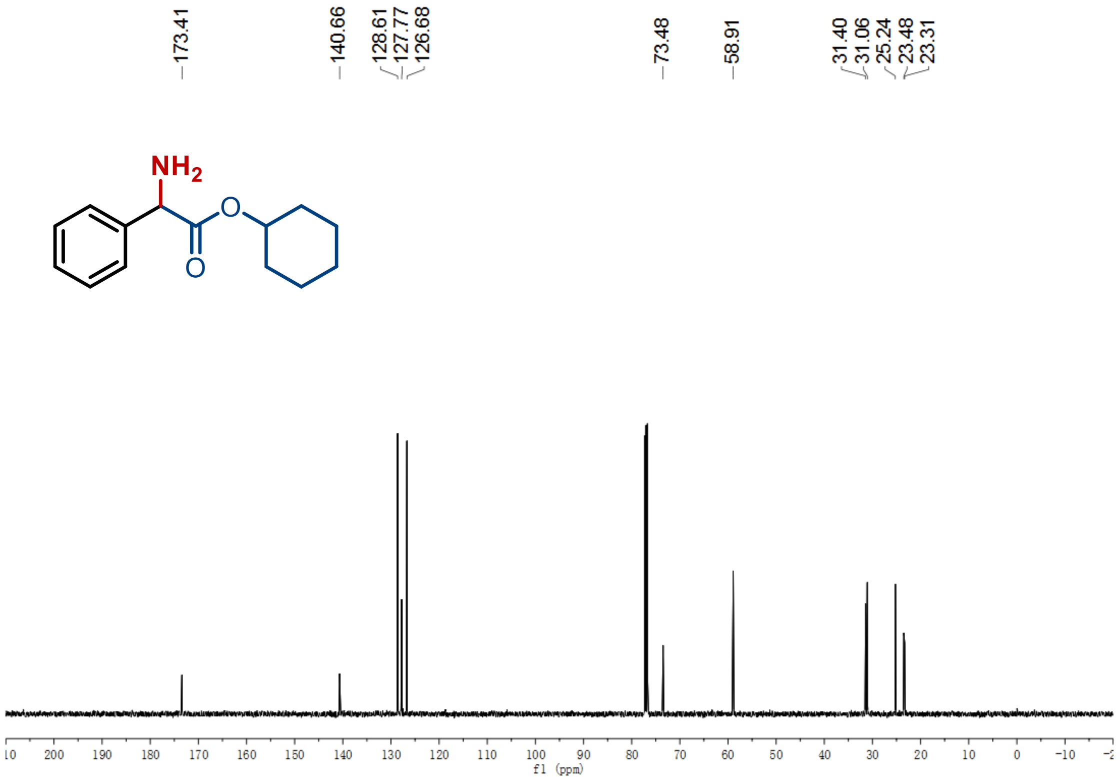

Supplementary Fig. 72 ^{13}C NMR (126 MHz, $CDCl_3$) spectrum of compound **32**.

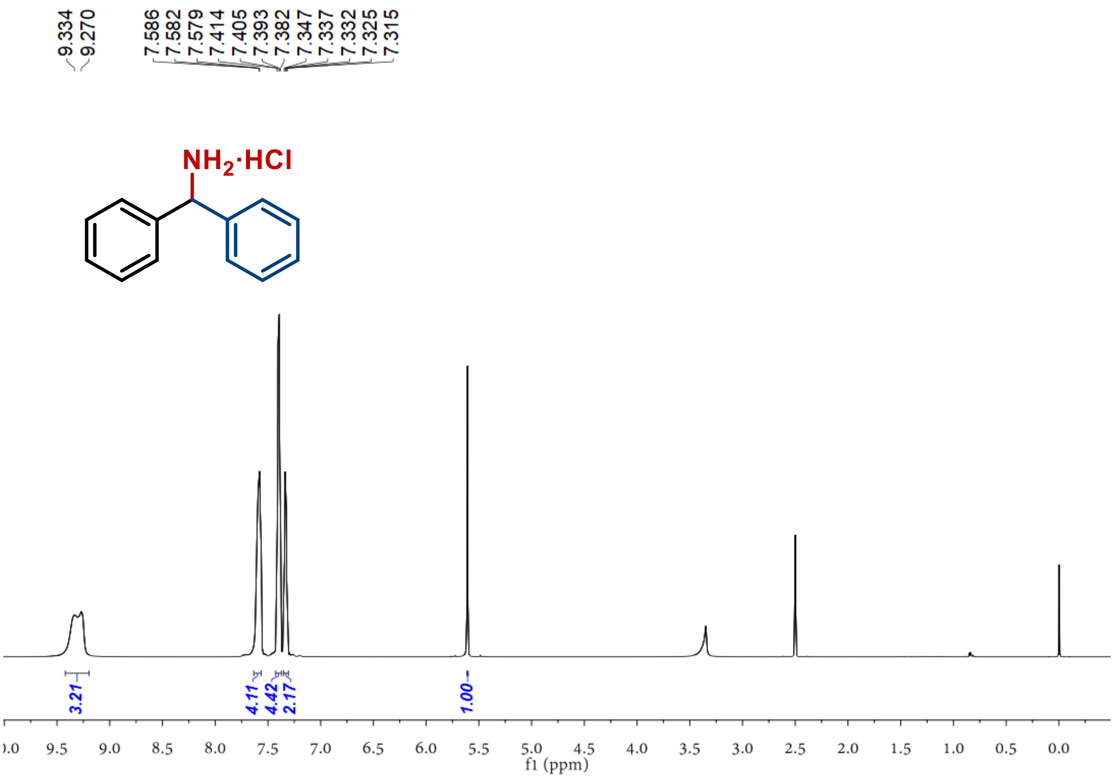

Supplementary Fig. 73 ^{1}H NMR (500 MHz, DMSO) spectrum of compound **33**.

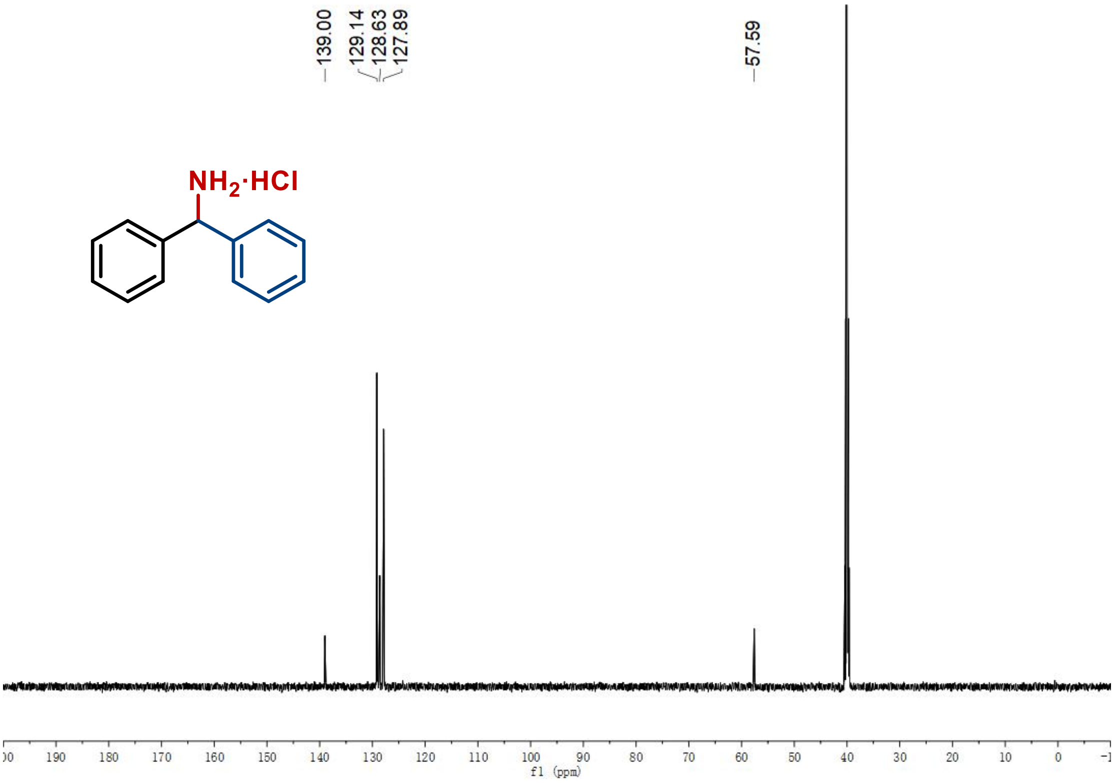

Supplementary Fig. 74 ^{13}C NMR (151 MHz, DMSO) spectrum of compound **33**.

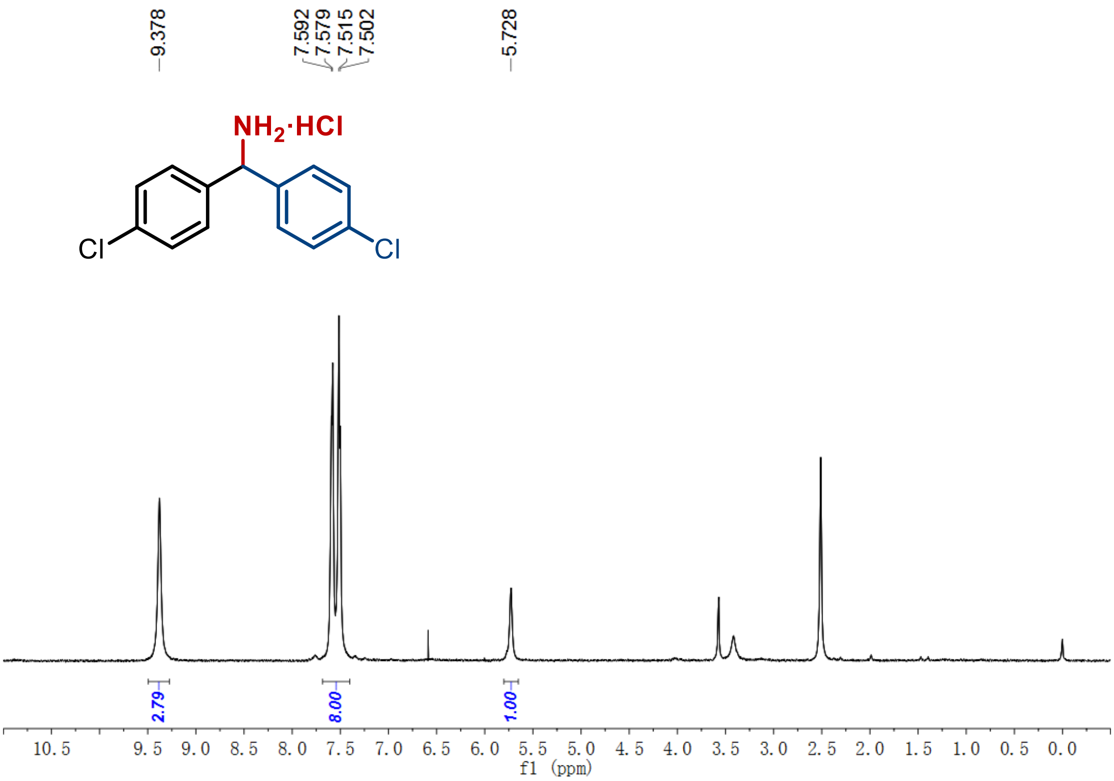

Supplementary Fig. 75 ^{1}H NMR (500 MHz, DMSO) spectrum of compound **34**.

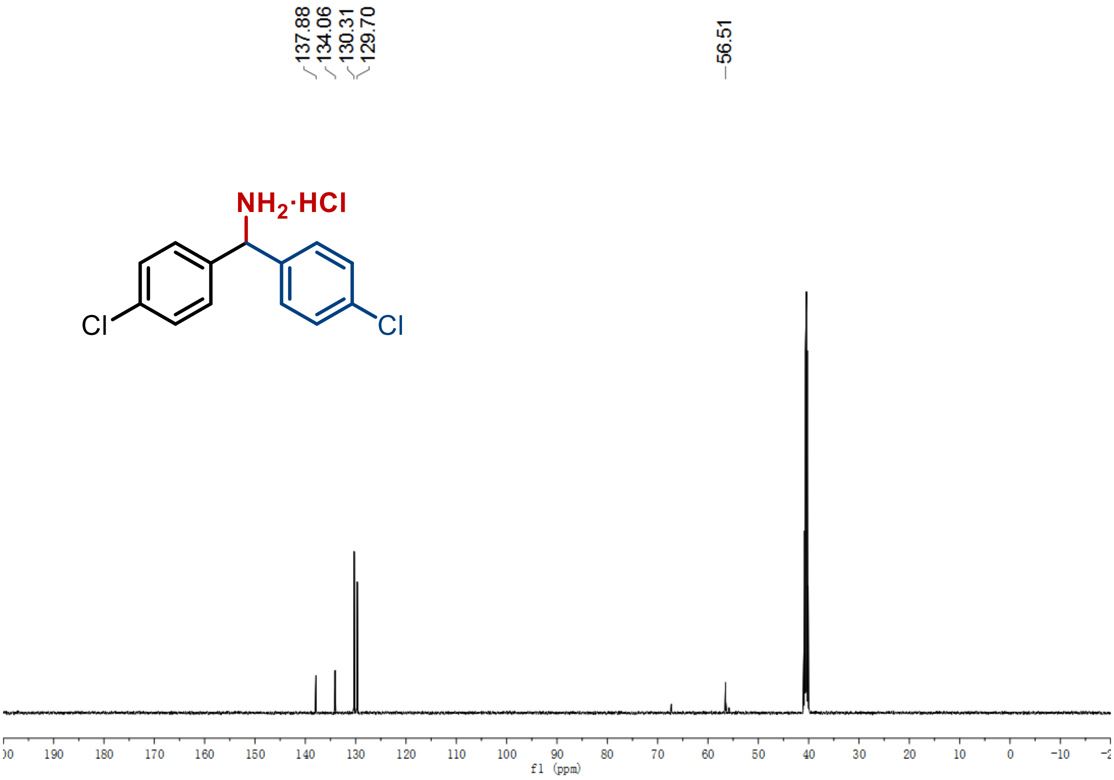

Supplementary Fig. 76 ^{13}C NMR (126 MHz, DMSO) spectrum of compound **34**.

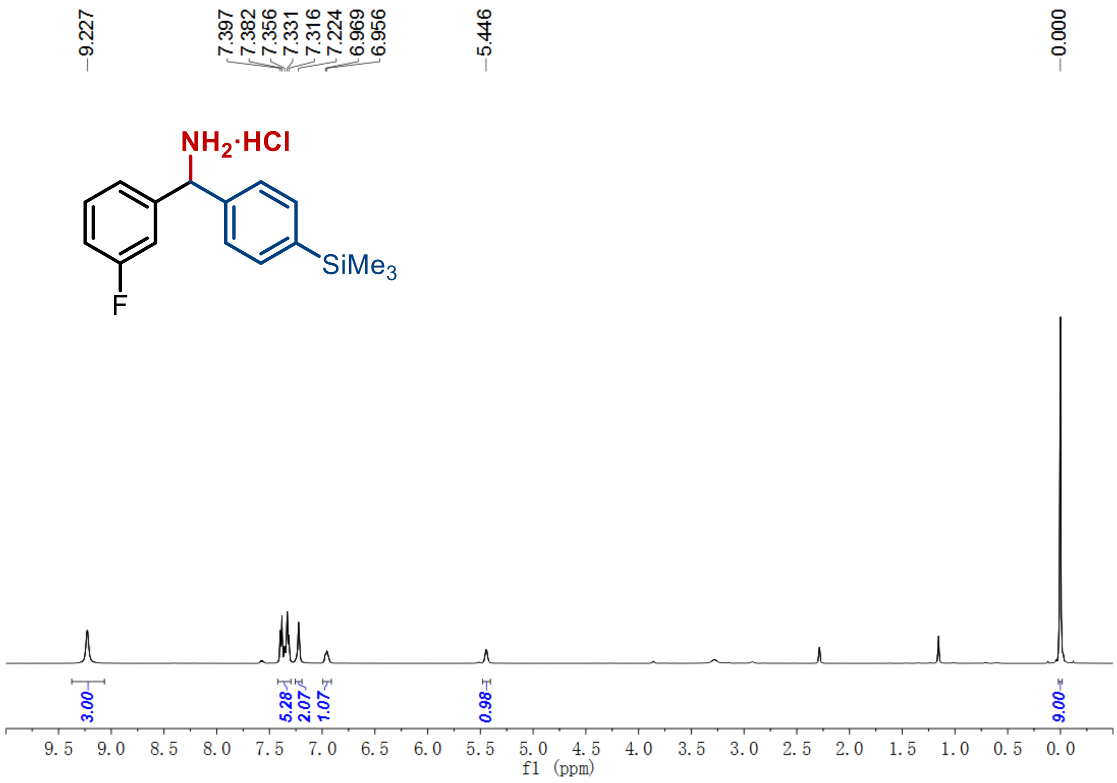

Supplementary Fig. 77 ^{1}H NMR (500 MHz, DMSO) spectrum of compound **36**.

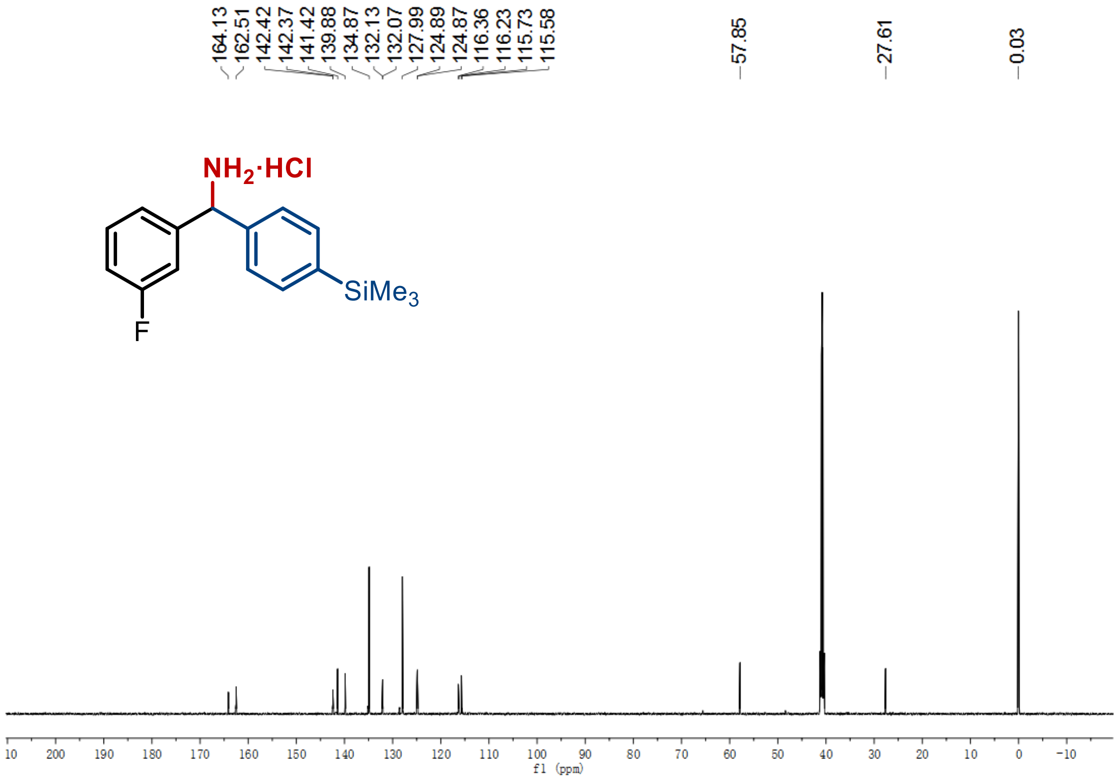

Supplementary Fig. 78 ^{13}C NMR (151 MHz, DMSO) spectrum of compound **36**.

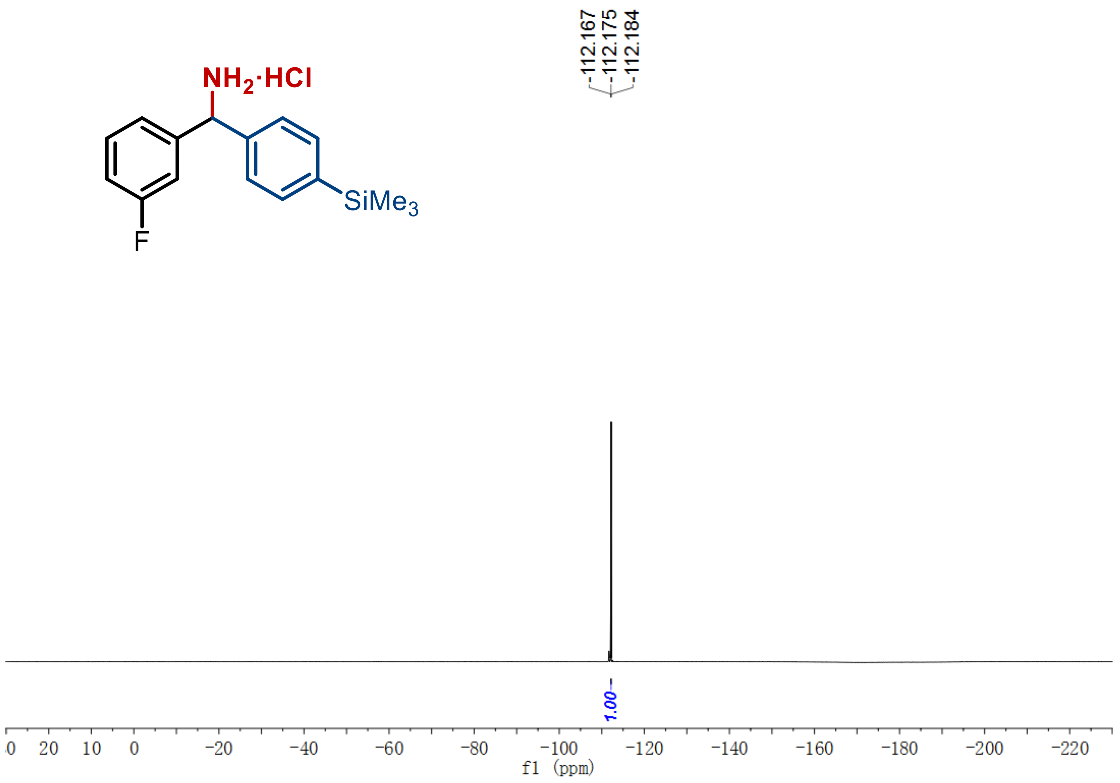

Supplementary Fig. 79 ^{19}F NMR (565 MHz, DMSO) spectrum of compound **36**.

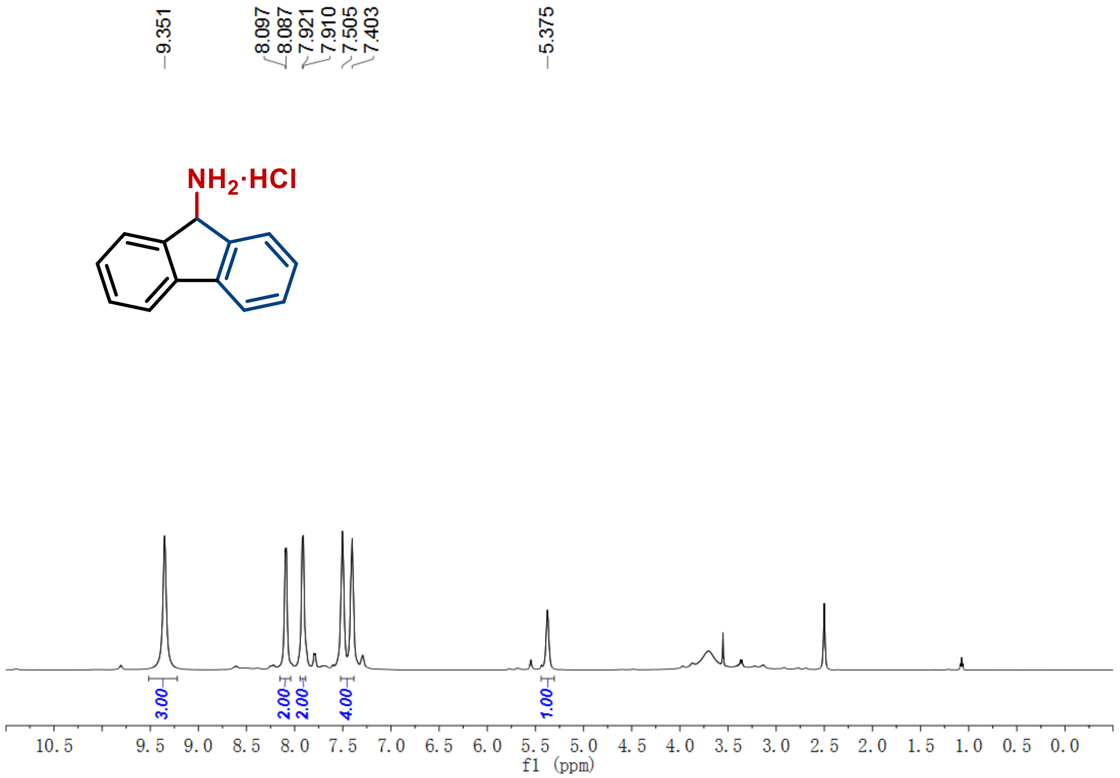

Supplementary Fig. 80 ^{1}H NMR (500 MHz, DMSO) spectrum of compound **37**.

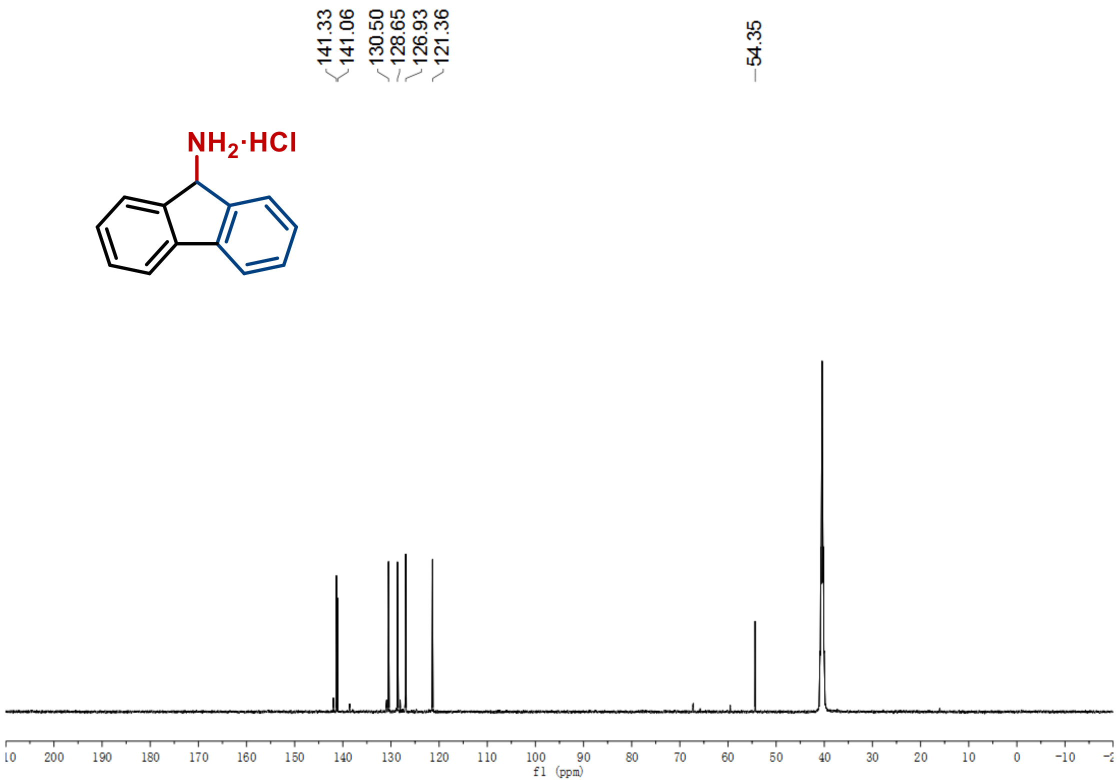

Supplementary Fig. 81 ^{13}C NMR (151 MHz, DMSO) spectrum of compound **37**.

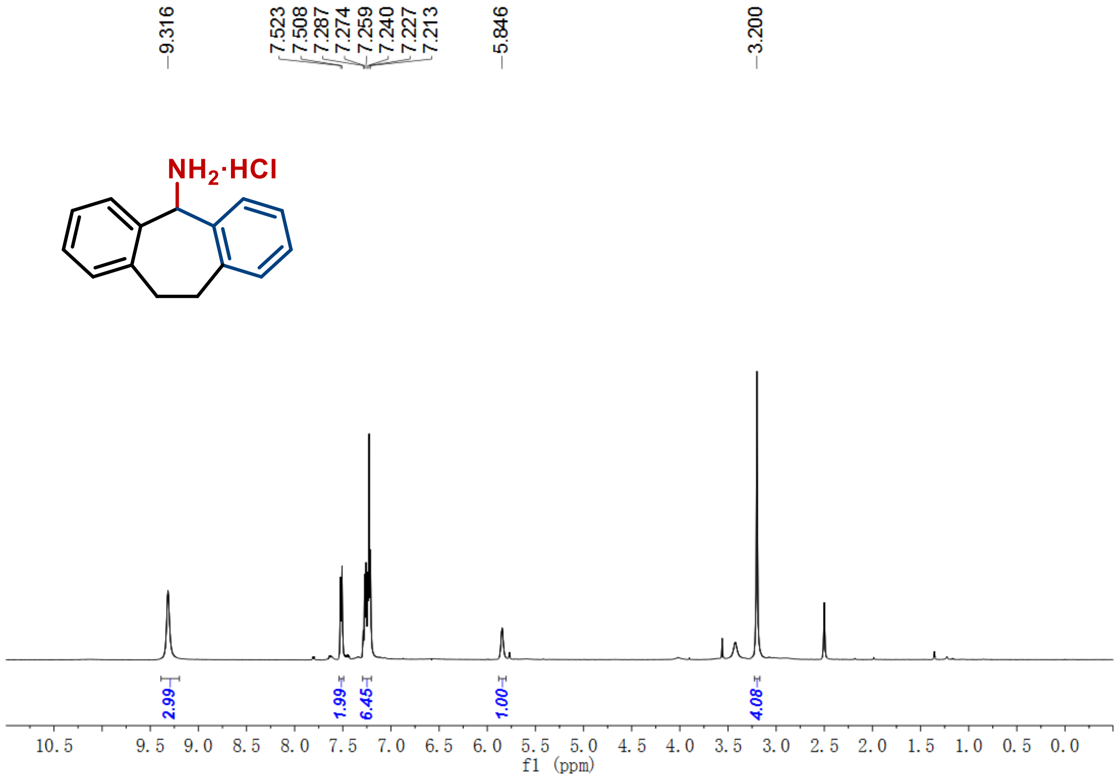

Supplementary Fig. 82 ^{1}H NMR (500 MHz, DMSO) spectrum of compound **38**.

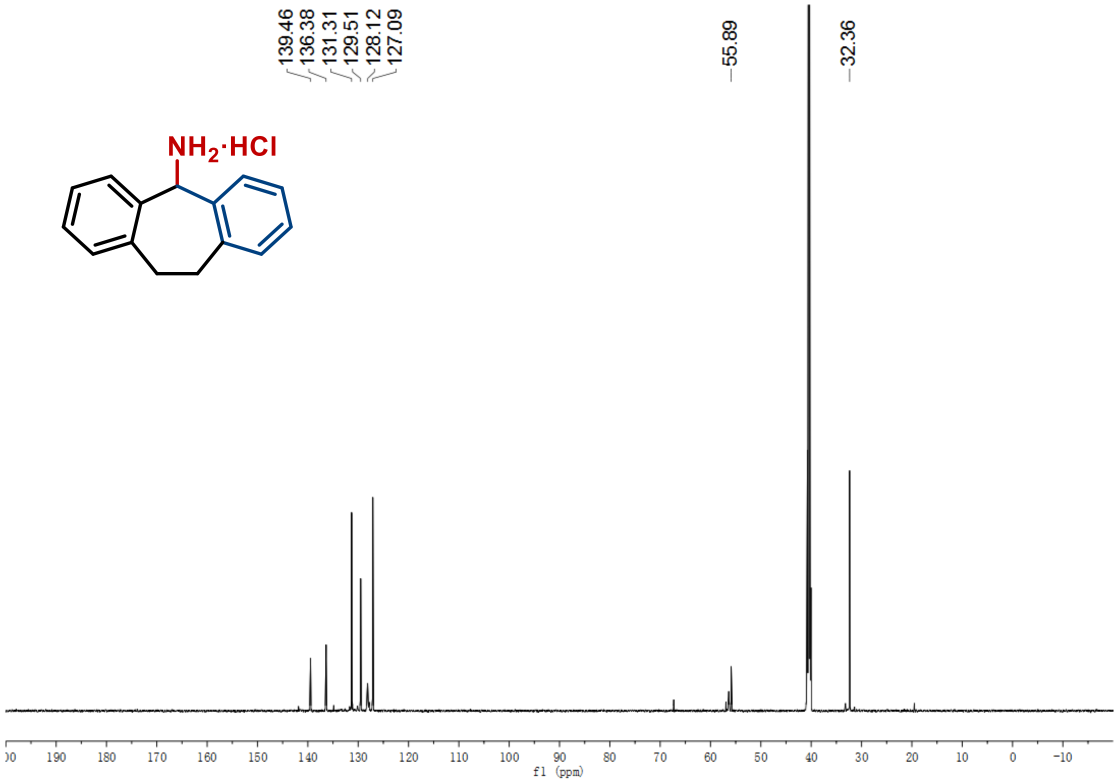

Supplementary Fig. 83 ^{13}C NMR (151 MHz, DMSO) spectrum of compound **38**.

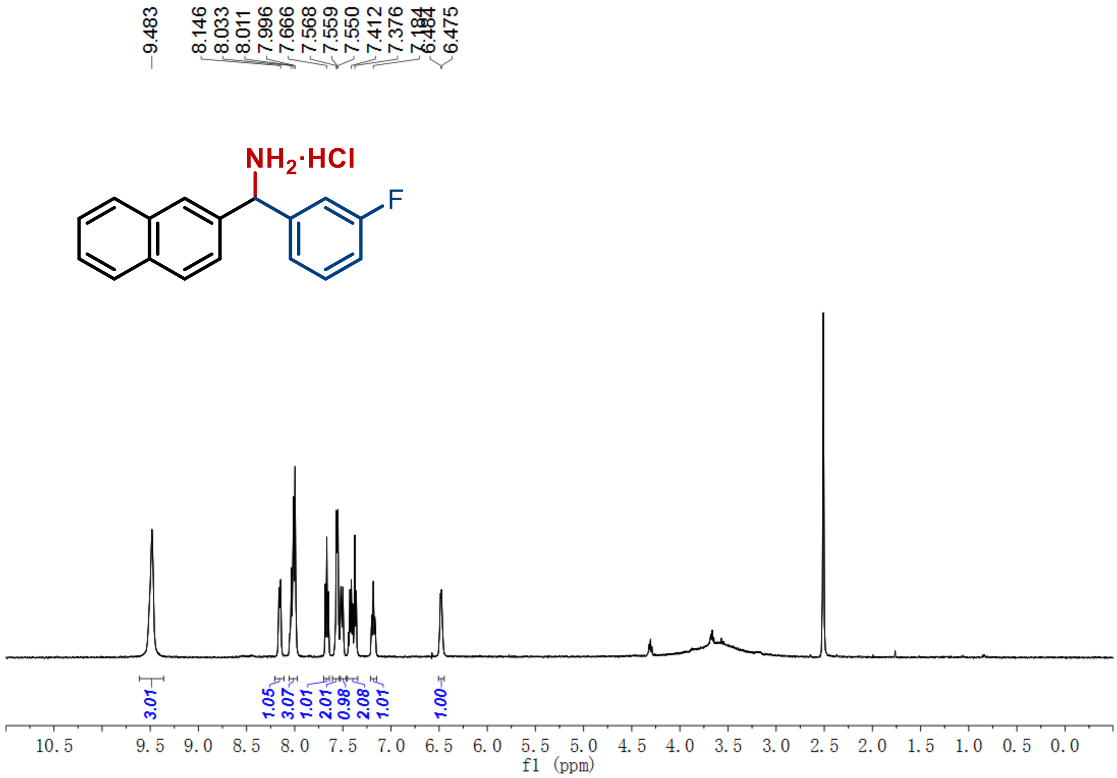

Supplementary Fig. 84 ^{1}H NMR (500 MHz, DMSO) spectrum of compound **39**.

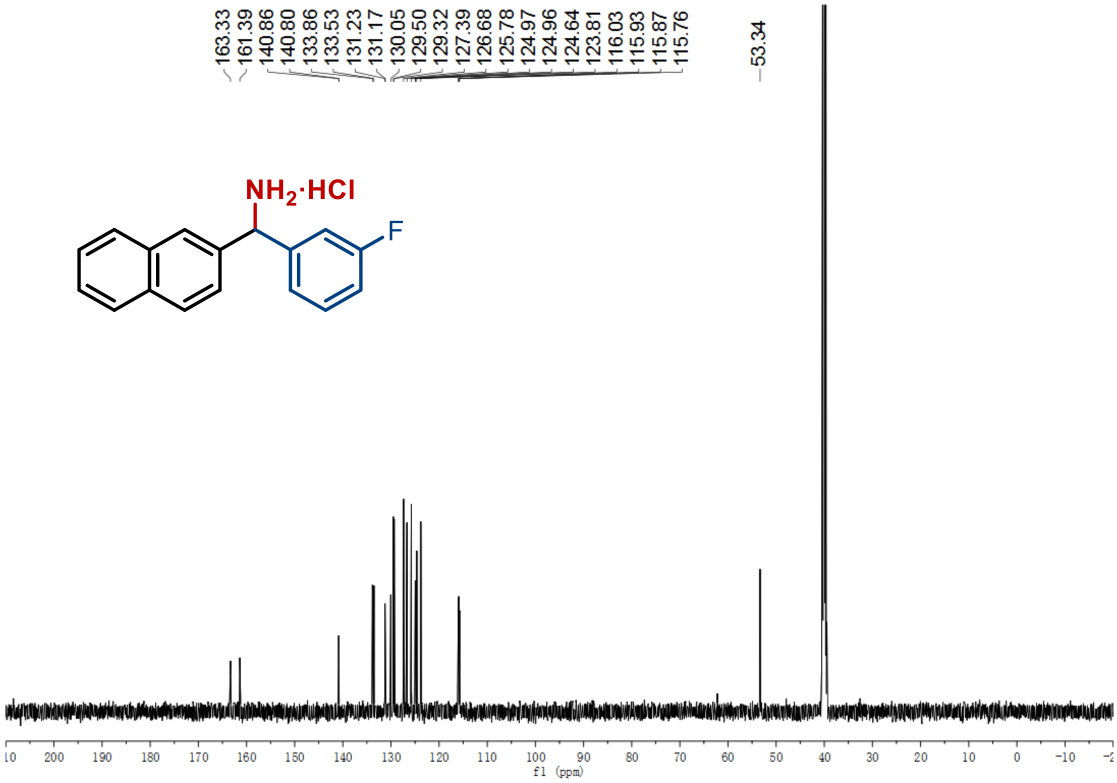

Supplementary Fig. 85 ^{13}C NMR (126 MHz, DMSO) spectrum of compound **39**.

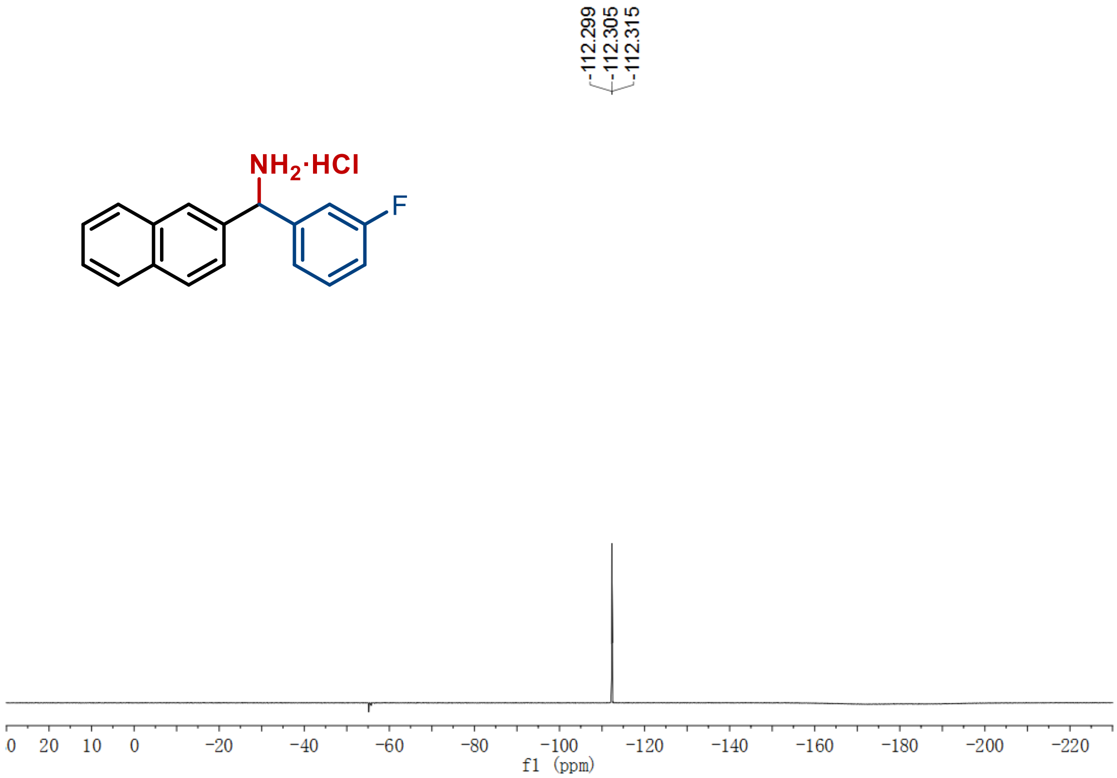

Supplementary Fig. 86 ^{19}F NMR (565 MHz, DMSO) spectrum of compound **39**.

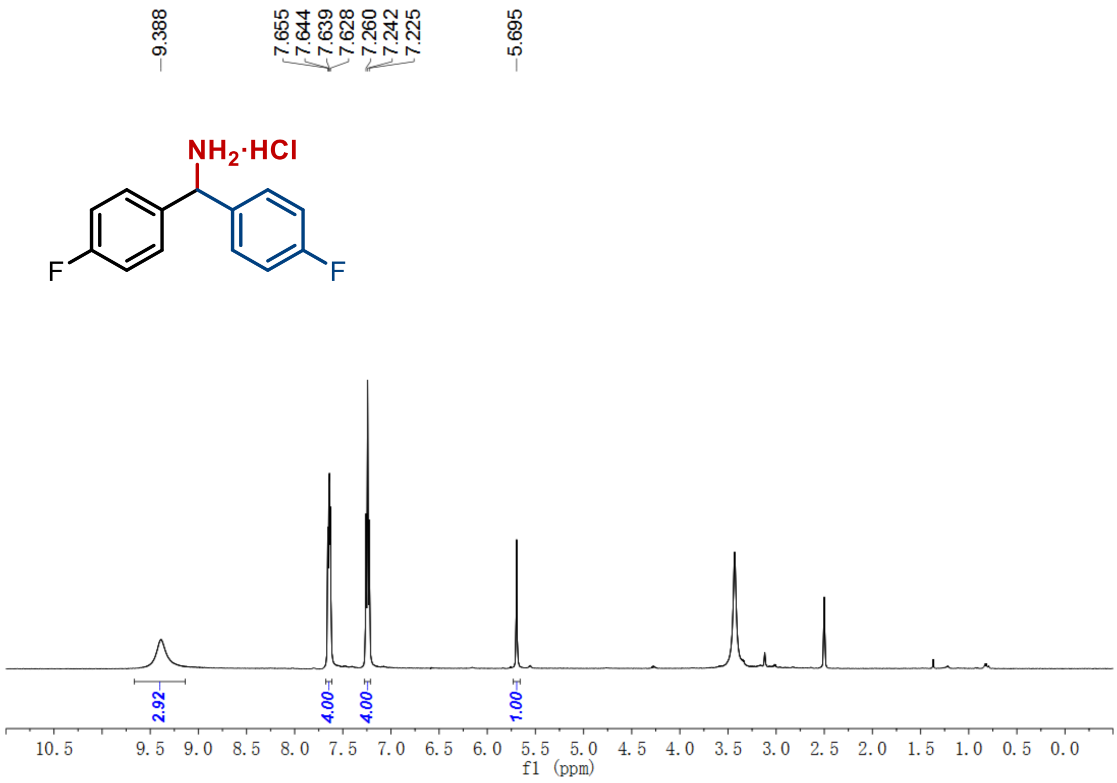

Supplementary Fig. 87 ^{1}H NMR (500 MHz, DMSO) spectrum of compound **40**.

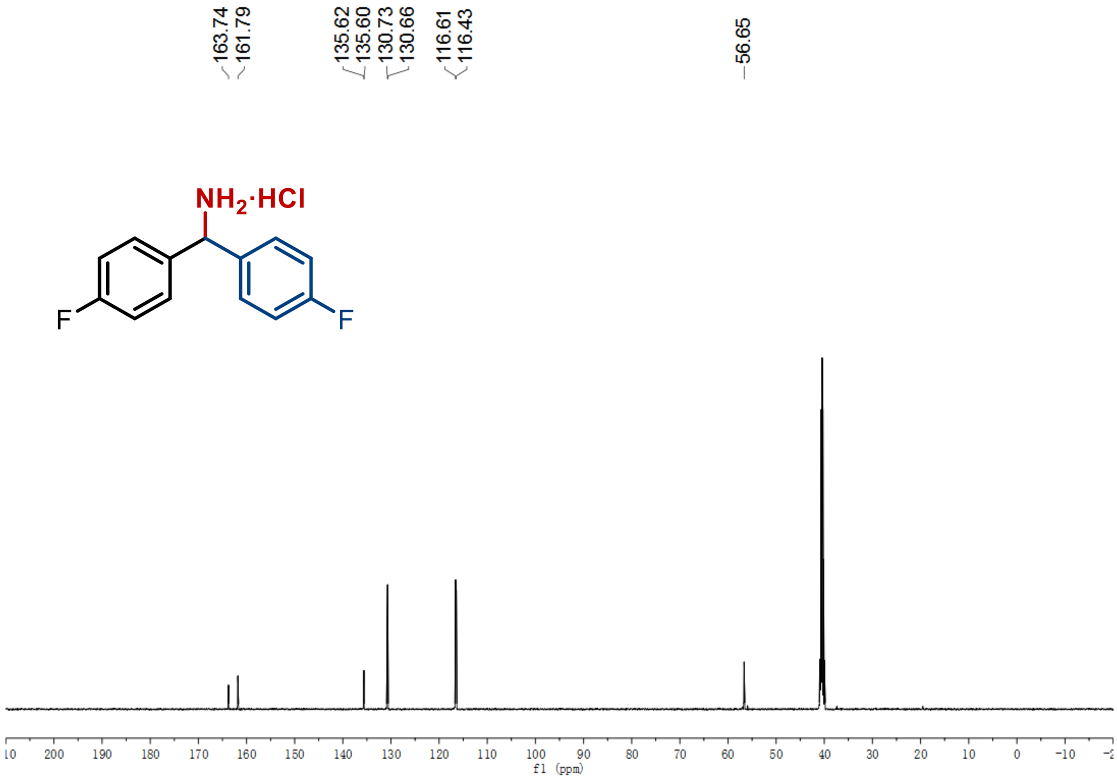

Supplementary Fig. 88 ^{13}C NMR (126 MHz, DMSO) spectrum of compound **40**.

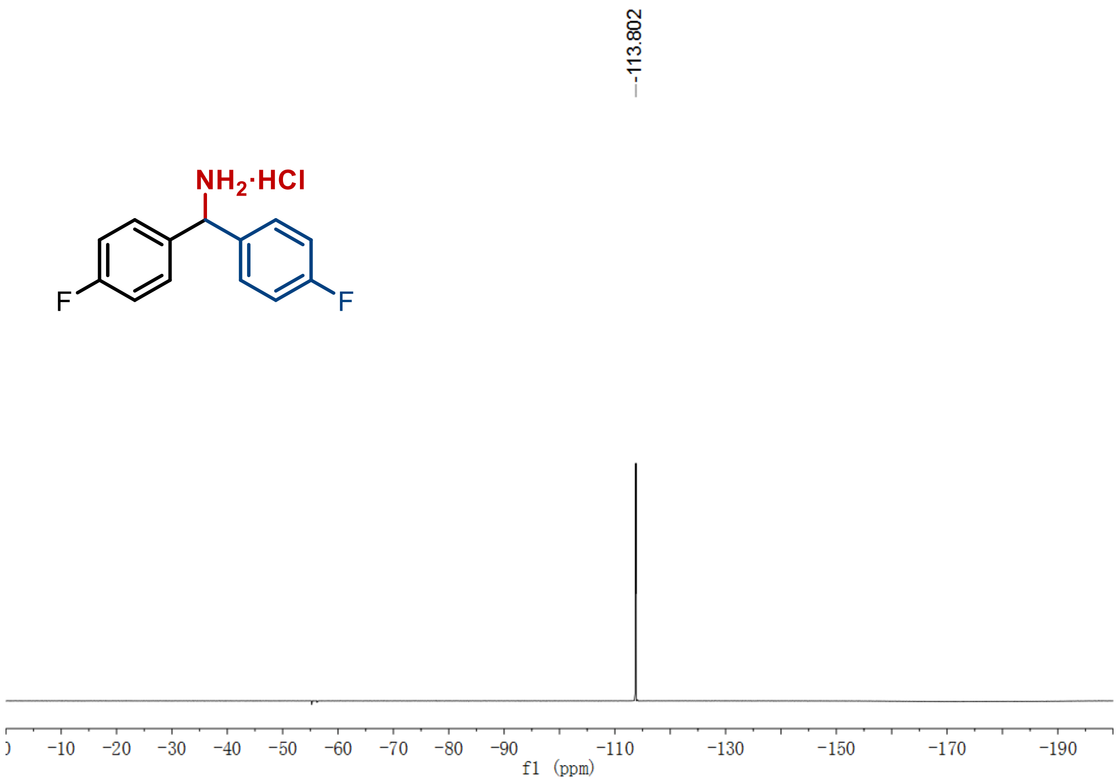

Supplementary Fig. 89 ^{19}F NMR (565 MHz, DMSO) spectrum of compound **40**.

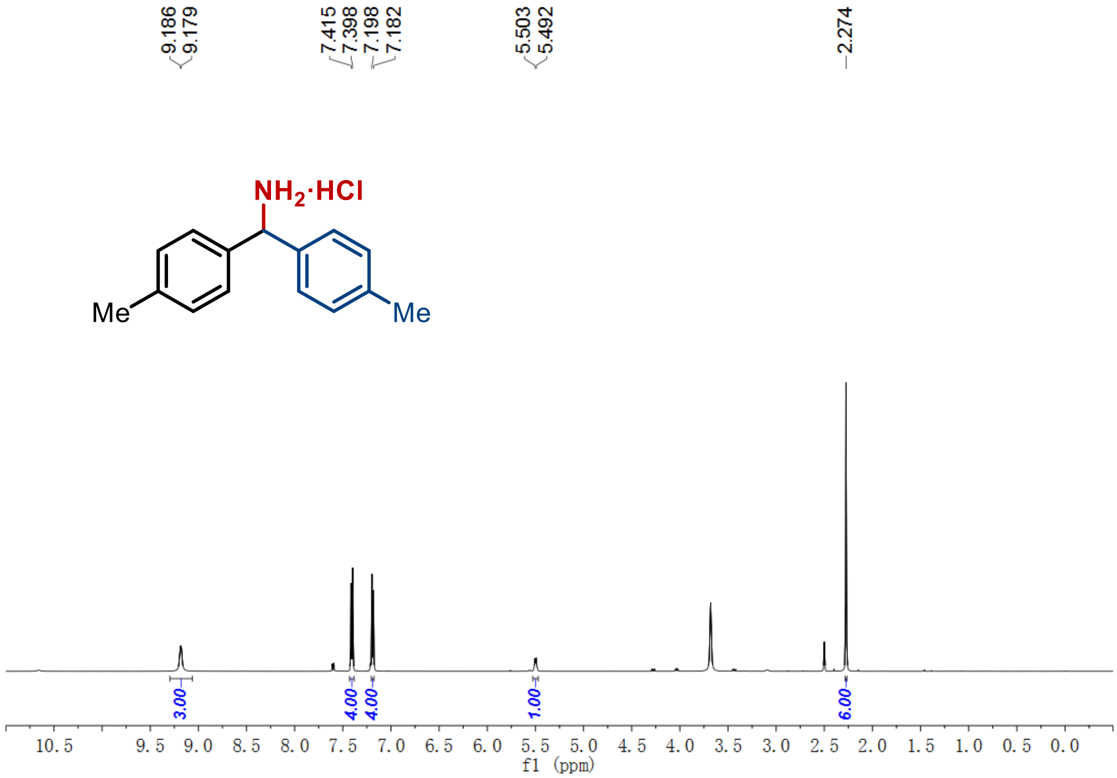

Supplementary Fig. 90 ^{1}H NMR (500 MHz, DMSO) spectrum of compound **41**.

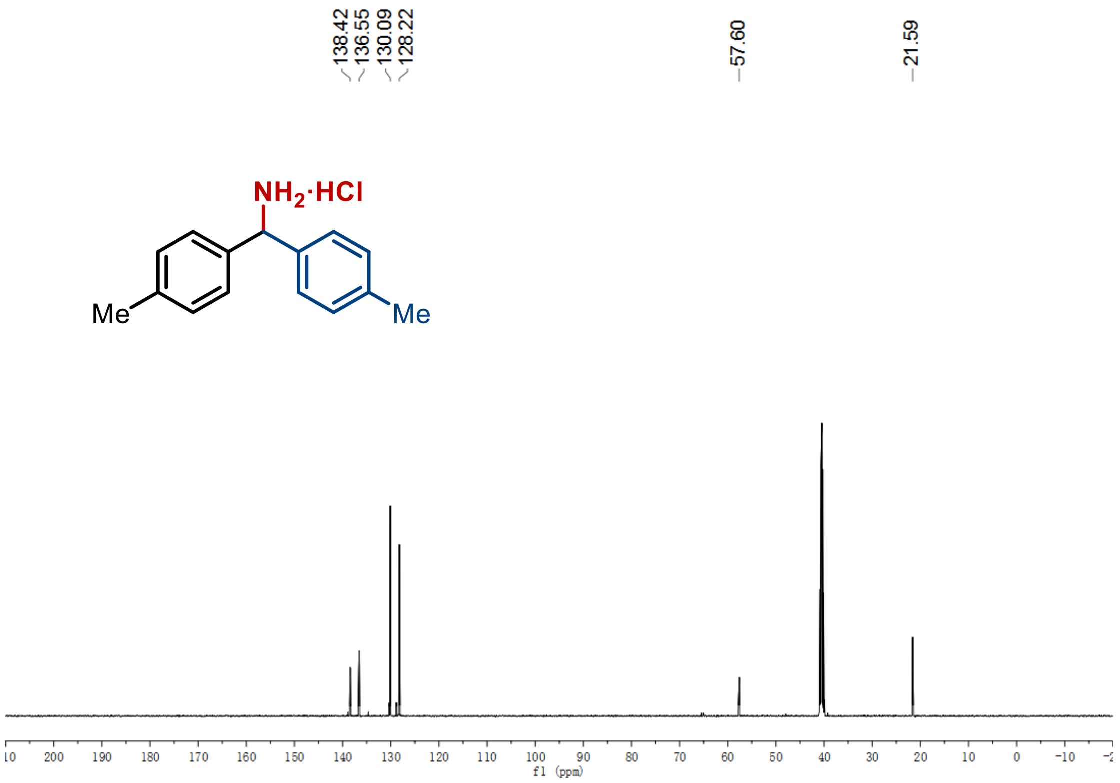

Supplementary Fig. 91 ^{13}C NMR (126 MHz, DMSO) spectrum of compound **41**.

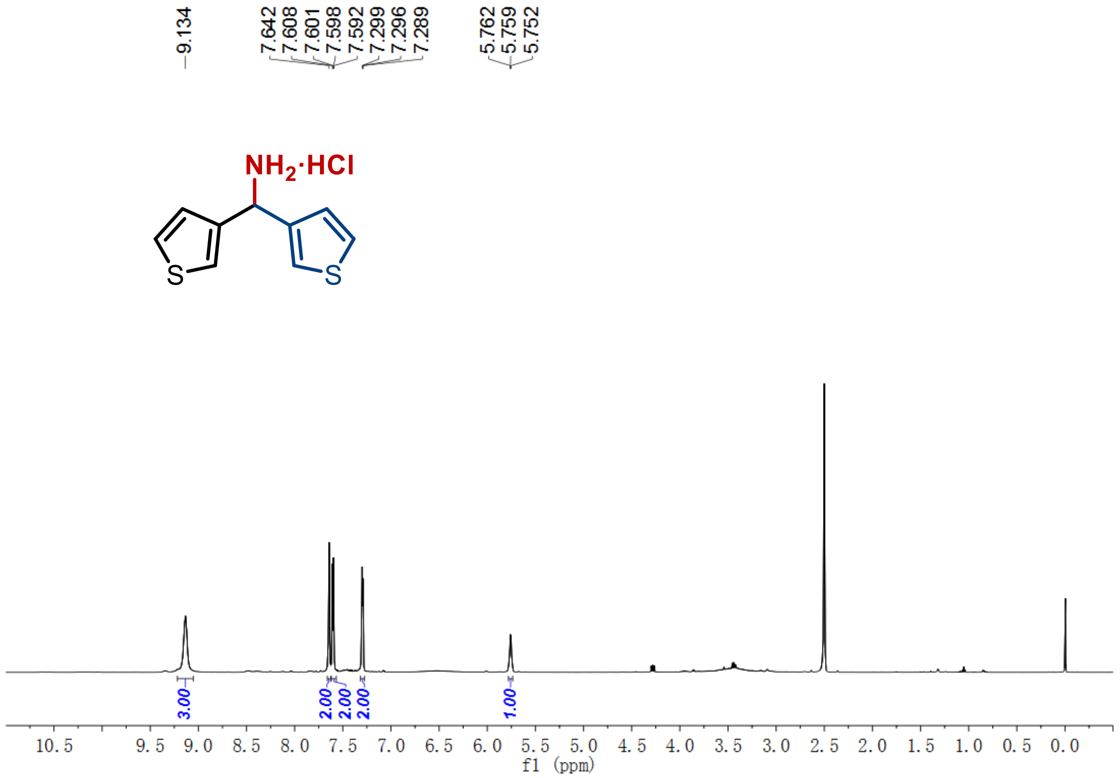

Supplementary Fig. 92 ^{1}H NMR (500 MHz, DMSO) spectrum of compound **42**.

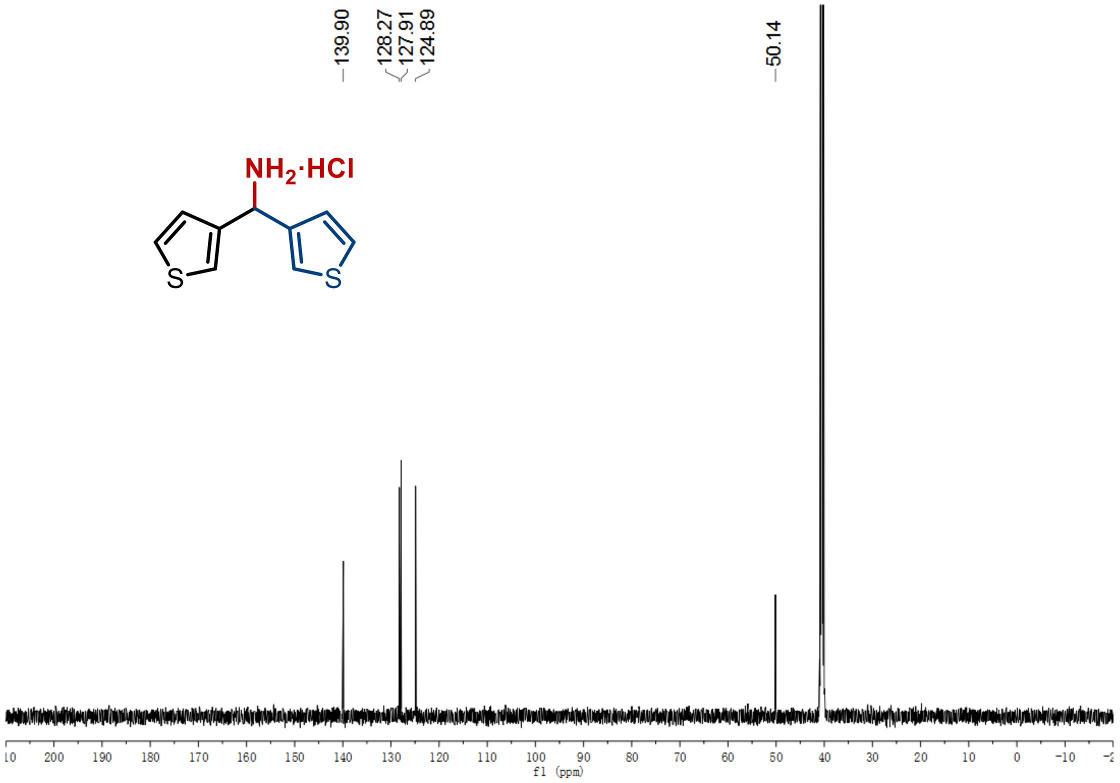

Supplementary Fig. 93 ^{13}C NMR (126 MHz, DMSO) spectrum of compound **42**.

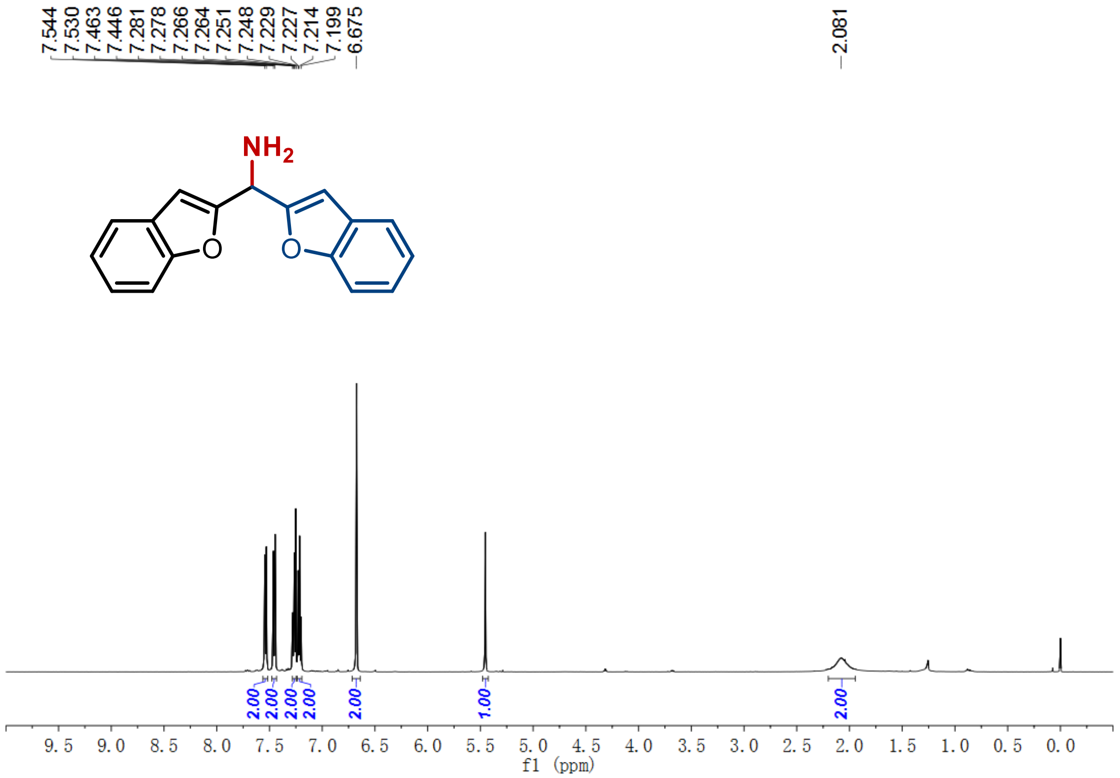

Supplementary Fig. 94 ^{1}H NMR (500 MHz, $CDCl_3$) spectrum of compound **43**.

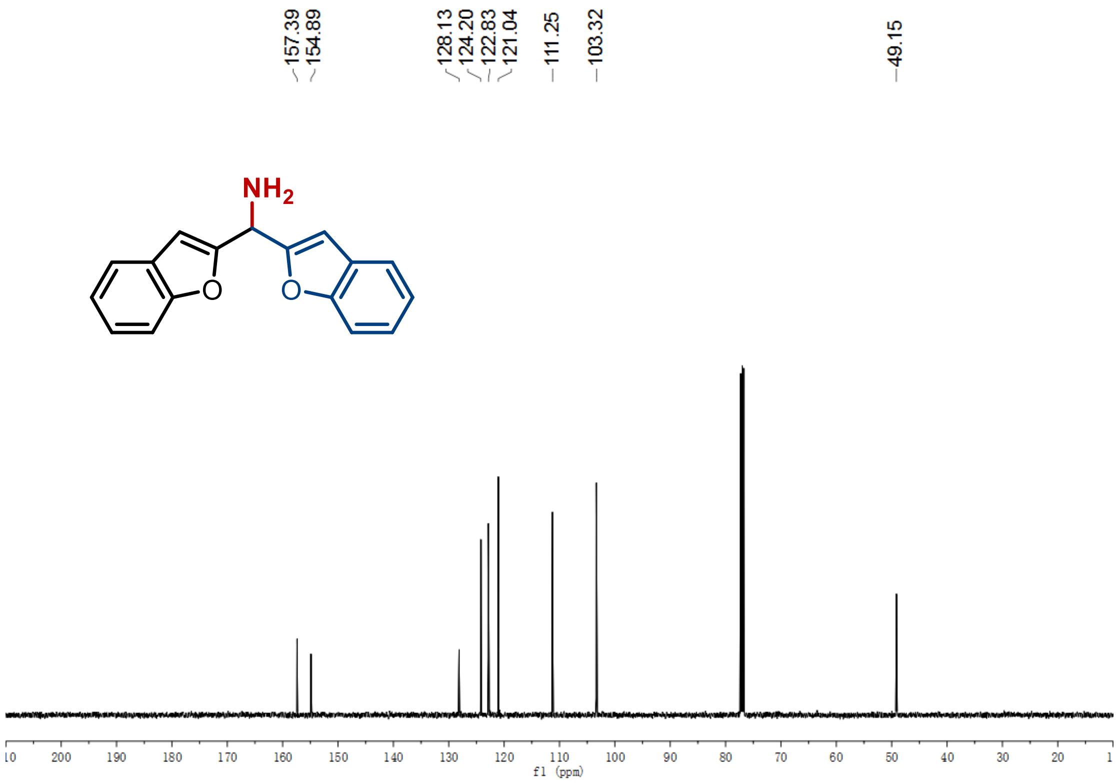

Supplementary Fig. 95 ^{13}C NMR (126 MHz, $CDCl_3$) spectrum of compound **43**.

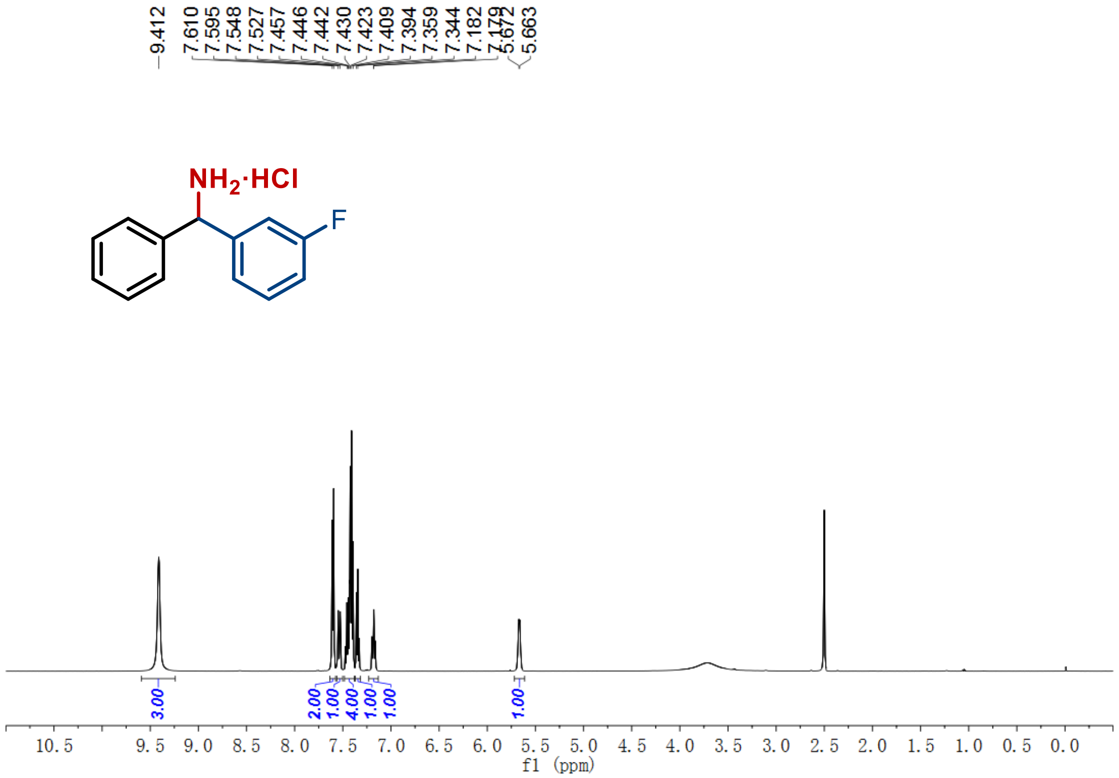

Supplementary Fig. 96 ^{1}H NMR (500 MHz, DMSO) spectrum of compound **44**.

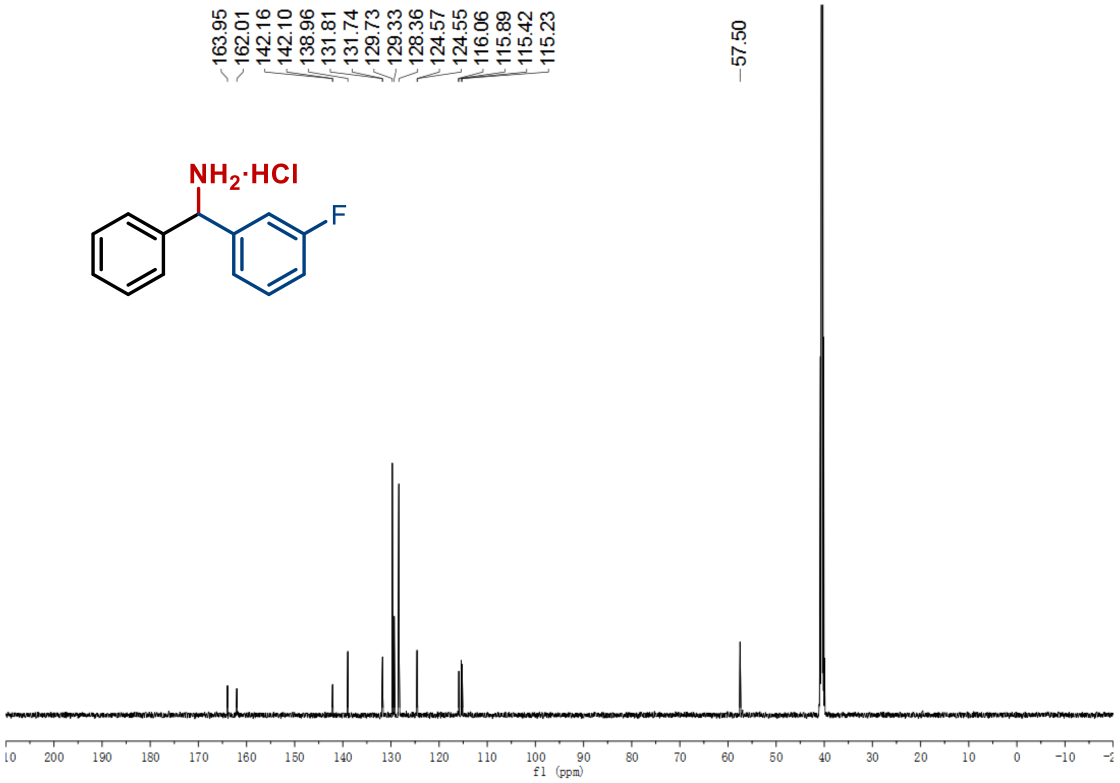

Supplementary Fig. 97 ^{13}C NMR (126 MHz, DMSO) spectrum of compound **44**.

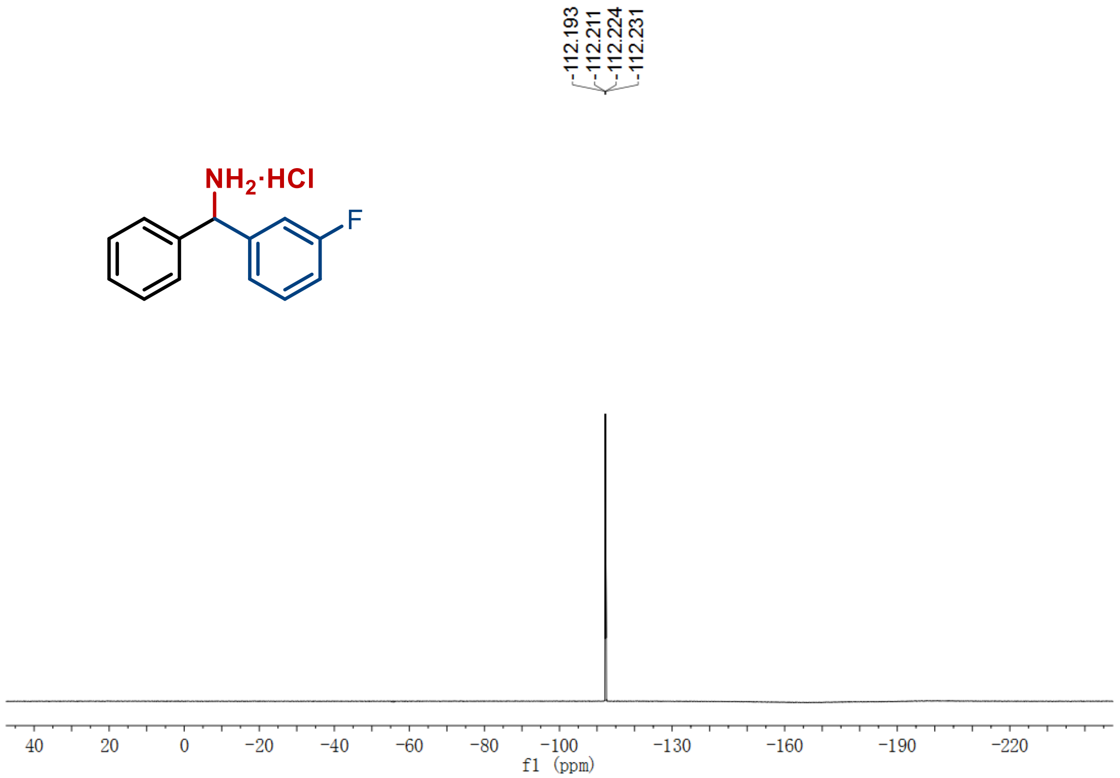

Supplementary Fig. 98 ^{19}F NMR (565 MHz, DMSO) spectrum of compound **44**.

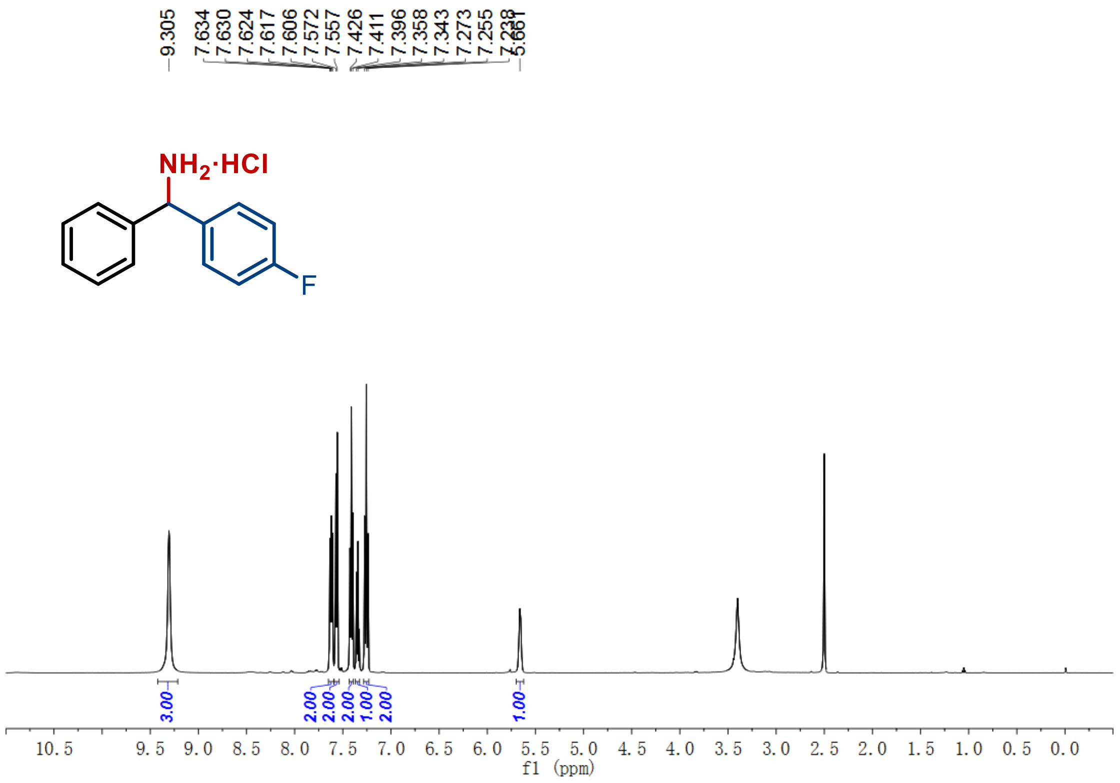

Supplementary Fig. 99 ^{1}H NMR (500 MHz, DMSO) spectrum of compound **45**.

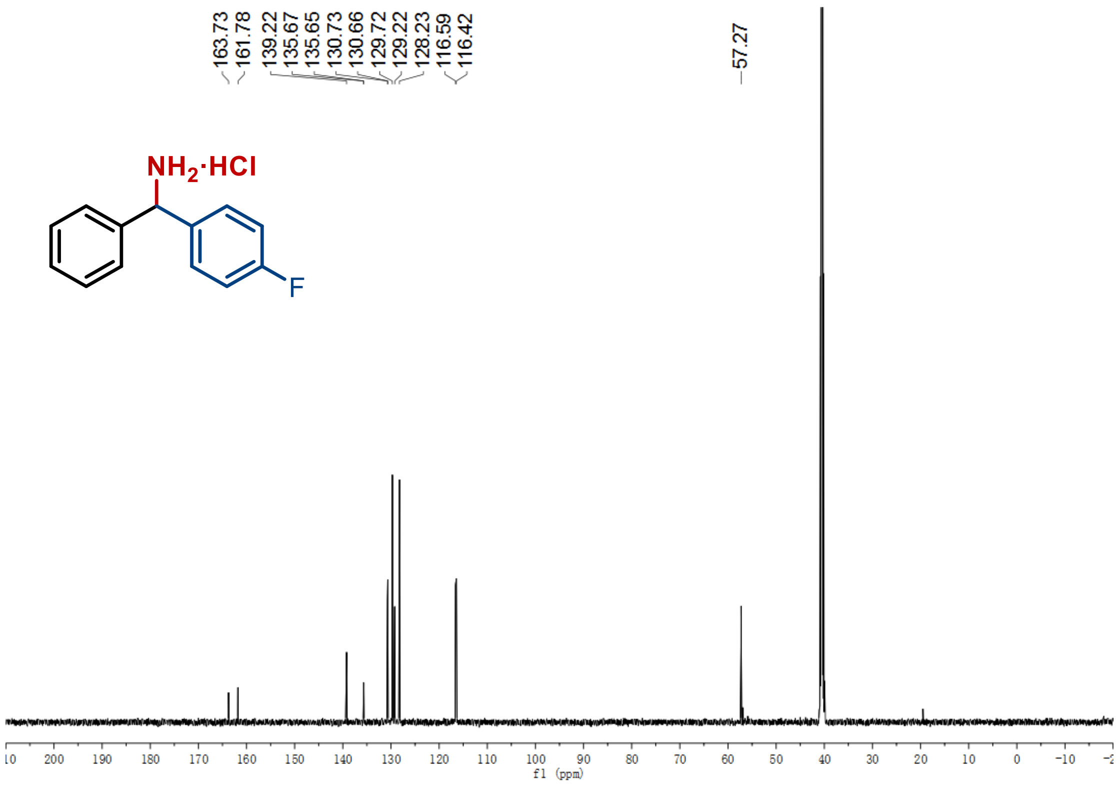

Supplementary Fig. 100 ^{13}C NMR (126 MHz, DMSO) spectrum of compound **45**.

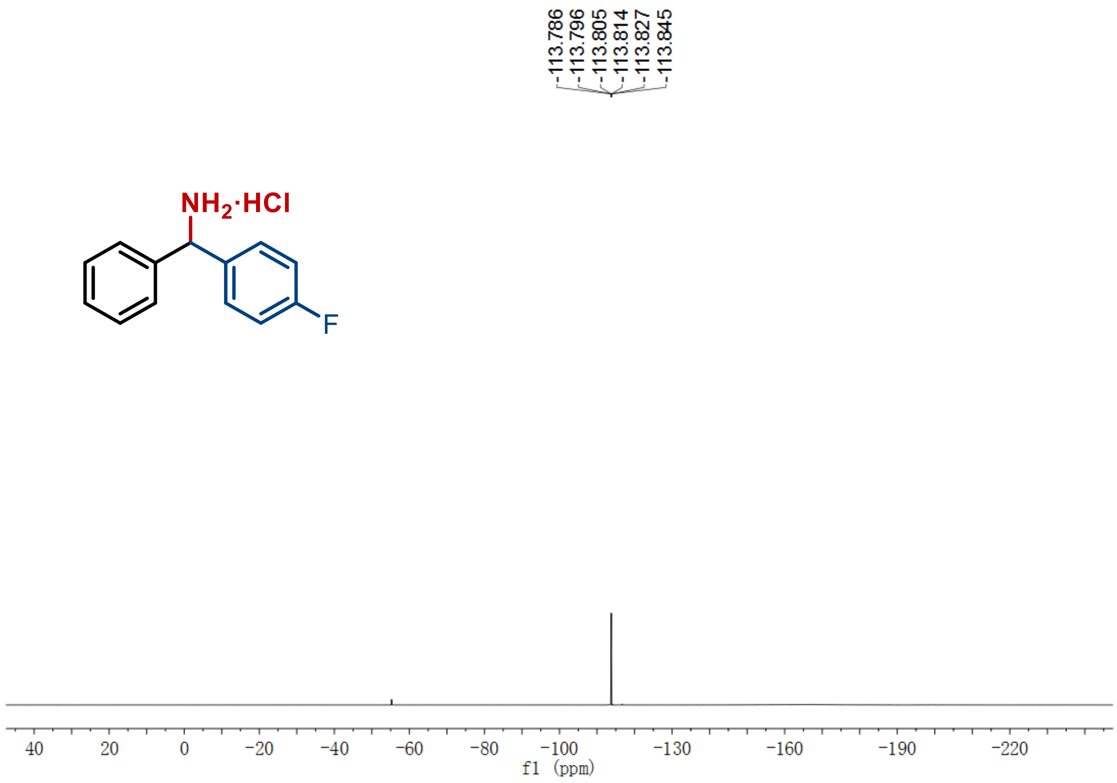

Supplementary Fig. 101 ^{19}F NMR (471 MHz, DMSO) spectrum of compound **45**.

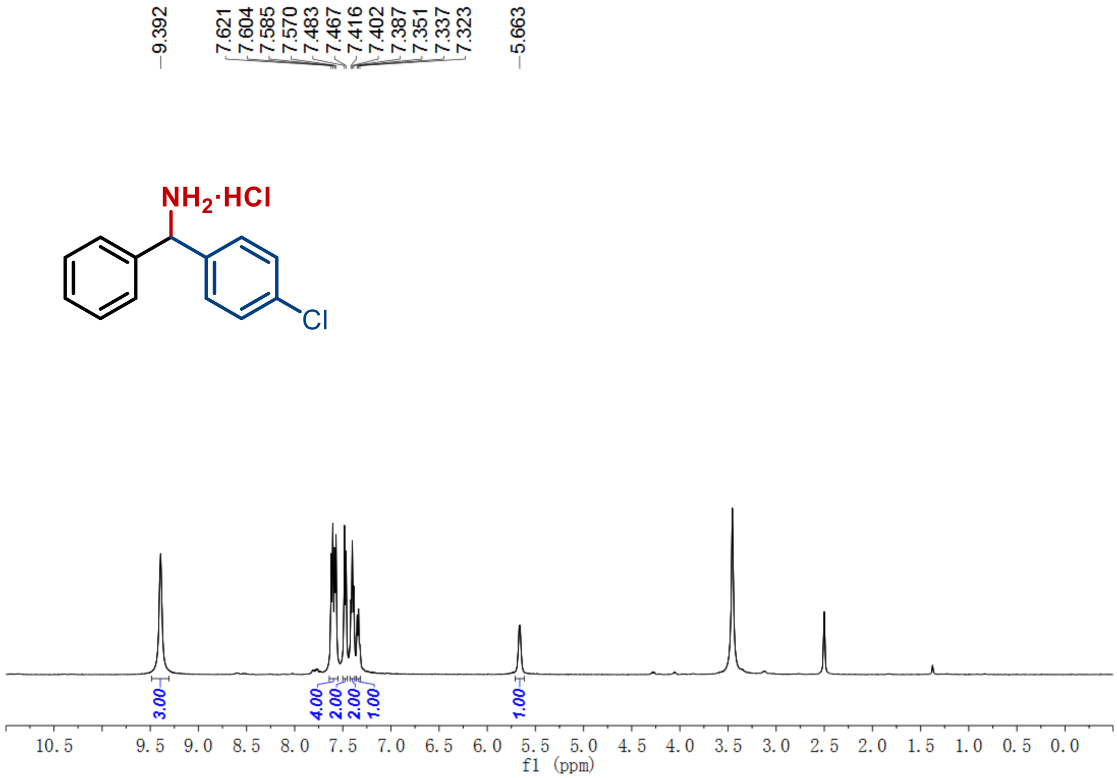

Supplementary Fig. 102 ^{1}H NMR (500 MHz, DMSO) spectrum of compound **46**.

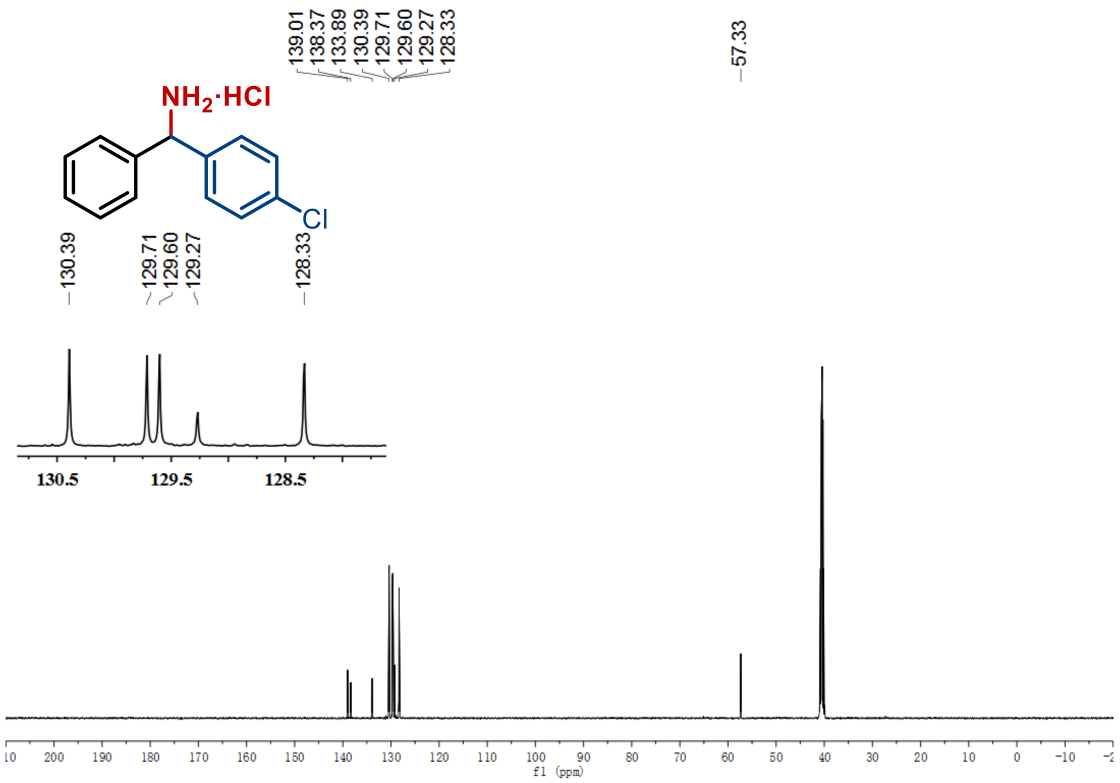

Supplementary Fig. 103 ^{13}C NMR (126 MHz, DMSO) spectrum of compound **46**.

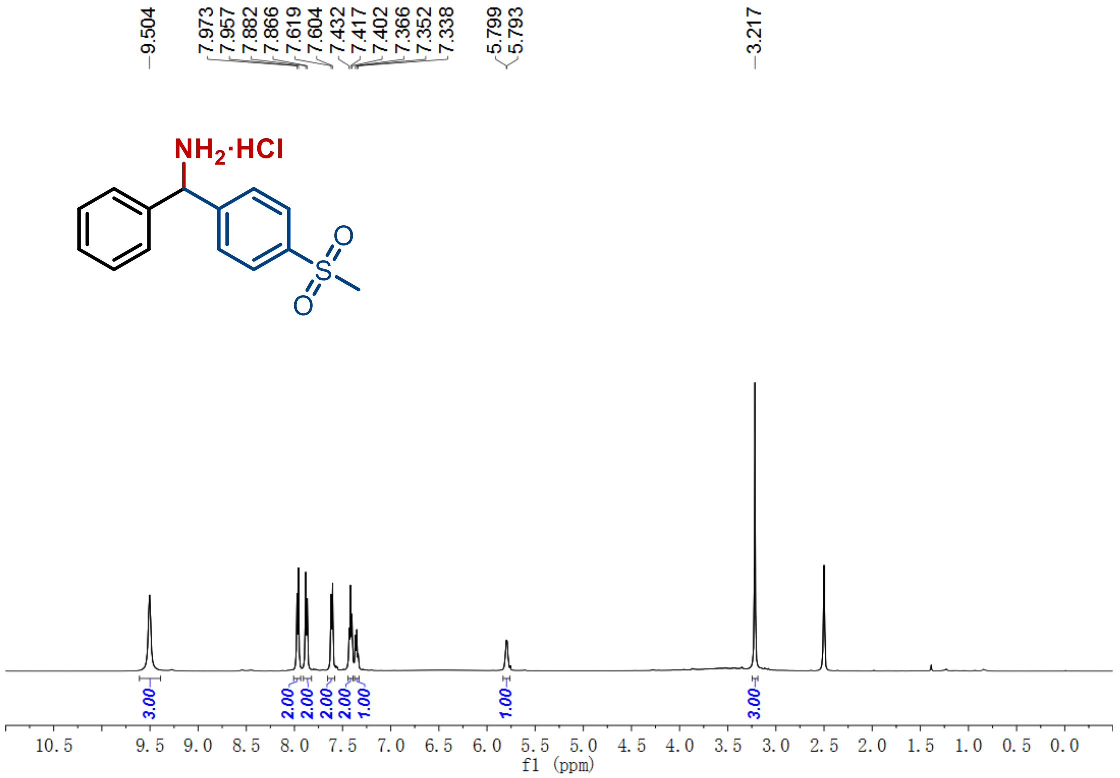

Supplementary Fig. 104 ^{1}H NMR (500 MHz, DMSO) spectrum of compound **47**.

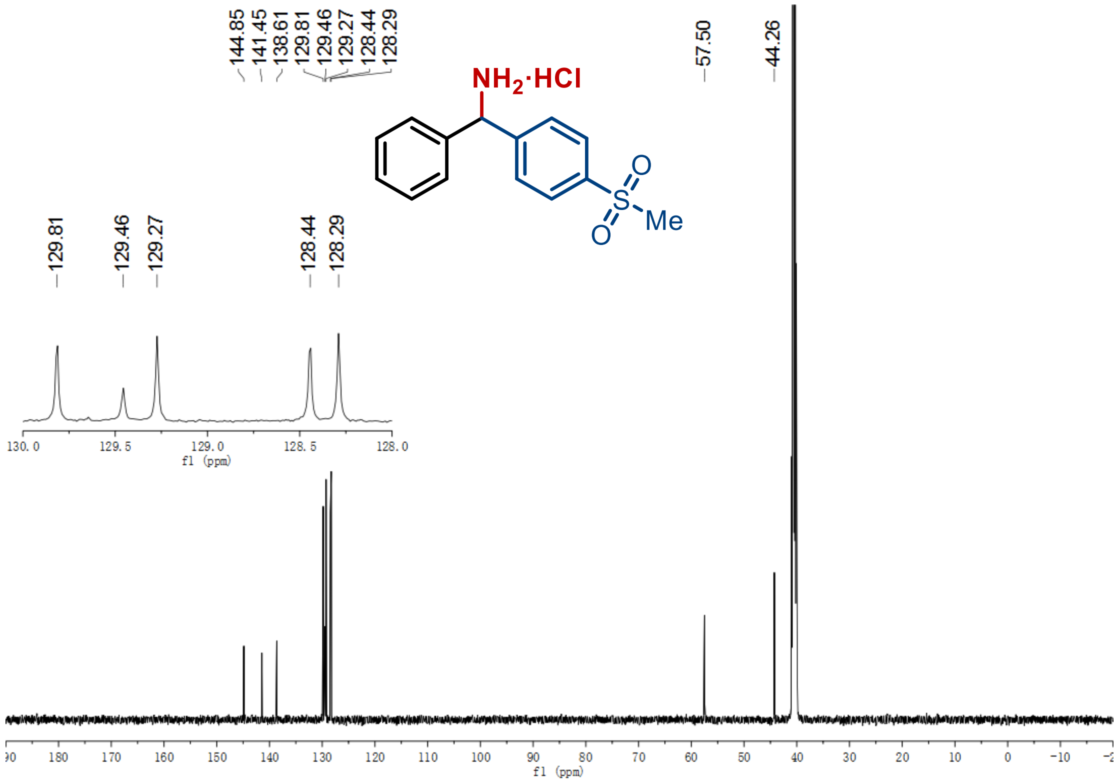

Supplementary Fig. 105 ^{13}C NMR (126 MHz, DMSO) spectrum of compound **47**.

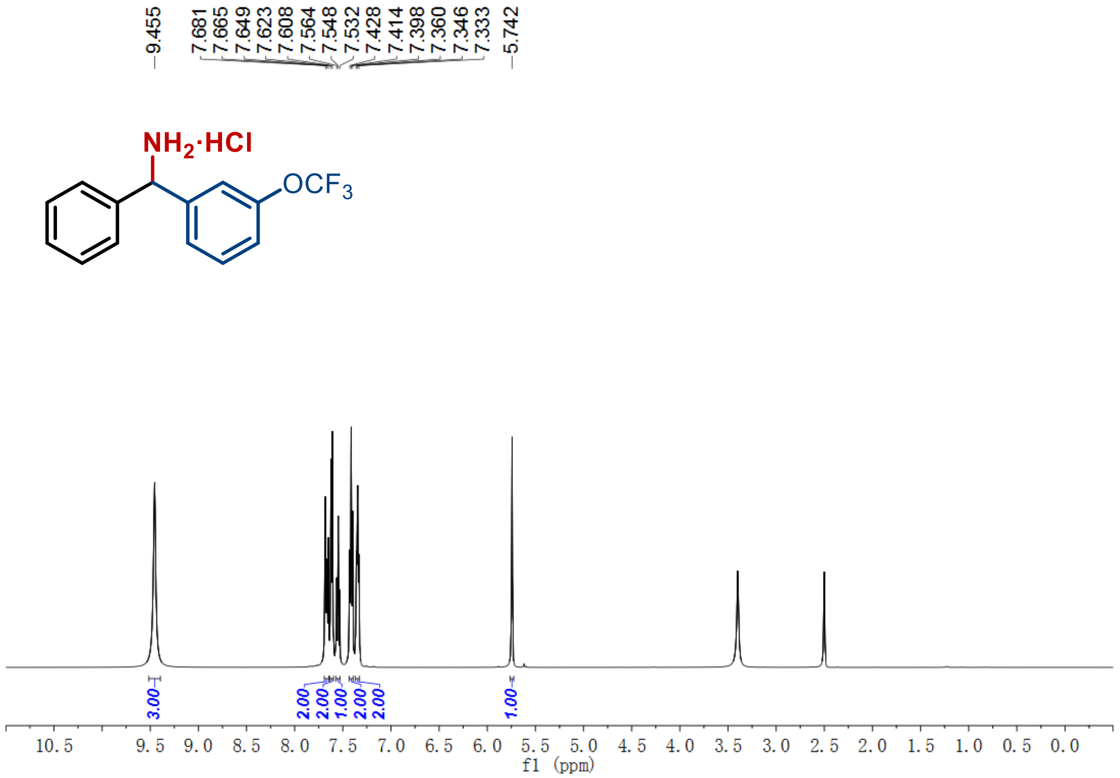

Supplementary Fig. 106 ^{1}H NMR (500 MHz, DMSO) spectrum of compound **48**.

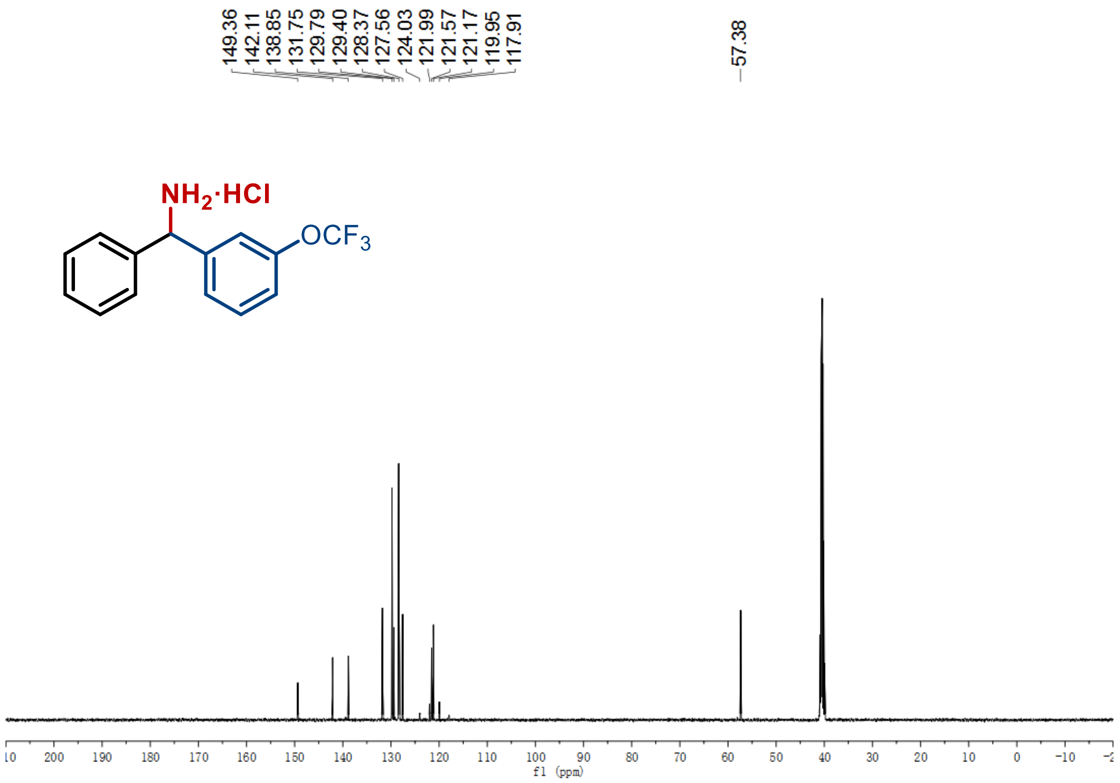

Supplementary Fig. 107 ^{13}C NMR (126 MHz, DMSO) spectrum of compound **48**.

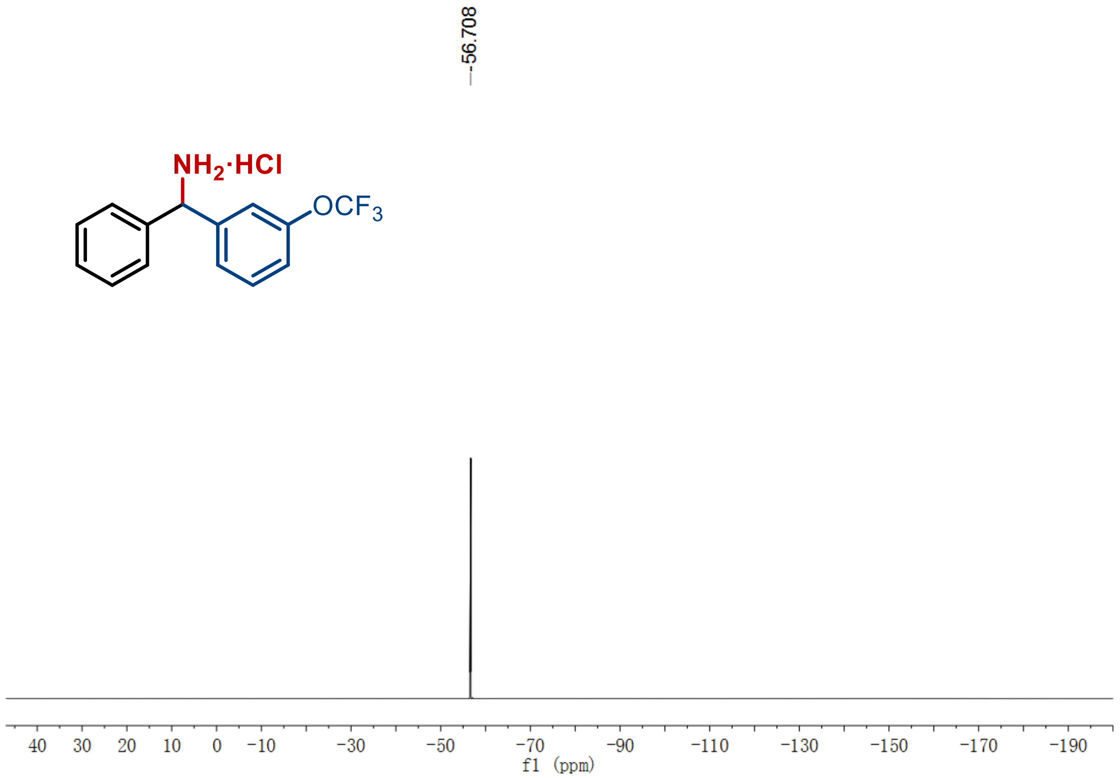

Supplementary Fig. 108 ^{19}F NMR (471 MHz, DMSO) spectrum of compound **48**.

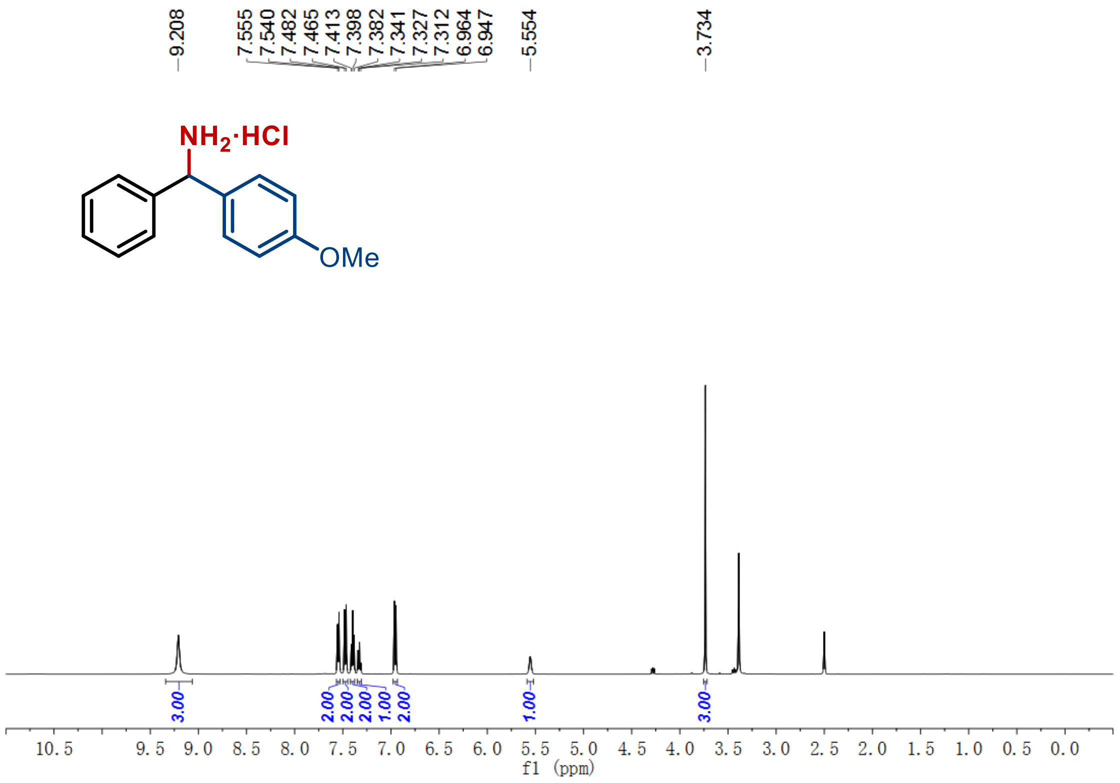

Supplementary Fig. 109 ^{1}H NMR (500 MHz, DMSO) spectrum of compound **49**.

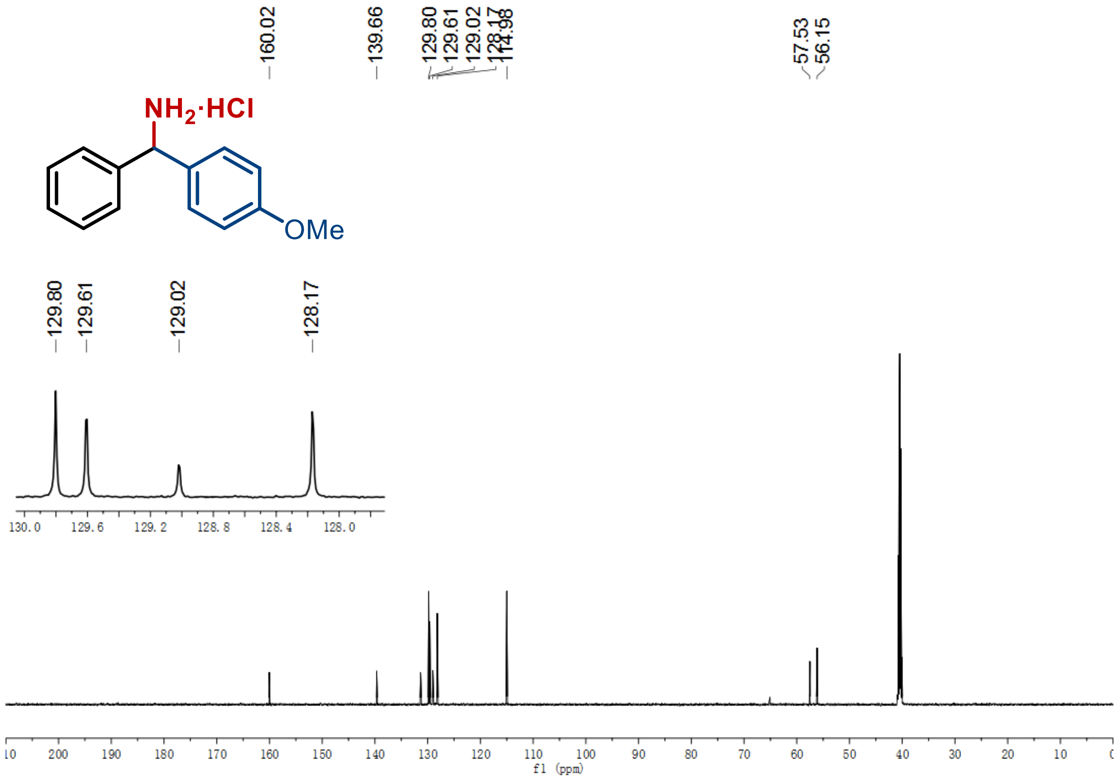

Supplementary Fig. 110 ^{13}C NMR (151 MHz, DMSO) spectrum of compound **49**.

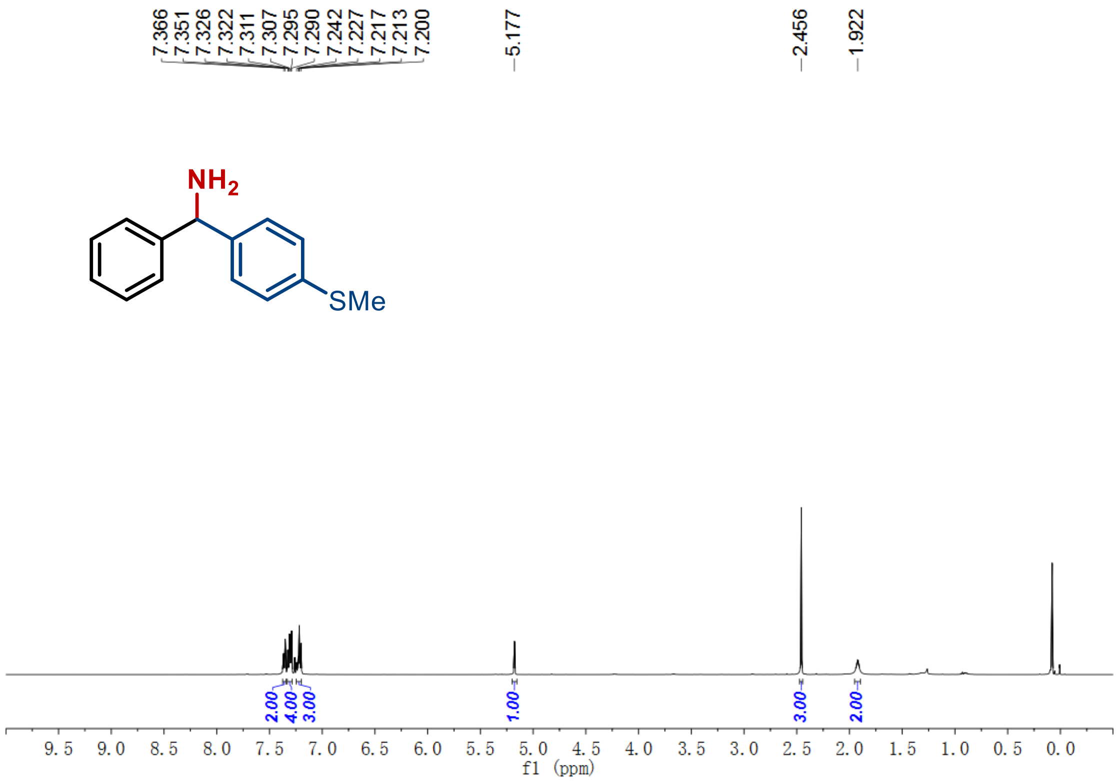

Supplementary Fig. 111 ^{1}H NMR (500 MHz, $CDCl_3$) spectrum of compound **50**.

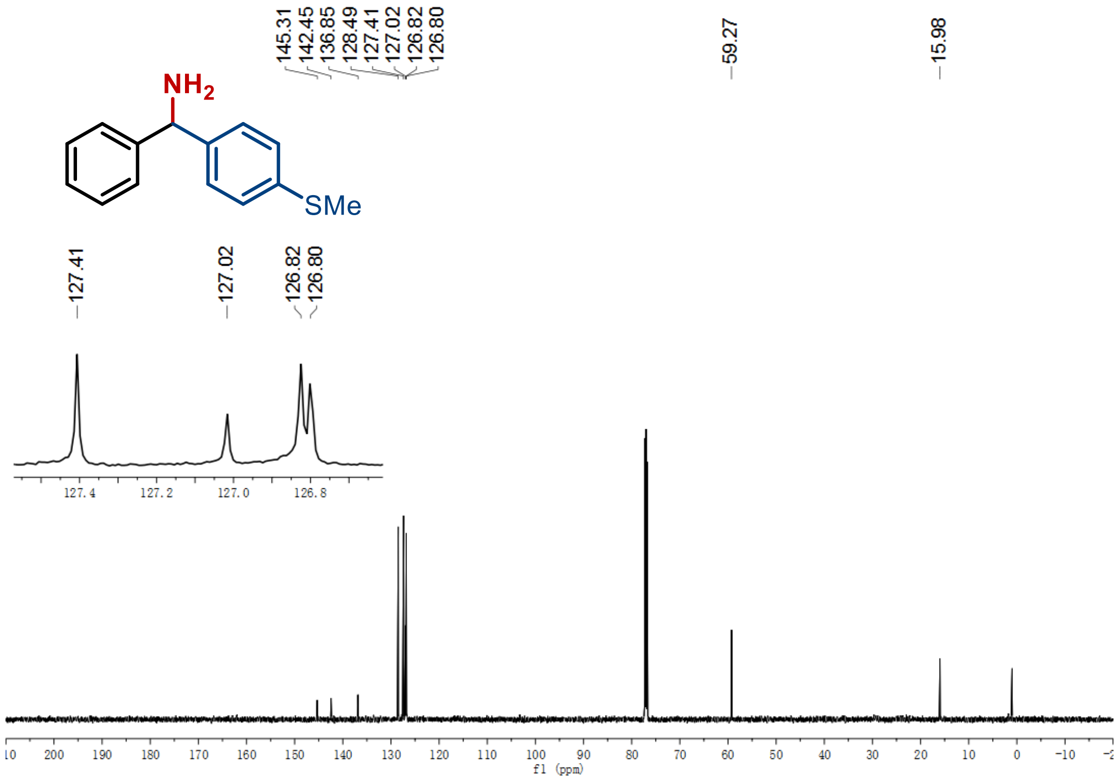

Supplementary Fig. 112 ^{13}C NMR (151MHz, $CDCl_3$) spectrum of compound **50**.

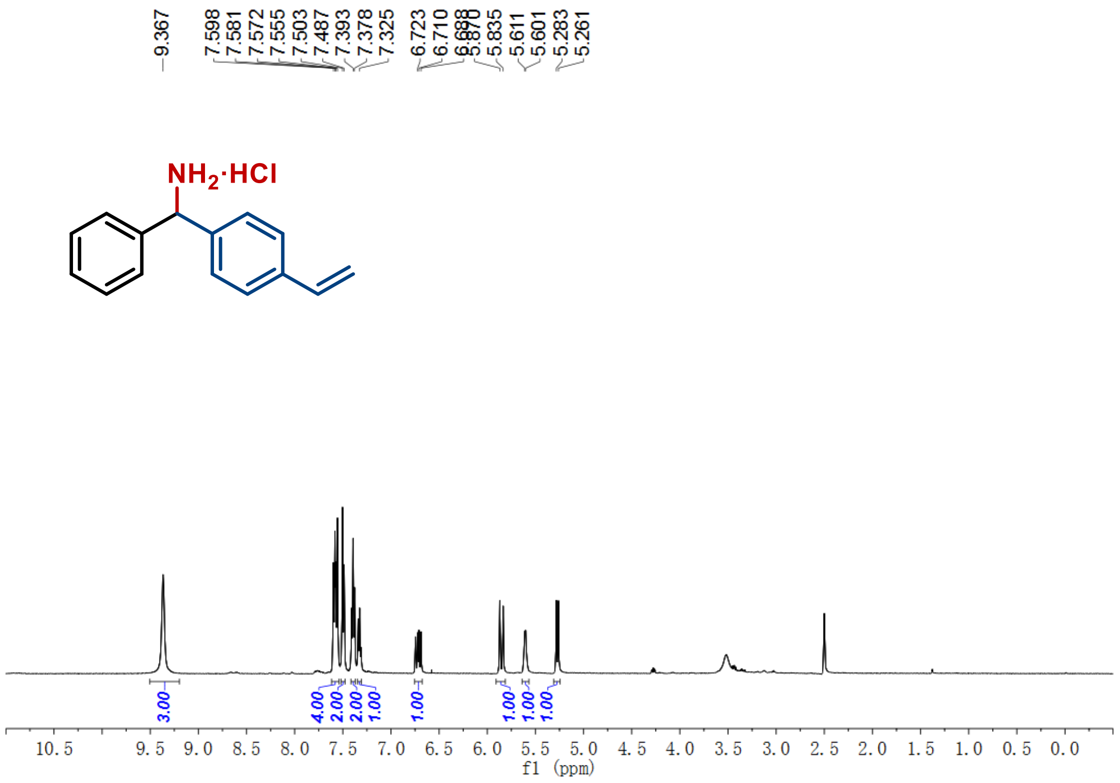

Supplementary Fig. 113 ^{1}H NMR (500 MHz, DMSO) spectrum of compound **51**.

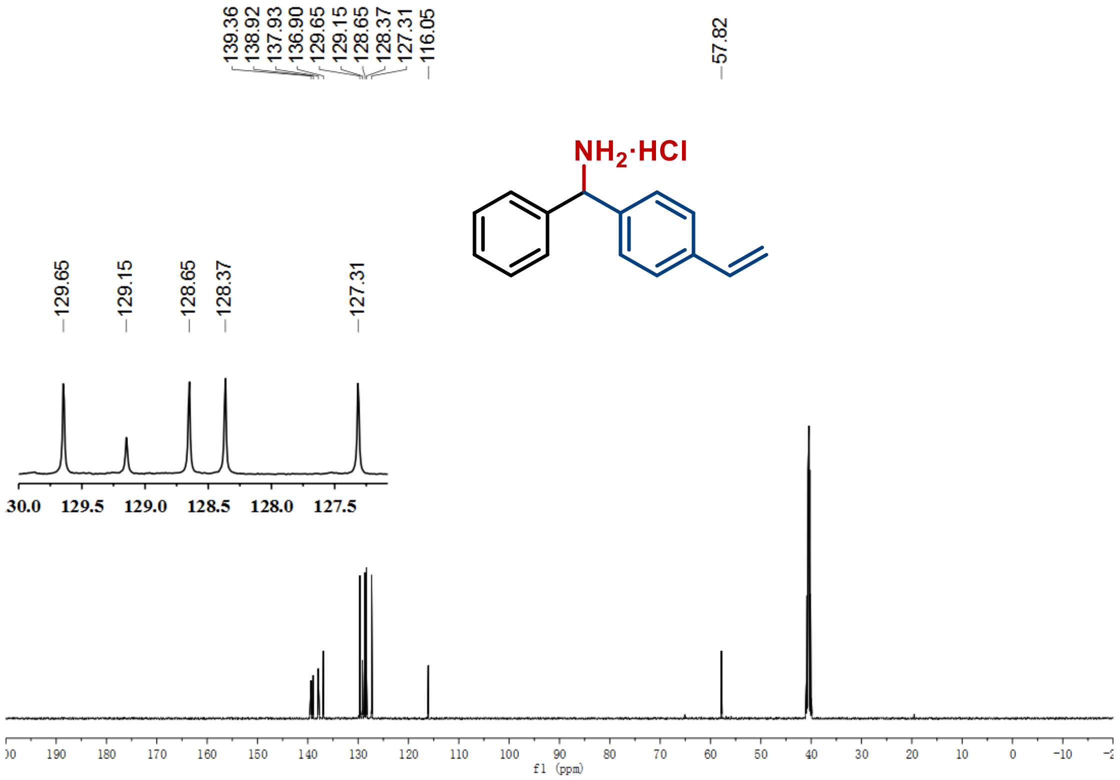

Supplementary Fig. 114 ^{13}C NMR (126 MHz, DMSO) spectrum of compound **51**.

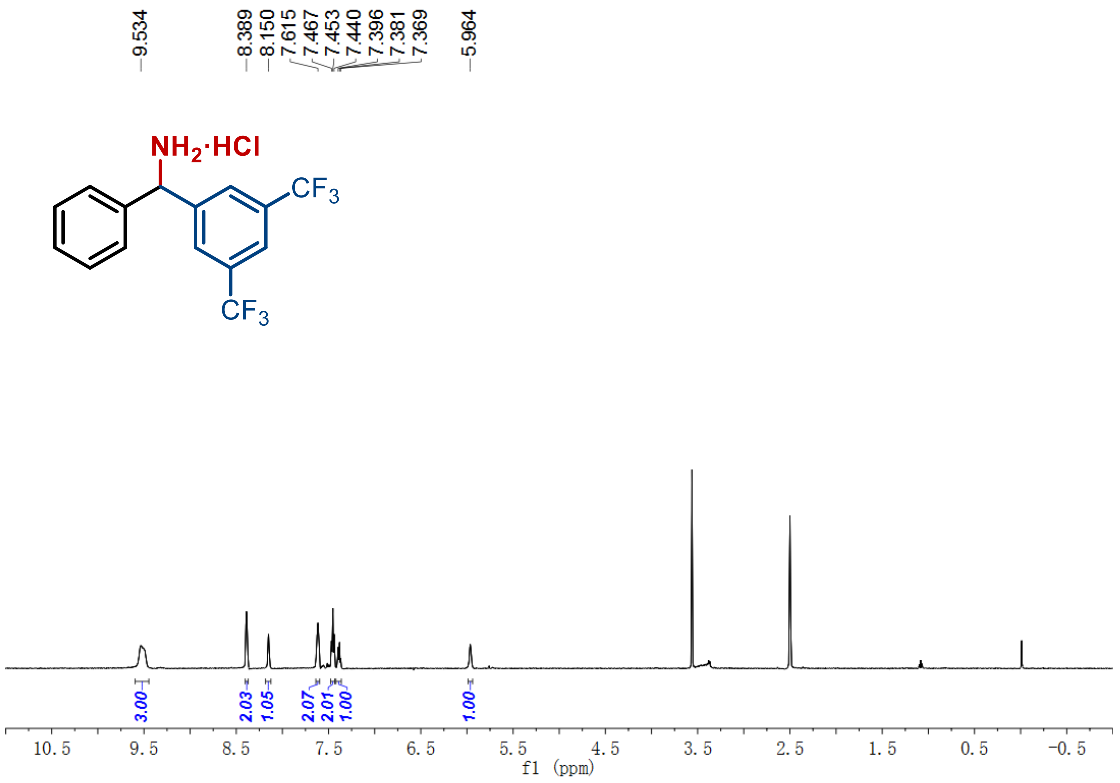

Supplementary Fig. 115 ^{1}H NMR (500 MHz, DMSO) spectrum of compound **52**.

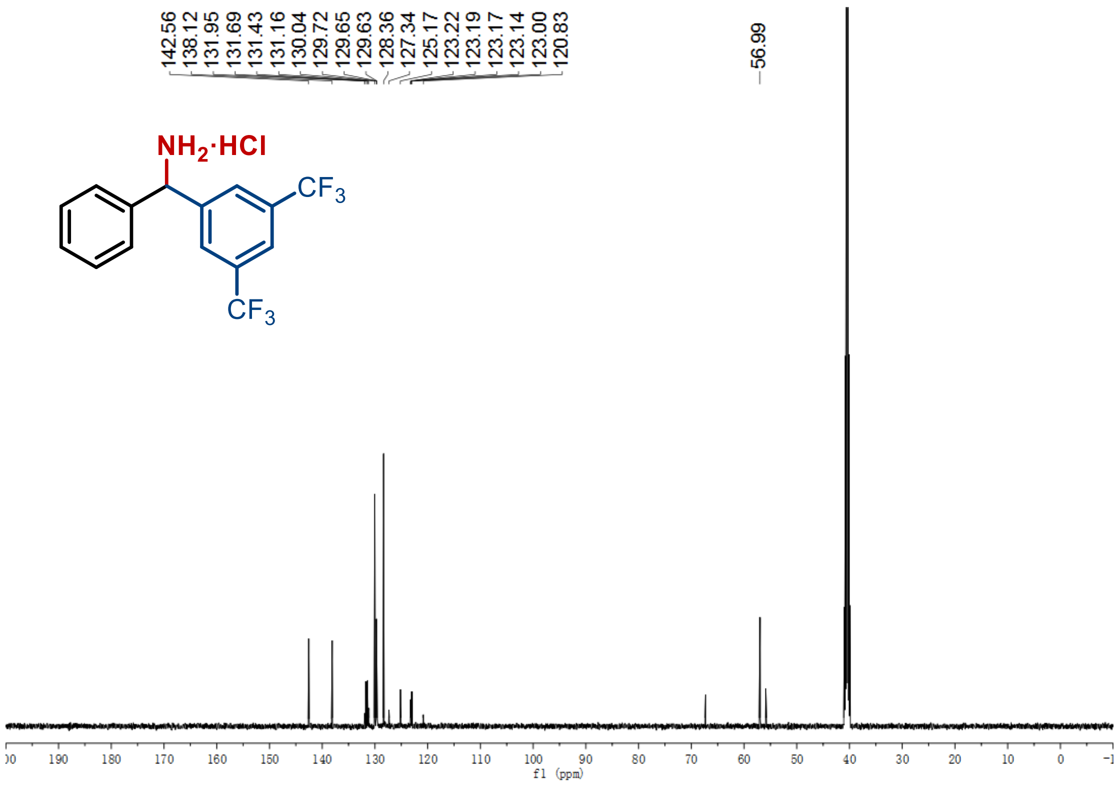

Supplementary Fig. 116 ^{13}C NMR (126 MHz, DMSO) spectrum of compound **52**.

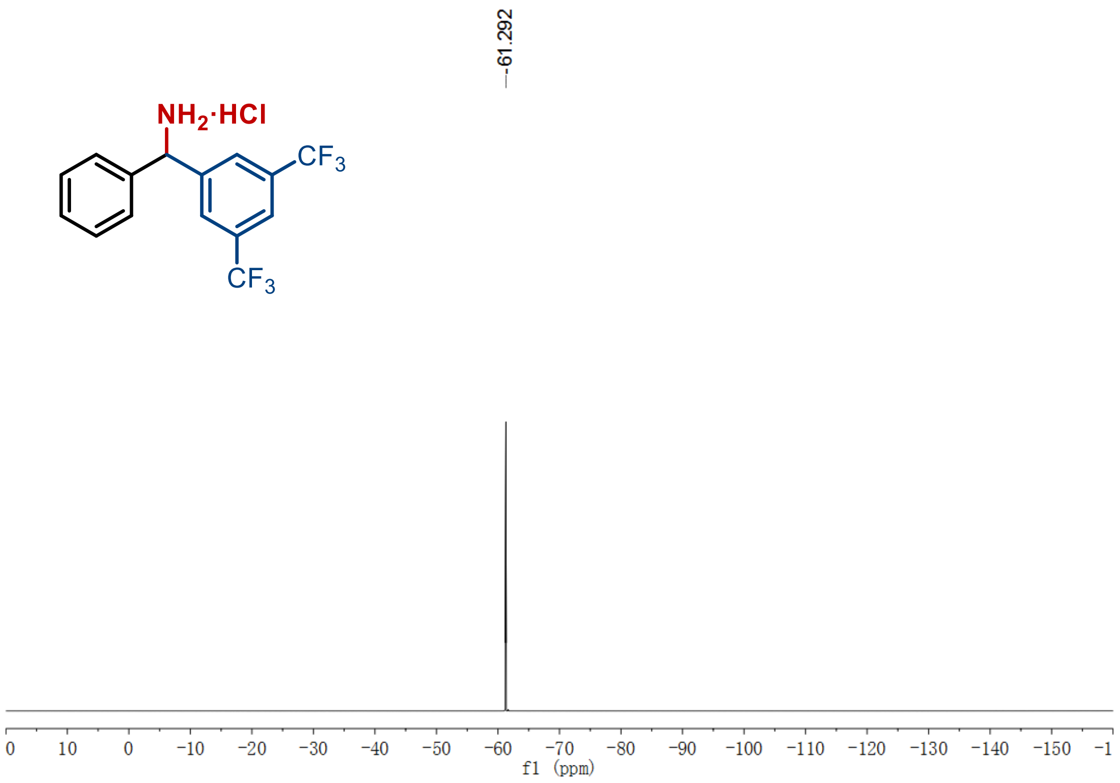

Supplementary Fig. 117 ^{19}F NMR (565 MHz, DMSO) spectrum of compound **52**.

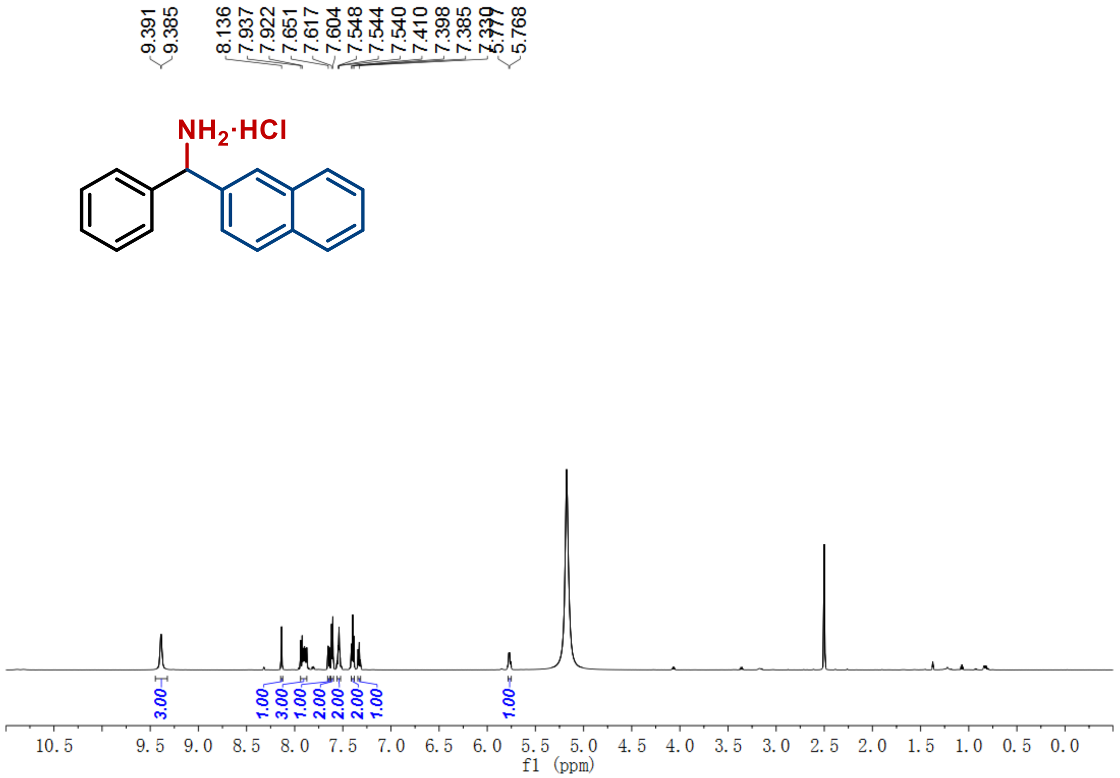

Supplementary Fig. 118 ^{1}H NMR (600 MHz, DMSO) spectrum of compound **53**.

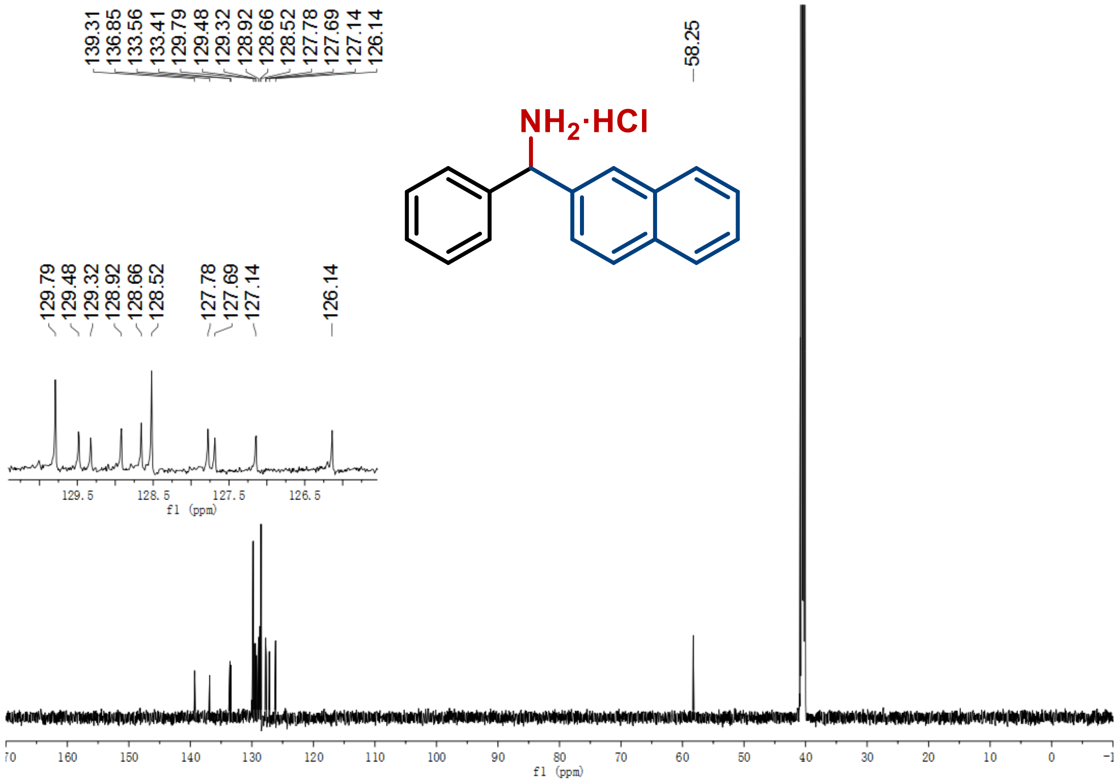

Supplementary Fig. 119 ^{13}C NMR (151 MHz, DMSO) spectrum of compound **53**.

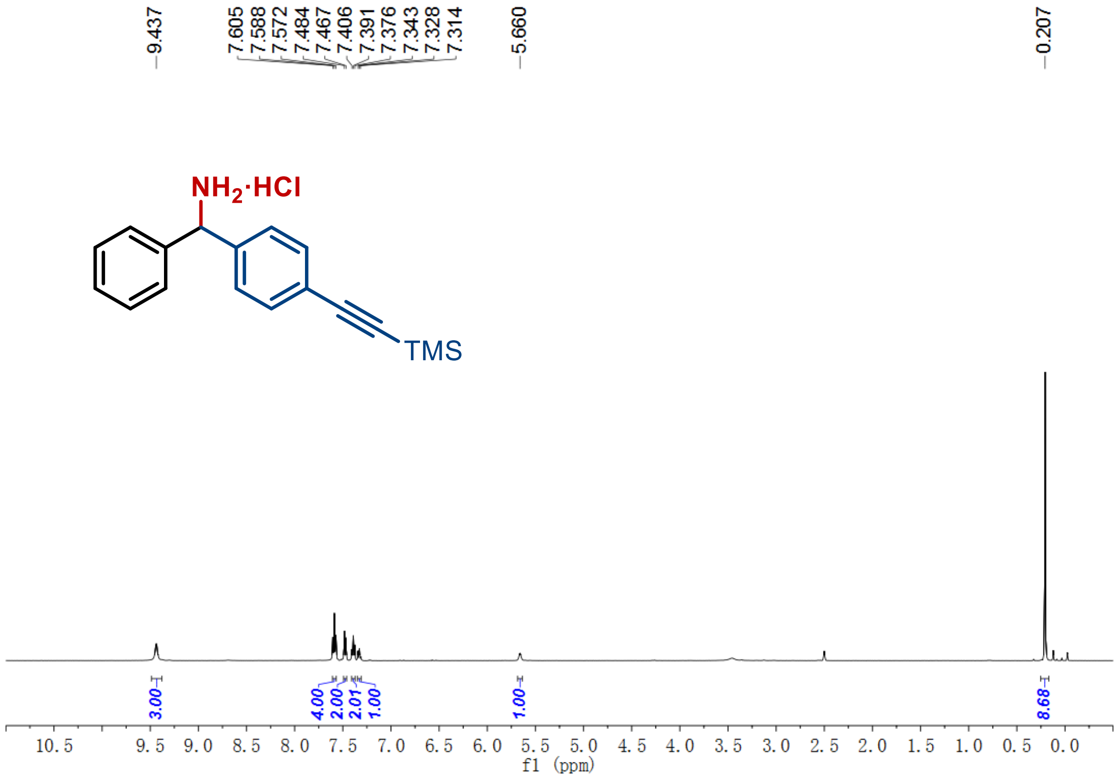

Supplementary Fig. 120 ^{1}H NMR (500 MHz, DMSO) spectrum of compound **54**.

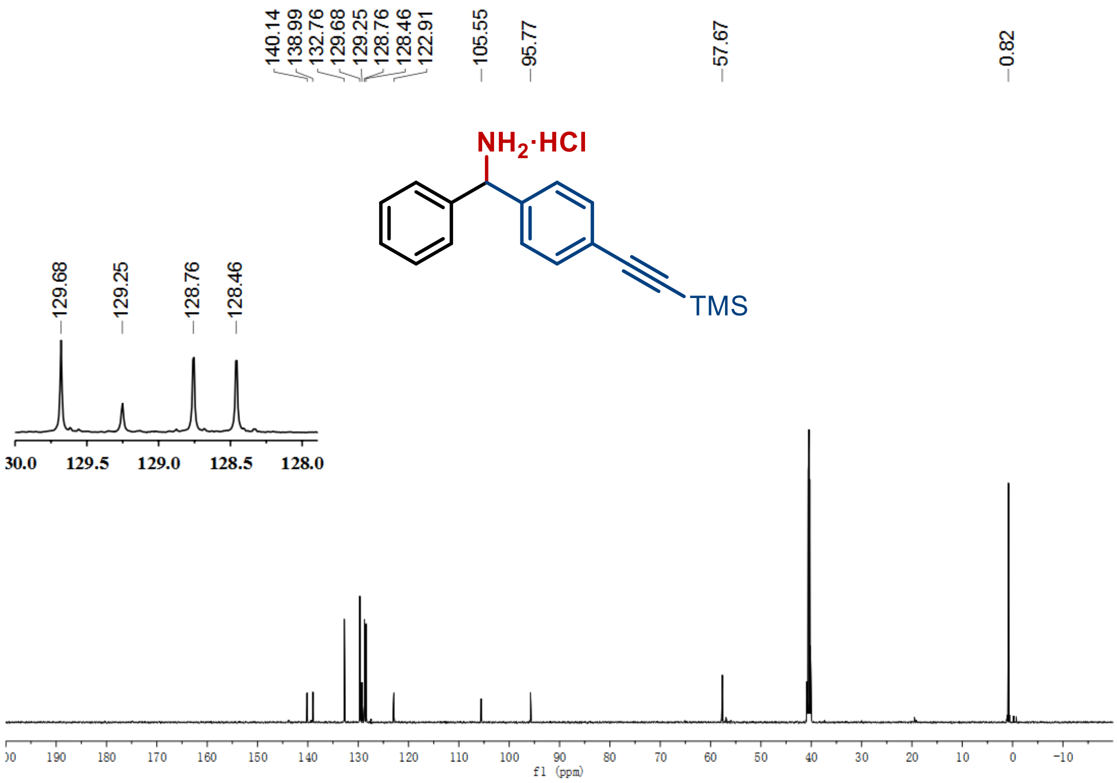

Supplementary Fig. 121 ^{13}C NMR (151 MHz, DMSO) spectrum of compound **54**.

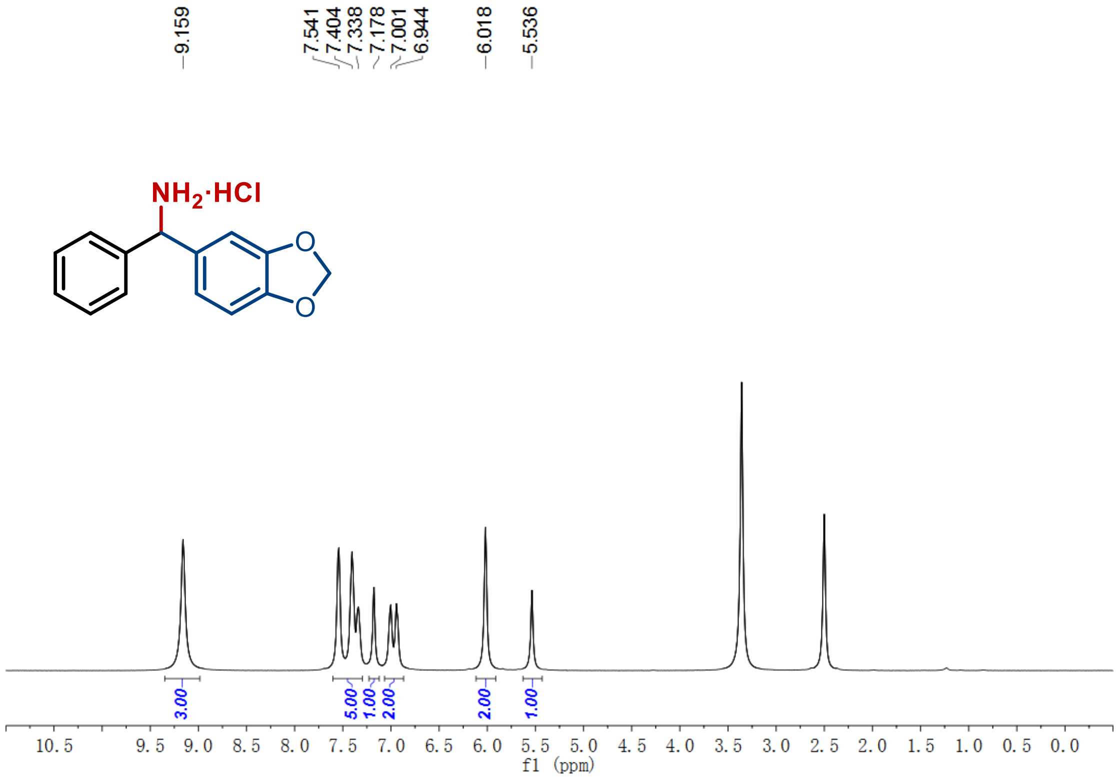

Supplementary Fig. 122 ^{1}H NMR (500 MHz, DMSO) spectrum of compound **55**.

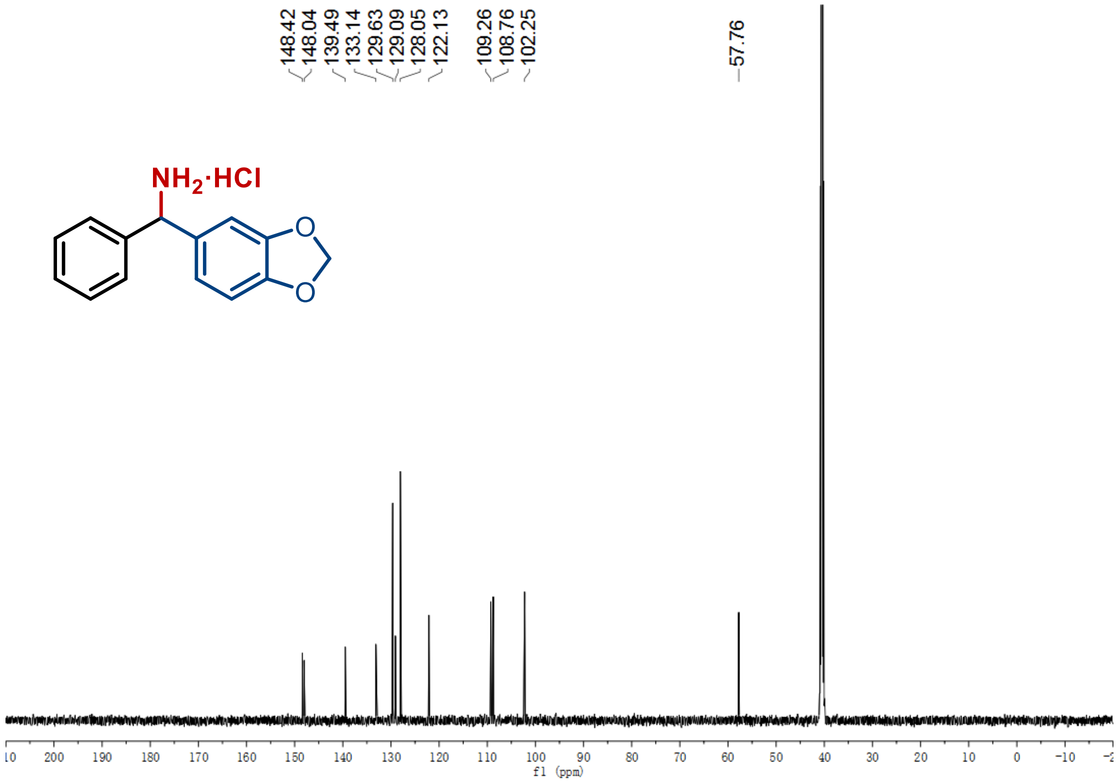

Supplementary Fig. 123 ^{13}C NMR (126 MHz, DMSO) spectrum of compound **55**.

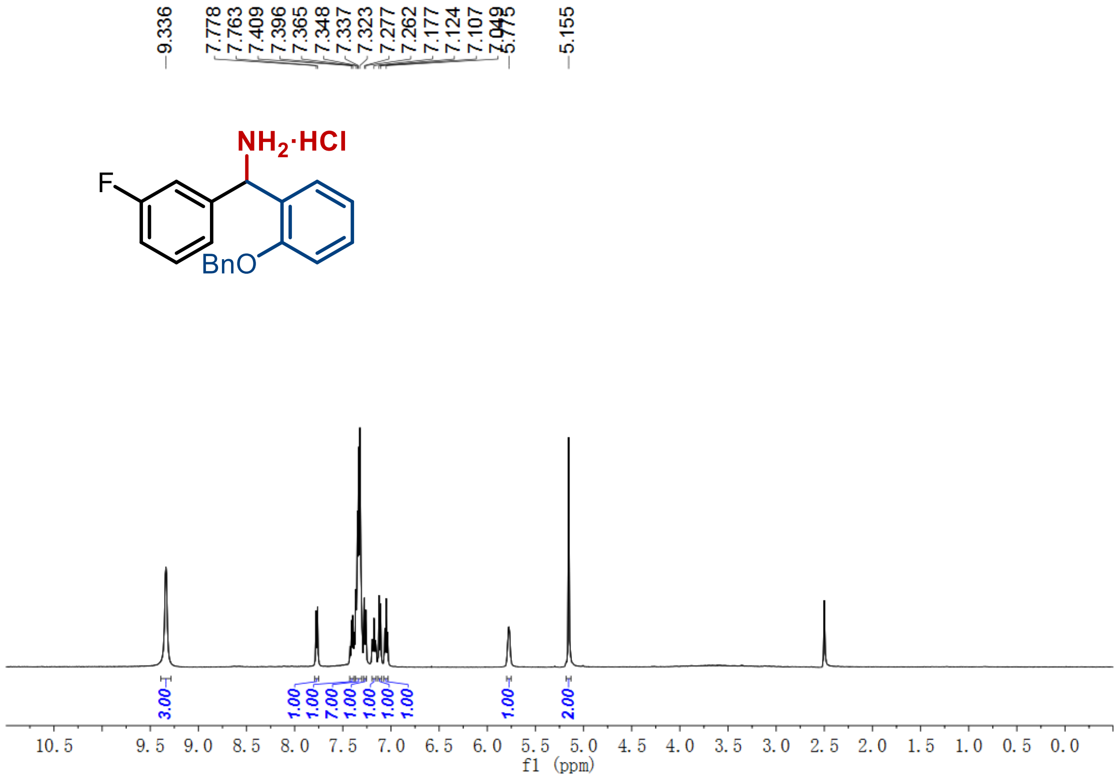

Supplementary Fig. 124 ^{1}H NMR (500 MHz, DMSO) spectrum of compound **56**.

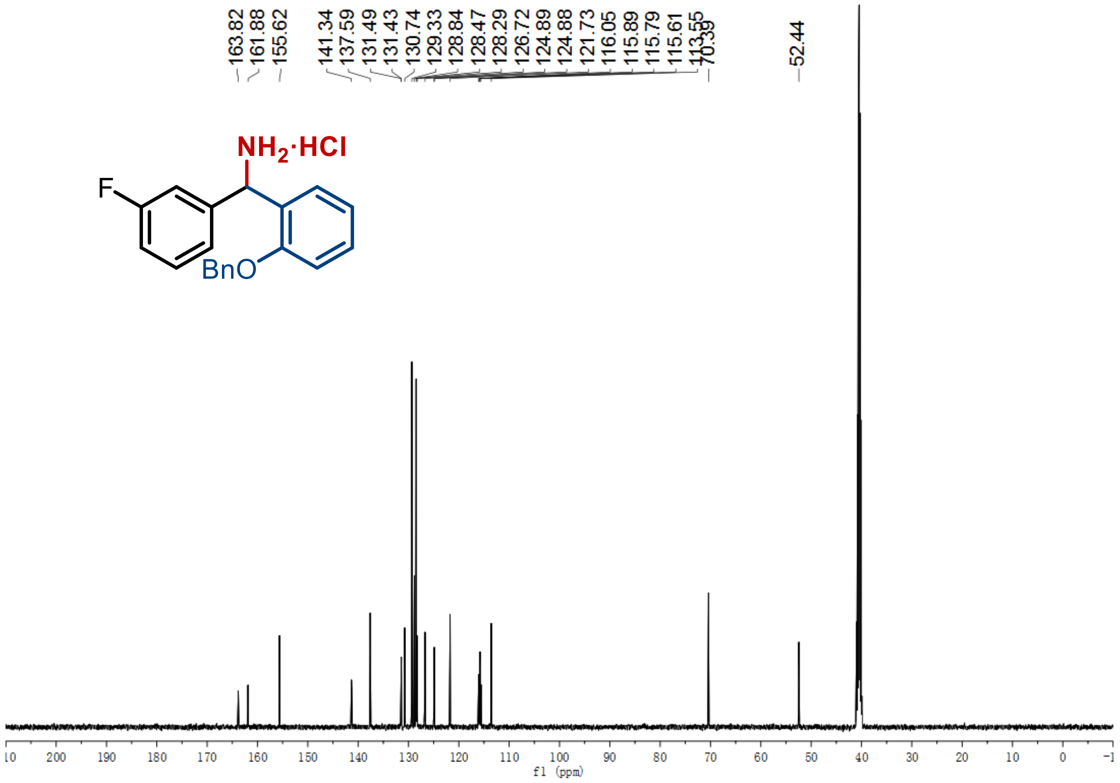

Supplementary Fig. 125 ^{13}C NMR (126 MHz, DMSO) spectrum of compound **56**.

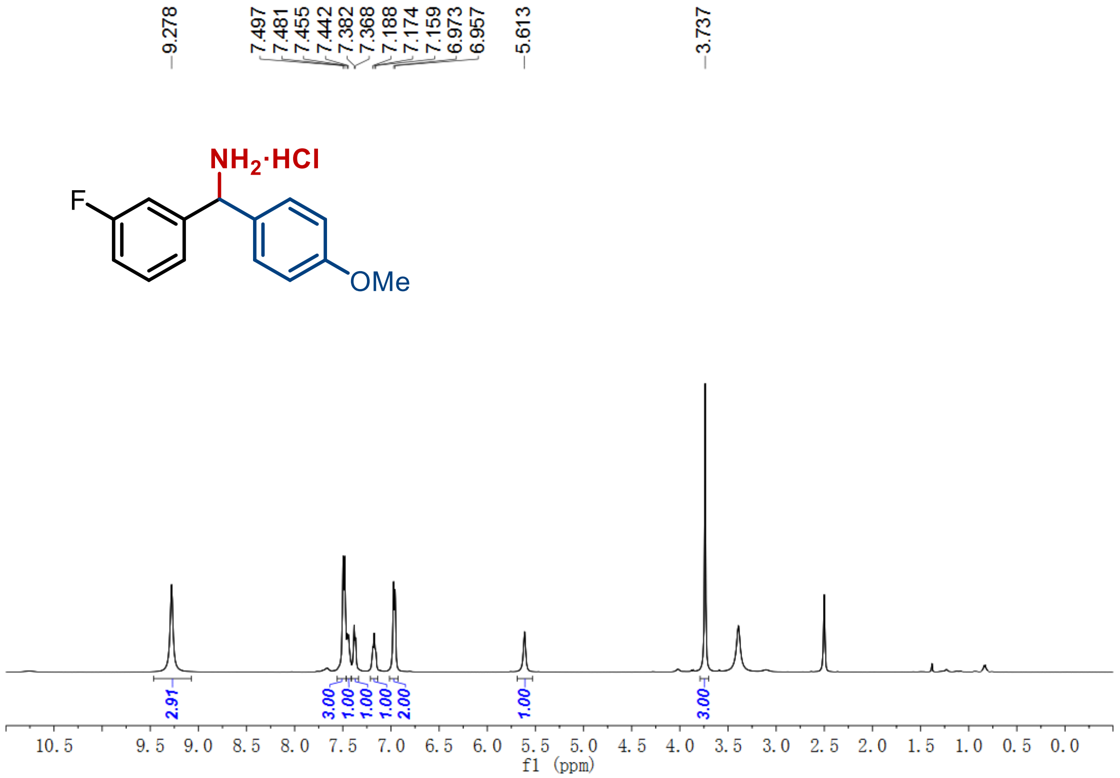

Supplementary Fig. 126 ^{1}H NMR (500 MHz, DMSO) spectrum of compound **57**.

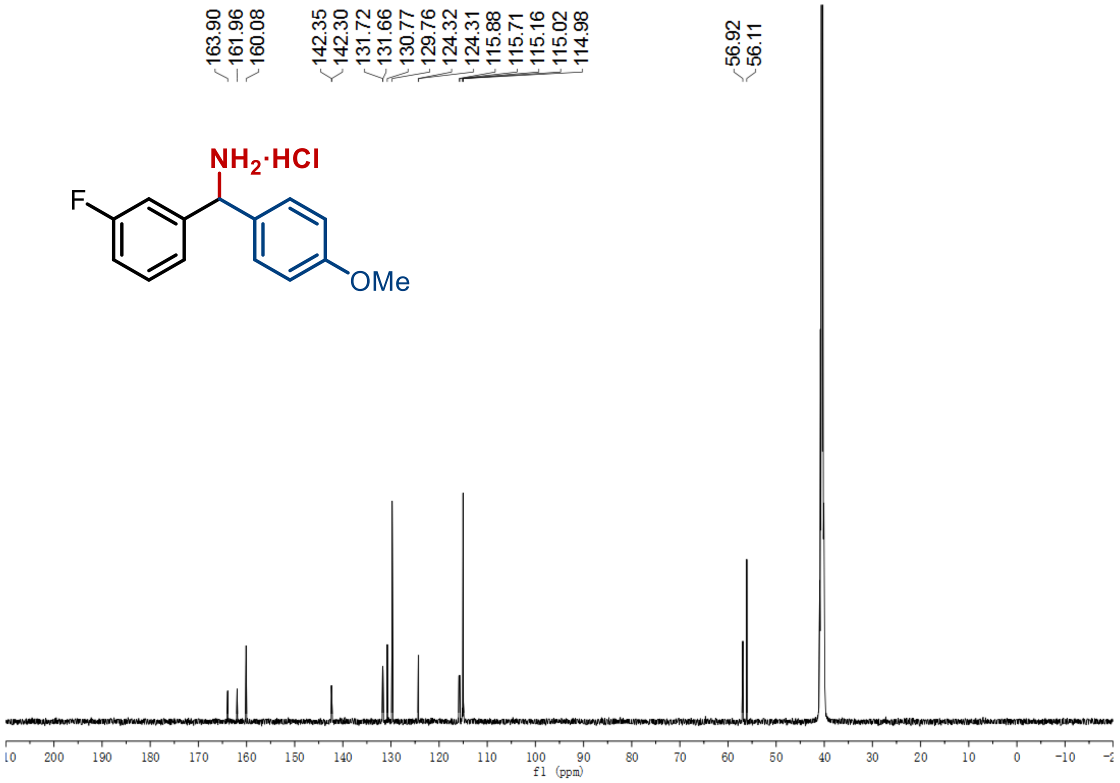

Supplementary Fig. 127 ^{13}C NMR (126 MHz, DMSO) spectrum of compound **57**.

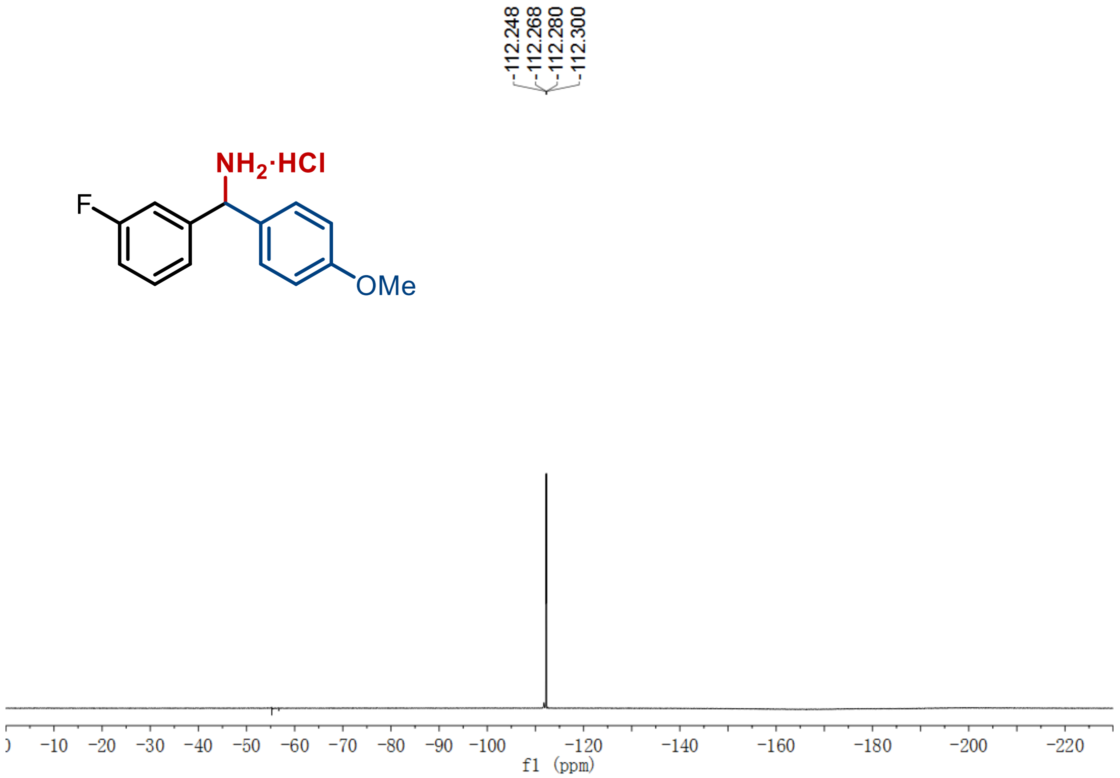

Supplementary Fig. 128 ^{19}F NMR (565 MHz, DMSO) spectrum of compound **57**.

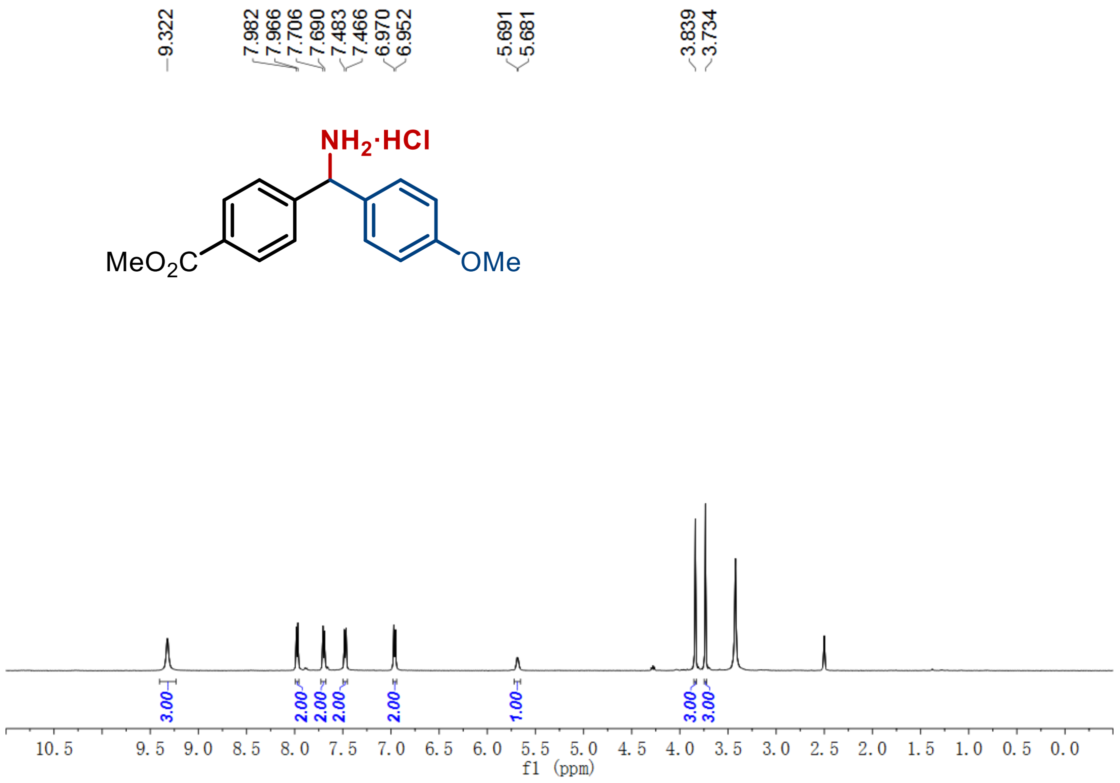

Supplementary Fig. 129 ^{1}H NMR (500 MHz, DMSO) spectrum of compound **58**.

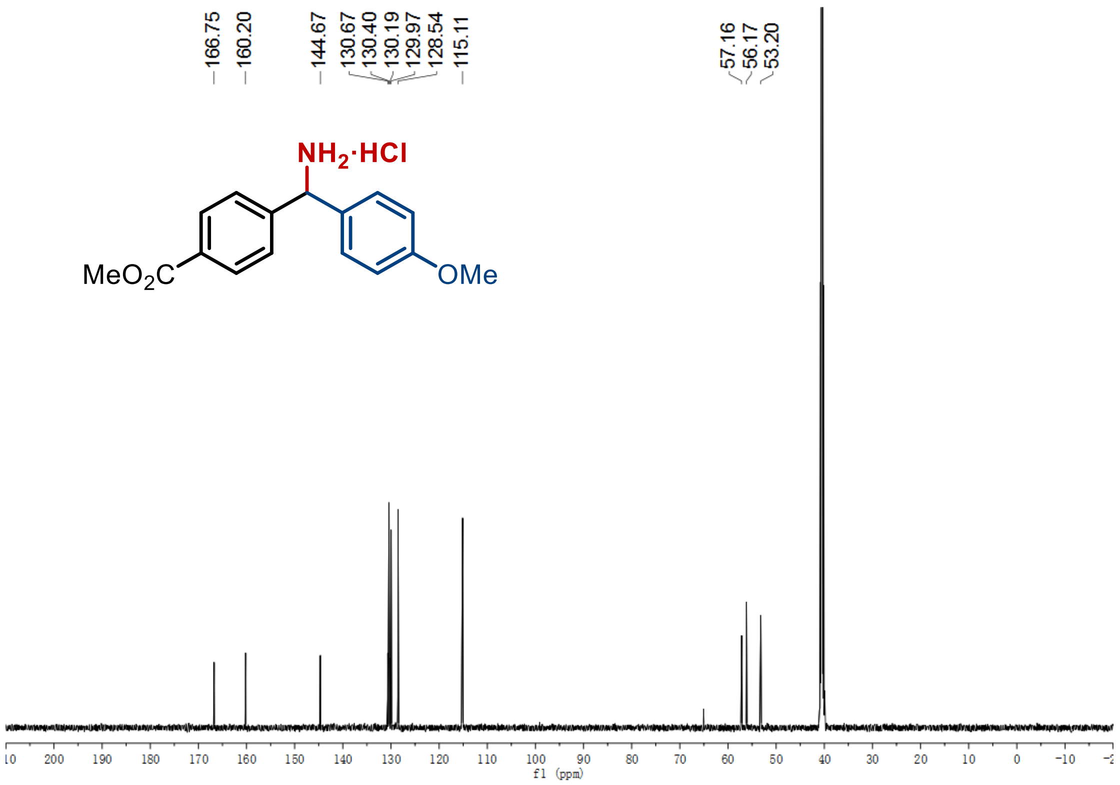

Supplementary Fig. 130 ^{13}C NMR (126 MHz, DMSO) spectrum of compound **58**.

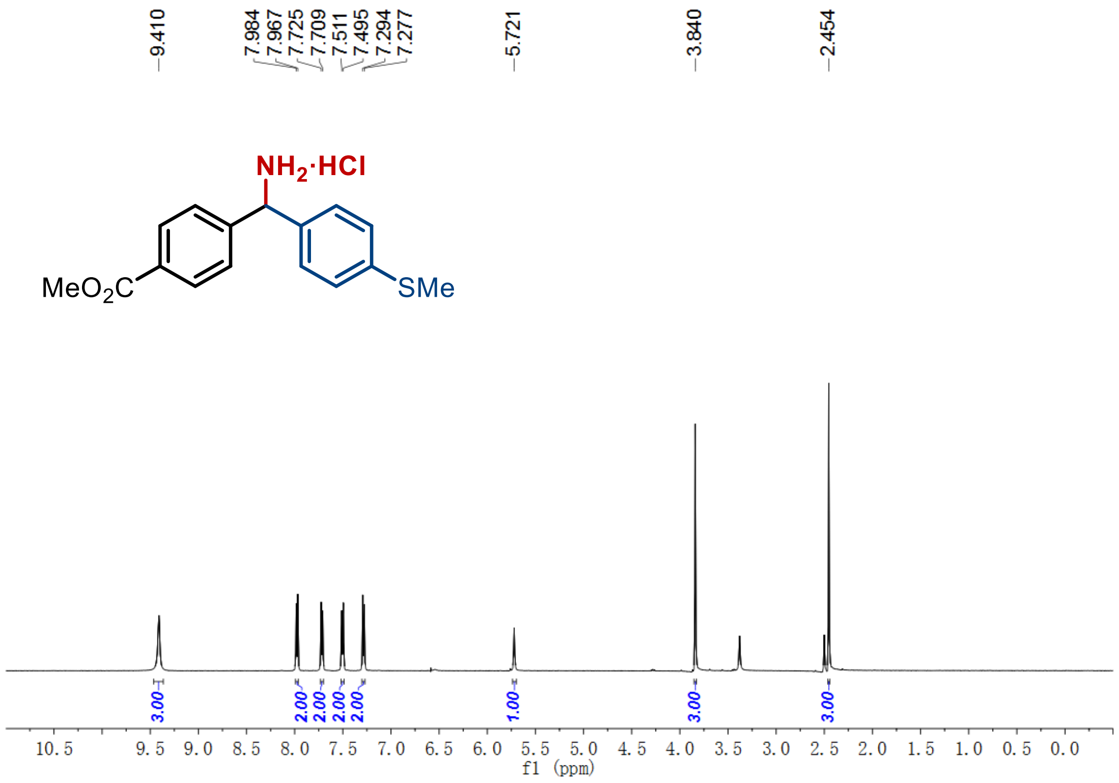

Supplementary Fig. 131 ^{1}H NMR (500 MHz, DMSO) spectrum of compound **59**.

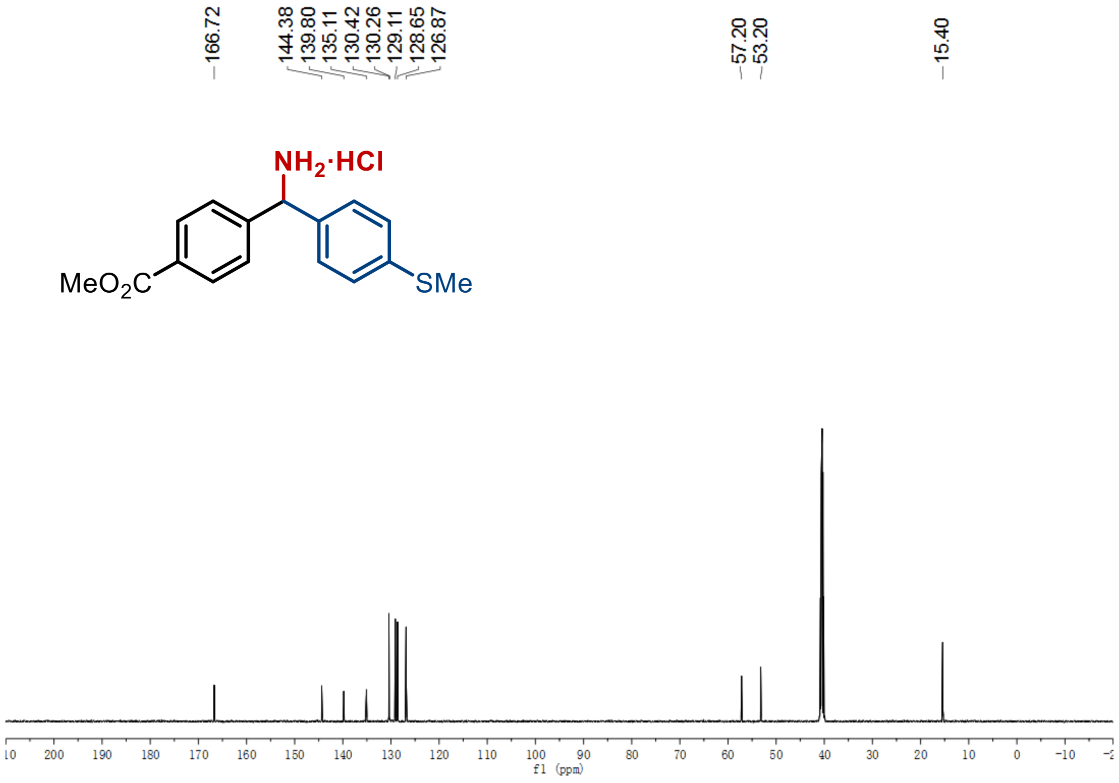

Supplementary Fig. 132 ^{13}C NMR (126 MHz, DMSO) spectrum of compound **59**.

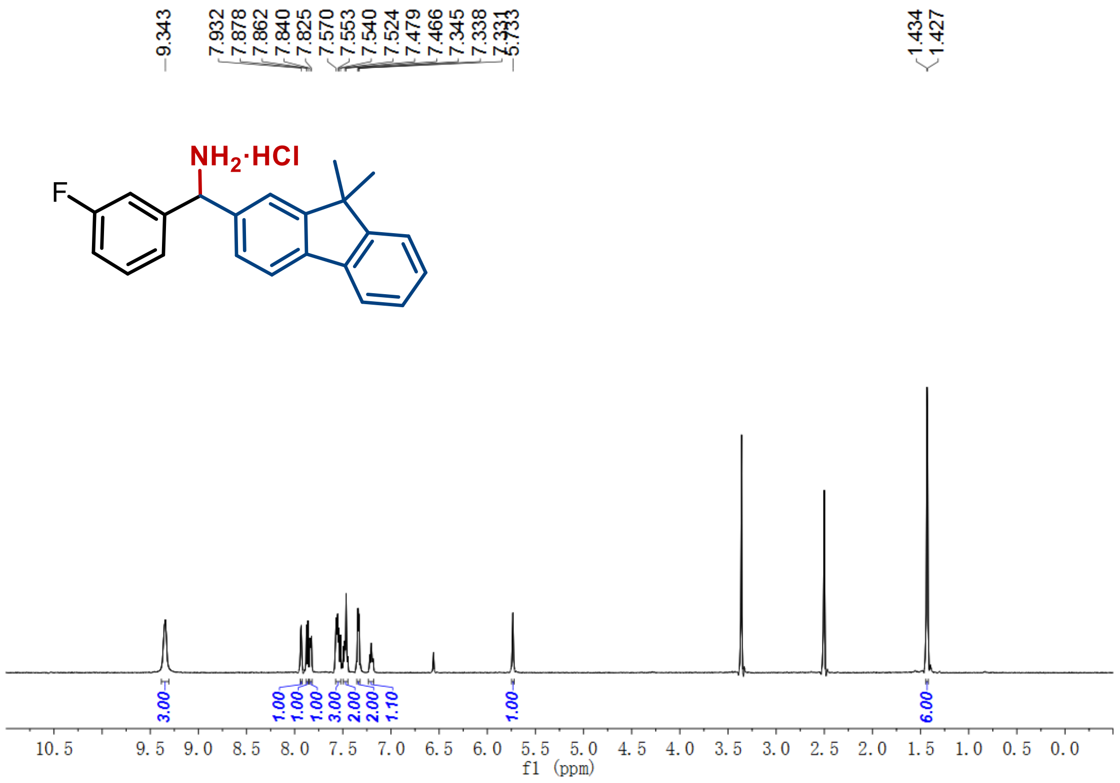

Supplementary Fig. 133 ^{1}H NMR (500 MHz, DMSO) spectrum of compound **60**.

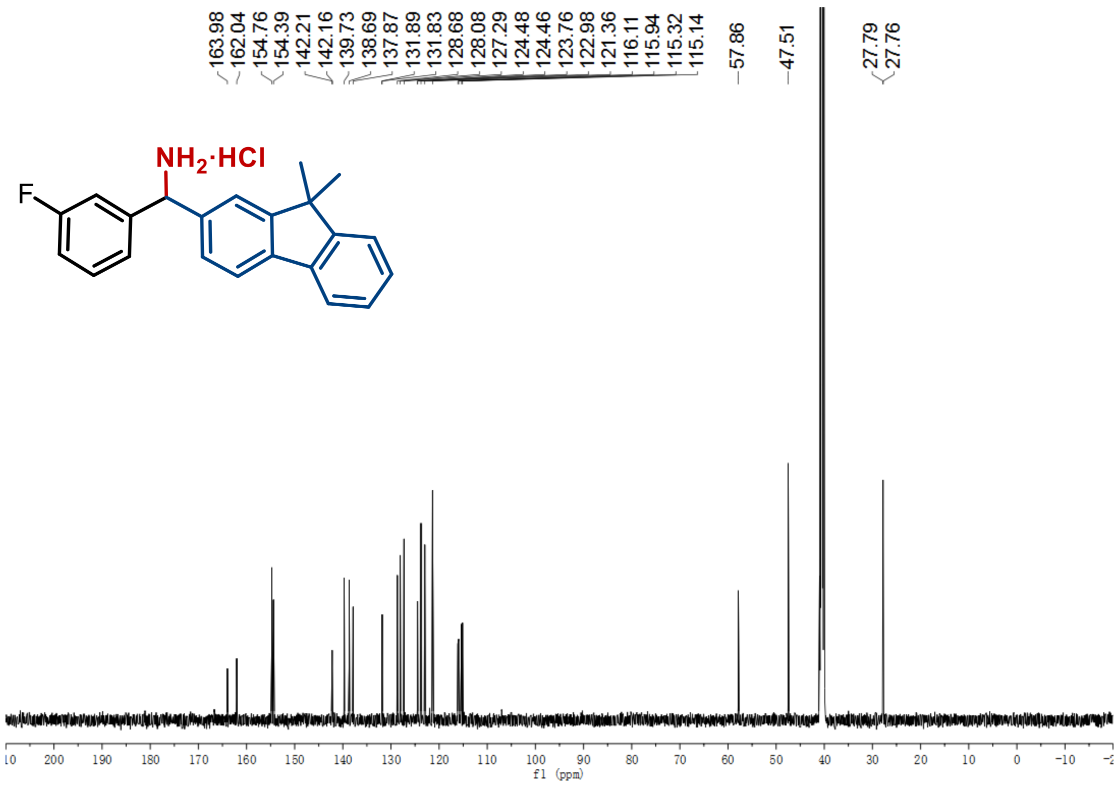

Supplementary Fig. 134 ^{13}C NMR (126 MHz, DMSO) spectrum of compound **60**.

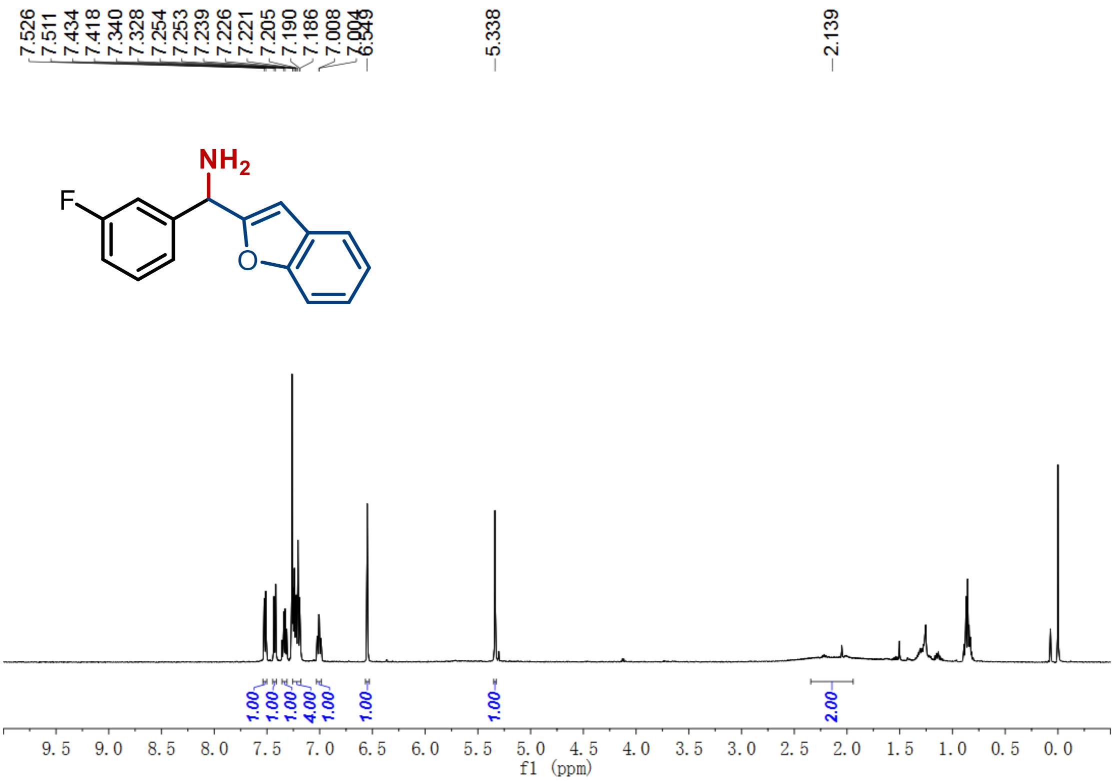

Supplementary Fig. 135 ^{1}H NMR (500 MHz, $CDCl_3$) spectrum of compound **61**.

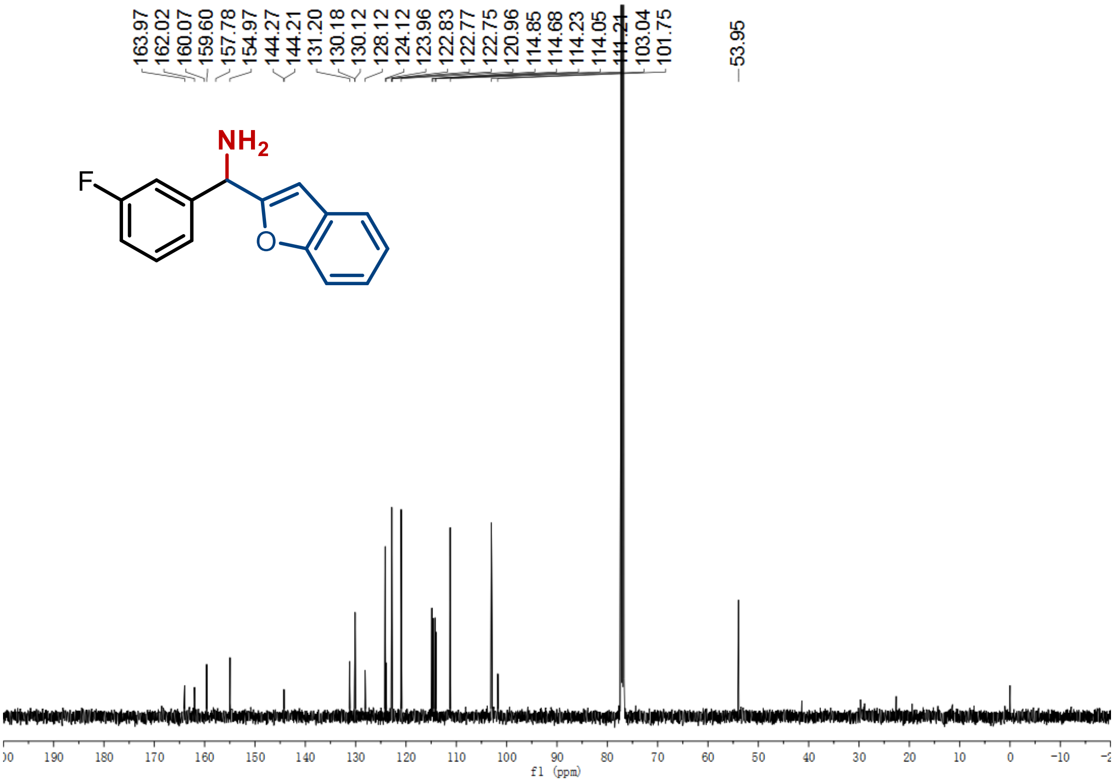

Supplementary Fig. 136 ^{13}C NMR (126 MHz, $CDCl_3$) spectrum of compound **61**.

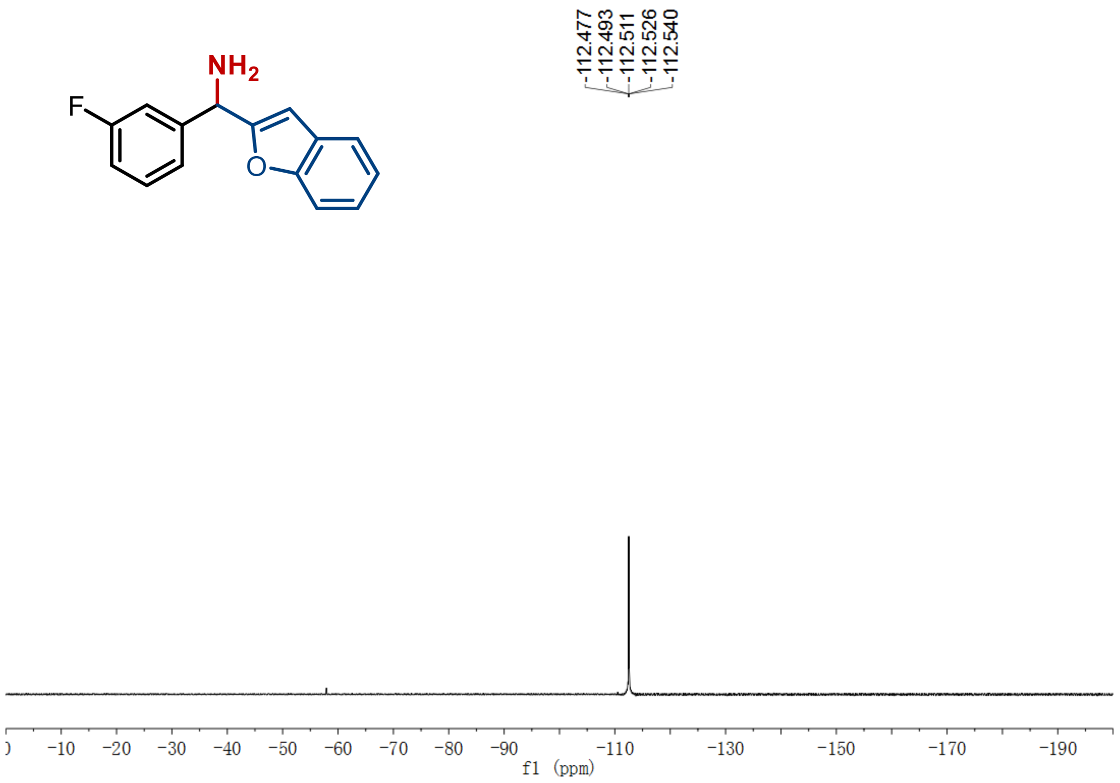

Supplementary Fig. 137 ^{19}F NMR (565 MHz, $CDCl_3$) spectrum of compound **61**.

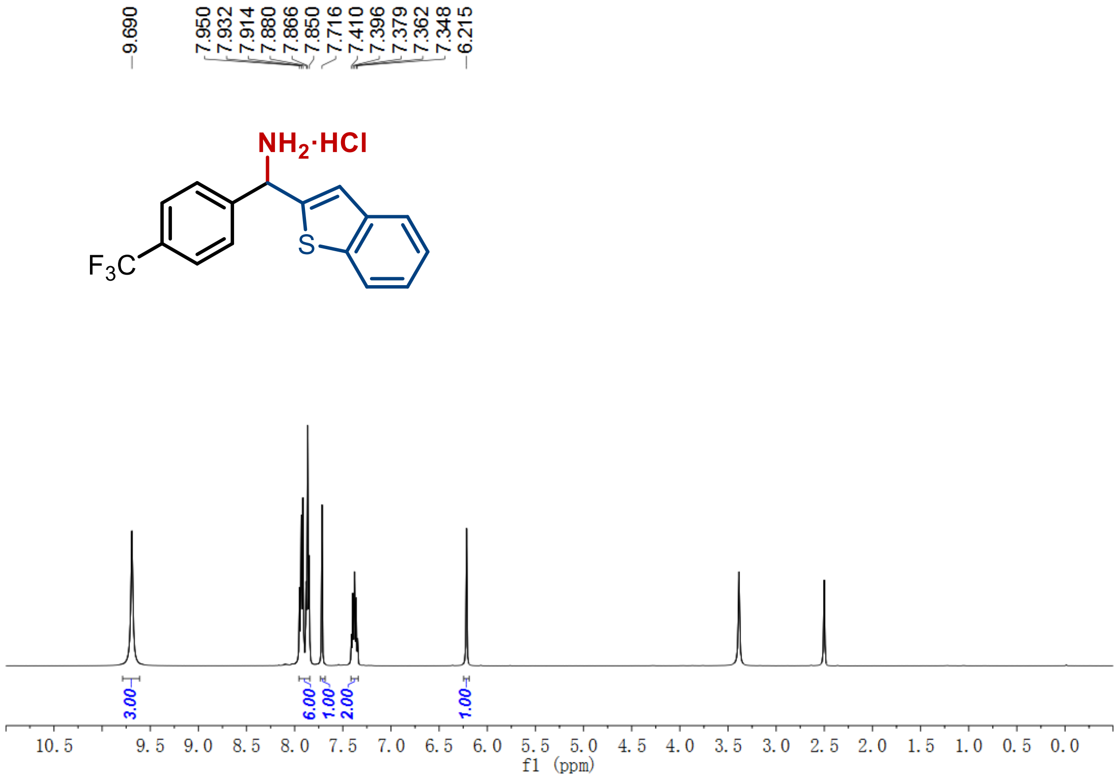

Supplementary Fig. 138 ^{1}H NMR (500 MHz, DMSO) spectrum of compound **62**.

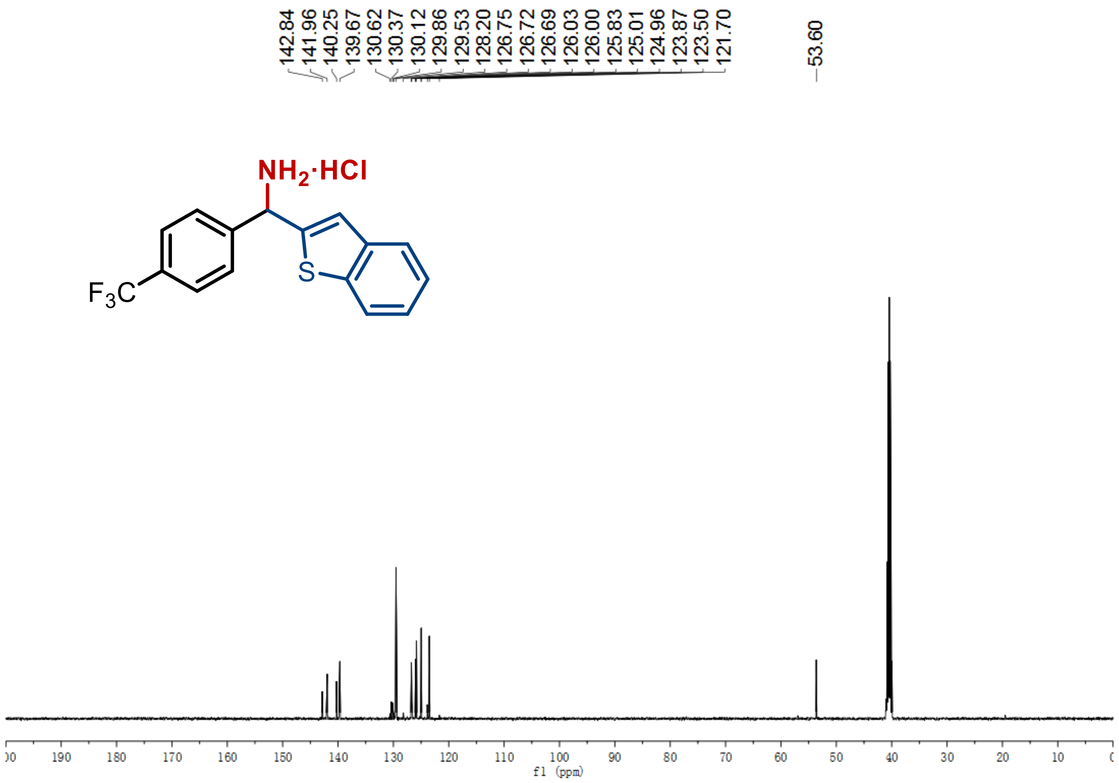

Supplementary Fig. 139 ^{13}C NMR (126 MHz, DMSO) spectrum of compound **62**.

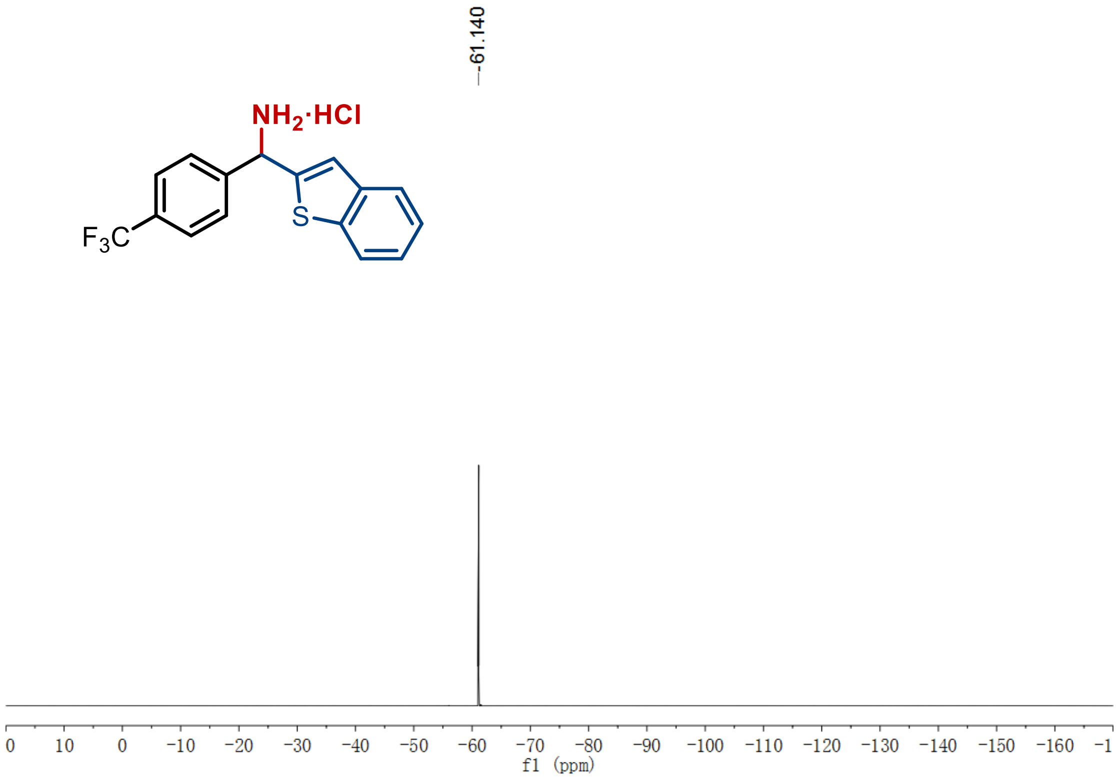

Supplementary Fig. 140 ^{19}F NMR (471 MHz, DMSO) spectrum of compound **62**.

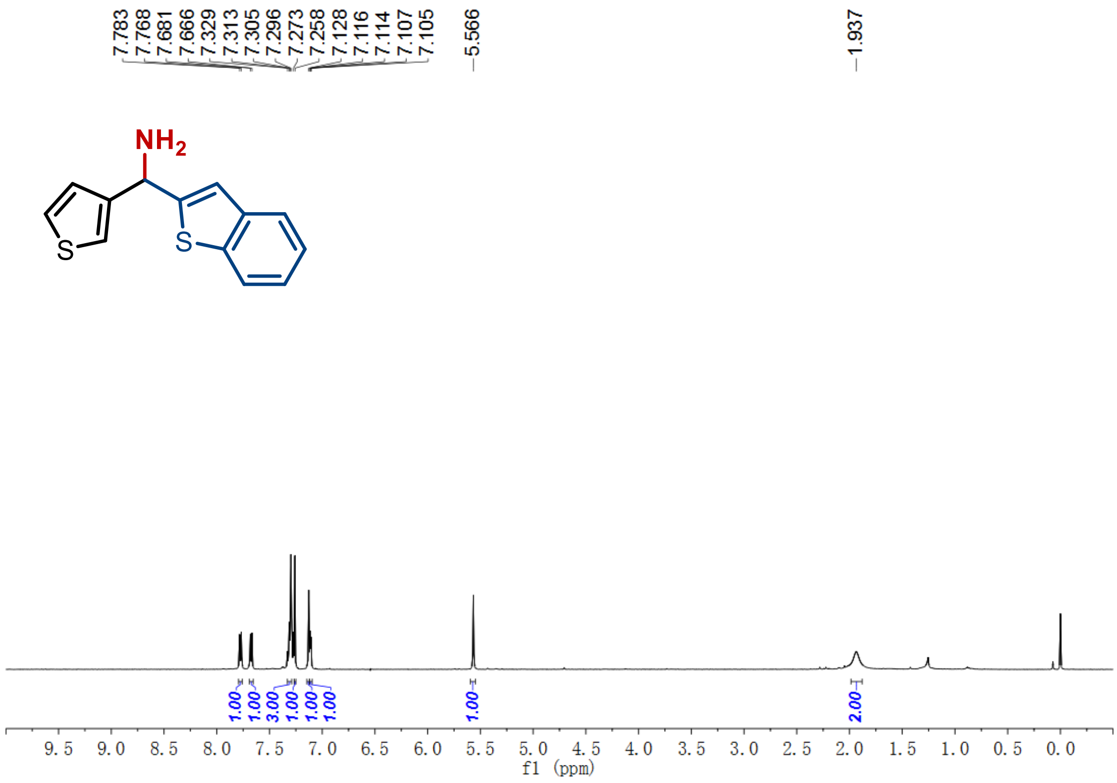

Supplementary Fig. 141 ^{1}H NMR (500 MHz, $CDCl_3$) spectrum of compound **63**.

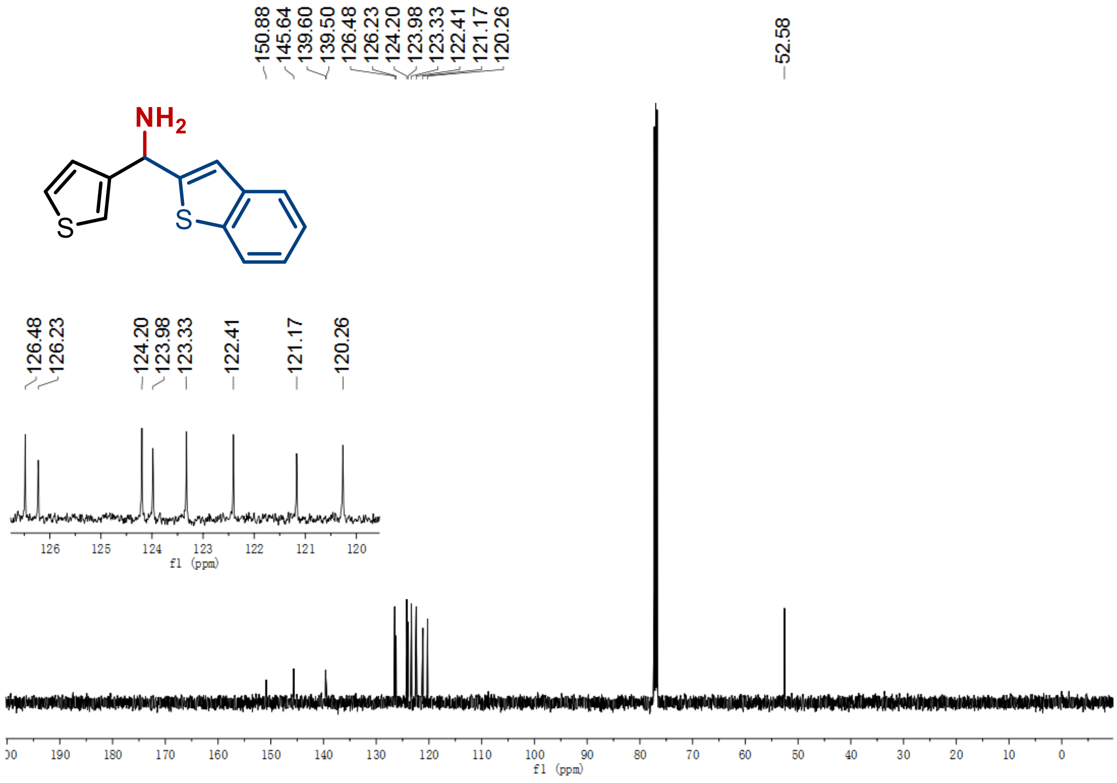

Supplementary Fig. 142 ^{13}C NMR (126 MHz, $CDCl_3$) spectrum of compound **63**.

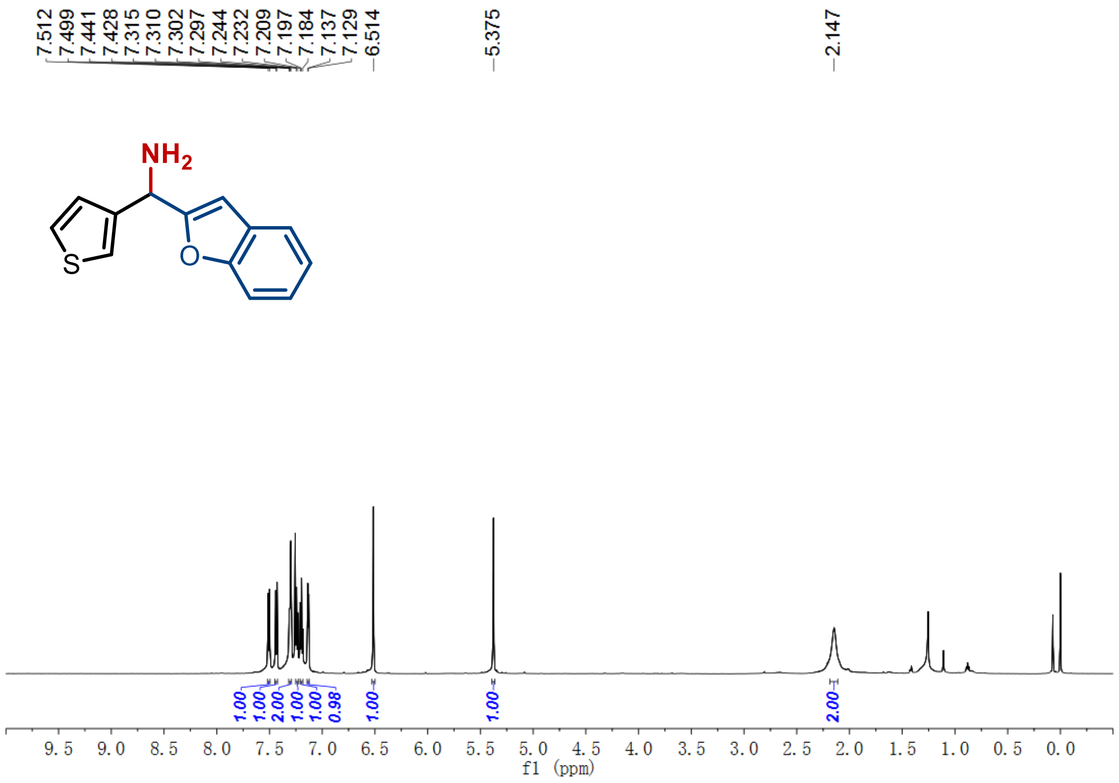

Supplementary Fig. 143 ^{1}H NMR (600 MHz, $CDCl_3$) spectrum of compound **64**.

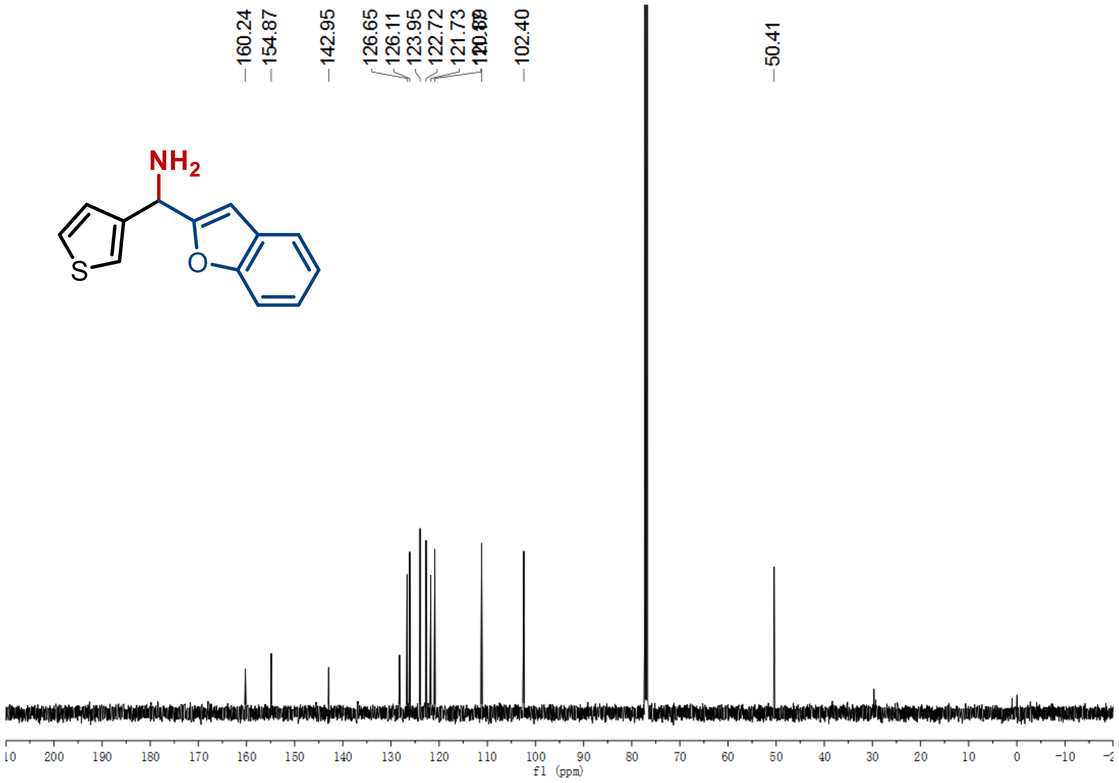

Supplementary Fig. 144 ^{13}C NMR (126 MHz, $CDCl_3$) spectrum of compound **64**.

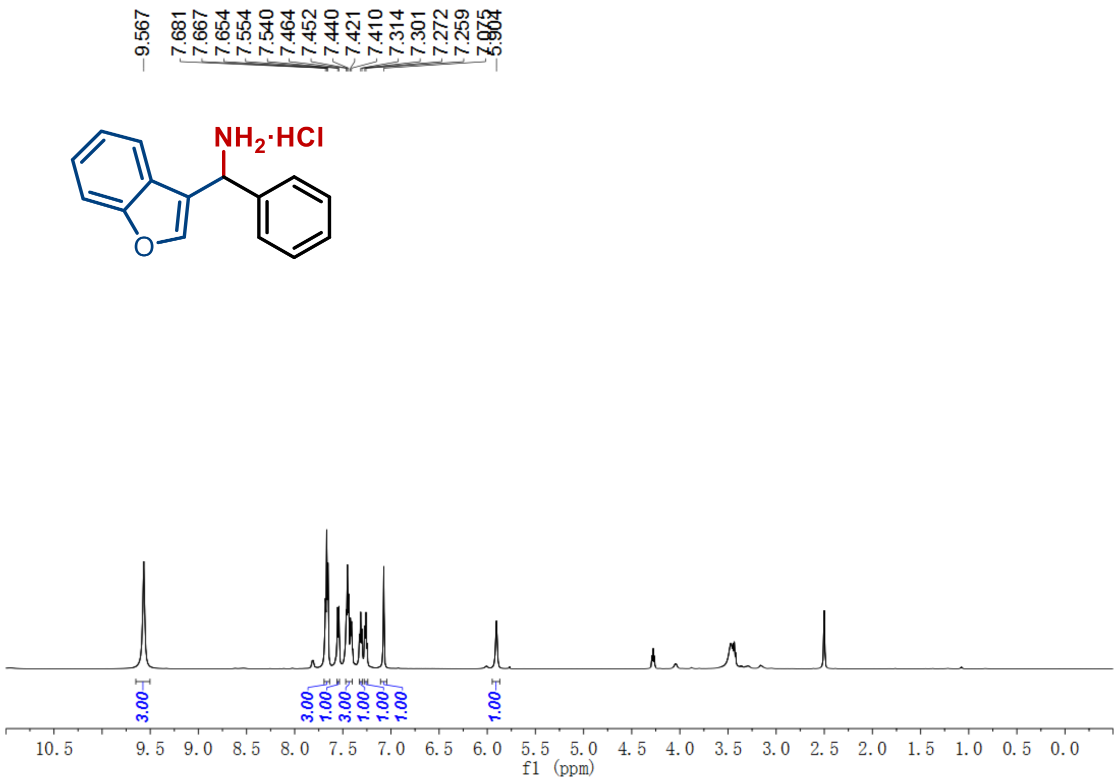

Supplementary Fig. 145 ^{1}H NMR (600 MHz, DMSO) spectrum of compound **65**.

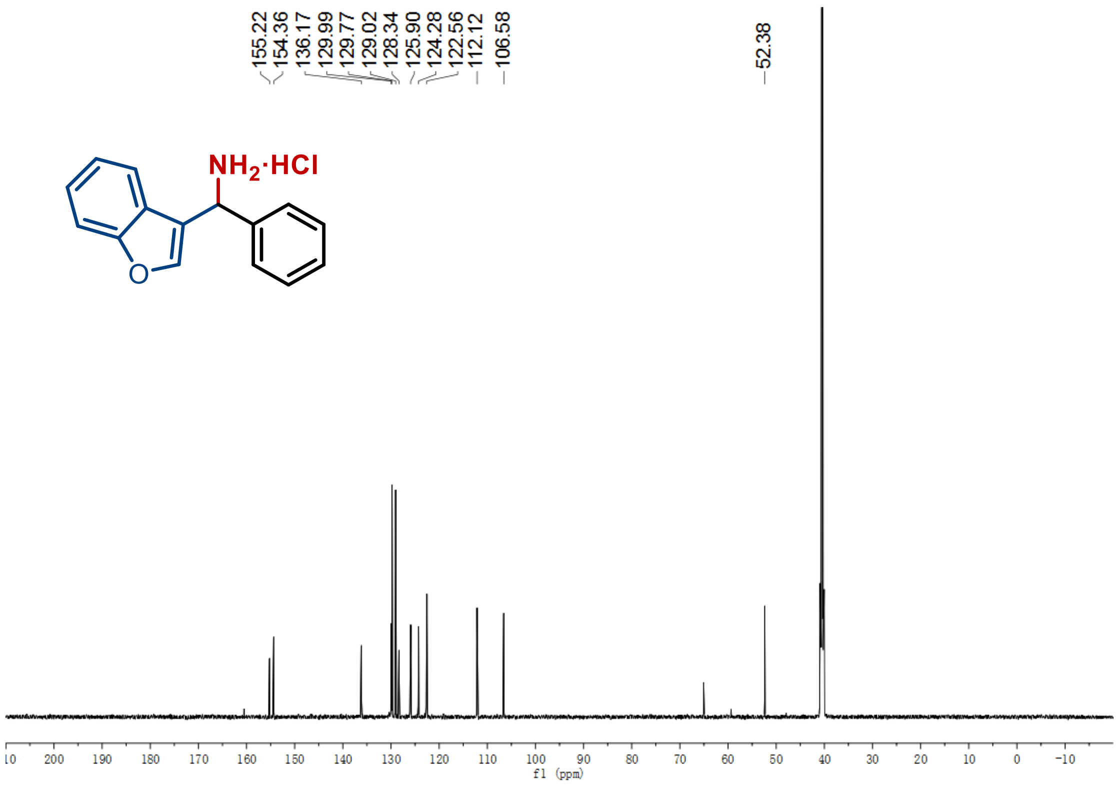

Supplementary Fig. 146 ^{13}C NMR (151 MHz, DMSO) spectrum of compound **65**.

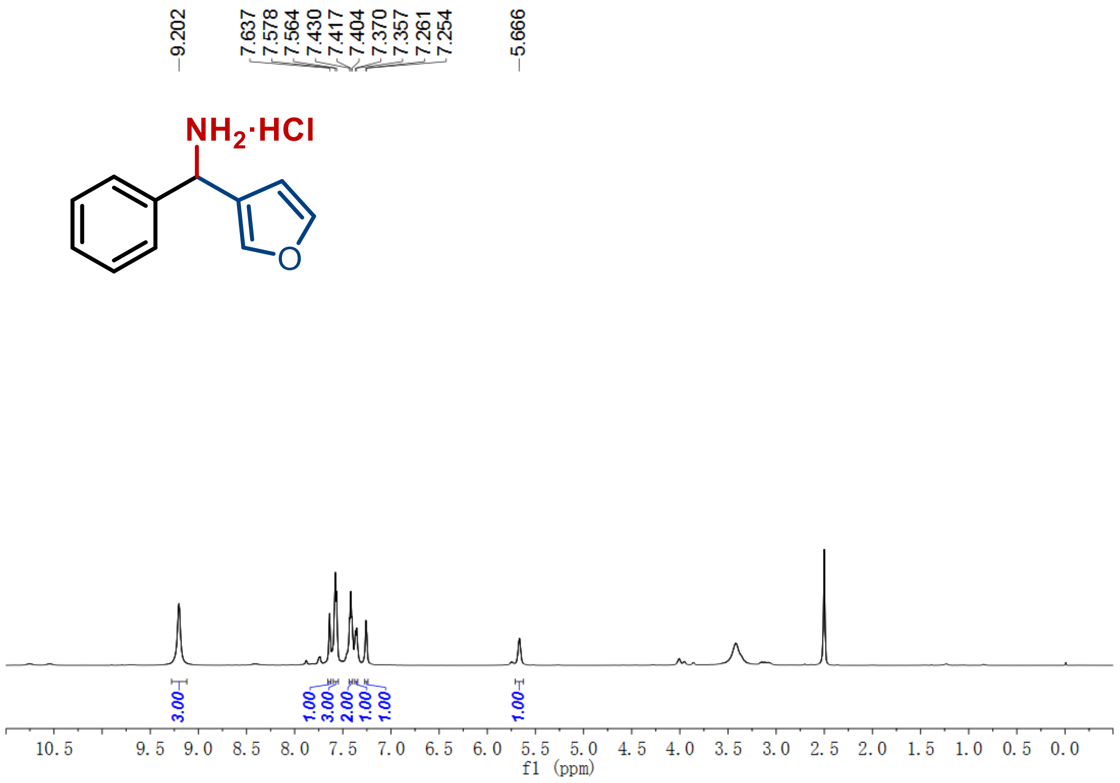

Supplementary Fig. 147 ^{1}H NMR (500 MHz, DMSO) spectrum of compound **66**.

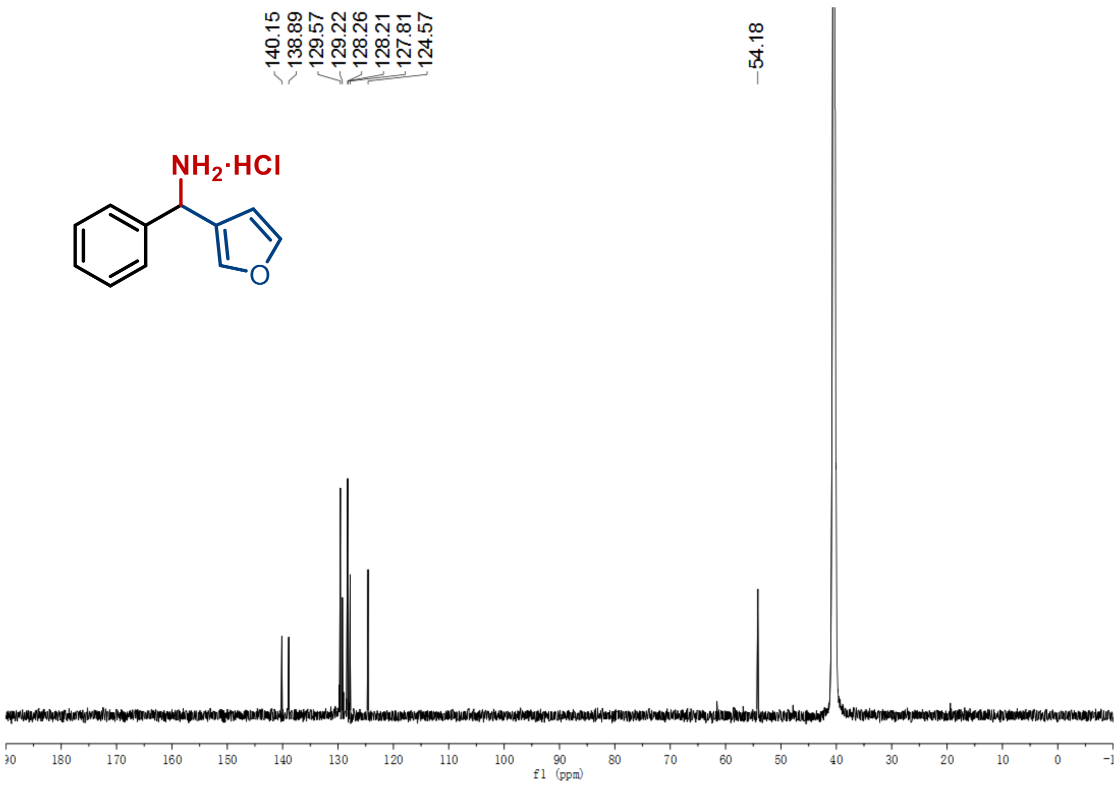

Supplementary Fig. 148 ^{13}C NMR (151 MHz, DMSO) spectrum of compound **66**.

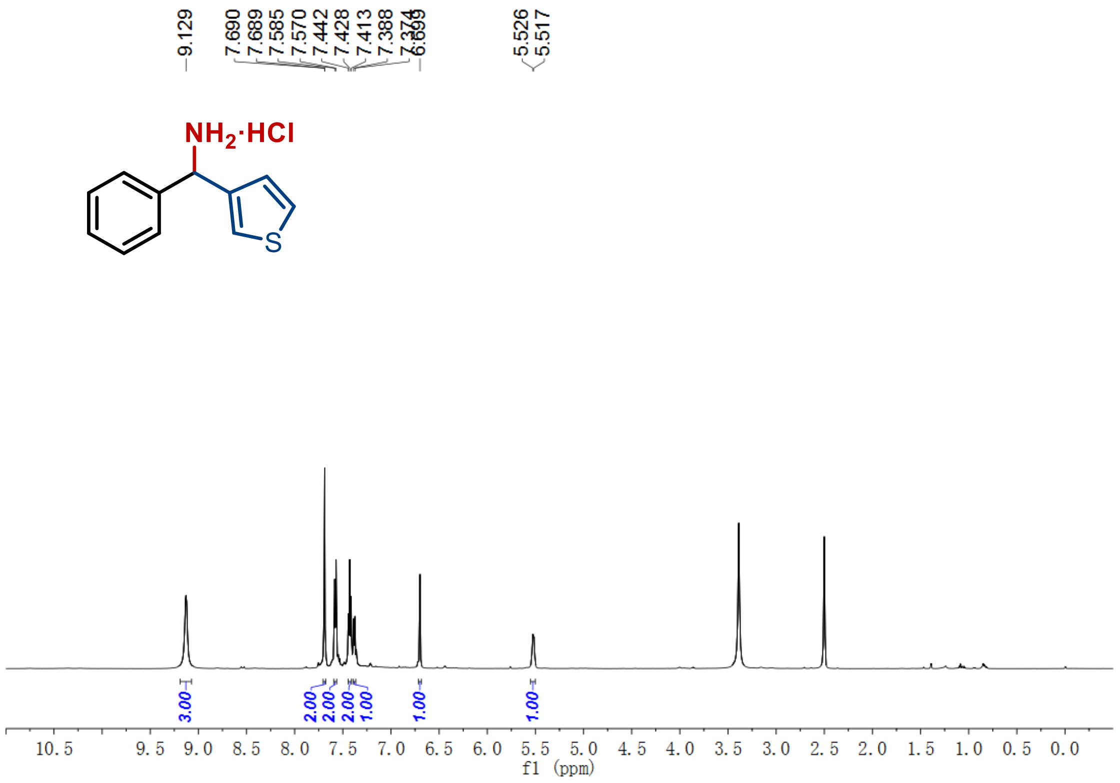

Supplementary Fig. 149 ^{1}H NMR (500 MHz, DMSO) spectrum of compound **67**.

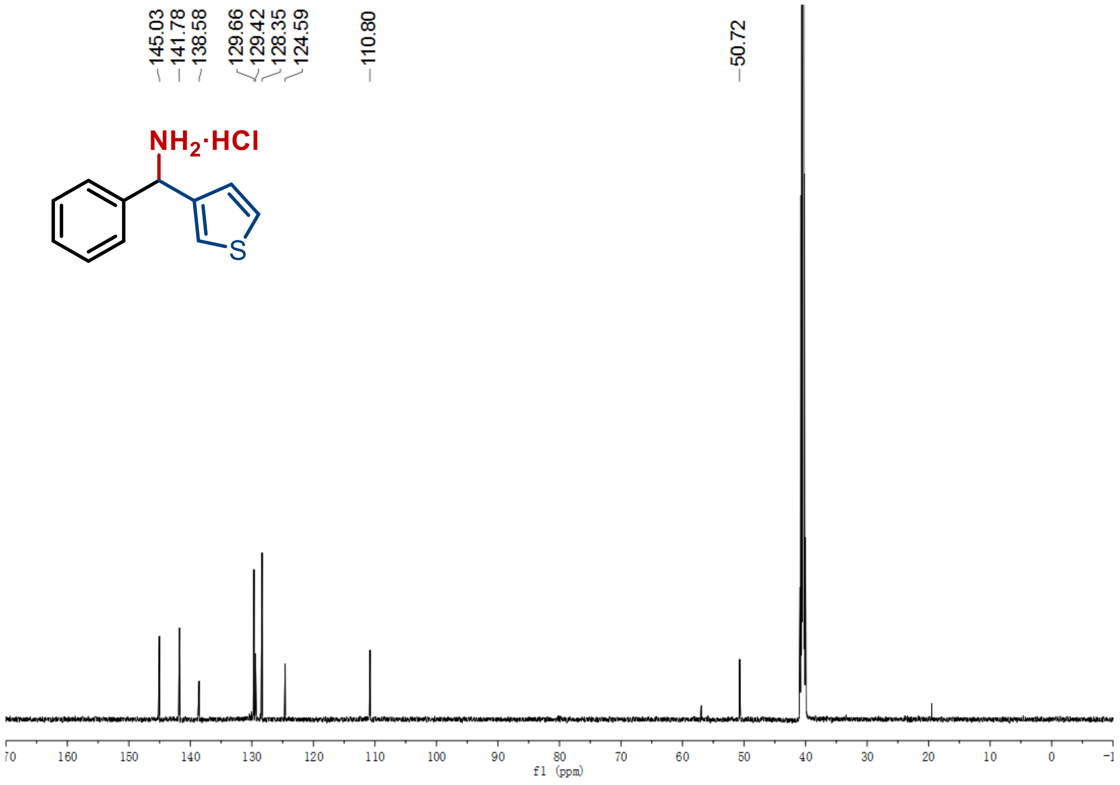

Supplementary Fig. 150 ^{13}C NMR (151 MHz, DMSO) spectrum of compound **67**.

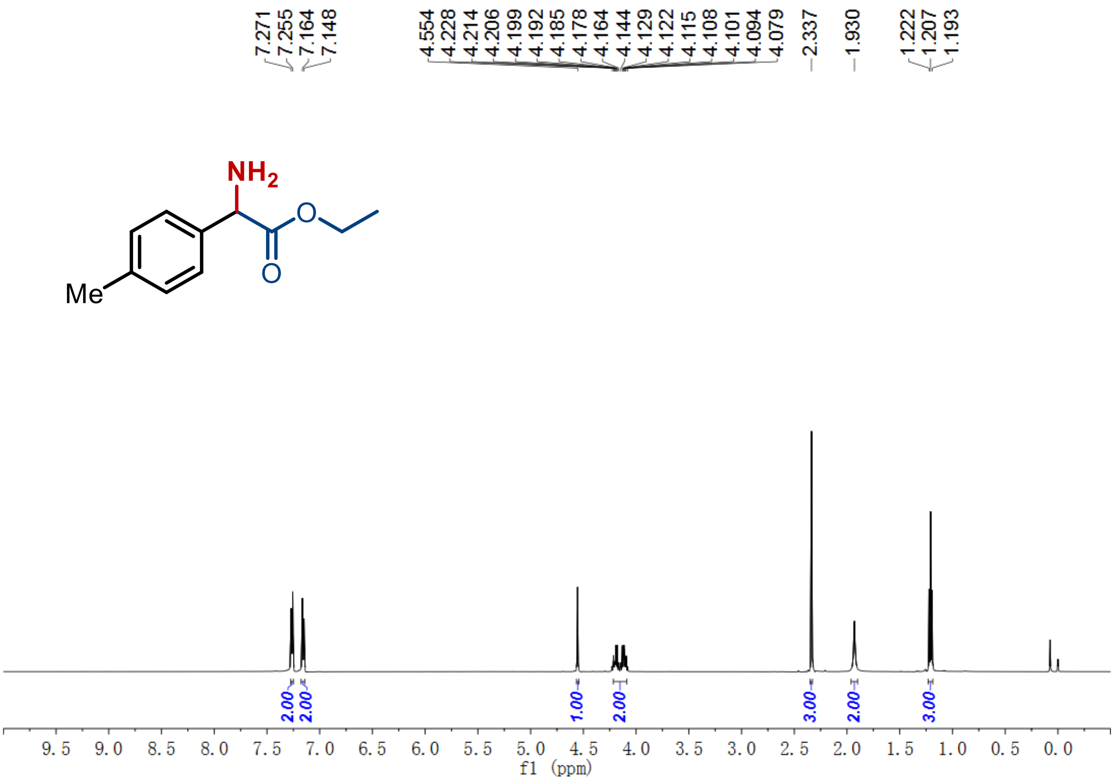

Supplementary Fig. 151 ^{1}H NMR (500 MHz, $CDCl_3$) spectrum of compound **68**.

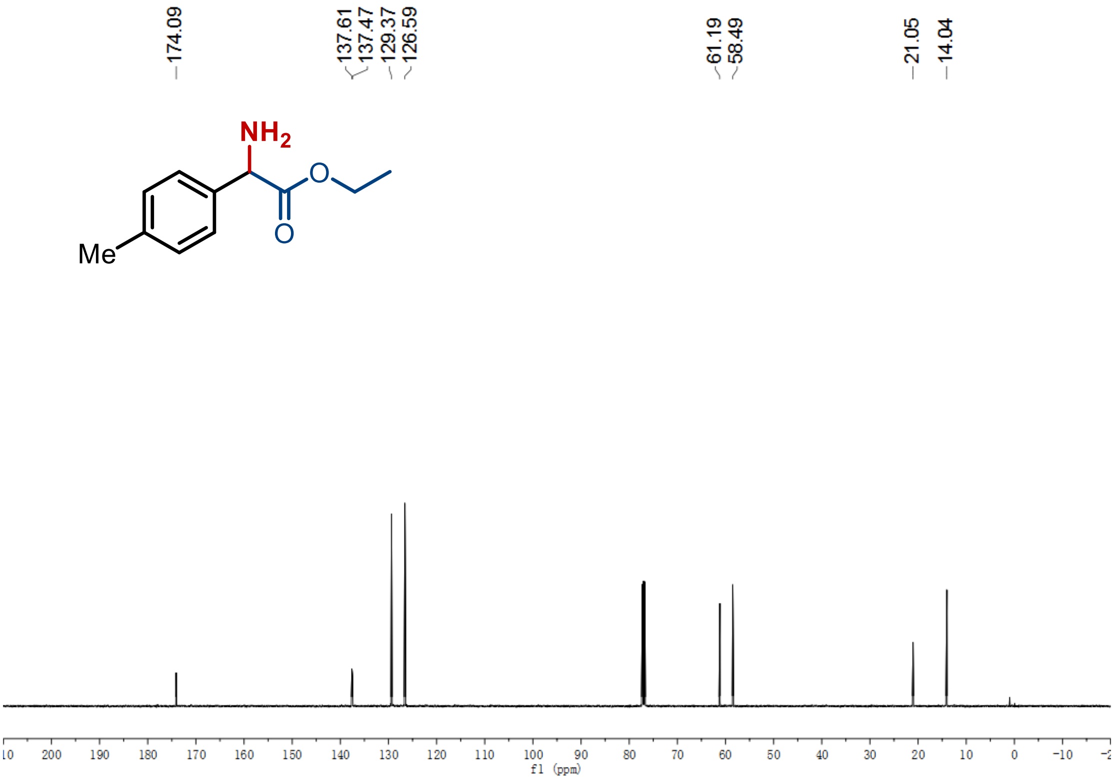

Supplementary Fig. 152 ^{13}C NMR (126 MHz, $CDCl_3$) spectrum of compound **68**.

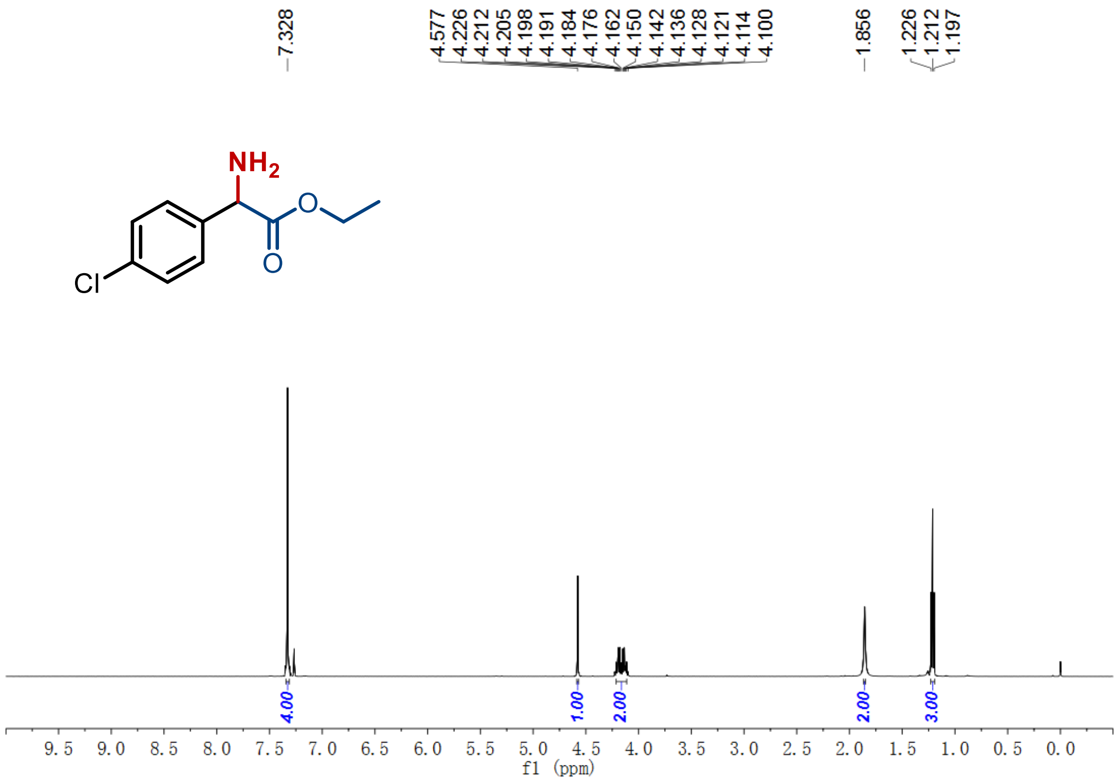

Supplementary Fig. 153 ^{1}H NMR (500 MHz, $CDCl_3$) spectrum of compound **69**.

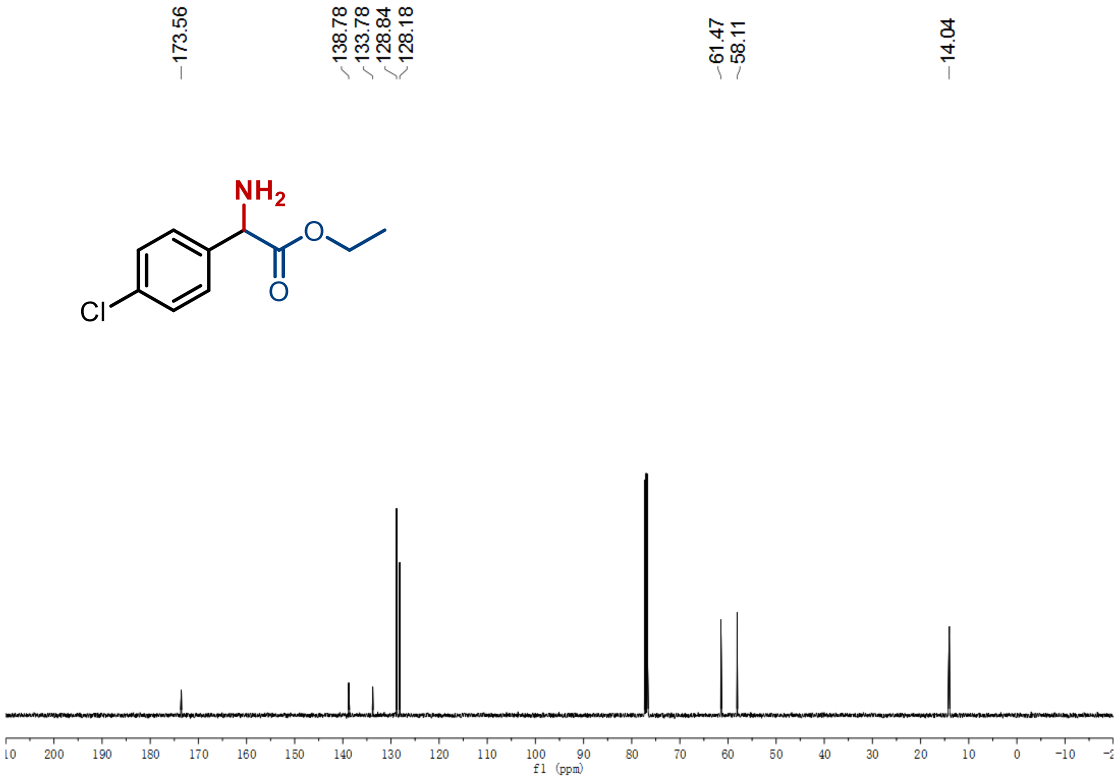

Supplementary Fig. 154 ^{13}C NMR (126 MHz, $CDCl_3$) spectrum of compound **69**.

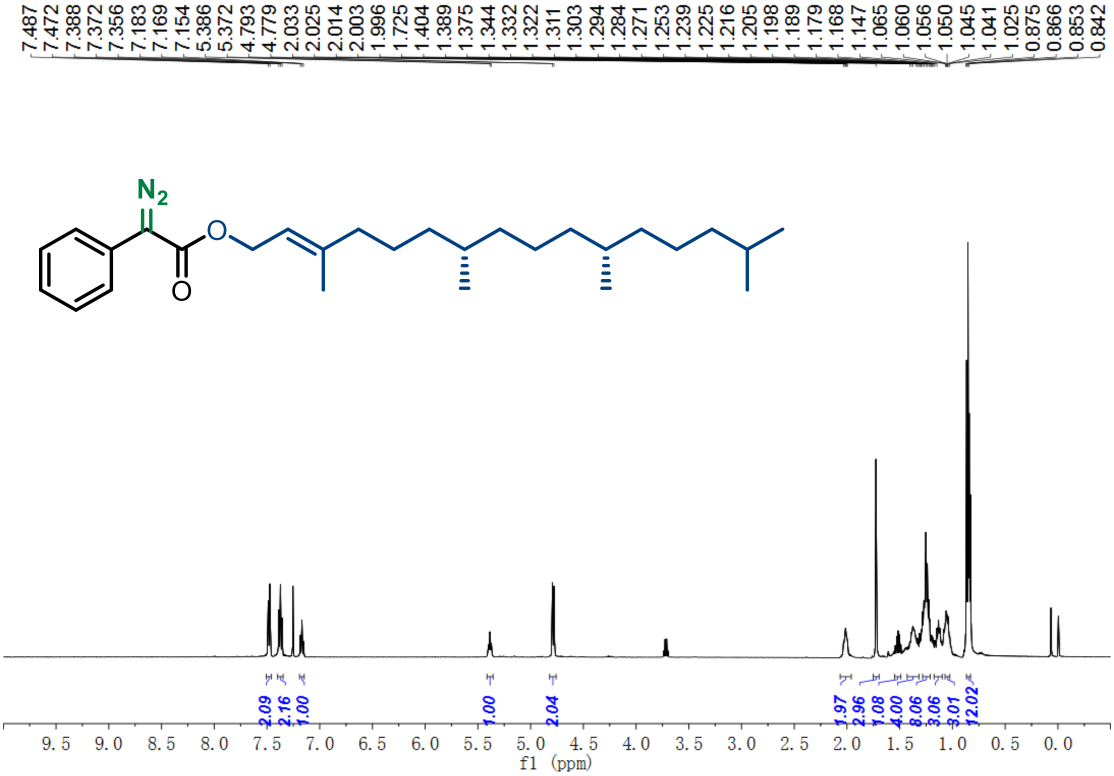

Supplementary Fig. 155 ^{1}H NMR (500 MHz, $CDCl_3$) spectrum of compound **S70**.

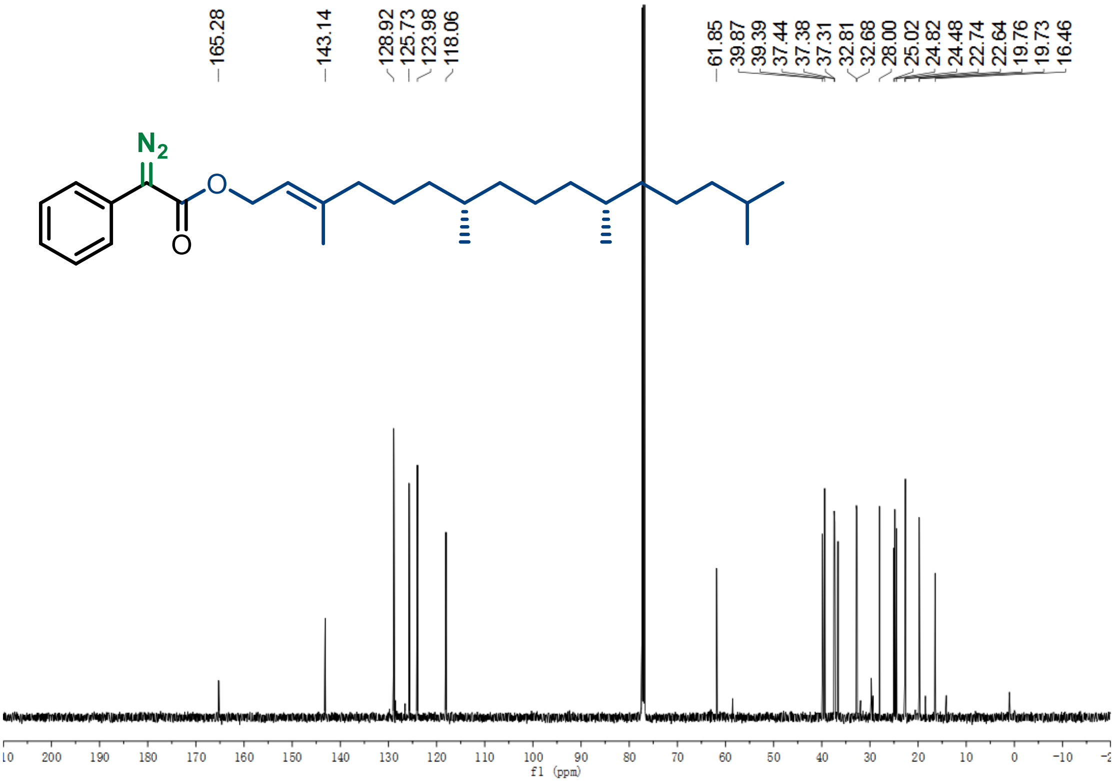

Supplementary Fig. 156 ^{13}C NMR (126 MHz, $CDCl_3$) spectrum of compound **S70**.

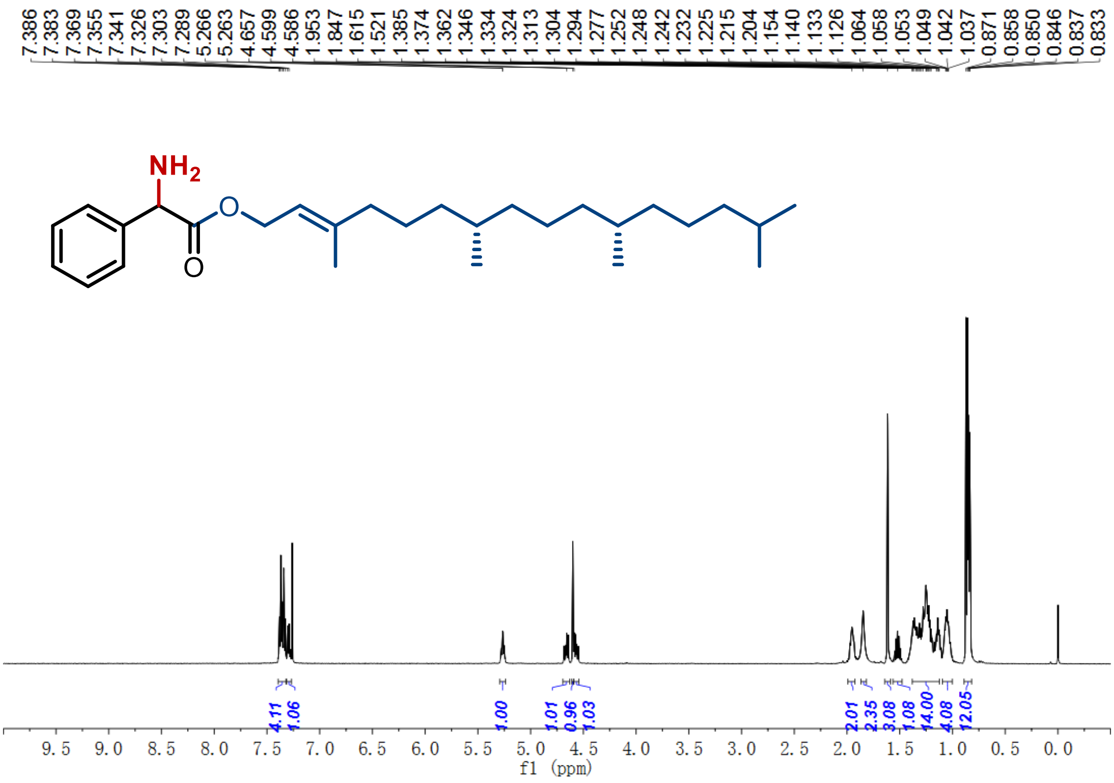

Supplementary Fig. 157 ^{1}H NMR (500 MHz, $CDCl_3$) spectrum of compound **70**.

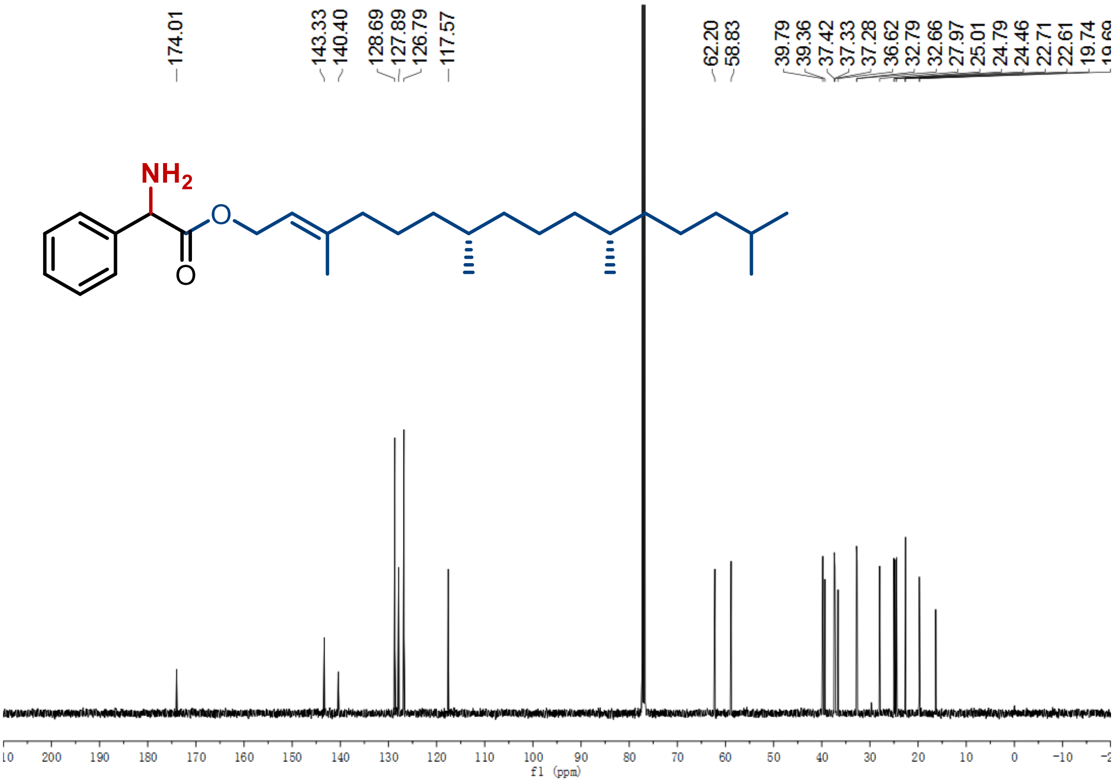

Supplementary Fig. 158 ^{13}C NMR (126 MHz, $CDCl_3$) spectrum of compound **70**.

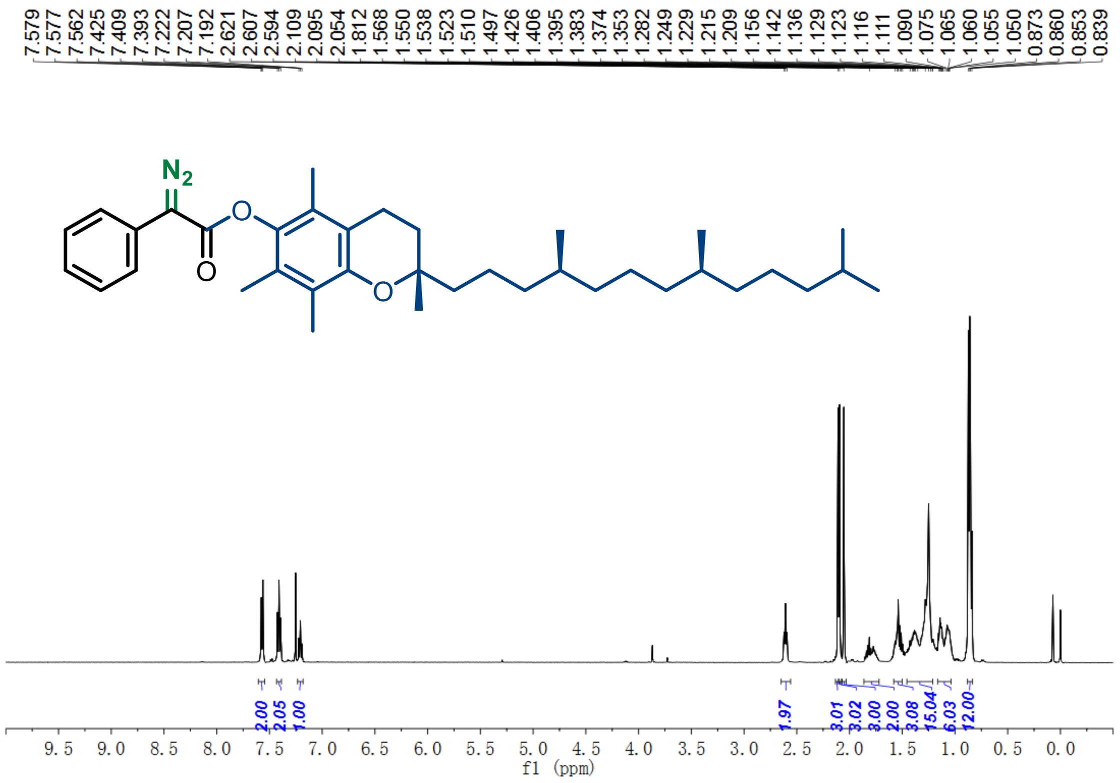

Supplementary Fig. 159 ^{1}H NMR (500 MHz, $CDCl_3$) spectrum of compound **S71**.

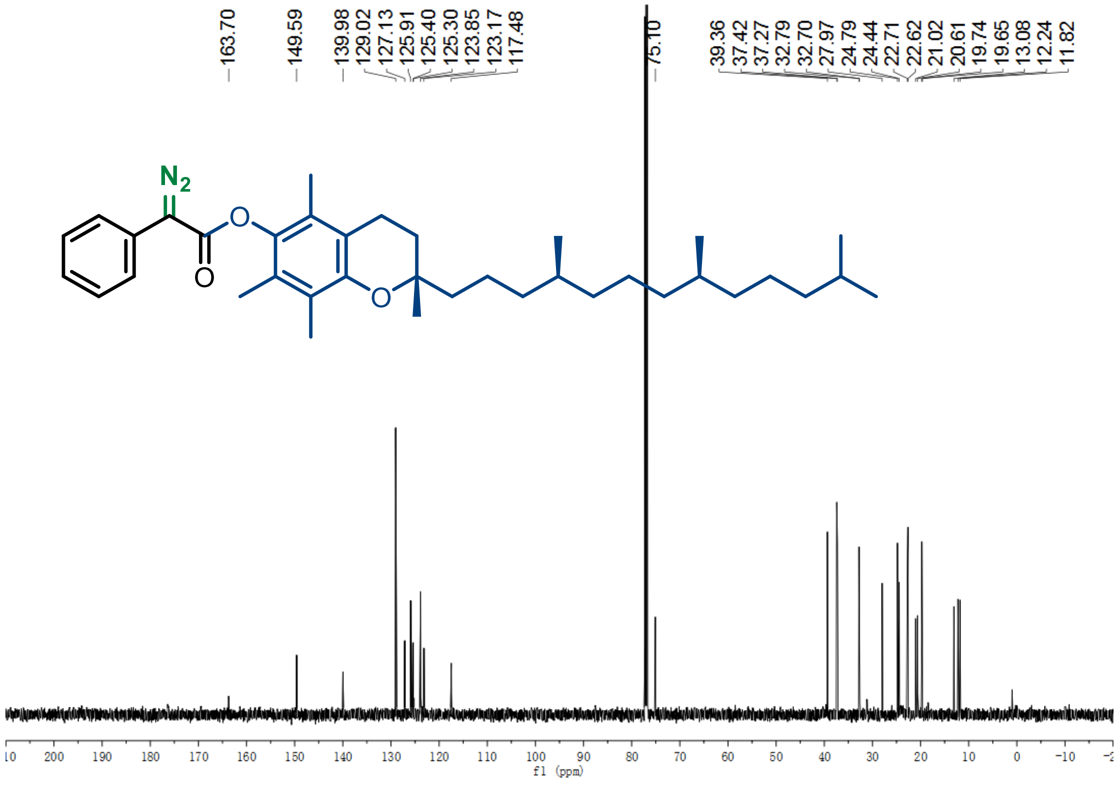

Supplementary Fig. 160 ^{13}C NMR (126 MHz, $CDCl_3$) spectrum of compound **S71**.

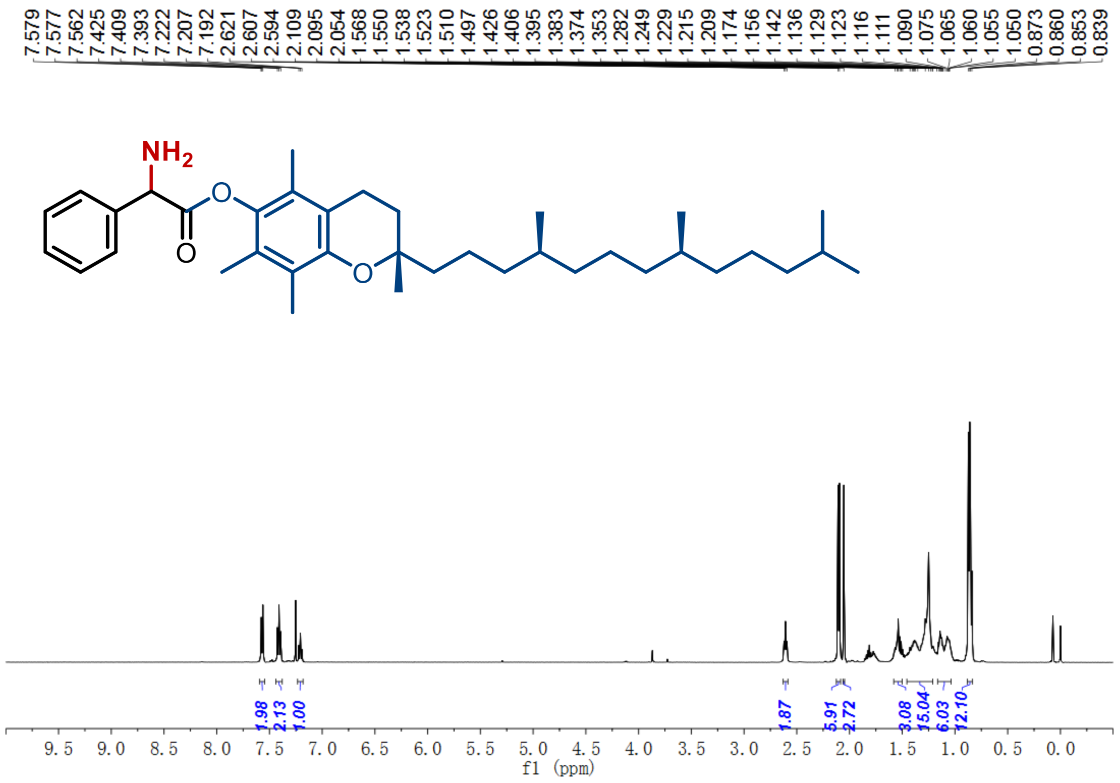

Supplementary Fig. 161 ^{1}H NMR (500 MHz, $CDCl_3$) spectrum of compound **71**.

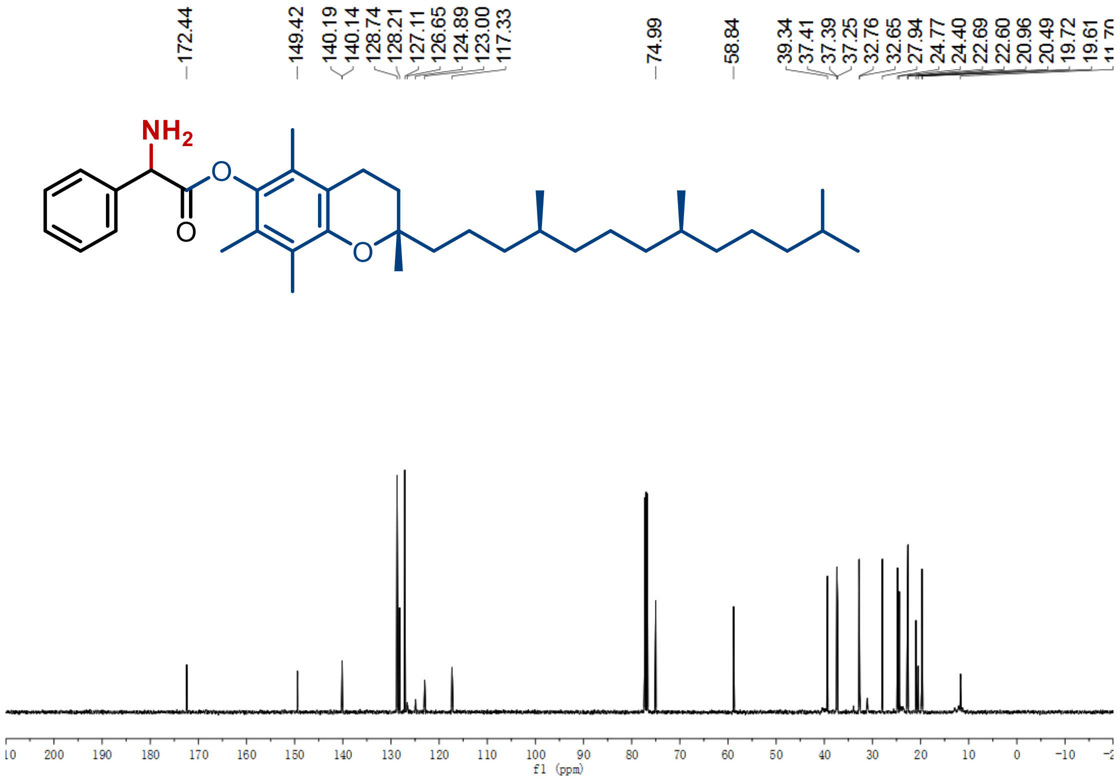

Supplementary Fig. 162 ^{13}C NMR (126 MHz, $CDCl_3$) spectrum of compound **71**.

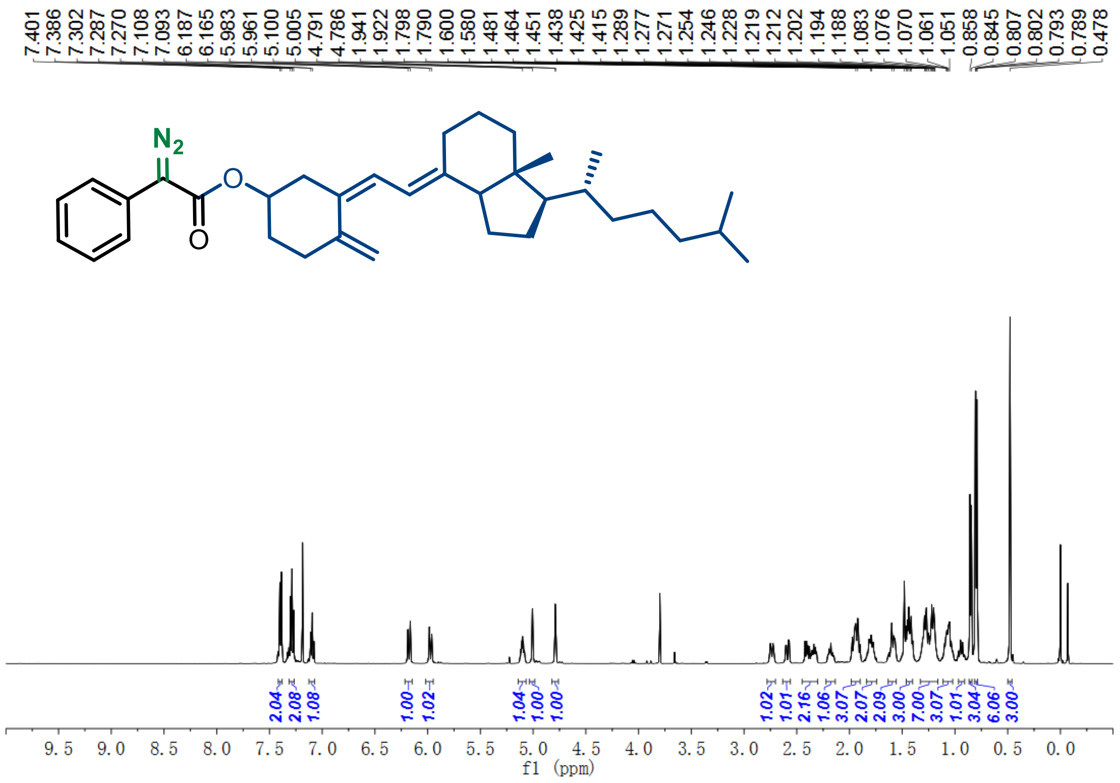

Supplementary Fig. 163 ^{1}H NMR (500 MHz, $CDCl_3$) spectrum of compound **S72**.

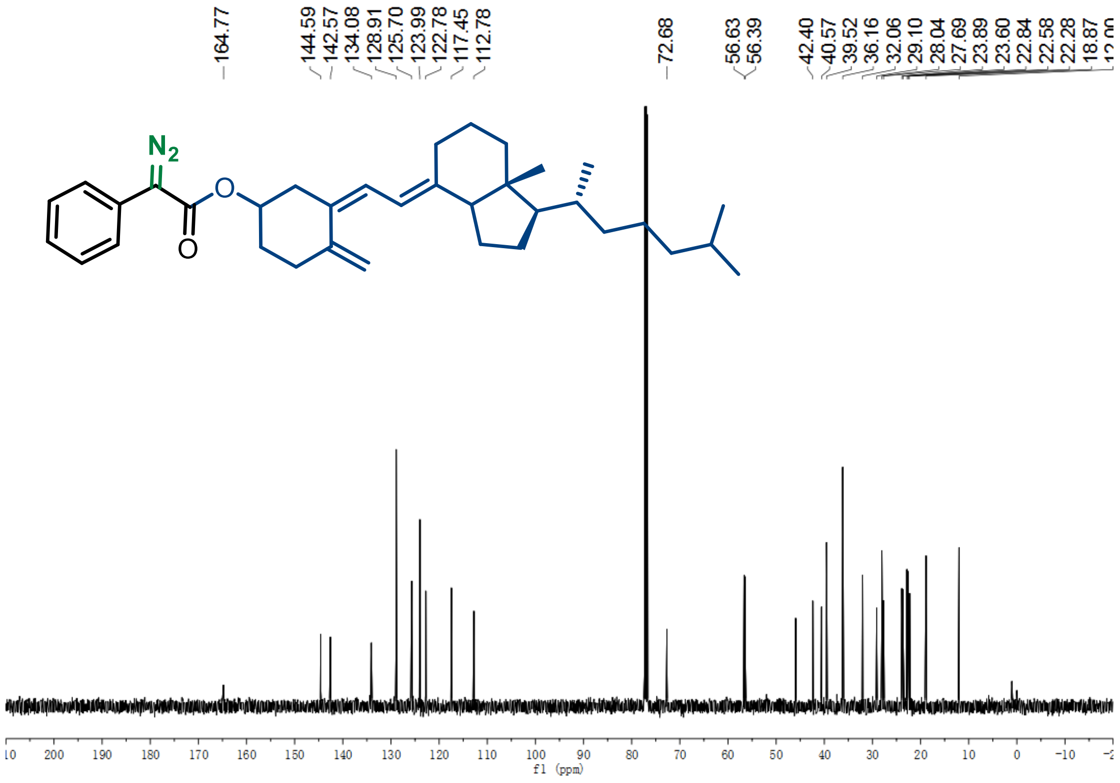

Supplementary Fig. 164 ^{13}C NMR (126 MHz, $CDCl_3$) spectrum of compound **S72**.

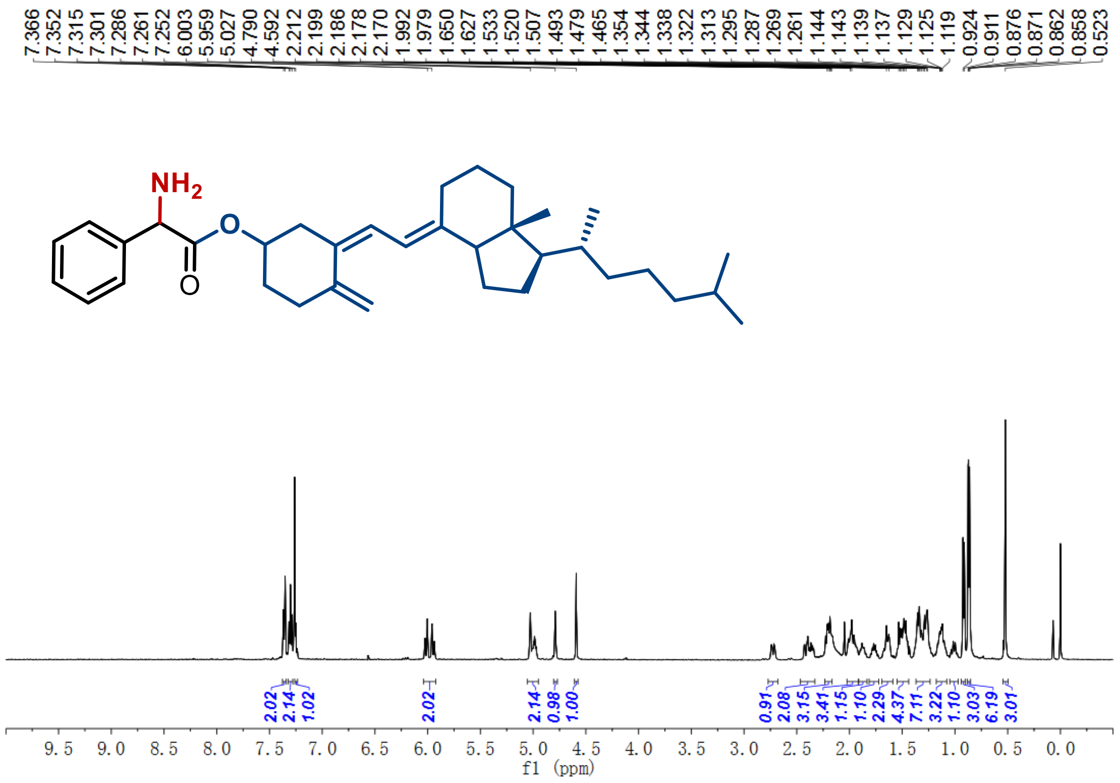

Supplementary Fig. 165 ^{1}H NMR (500 MHz, $CDCl_3$) spectrum of compound **72**.

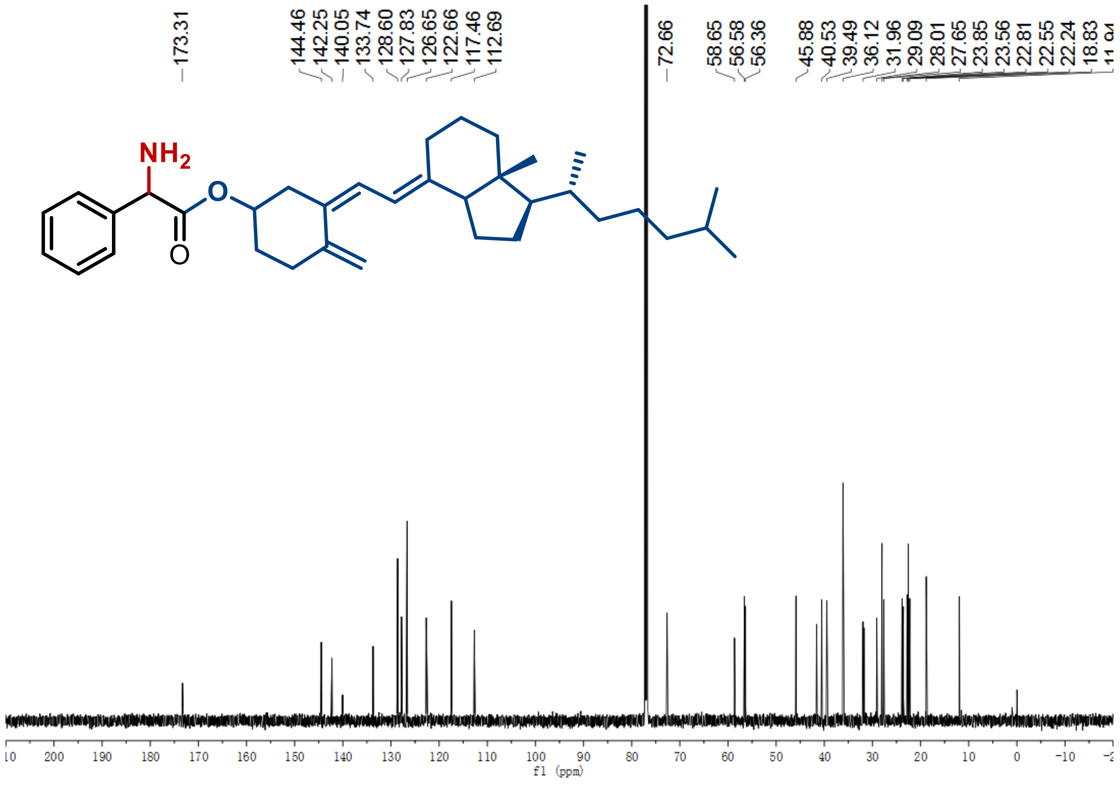

Supplementary Fig. 166 ^{13}C NMR (151 MHz, $CDCl_3$) spectrum of compound **72**.

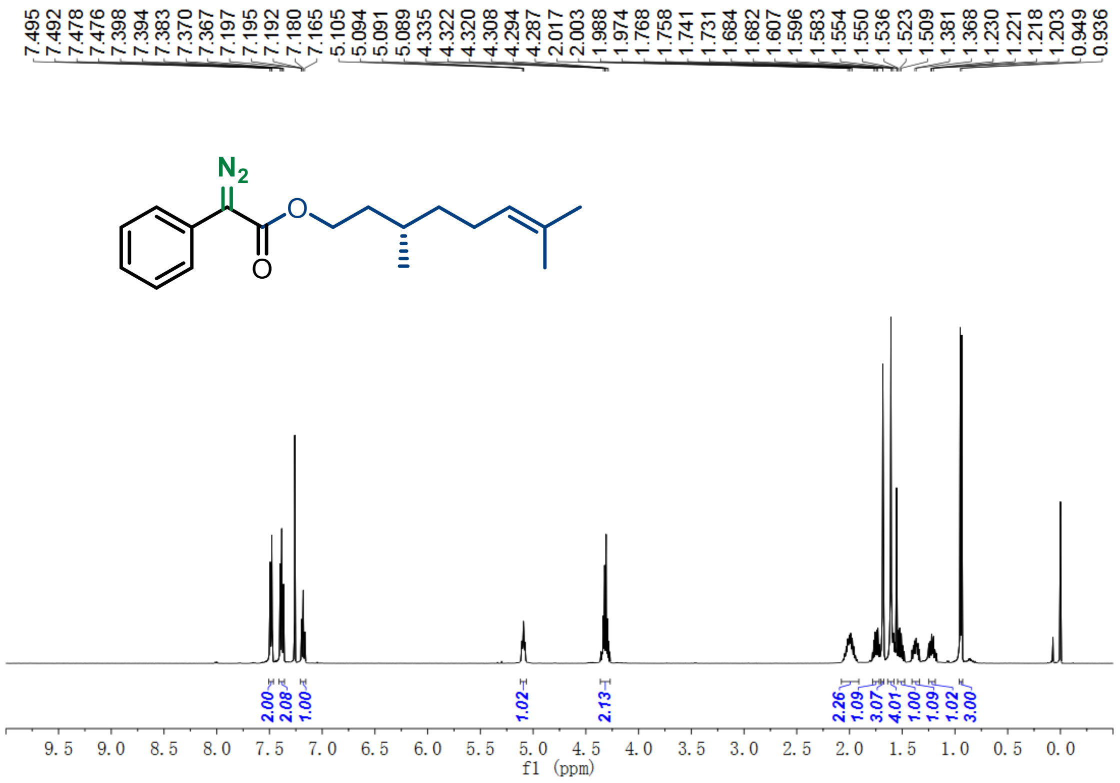

Supplementary Fig. 167 ^{1}H NMR (500 MHz, $CDCl_3$) spectrum of compound **S73**.

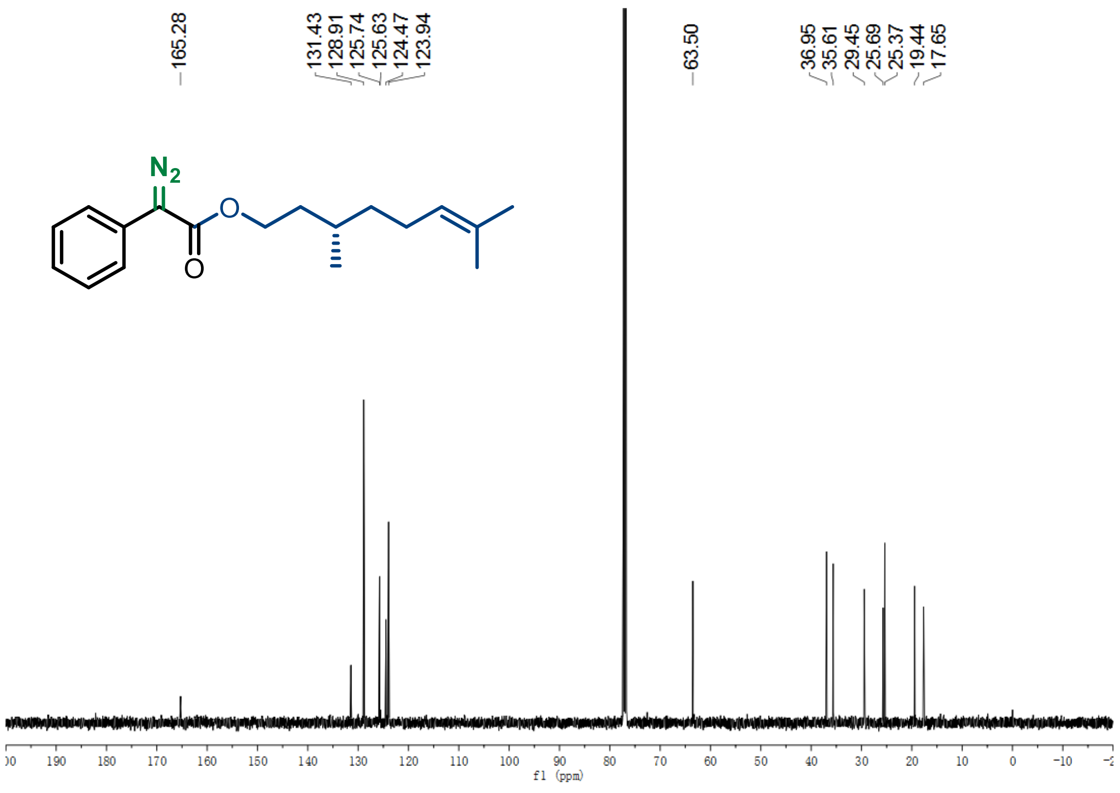

Supplementary Fig. 168 ^{13}C NMR (126 MHz, $CDCl_3$) spectrum of compound **S73**.

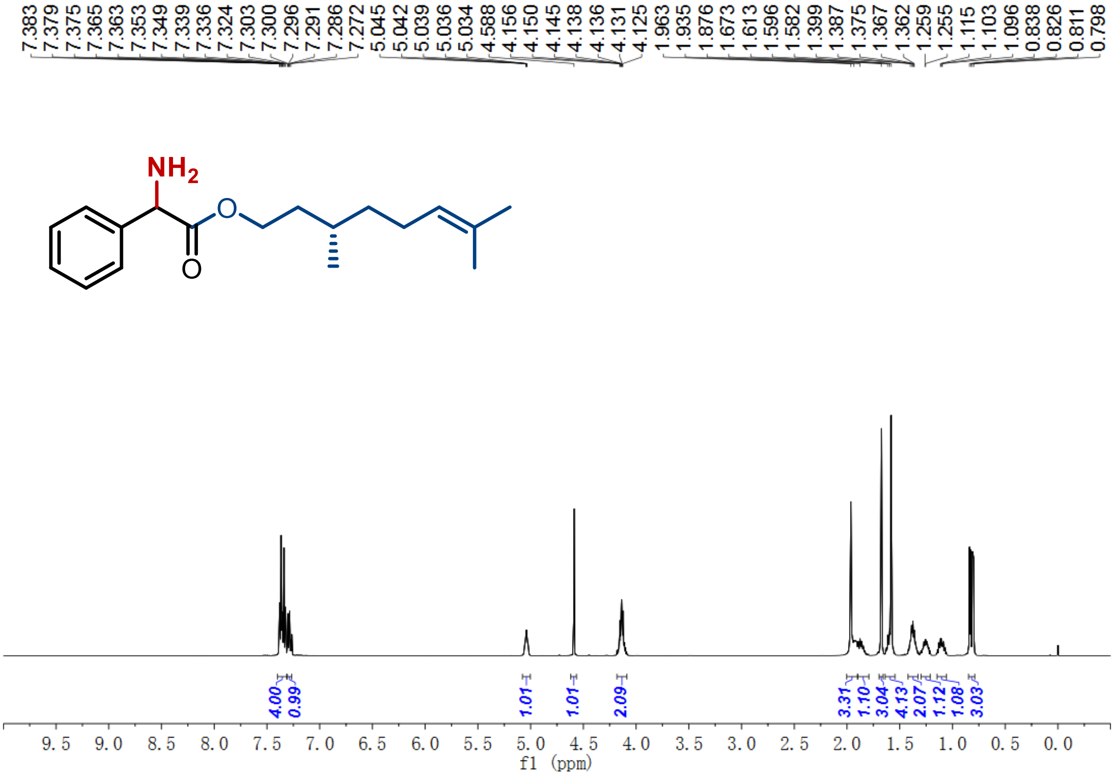

Supplementary Fig. 169 ^{1}H NMR (500 MHz, $CDCl_3$) spectrum of compound **73**.

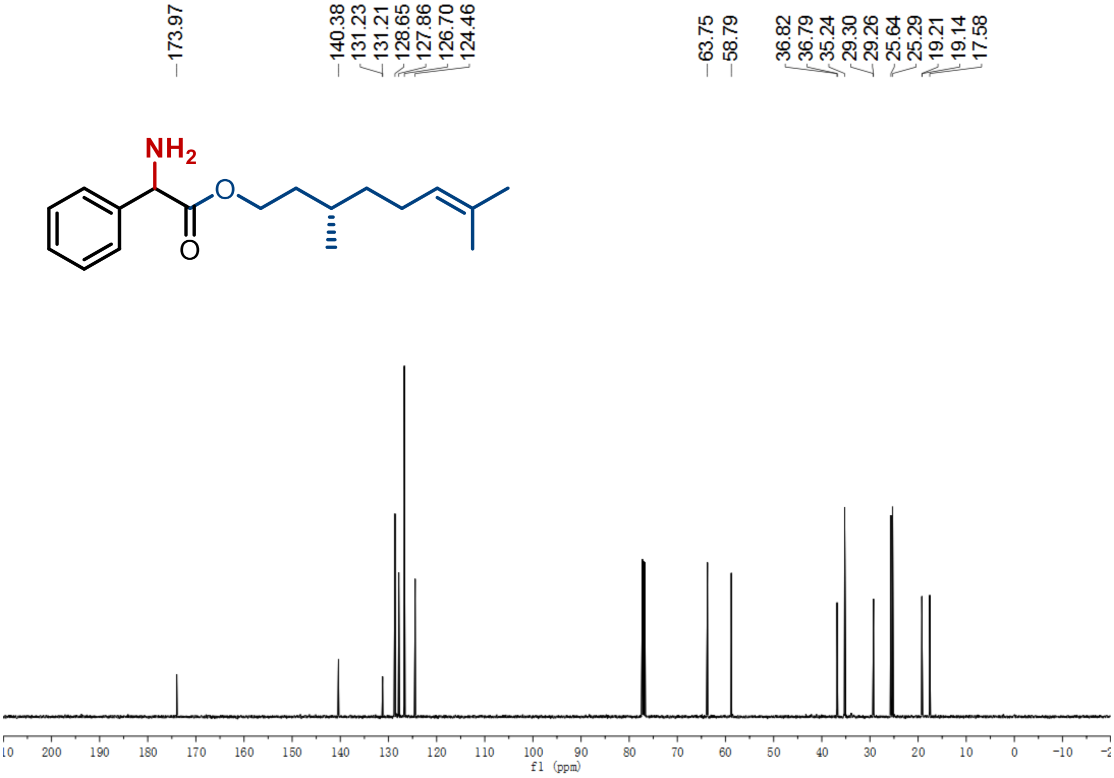

Supplementary Fig. 170 ^{13}C NMR (126 MHz, $CDCl_3$) spectrum of compound **73**.

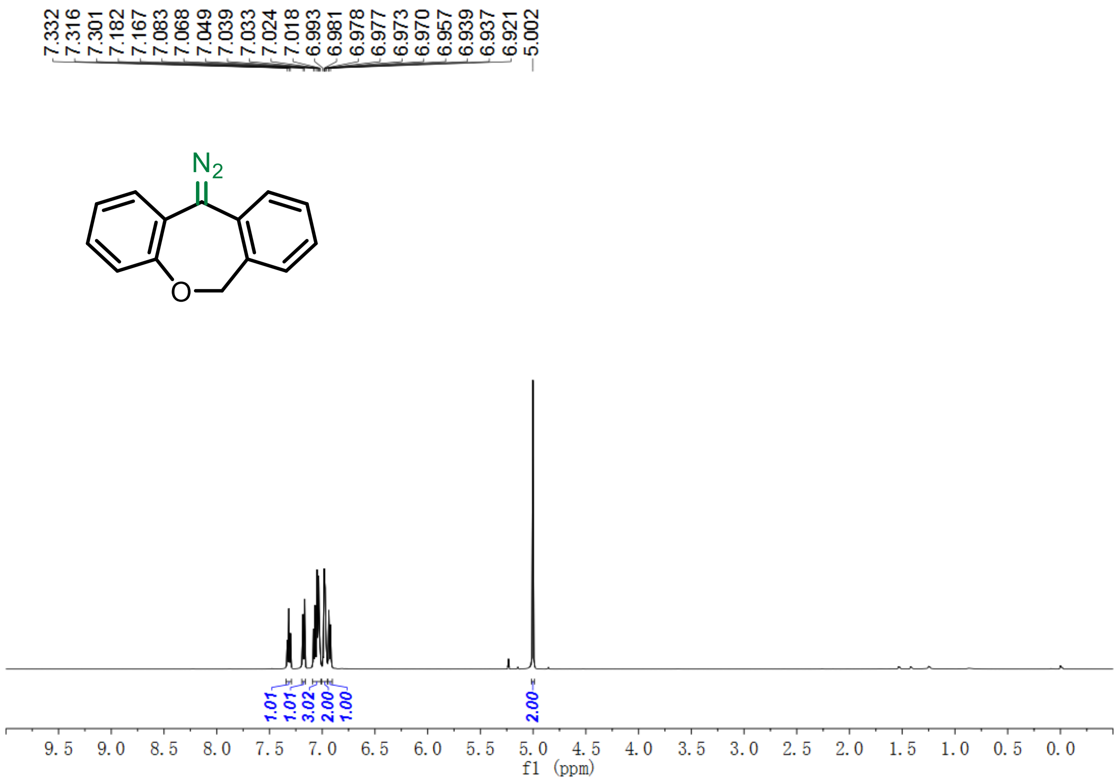

Supplementary Fig. 171 ^{1}H NMR (500 MHz, $CDCl_3$) spectrum of compound **S74**.

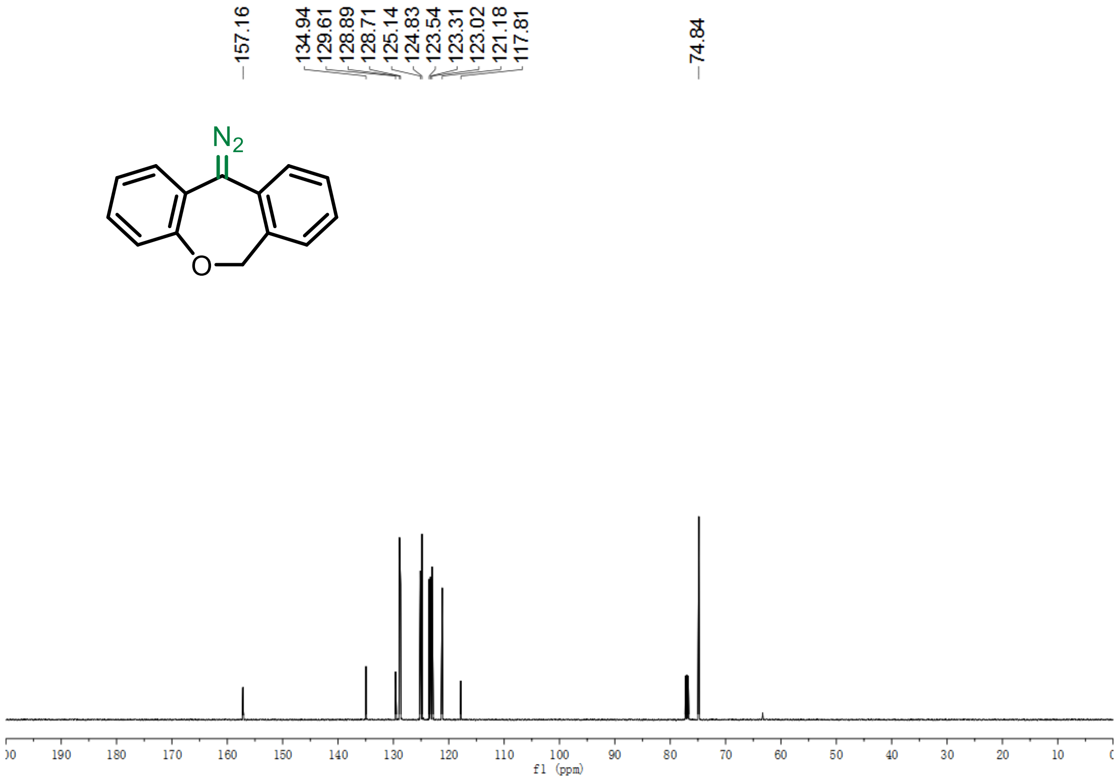

Supplementary Fig. 172 ^{13}C NMR (126 MHz, $CDCl_3$) spectrum of compound **S74**.

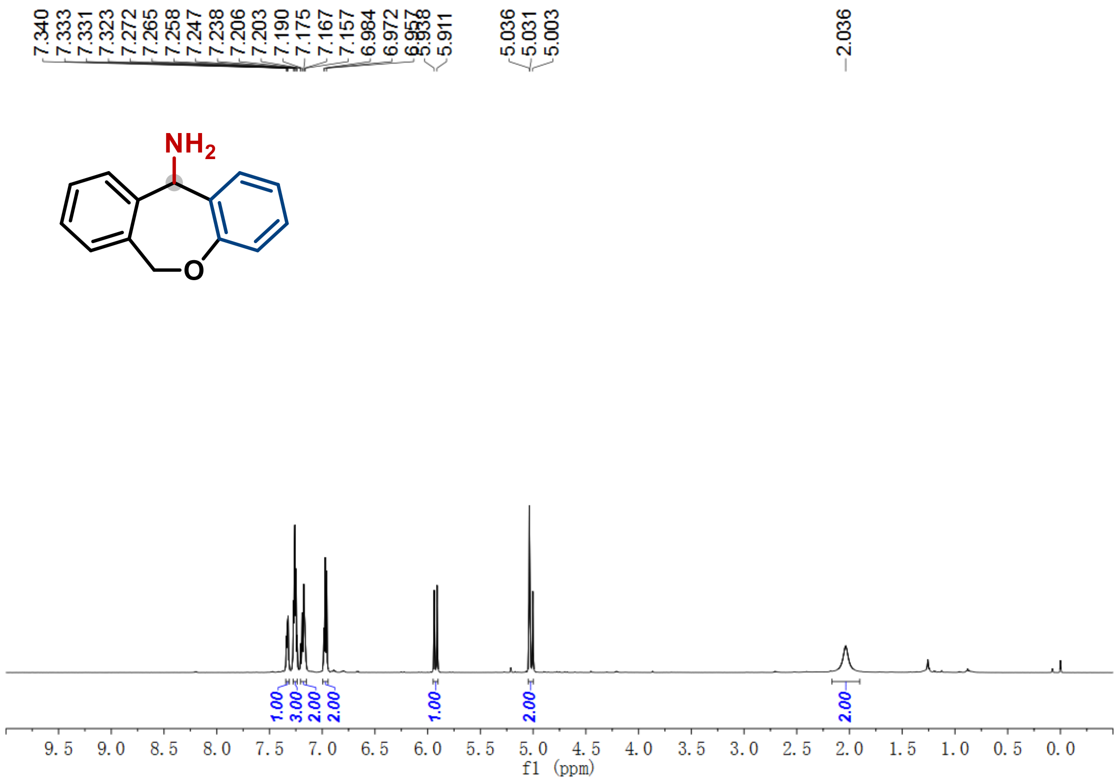

Supplementary Fig. 173 ^{1}H NMR (500 MHz, $CDCl_3$) spectrum of compound **74**.

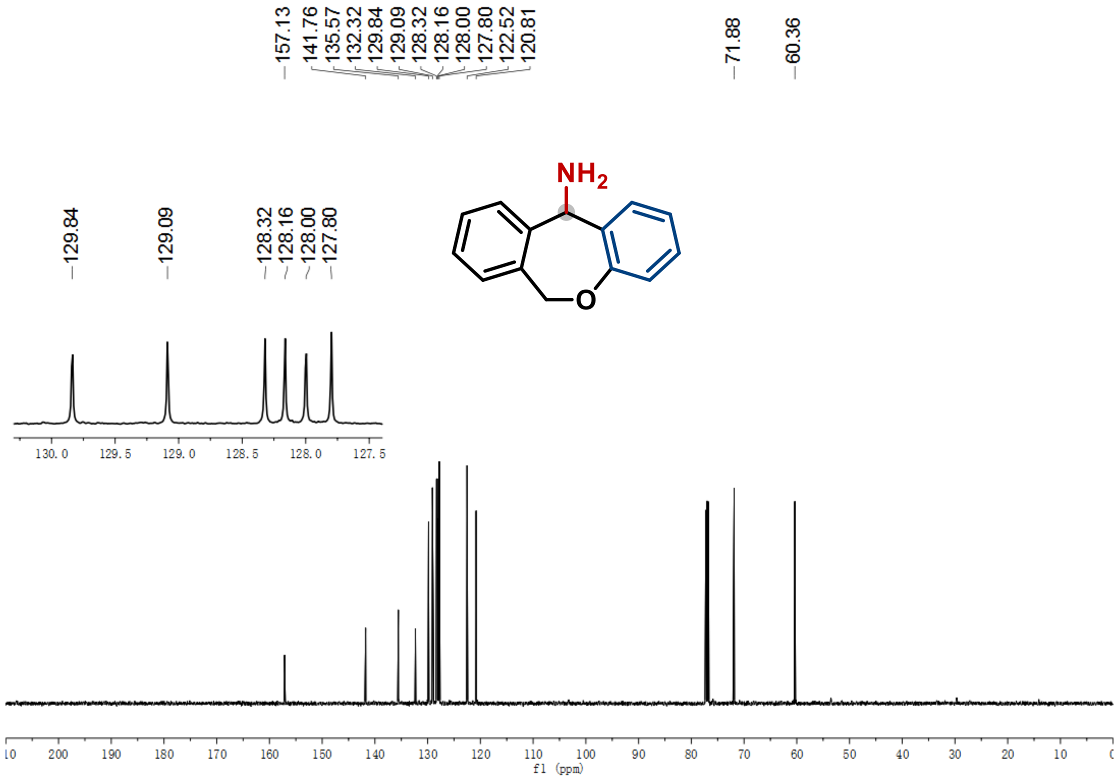

Supplementary Fig. 174 ^{13}C NMR (126 MHz, $CDCl_3$) spectrum of compound **74**.

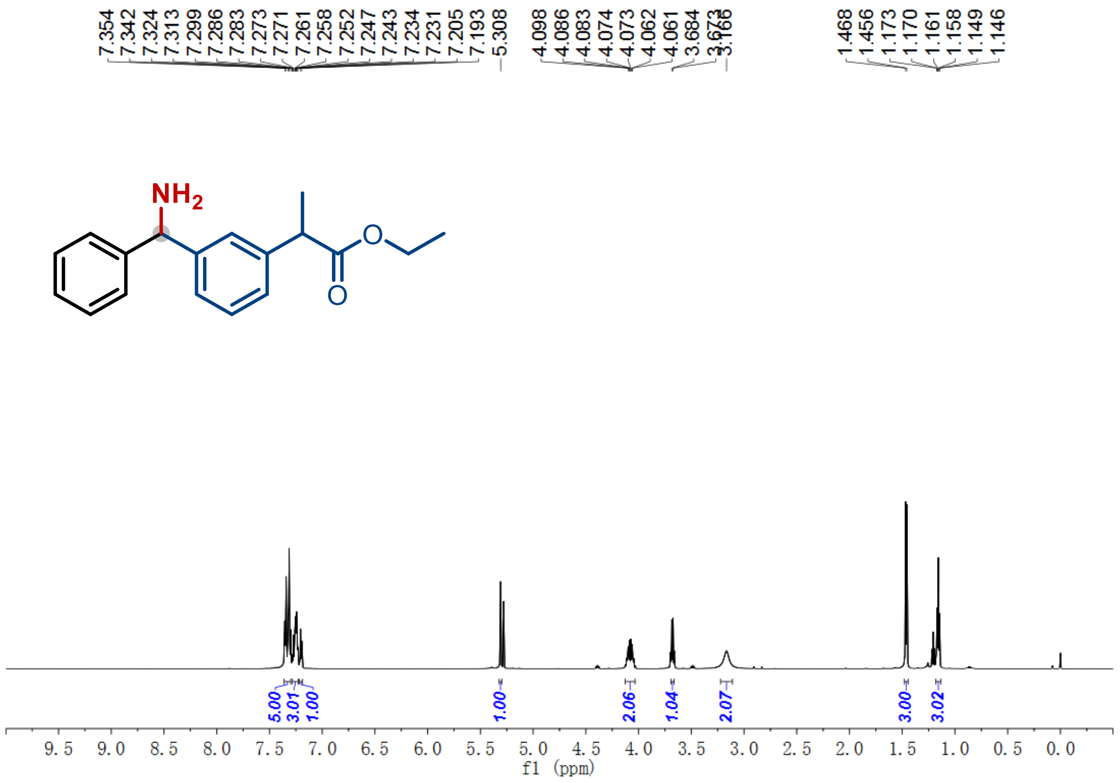

Supplementary Fig. 175 ^{1}H NMR (600 MHz, $CDCl_3$) spectrum of compound **75**.

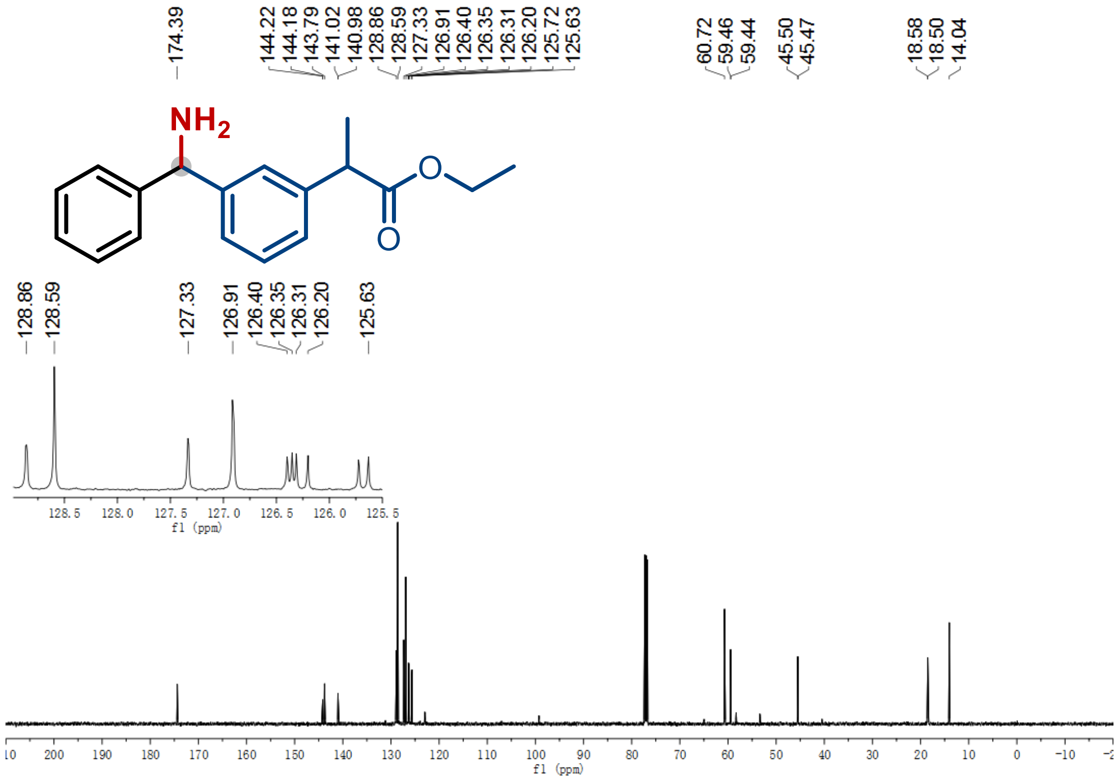

Supplementary Fig. 176 ^{13}C NMR (126 MHz, $CDCl_3$) spectrum of compound **75**.

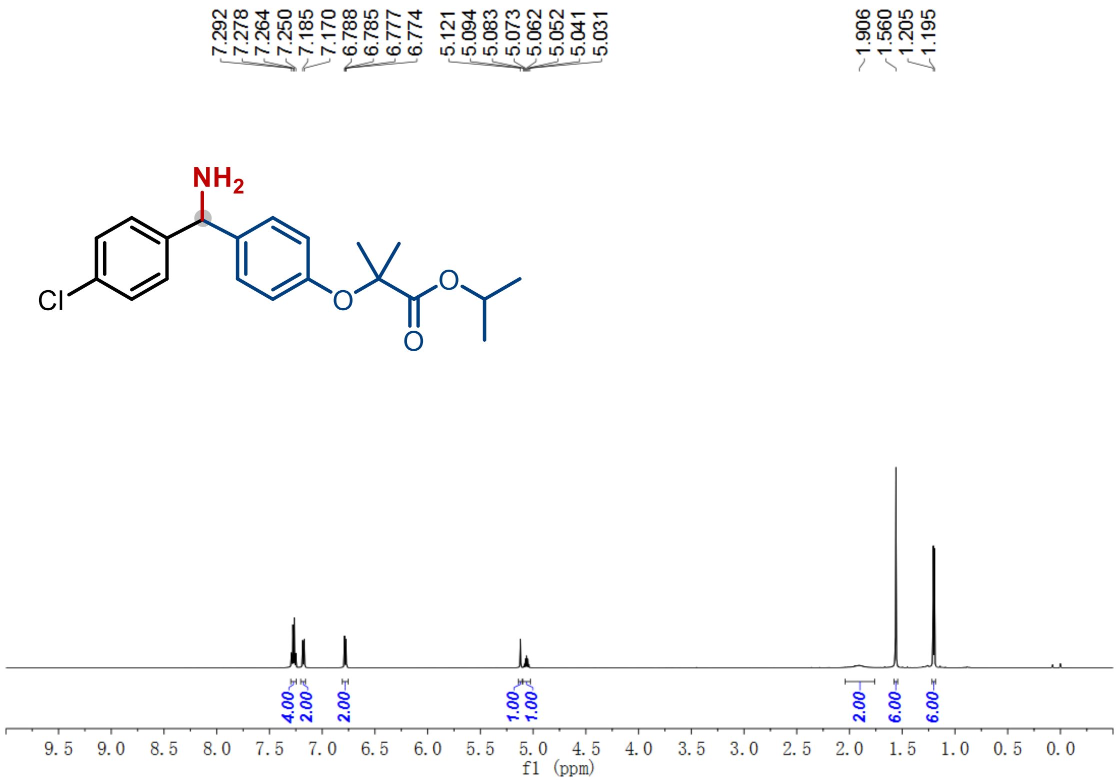

Supplementary Fig. 177 ^{1}H NMR (600 MHz, $CDCl_3$) spectrum of compound **76**.

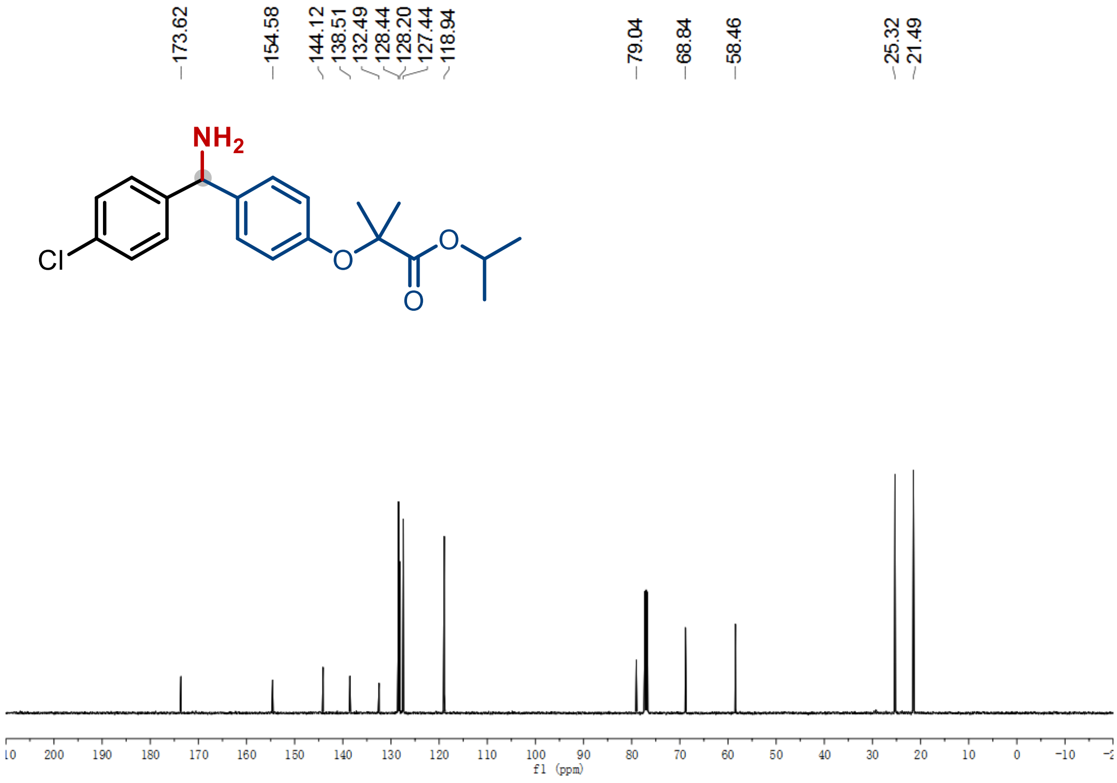

Supplementary Fig. 178 ^{13}C NMR (126 MHz, $CDCl_3$) spectrum of compound **76**.

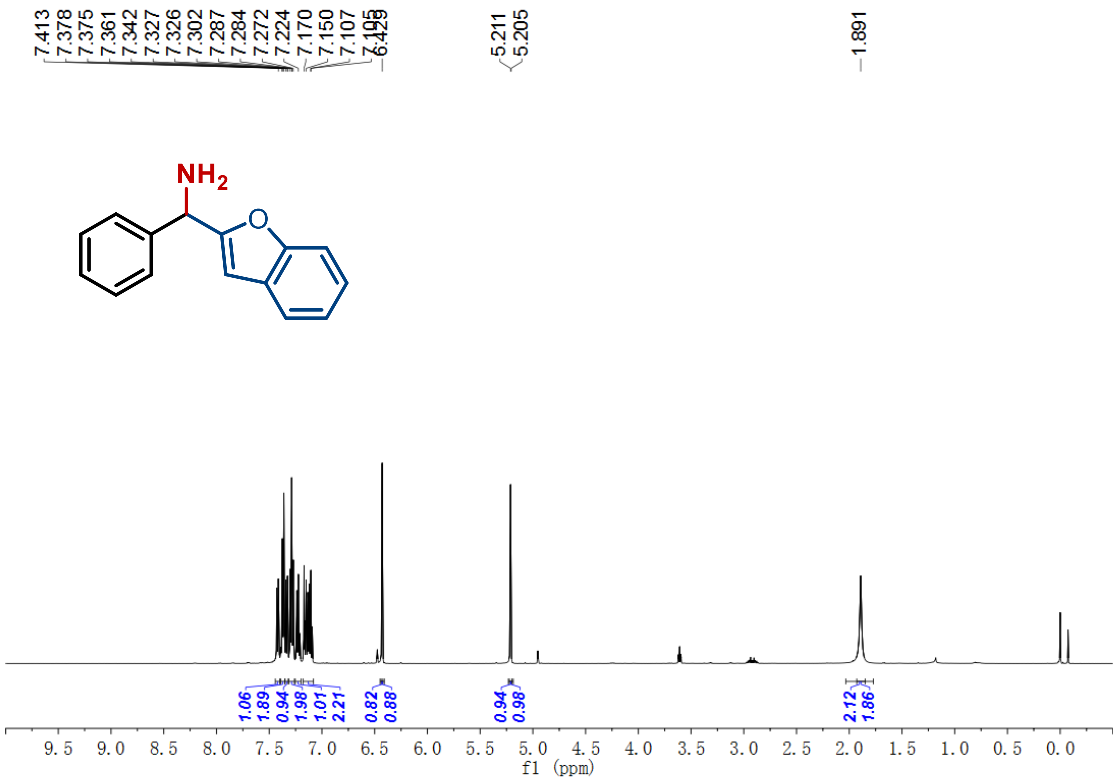

Supplementary Fig. 179 ^{1}H NMR (500 MHz, $CDCl_3$) spectrum of compound **77**.

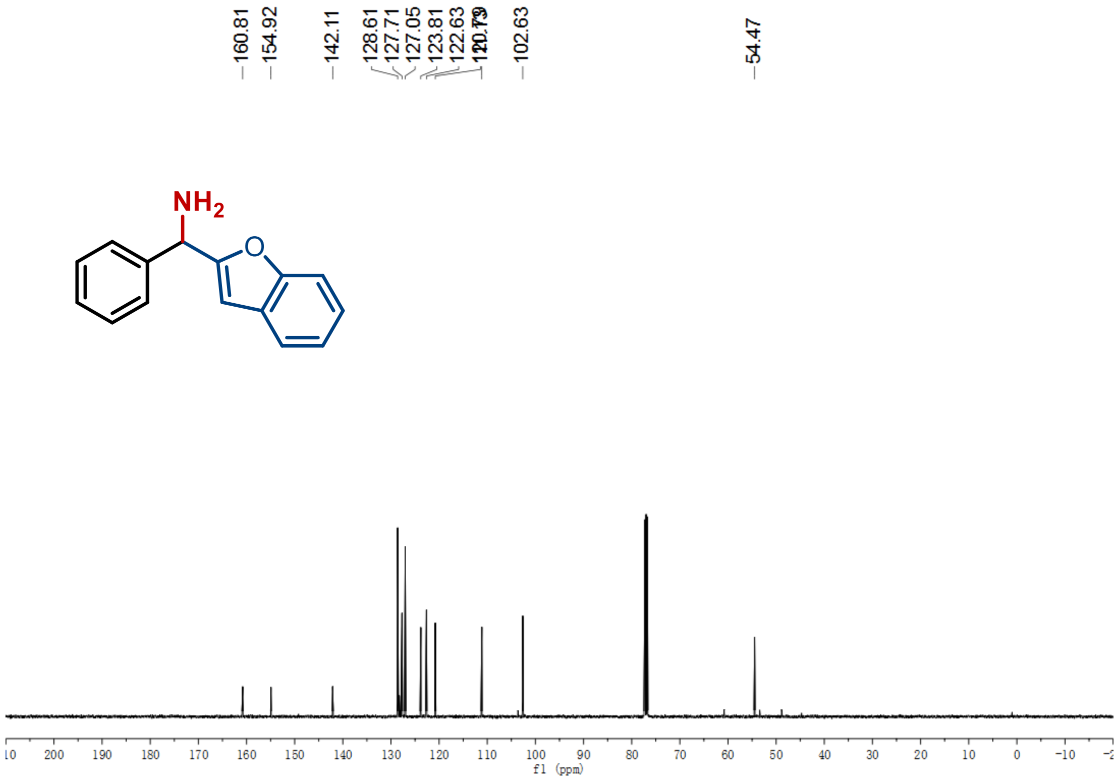

Supplementary Fig. 180 ^{13}C NMR (126 MHz, $CDCl_3$) spectrum of compound **77**.

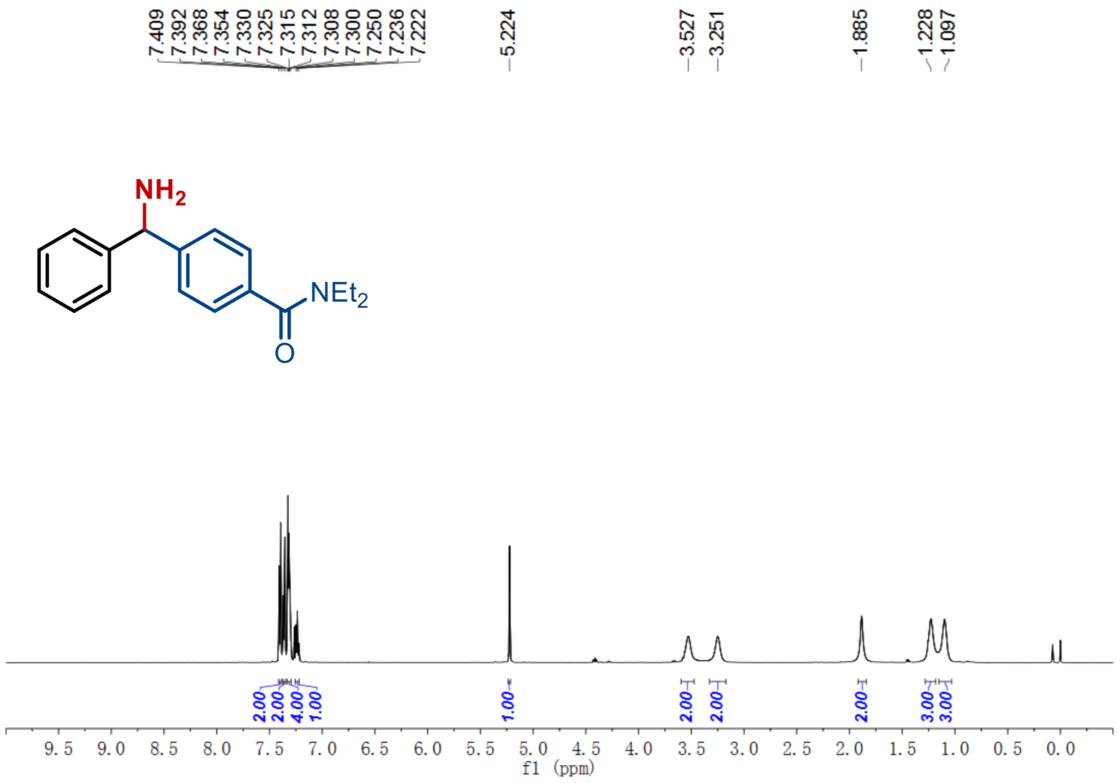

Supplementary Fig. 181 ^{1}H NMR (500 MHz, $CDCl_3$) spectrum of compound **78**.

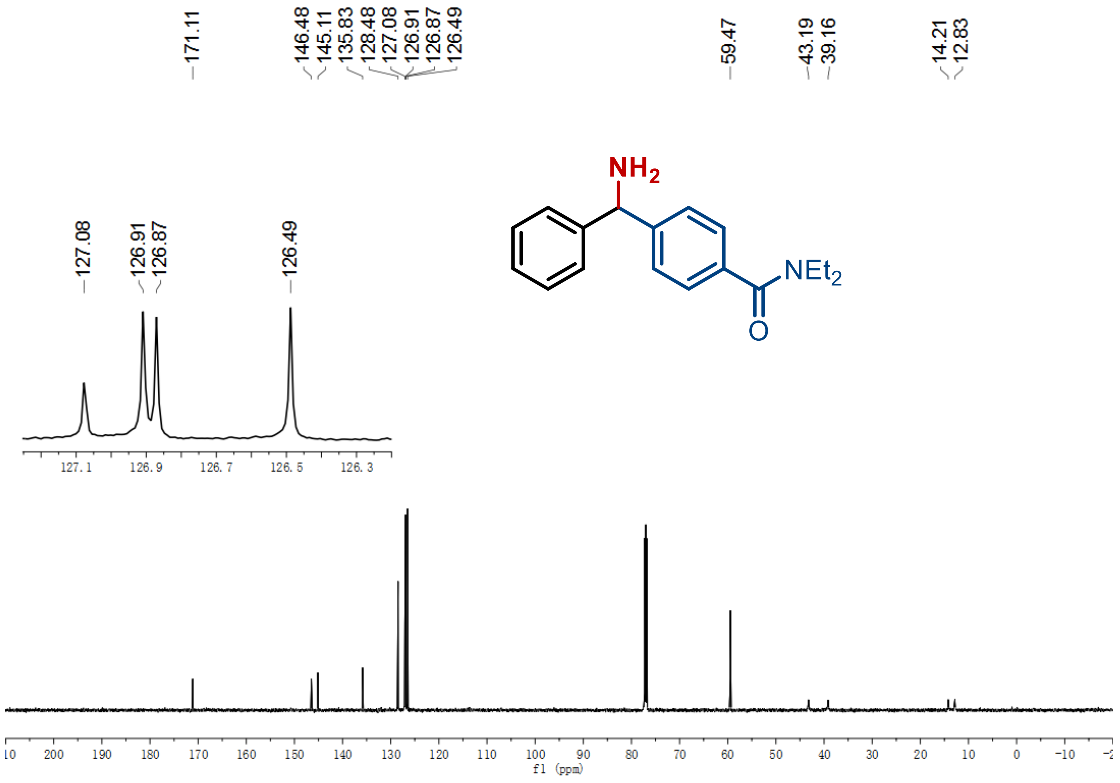

Supplementary Fig. 182 ^{13}C NMR (151 MHz, $CDCl_3$) spectrum of compound **78**.

Supplementary References

1. Urbano, J., Belderraín, T. R., Nicasio, M. C., Trofimenko, S., Díaz-Requejo, M. M. & Pérez, P. J. Functionalization of primary carbon−hydrogen bonds of alkanes by carbene insertion with a silver-based catalyst. *Organometallics* **24**, 1528 – 1532 (2005).
2. Dias, H.R. & Jin, W. Monomeric indium(I) and silver(I) complexes of a polyfluorinated tris(pyrazolyl)borate. *Inorg. Chem.* **35**, 267 – 268 (1996).
3. Tsuji, T., Tanaka, T., Tanaka, T., Yazaki, R. & Ohshima, T. Catalytic aerobic cross-dehydrogenative coupling of azlactones en route to α,α-disubstituted α-amino acids. *Org. Lett.* **22**, 4164 – 4170 (2020).
4. Smulik, J. A. & Vedejs, E. Improved reagent for electrophilic amination of stabilized carbanions. *Org. Lett.* **5**, 4187 – 4190 (2003).
5. Lin, H., Wang, C., Bannister, T. D. & Kamenecka, T. M. Site-selective γ-C(sp^3)−H and γ-C(sp^2)−H arylation of free amino esters promoted by a catalytic transient directing group. *Chem. –A Eur. J.* **24**, 9535 – 9541 (2018).
6. Kolar, P. & Tišler, M. Heterocyclic amino acids as synthons. Reactions with dicarbonyl compounds. *J. Heterocycl. Chem.* **30**, 1253 – 1260 (1993).
7. Lin, L., Fukagawa, S., Sekine, D., Tomita, E., Yoshino, T. & Matsunaga, S. Chiral carboxylic acid enabled achiral rhodium(III)-catalyzed enantioselective C−H functionalization. *Angew. Chem. Int. Ed.* **57**, 12048 – 12052 (2018).
8. Thurow, S., Fernandes, A. A. G., Quevedo-Acosta, Y., de Oliveira, M.F., de Oliveira, M.G. & Jurberg, I.D. Preparation of organic nitrates from aryldiazoacetates and $Fe(NO_3)_3\cdot 9H_2O$. O*rg. Lett.* **21**, 6909 – 6913 (2019).
9. Lu, J., Li, L., He, X. K., Xu, G. Y. & Xuan, J. Visible light-promoted sulfoxonium ylides synthesis from aryl diazoacetates and sulfoxides. *Chin. J. Chem.* **39**, 1646 – 1650. (2021).
10. Yang, L. L., Evans, D., Xu, B., Li, W.-T., Li, M.-L., Zhu, S.-F., Houk, K.N. & Zhou, Q.-L. Enantioselective diarylcarbene insertion into Si–H bonds Induced by electronic properties of the carbenes. *J. Am. Chem. Soc*. **142**, 12394 – 12399 (2020).
11. Nicolle, S. M. & Moody, C. J. Potassium *N*-Iodo *p*-toluenesulfonamide (TsNIK, Iodamine-T): A new reagent for the oxidation of hydrazones to diazo compounds. *Chem. Eur. J.* **20**, 4420 – 4425 (2014).
12. Roberts, E. CII. Preparation of potassium & sodium arylsulphoniodoamides. *J. Chem. Soc., Trans.* **123**, 849 – 853 (1923).
13. Coleman A. C., Areephong, J., Vicario, J., Meetsma, A., Browne, W. R. & Feringa, B. L. In situ generation of wavelength-shifting donor–acceptor mixed-monolayer-modified surfaces. *Angew. Chem. Int. Ed*. **49**, 6580 – 6584 (2010).
14. Liu, Z., Babu, K.R., Wang, F., Yang, Y. & Bi, X. Influence of sulfonyl substituents on the decomposition of N-sulfonylhydrazones at room temperature. *Org. Chem. Front. 6*, 121 – 124.
15. Frisch, M. J. Mennucci, B., Petersson, G.A., et al. Gaussian 16 Revision C.01, Gaussian, Inc., Wallingford CT, (2019).
16. Becke, A. D. Density-functional thermochemistry. III. The role of exact exchange. *J. Chem. Phys.* **98**, 5648−5652 (1993).

17. Perdew, J. P. & Wang, Y. Accurate and simple analytic representation of the electron-gas correlation energy. *Phys. Rev. B* **45**, 13244 – 13249 (1992).
18. Grimme, S., Ehrlich, S. & Goerigk, L. Effect of the damping function in dispersion corrected density functional theory. *J. Comp. Chem.* **32**, 1456 – 1465 (2011).
19. Küchle, W., Dolg, M., Stoll, H. & Preuss, H. Energy-adjusted pseudopotentials for the actinides. Parameter sets and test calculations for thorium and thorium monoxide. J. *Chem. Phys.* **100**, 7535 – 7542 (1994).
20. Cao, X., Dolg, M. & Stoll, H. Valence basis sets for relativistic energy-consistent small-core actinide pseudopotentials. *J. Chem. Phys.* **118**, 487 – 496 (2003).
21. McLean, A. D. & Chandler, G. S. Contracted Gaussian basis sets for molecular calculations. I. Second row atoms, $Z = 11–18$. *J. Chem. Phys.* **72**, 5639 – 5648 (1980).
22. Hehre, W. J., Ditchfield, R. & Pople, J. A. Self – consistent molecular orbital methods. XII. Further extensions of gaussian – type basis sets for use in molecular orbital studies of organic molecules. *J. Chem. Phys.* **56**, 2257 – 2261 (1972).
23. Fukui, K. Formulation of the reaction coordinate. *J. Phys. Chem.* **74**, 4161 – 4163 (1970).
24. Fukui, K. The path of chemical reactions - the IRC approach. *Acc. Chem. Res.* **14**, 363 – 368 (1981).
25. Zhao, Y. & Truhlar, D. G.. The M06 suite of density functionals for main group thermochemistry, thermochemical kinetics, noncovalent interactions, excited states, and transition elements: two new functionals and systematic testing of four M06-class functionals and 12 other functionals. *Theor. Chem. Acc.* **120**, 215 – 241 (2008).
26. Marenich, A. V., Cramer, C.J. & Truhlar, D. G. Universal solvation model based on solute electron density and on a continuum model of the solvent defined by the bulk dielectric constant and atomic surface tensions. *J. Phys. Chem. B*, **113**, 6378 – 6396 (2009).
27. Yang, Y.-F., Cheng, G.-J., Liu, P., Leow, D., Sun, T.-Y., Chen, P., Zhang, X., Yu, J.-Q., Wu, Y.-D. & Houk, K.N.. Palladium-catalyzed meta-selective C–H bond activation with a nitrile-containing template: computational study on mechanism and origins of selectivity. *J. Am. Chem. Soc.* **136**, 344 – 355 (2014).
28. Legault, C. Y. CYLview, 1.0b; Université de Sherbrooke: Canada, (2009).
29. Lu, T. & Chen, F. Multiwfn: A multifunctional wavefunction analyzer. *J. Comput. Chem.* **33**, 580−592 (2012).
