# Supplementary material for: Chemoselective carbene insertion into the N−H bonds of NH3·H2O
Source: Nat Commun. 2022 Dec 10;13:7649. doi: 10.1038/s41467-022-35394-z (PMC9741638; doi:10.1038/s41467-022-35394-z)
Supplement: Supplementary file 3 — Description of Additional Supplementary Files [file 41467_2022_35394_MOESM3_ESM.pdf]

Description of Additional Supplementary Files

Supplementary Data 1: Cartesian coordinates and energies of the computed structures
