# Supplementary material for: Chemoselective carbene insertion into the N−H bonds of NH3·H2O
Source: Nat Commun. 2022 Dec 10;13:7649. doi: 10.1038/s41467-022-35394-z (PMC9741638; doi:10.1038/s41467-022-35394-z)
Supplement: Supplementary file 4 — Supplementary Data 1 [file 41467_2022_35394_MOESM4_ESM.pdf]

Cartesian coordinates and energies of the computed structures

Cartesian coordinates of the SMD//M06/[6-311+G(d,p)-SDD(Ag/Br)] computed structures

$Tp^{Br3}Ag$

Zero-point correction=	0.105113 (Hartree/Particle)
Thermal correction to Energy=	0.134242
Thermal correction to Enthalpy=	0.135187
Thermal correction to Gibbs Free Energy=	0.034569
Sum of electronic and zero-point Energies=	-963.703966
Sum of electronic and thermal Energies=	-963.674837
Sum of electronic and thermal Enthalpies=	-963.673893
Sum of electronic and thermal Free Energies=	-963.774510

Ag	0.00371600	0.00001600	-2.38941000
N	1.78116000	-0.33558100	-0.93559200
N	1.45241900	-0.26653500	0.39099900
N	-0.59948100	1.70852700	-0.93791100
N	-0.49616100	1.39037700	0.38916500
N	-1.17780000	-1.37531000	-0.93826900
N	-0.95626700	-1.12498200	0.38886000
B	-0.00006900	-0.00023000	0.87287300
C	3.08181600	-0.57036000	-1.00567700
C	3.64910400	-0.66398400	0.27358700
C	2.57316200	-0.46390100	1.12922200
C	-1.04670900	2.95235900	-1.00935700
C	-1.25129300	3.49103100	0.26925000
C	-0.88742300	2.45974600	1.12600000
C	-2.03278500	-2.38325900	-1.00940700
C	-2.40083600	-2.82684400	0.26920600
C	-1.69033800	-1.99558300	1.12596800
H	0.00035300	0.00023000	2.05234200
Br	2.60924000	-0.45653900	3.03009000
Br	5.46535700	-0.98829500	0.73203300
Br	3.93311900	-0.73454000	-2.70194700
Br	-1.70610200	-2.03025100	3.02678700
Br	-3.59140700	-4.23671900	0.72638600
Br	-2.59754100	-3.03916600	-2.70640000
Br	-0.91249800	2.48819000	3.02682600
Br	-1.87702800	5.22695700	0.72600800
Br	-1.32799500	3.77120600	-2.70629500

Int0

Zero-point correction= 0.298547 (Hartree/Particle)
Thermal correction to Energy= 0.339813
Thermal correction to Enthalpy= 0.340757
Thermal correction to Gibbs Free Energy= 0.214590
Sum of electronic and zero-point Energies= -1574.086687
Sum of electronic and thermal Energies= -1574.045421
Sum of electronic and thermal Enthalpies= -1574.044477
Sum of electronic and thermal Free Energies= -1574.170644

Ag	1.31497900	0.13324100	-0.64430700
N	-0.61683400	-0.91667800	-1.59711300
N	-1.68411700	-1.02749600	-0.74965000
N	0.40047600	-0.81950900	1.35952400
N	-0.90706000	-0.49783800	1.60261800
N	-0.28996000	1.80882900	-0.20094500
N	-1.56785200	1.38340000	0.03499400
B	-1.86334000	-0.07399400	0.45955300
C	-0.74296400	-1.88252200	-2.49504000
C	-1.89064400	-2.65437900	-2.26352500
C	-2.45575200	-2.07037800	-1.13671600
C	0.94000100	-1.12858900	2.53067200
C	0.00534500	-1.01738100	3.57078200
C	-1.15657200	-0.61042000	2.92826800
C	-0.36879200	3.07903700	-0.56980600
C	-1.69947300	3.52377800	-0.58173400
C	-2.42587200	2.40634100	-0.19152000
H	-2.97978900	-0.14591700	0.84369200
Br	-4.04058700	-2.60359900	-0.23064600
Br	-2.53480700	-4.14077600	-3.26086400
Br	0.54872500	-2.07543000	-3.88602300
Br	-4.31250900	2.26641900	0.00707800
Br	-2.35770700	5.25489300	-1.01667100
Br	1.21339400	4.04368200	-1.00261000
Br	-2.84767500	-0.24568200	3.71871800
Br	0.25980200	-1.35859500	5.42519900
Br	2.76710500	-1.65590500	2.63929400
N	3.32028500	0.09744500	-3.80046700
N	3.46219000	0.12044500	-2.67406600
C	3.60993800	0.13214400	-1.34139800
C	4.11827000	-1.17177500	-0.78997800
C	3.47072200	-2.37774100	-1.09932600
C	5.24871600	-1.19468200	0.03664000
C	3.94143300	-3.58273900	-0.58469200
H	2.58772800	-2.37031900	-1.73134100

C	5.70623700	-2.40314200	0.56283000
H	5.76168100	-0.27026900	0.27644500
C	5.05655800	-3.60007000	0.25579300
H	3.42387000	-4.50585100	-0.82744800
H	6.58088000	-2.40685900	1.20699200
H	5.41527700	-4.53803200	0.66851700
C	4.00806000	1.45744700	-0.76912500
C	4.48496400	2.50111800	-1.57205100
C	3.89321200	1.66211700	0.61716500
C	4.82474400	3.72972100	-1.00415300
H	4.59114000	2.36347200	-2.64419000
C	4.24471400	2.88551900	1.17920700
H	3.52807300	0.85987000	1.25004600
C	4.70760100	3.92774200	0.37191200
H	5.19205700	4.52841500	-1.64199500
H	4.14385800	3.02714100	2.25101800
H	4.97442500	4.88365700	0.81181600

TS1

Zero-point correction= 0.296815 (Hartree/Particle)
Thermal correction to Energy= 0.338536
Thermal correction to Enthalpy= 0.339481
Thermal correction to Gibbs Free Energy= 0.213462
Sum of electronic and zero-point Energies= -1574.070196
Sum of electronic and thermal Energies= -1574.028475
Sum of electronic and thermal Enthalpies= -1574.027530
Sum of electronic and thermal Free Energies= -1574.153549

Ag	-0.74065300	0.84329700	-0.92179200
N	1.39460800	1.42369000	-0.53124200
N	2.07378200	0.87549900	0.52161100
N	-0.49021700	0.82094900	1.70426500
N	0.28578900	-0.29303600	1.87746200
N	0.22871800	-1.46896600	-0.71825200
N	1.39783700	-1.49758200	-0.01045100
B	1.63220200	-0.47887400	1.13138700
C	2.01118500	2.55573800	-0.84243900
C	3.11383300	2.77943600	-0.00731100
C	3.11017500	1.68147600	0.84535700
C	-1.61148000	0.59560300	2.37968000
C	-1.60556300	-0.66867900	2.99211100
C	-0.36965800	-1.19663600	2.63815300
C	0.33971400	-2.39207700	-1.66221400

C	1.57807100	-3.05218100	-1.59556200
C	2.21809500	-2.44072000	-0.52542800
H	2.47522600	-0.88074000	1.86526000
Br	4.33777300	1.31154600	2.24706200
Br	4.33813000	4.23402600	-0.03873300
Br	1.36384800	3.64722900	-2.26530200
Br	3.95490900	-2.79978000	0.16132500
Br	2.22697200	-4.46535700	-2.69316100
Br	-1.07799800	-2.68381000	-2.90744600
Br	0.35472700	-2.90349600	3.05965000
Br	-2.97222900	-1.49130200	4.03115400
Br	-3.01442600	1.88822300	2.37773100
N	-2.07717600	1.08886600	-4.31086000
N	-2.54821500	0.92579800	-3.31514300
C	-2.80437100	0.88122900	-1.58564600
C	-3.52566000	2.15393900	-1.31851800
C	-2.82925300	3.37607200	-1.38739300
C	-4.86373400	2.16078100	-0.88947500
C	-3.45319800	4.56971400	-1.04796300
H	-1.78581400	3.37276300	-1.69155700
C	-5.48046200	3.35626800	-0.52354300
H	-5.40653200	1.22631000	-0.81118900
C	-4.78109300	4.56131200	-0.60610400
H	-2.90112500	5.50299400	-1.10264300
H	-6.50847800	3.34563500	-0.17373300
H	-5.26393000	5.49120400	-0.32013400
C	-3.54935400	-0.39761100	-1.40081900
C	-4.57277500	-0.82469100	-2.26091200
C	-3.22918900	-1.18741900	-0.28681300
C	-5.23819700	-2.02742800	-2.02858100
H	-4.84149400	-0.21622700	-3.12074500
C	-3.92475900	-2.36684500	-0.03051300
H	-2.42650500	-0.88629400	0.37355000
C	-4.92157800	-2.79779800	-0.90715000
H	-6.01152500	-2.35600700	-2.71674700
H	-3.66448300	-2.95788700	0.84210400
H	-5.44573400	-3.73053700	-0.72190700

Int1

Zero-point correction= 0.296630 (Hartree/Particle)
Thermal correction to Energy= 0.339850
Thermal correction to Enthalpy= 0.340794
Thermal correction to Gibbs Free Energy= 0.208952

Sum of electronic and zero-point Energies= -1574.108439
Sum of electronic and thermal Energies= -1574.065219
Sum of electronic and thermal Enthalpies= -1574.064275
Sum of electronic and thermal Free Energies= -1574.196117

Ag	-0.70437300	1.14747800	-0.68599400
N	1.40677500	1.32078700	-0.02869900
N	1.93907000	0.51001500	0.93704800
N	-0.72311800	0.47262400	1.89030400
N	-0.05631900	-0.72118300	1.90254800
N	-0.06673600	-1.50703600	-0.79042000
N	1.11751800	-1.61888000	-0.11698800
B	1.32823600	-0.88522300	1.23083900
C	2.18506500	2.39432300	-0.10513600
C	3.25039400	2.31253400	0.80038800
C	3.04868400	1.09528700	1.44054100
C	-1.92317000	0.22801800	2.39866300
C	-2.07850700	-1.12456200	2.74477200
C	-0.85479800	-1.68905100	2.40519600
C	0.09807900	-2.15950000	-1.93311100
C	1.38755800	-2.70841400	-2.03962700
C	2.00172900	-2.32820000	-0.85378100
H	2.07057600	-1.50499700	1.92253700
Br	4.12382200	0.32663700	2.80400800
Br	4.65198400	3.56583100	1.08204900
Br	1.78413600	3.80273500	-1.32224500
Br	3.78981800	-2.64914700	-0.29287400
Br	2.13618700	-3.71176700	-3.47268400
Br	-1.31172200	-2.24240600	-3.21986000
Br	-0.31209400	-3.50656200	2.53881400
Br	-3.60246000	-1.99559500	3.48289100
Br	-3.22352800	1.62259600	2.51350400
N	2.24290200	0.27340200	-3.01664400
N	1.30253100	0.67730500	-3.43341500
C	-2.68607700	1.42027900	-1.30311000
C	-3.14183600	2.76924600	-1.54552900
C	-2.27515400	3.68649500	-2.19040100
C	-4.39854300	3.24224100	-1.08778000
C	-2.67519600	4.99269700	-2.43303900
H	-1.29809100	3.33590000	-2.50735800
C	-4.76739300	4.56887200	-1.27881200
H	-5.04392900	2.57242700	-0.53088700
C	-3.91750000	5.43988000	-1.96693600
H	-2.01270100	5.67491800	-2.95688400

H	-5.71644900	4.92774300	-0.89209300
H	-4.21344200	6.47323800	-2.12292100
C	-3.63879900	0.34781100	-1.28519500
C	-4.87650900	0.40103700	-1.98553800
C	-3.32788200	-0.83744900	-0.56860300
C	-5.76586700	-0.66173000	-1.93036000
H	-5.10120500	1.26381900	-2.60221600
C	-4.24634200	-1.87183400	-0.46897700
H	-2.35919400	-0.92346700	-0.09525300
C	-5.46366900	-1.78772200	-1.15263200
H	-6.69635800	-0.62136400	-2.48840000
H	-4.00398100	-2.75403900	0.11412000
H	-6.16985300	-2.61161400	-1.10133800

Int1-N

Zero-point correction= 0.325343 (Hartree/Particle)
Thermal correction to Energy= 0.369204
Thermal correction to Enthalpy= 0.370148
Thermal correction to Gibbs Free Energy= 0.237598
Sum of electronic and zero-point Energies= -1521.136130
Sum of electronic and thermal Energies= -1521.092269
Sum of electronic and thermal Enthalpies= -1521.091325
Sum of electronic and thermal Free Energies= -1521.223875

Ag	0.43744200	1.24614900	-0.62516800
N	-1.69292900	0.64546700	-0.64569600
N	-2.10783300	-0.63954100	-0.42727000
N	0.28738600	-1.29592100	-1.72652700
N	-0.04045500	-2.06428900	-0.64312400
N	0.74404100	-0.55943000	1.56003300
N	-0.56984300	-0.93716800	1.53523800
B	-1.18696100	-1.65246100	0.30092000
C	-2.68429400	1.27209700	-1.27429000
C	-3.77296200	0.41393200	-1.47523500
C	-3.35519700	-0.79084500	-0.92070700
C	1.37643500	-1.84387300	-2.24571900
C	1.79926300	-2.96584100	-1.51061800
C	0.85772000	-3.06318000	-0.49477300
C	0.92210600	0.05510000	2.72207700
C	-0.26470700	0.10610000	3.47696700
C	-1.18908400	-0.54324900	2.66994000
H	-1.82795700	-2.59485400	0.64625800
Br	-4.33427900	-2.41404500	-0.82586100

Br	-5.44167800	0.80585100	-2.29783400
Br	-2.50790300	3.09347400	-1.79600500
Br	-3.04527900	-0.82774400	2.97808500
Br	-0.55589000	0.88266100	5.19230700
Br	2.62572200	0.77685300	3.20167600
Br	0.77866500	-4.32004900	0.92917900
Br	3.31098300	-4.08513600	-1.81436700
Br	2.21351300	-1.06932900	-3.77794300
C	2.29311700	2.21579600	-0.61716800
C	2.33491800	3.60762900	-0.99426000
C	1.25159400	4.44863100	-0.62895100
C	3.39019700	4.15407500	-1.76925700
C	1.26473100	5.79336100	-0.97592200
H	0.44166800	4.02916000	-0.03356800
C	3.35747300	5.48559300	-2.16623800
H	4.19520100	3.50660600	-2.10004100
C	2.30562400	6.31094100	-1.75614700
H	0.44984100	6.43846200	-0.66066100
H	4.15038200	5.88439700	-2.79191800
H	2.28991100	7.35476700	-2.05682600
C	3.50601800	1.52831700	-0.27923200
C	4.66861600	2.20047800	0.18899100
C	3.52645900	0.11054200	-0.31968200
C	5.79537900	1.48316100	0.56399600
H	4.64976700	3.27832900	0.30174600
C	4.67091400	-0.60001000	0.00777000
H	2.62531200	-0.41060800	-0.60983400
C	5.80488400	0.08596600	0.45573200
H	6.66892200	2.00344800	0.94513300
H	4.67078200	-1.68323600	-0.05534500
H	6.69235000	-0.46966500	0.74545900
N	-0.90526600	3.05132100	1.65286900
H	-0.32020700	2.52873800	2.30313500
H	-1.61331900	2.39230300	1.33287200
H	-1.39343100	3.75527800	2.20625400

TS1-N

Zero-point correction= 0.325067 (Hartree/Particle)
Thermal correction to Energy= 0.368277
Thermal correction to Enthalpy= 0.369221
Thermal correction to Gibbs Free Energy= 0.238768
Sum of electronic and zero-point Energies= -1521.133246
Sum of electronic and thermal Energies= -1521.090036

Sum of electronic and thermal Enthalpies= -1521.089092
Sum of electronic and thermal Free Energies= -1521.219546

Ag	0.70856200	1.18637000	0.43361900
N	-1.47872900	1.23306100	0.05537300
N	-2.03937900	0.40295400	-0.87784500
N	0.56318900	0.31192800	-1.98443300
N	-0.10686900	-0.87768200	-1.91090500
N	0.12607100	-1.54914000	0.80177300
N	-1.12291000	-1.63192100	0.24996700
B	-1.43423000	-1.00442200	-1.13273300
C	-2.28558700	2.28185300	0.17283900
C	-3.39342600	2.16710200	-0.67612400
C	-3.18994300	0.95292700	-1.32310500
C	1.72908300	0.04343000	-2.55899900
C	1.85753100	-1.32208600	-2.86079400
C	0.65578100	-1.86712400	-2.42472500
C	0.01504200	-2.06068400	2.02047600
C	-1.29850100	-2.47464900	2.30257100
C	-1.98781200	-2.17402800	1.13670800
H	-2.21992600	-1.67300000	-1.72514200
Br	-4.30999700	0.14198300	-2.62311600
Br	-4.84601200	3.37572500	-0.88474600
Br	-1.87502400	3.69250600	1.38695400
Br	-3.84026900	-2.40042300	0.76436700
Br	-1.98008500	-3.27100400	3.89186900
Br	1.53231100	-2.19023900	3.18033800
Br	0.10037000	-3.68446600	-2.45899900
Br	3.33464400	-2.22926800	-3.64796700
Br	3.01910900	1.42664000	-2.81359900
C	2.67925900	1.55854900	1.02047000
C	3.19161900	2.90229700	1.09566300
C	2.29931700	3.97767100	1.33090500
C	4.55698500	3.20674000	0.85732500
C	2.75675000	5.28443200	1.39028400
H	1.25091100	3.74921700	1.48627800
C	5.00391100	4.52261000	0.87441800
H	5.24267600	2.40493000	0.60937200
C	4.10998700	5.55990500	1.15584400
H	2.06411800	6.09471400	1.59598200
H	6.04571000	4.74439800	0.66375400
H	4.46294400	6.58716500	1.17340000
C	3.57589000	0.45488100	1.24816500
C	4.60358900	0.50378100	2.22503800

C	3.41141700	-0.73732400	0.50152500
C	5.43546900	-0.59072400	2.42544500
H	4.68971200	1.38277800	2.85401100
C	4.29732500	-1.79479800	0.65097400
H	2.59290100	-0.80983900	-0.20206800
C	5.30315400	-1.72883400	1.62071600
H	6.19599600	-0.55825400	3.19992400
H	4.17917600	-2.68519800	0.04171500
H	5.97095300	-2.57321100	1.76577300
N	1.30702600	1.59100400	3.83607700
H	1.45601400	0.61613300	3.57916100
H	0.49608900	1.88955700	3.29525300
H	1.01500200	1.58543900	4.81387600

Int2-N

Zero-point correction= 0.332241 (Hartree/Particle)
Thermal correction to Energy= 0.373996
Thermal correction to Enthalpy= 0.374940
Thermal correction to Gibbs Free Energy= 0.249565
Sum of electronic and zero-point Energies= -1521.161350
Sum of electronic and thermal Energies= -1521.119595
Sum of electronic and thermal Enthalpies= -1521.118650
Sum of electronic and thermal Free Energies= -1521.244025

Ag	0.46188900	1.20952200	0.70359300
N	-1.56263300	1.08125400	-0.13113400
N	-1.93400500	0.09083600	-0.99712400
N	0.80037200	0.12307200	-1.71956800
N	0.19882100	-1.10438800	-1.63736700
N	0.21615400	-1.74363400	1.08183300
N	-1.01065000	-1.70960000	0.48640300
B	-1.19125800	-1.26923400	-0.99908700
C	-2.40249900	2.09490800	-0.31249900
C	-3.34899900	1.79059700	-1.29973300
C	-3.00730300	0.50536400	-1.70498100
C	1.99275000	-0.08867700	-2.25664800
C	2.20908100	-1.45077000	-2.52927000
C	1.03353100	-2.05747800	-2.11047700
C	-0.00630000	-2.12113700	2.33102800
C	-1.36802100	-2.33355900	2.59579600
C	-1.96969500	-2.05643800	1.37468100
H	-1.84304900	-2.07460900	-1.58625100
Br	-3.87436600	-0.55541200	-3.01874200

Br	-4.78025500	2.87332000	-1.92753600
Br	-2.20596200	3.70097200	0.68958800
Br	-3.81703200	-2.10381600	0.92813200
Br	-2.20051600	-2.87813100	4.21885300
Br	1.46283300	-2.33121300	3.55861600
Br	0.58896000	-3.90694500	-2.12379000
Br	3.74033800	-2.27802900	-3.30094700
Br	3.18823300	1.36315300	-2.57788900
C	2.30117900	1.68004100	1.73822600
C	2.37848200	3.17507500	1.80955300
C	2.27640900	3.92101100	2.99314200
C	2.52678300	3.88627000	0.59976300
C	2.29913600	5.32092200	2.97171900
H	2.16305900	3.43912500	3.96159200
C	2.56043600	5.27332800	0.58081500
H	2.60247700	3.32773100	-0.32827000
C	2.44159400	6.00501800	1.76989600
H	2.20816800	5.86823200	3.90586800
H	2.67012300	5.79075000	-0.36809000
H	2.45783100	7.09039400	1.75229400
C	3.51091900	0.98589000	1.14653300
C	4.75898900	1.62135600	1.05502700
C	3.41007600	-0.35365300	0.73195100
C	5.86737600	0.94383800	0.54491800
H	4.85811100	2.65765700	1.36124700
C	4.52159100	-1.03521100	0.24140000
H	2.44573100	-0.85243100	0.75782600
C	5.75364300	-0.38619400	0.13871800
H	6.82138100	1.45803300	0.46685700
H	4.41543800	-2.06372500	-0.08888900
H	6.61497300	-0.91090300	-0.26429700
N	2.19555800	1.11902100	3.16890300
H	2.12815400	0.09477800	3.12713600
H	1.35147900	1.46133300	3.63538800
H	3.01516000	1.35687200	3.75322900

Int3-N

Zero-point correction= 0.408236 (Hartree/Particle)
Thermal correction to Energy= 0.456516
Thermal correction to Enthalpy= 0.457460
Thermal correction to Gibbs Free Energy= 0.318822
Sum of electronic and zero-point Energies= -1750.394193
Sum of electronic and thermal Energies= -1750.345913

Sum of electronic and thermal Enthalpies= -1750.344969
Sum of electronic and thermal Free Energies= -1750.483607

Ag	-0.57936500	0.78348600	-1.14021300
N	1.44068500	1.27342500	-0.20531000
N	1.67217500	0.79892000	1.06223600
N	-1.01136200	0.68807500	1.93501600
N	-0.38612700	-0.51769500	1.74734700
N	0.52578500	-1.59601800	-0.76306700
N	1.39551900	-1.55779600	0.28500100
B	1.13531400	-0.59985700	1.46953600
C	2.30309600	2.26660600	-0.39463000
C	3.09459600	2.48745000	0.73998600
C	2.65557700	1.52457600	1.63960500
C	-2.22234400	0.39730600	2.39863200
C	-2.42587500	-0.98697300	2.51510200
C	-1.21746700	-1.52626000	2.10010800
C	1.08742700	-2.35539900	-1.68625900
C	2.34174600	-2.83777200	-1.27527000
C	2.49552400	-2.29141800	-0.00679200
H	1.72341100	-0.98075400	2.43149600
Br	3.28630000	1.22942900	3.40411100
Br	4.46540300	3.78053200	0.98624500
Br	2.48046300	3.14132900	-2.08094200
Br	3.97969500	-2.46139300	1.16993500
Br	3.54542400	-3.97149000	-2.21699900
Br	0.19335600	-2.67360200	-3.35363200
Br	-0.70971500	-3.35886600	2.06036300
Br	-3.98325000	-1.91217700	3.08846500
Br	-3.44508200	1.77748200	2.90662300
C	-2.53682600	0.52451700	-2.11524100
C	-3.33245600	1.78005500	-1.93321300
C	-3.44091600	2.80073000	-2.88941200
C	-4.00447100	1.96423300	-0.70535500
C	-4.15797500	3.97028600	-2.61855300
H	-2.93848300	2.72762100	-3.84806200
C	-4.71781900	3.12704600	-0.43806200
H	-3.96412800	1.17367800	0.03580700
C	-4.79515400	4.14693400	-1.39493700
H	-4.20294100	4.74849000	-3.37487800
H	-5.22006100	3.23415500	0.51936000
H	-5.34664100	5.05815600	-1.18533800
C	-3.33174700	-0.71509000	-1.72775500
C	-4.66811800	-0.83953300	-2.15000700

C	-2.76981000	-1.76207300	-0.99014600
C	-5.41504500	-1.97535600	-1.84568100
H	-5.13514200	-0.02294500	-2.69579800
C	-3.51754000	-2.89985200	-0.67706100
H	-1.74187200	-1.68393900	-0.66104300
C	-4.83983300	-3.01106800	-1.10422200
H	-6.44742800	-2.04864100	-2.17661400
H	-3.06264100	-3.69231900	-0.09085400
H	-5.42194700	-3.89434200	-0.85710900
N	-2.19153400	0.35995000	-3.59842100
H	-1.72295100	-0.54300700	-3.71273900
H	-1.51166900	1.11477000	-3.89114900
H	-3.02129500	0.37036500	-4.21107900
O	-0.75831600	4.15586600	-2.11084800
H	-2.25049000	3.14093100	0.37889000
H	0.06219700	4.65234200	-1.96896600
O	-0.36789100	2.32937800	-4.05443300
H	0.50947200	1.97838700	-3.83391500
H	-0.54136300	3.00830900	-3.33736700
O	-1.29384800	3.30918100	0.45905500
H	-0.95777200	2.53931500	0.96323000
H	-0.95849600	3.77023400	-1.22227300

Int4-N

Zero-point correction= 0.299065 (Hartree/Particle)
Thermal correction to Energy= 0.317425
Thermal correction to Enthalpy= 0.318369
Thermal correction to Gibbs Free Energy= 0.252677
Sum of electronic and zero-point Energies= -786.634167
Sum of electronic and thermal Energies= -786.615807
Sum of electronic and thermal Enthalpies= -786.614863
Sum of electronic and thermal Free Energies= -786.680555

C	0.15500600	0.27115800	-0.21010400
C	-0.77316300	-0.85307200	-0.05379900
C	-1.75994600	-1.21270200	-0.99780400
C	-0.72832600	-1.61736500	1.13652300
C	-2.66071700	-2.25462400	-0.75516000
H	-1.82740300	-0.69795200	-1.95302200
C	-1.61197300	-2.66164200	1.36636500
H	0.01530700	-1.36396500	1.88692600
C	-2.59704900	-2.98797000	0.42523500
H	-3.40649500	-2.49669600	-1.50847600

H	-1.54447800	-3.22112400	2.29602500
H	-3.29666600	-3.79648300	0.61425100
C	1.62200100	0.02968700	-0.13179400
C	2.18973700	-1.25839500	-0.13715700
C	2.50841900	1.12660800	-0.06399000
C	3.57043000	-1.44020700	-0.07626400
H	1.53649600	-2.12309700	-0.19215700
C	3.88830200	0.94472900	-0.02933500
H	2.10044200	2.13263200	0.01332500
C	4.43079900	-0.34229000	-0.03215400
H	3.97683200	-2.44860300	-0.08120600
H	4.54271600	1.81126400	0.02358100
H	5.50670600	-0.48634300	0.00842700
N	-0.15475300	1.06961700	-1.45679300
H	0.44048600	1.89922700	-1.46898700
H	-1.16892900	1.39714000	-1.43205500
H	0.01362900	0.56508900	-2.34327500
O	-2.70522300	3.22583900	1.00708800
H	-0.09779500	1.57888000	0.82581200
H	-2.68219000	4.17403000	0.80921100
O	-2.76822100	1.76696200	-1.22713200
H	-3.11129900	0.91431800	-0.91047400
H	-2.83282500	2.36469100	-0.42557100
O	-0.14667300	2.55496300	1.22711400
H	0.23316500	2.47100300	2.11540700
H	-1.75461800	2.99308400	1.21987500

TS2-N

Zero-point correction=	0.295965 (Hartree/Particle)
Thermal correction to Energy=	0.313835
Thermal correction to Enthalpy=	0.314779
Thermal correction to Gibbs Free Energy=	0.250015
Sum of electronic and zero-point Energies=	-786.632574
Sum of electronic and thermal Energies=	-786.614705
Sum of electronic and thermal Enthalpies=	-786.613760
Sum of electronic and thermal Free Energies=	-786.678525

C	0.13884000	0.33942000	-0.12067200
C	-0.76460000	-0.82676500	0.00890900
C	-1.65734400	-1.26487200	-0.98702400
C	-0.75474500	-1.54333700	1.22388000
C	-2.51656800	-2.34679800	-0.76995700
H	-1.68663700	-0.77625300	-1.95764500

C	-1.59509600	-2.62917300	1.43263600
H	-0.07336200	-1.22526100	2.00808100
C	-2.49256300	-3.03623800	0.43877000
H	-3.19595800	-2.65427900	-1.56087600
H	-1.56221300	-3.15579400	2.38280700
H	-3.15885600	-3.87677700	0.60820000
C	1.61571100	0.06873400	-0.08834800
C	2.15649400	-1.21561500	-0.26508600
C	2.51012900	1.14001200	0.10339700
C	3.53586500	-1.42269900	-0.25003300
H	1.48691800	-2.05765700	-0.40942600
C	3.88784200	0.93543100	0.09925300
H	2.10531400	2.13183100	0.29061200
C	4.40898800	-0.34860000	-0.07575600
H	3.92911900	-2.42700100	-0.38542600
H	4.55735800	1.77814000	0.25157400
H	5.48328800	-0.51028600	-0.06841900
N	-0.17462300	1.14338000	-1.37247400
H	0.31364800	2.03847700	-1.28804000
H	-1.22089700	1.36559600	-1.40514800
H	0.11861900	0.70060100	-2.25352100
O	-2.74537300	3.13966200	0.91365500
H	-0.10891300	1.47391600	0.74958700
H	-2.78728400	4.08478500	0.70460400
O	-2.82660500	1.65918500	-1.27426000
H	-3.16591100	0.81024600	-0.94583300
H	-2.89290000	2.27056100	-0.47568500
O	-0.19684100	2.61677600	1.08387600
H	0.15440200	2.63975000	1.98757900
H	-1.76053200	2.96376500	1.08831100

TS2-N1

Zero-point correction= 0.406702 (Hartree/Particle)
Thermal correction to Energy= 0.455410
Thermal correction to Enthalpy= 0.456354
Thermal correction to Gibbs Free Energy= 0.313389
Sum of electronic and zero-point Energies= -1750.368154
Sum of electronic and thermal Energies= -1750.319445
Sum of electronic and thermal Enthalpies= -1750.318501
Sum of electronic and thermal Free Energies= -1750.461467

Ag	-0.64711900	0.71504900	-1.04741400
N	1.57426700	1.74259100	-0.53747500

N	1.96615000	1.19245900	0.65268500
N	-0.50215000	0.21414400	1.57266900
N	0.55762700	-0.65193500	1.61695200
N	0.87566900	-1.12031700	-1.28103100
N	1.95836200	-1.08087900	-0.44631700
B	1.89316600	-0.33185300	0.90562800
C	1.71734400	3.05392900	-0.39540400
C	2.18646500	3.39869200	0.87978400
C	2.32428500	2.16927900	1.51349700
C	-1.43615800	-0.32015900	2.34861100
C	-1.02309400	-1.53668800	2.91510400
C	0.25670400	-1.70735500	2.40764500
C	1.26401500	-1.78360800	-2.36056300
C	2.59882800	-2.19907600	-2.26707800
C	2.99907200	-1.72212000	-1.02494000
H	2.80516600	-0.67638700	1.58580900
Br	2.88333600	1.82233300	3.29542600
Br	2.56614200	5.12589800	1.58036500
Br	1.36264100	4.23401500	-1.86158800
Br	4.70767400	-1.90991300	-0.21639100
Br	3.61835000	-3.19539800	-3.52589400
Br	0.06711900	-2.08205300	-3.82370100
Br	1.44710000	-3.16810400	2.66523000
Br	-1.97369600	-2.67791800	4.09969500
Br	-3.11325900	0.55685600	2.61179800
C	-3.53183600	-0.26376800	-1.41329300
C	-4.71300200	0.59634600	-1.56736900
C	-5.03964200	1.23689800	-2.78443400
C	-5.52374200	0.91523700	-0.44817500
C	-6.10864900	2.13041500	-2.87730800
H	-4.43929600	1.08159300	-3.67651500
C	-6.58791500	1.80003400	-0.54895000
H	-5.29453000	0.46372100	0.51074400
C	-6.89606000	2.41864400	-1.76650800
H	-6.31784700	2.60515100	-3.83235000
H	-7.18479300	2.01278200	0.33440200
H	-7.72632800	3.11406500	-1.84026700
C	-3.60414900	-1.50870600	-0.58744300
C	-4.81434600	-2.09630300	-0.17498300
C	-2.41407700	-2.18938000	-0.25701700
C	-4.82934100	-3.28654500	0.55233700
H	-5.75381800	-1.61918500	-0.43157300
C	-2.42823200	-3.39476800	0.44038400
H	-1.45584300	-1.75116500	-0.52390100

C	-3.63913900	-3.94661300	0.86119300
H	-5.78115900	-3.70983900	0.86263700
H	-1.48892600	-3.88303900	0.68477400
H	-3.65286800	-4.87249200	1.42830400
N	-3.00889900	-0.65960200	-2.78302300
H	-2.33284600	-1.41863600	-2.66876900
H	-2.49529900	0.11533500	-3.24475000
H	-3.75449000	-1.00048400	-3.41661600
O	-2.08766500	3.76749300	-2.59092500
H	-2.89707500	1.43716300	-0.59587900
H	-1.27630000	4.23941900	-2.33778900
O	-1.29022700	1.40435700	-3.42826000
H	-0.47894700	1.44426400	-3.95648700
H	-1.57345000	2.35145600	-3.22260900
O	-2.42092700	2.28358500	-0.34995700
H	-2.42669300	2.32155900	0.62076800
H	-2.41577300	3.41161500	-1.73115300

33

Zero-point correction= 0.224439 (Hartree/Particle)
Thermal correction to Energy= 0.235851
Thermal correction to Enthalpy= 0.236795
Thermal correction to Gibbs Free Energy= 0.186765
Sum of electronic and zero-point Energies= -557.455623
Sum of electronic and thermal Energies= -557.444211
Sum of electronic and thermal Enthalpies= -557.443267
Sum of electronic and thermal Free Energies= -557.493297

C	0.01750800	1.18686900	-0.44629400
C	1.28370300	0.35782300	-0.25127300
C	2.45100700	0.93797300	0.25617900
C	1.30931200	-0.99346200	-0.62148900
C	3.61499300	0.18096300	0.40126700
H	2.43735700	1.98779000	0.52691300
C	2.47289000	-1.74777900	-0.48363200
H	0.40923300	-1.45803600	-1.01290900
C	3.63108900	-1.16377100	0.03301400
H	4.51171100	0.64671200	0.80152500
H	2.47316400	-2.79456600	-0.77543100
H	4.53708700	-1.75276800	0.14632800
C	-1.25620500	0.39415600	-0.17707700
C	-1.43757800	-0.27188300	1.04334900
C	-2.29172600	0.36802600	-1.11472900

C	-2.62800700	-0.93889800	1.31964800
H	-0.62997200	-0.27887300	1.77135400
C	-3.48737600	-0.30158300	-0.84329100
H	-2.16000900	0.87575000	-2.06783900
C	-3.65917600	-0.95498600	0.37583400
H	-2.75174700	-1.45307400	2.26892100
H	-4.28075600	-0.31354200	-1.58566500
H	-4.58687700	-1.47803900	0.59009600
N	0.08997600	2.43164000	0.34224900
H	-0.74968600	2.97858300	0.15608400
H	0.04929500	2.19328100	1.33387900
H	-0.01123900	1.50461000	-1.49803500

Int1-N'

Zero-point correction= 0.515308 (Hartree/Particle)
Thermal correction to Energy= 0.569280
Thermal correction to Enthalpy= 0.570224
Thermal correction to Gibbs Free Energy= 0.414805
Sum of electronic and zero-point Energies= -2022.093324
Sum of electronic and thermal Energies= -2022.039351
Sum of electronic and thermal Enthalpies= -2022.038407
Sum of electronic and thermal Free Energies= -2022.193827

Ag	0.10784600	-0.45569100	-1.38238100
N	0.58910800	-0.81999000	1.44480500
N	0.85778400	0.49502400	1.71077800
N	2.77908700	-0.11345500	-1.04525400
N	2.88647200	0.19425700	0.28313700
N	0.50042200	1.67459900	-1.06463600
N	1.35306000	2.17565200	-0.12146400
B	1.98434900	1.25519800	0.95214200
C	-0.36305100	-1.17486400	2.29734400
C	-0.75413300	-0.10575400	3.12409100
C	0.06455000	0.93703700	2.71332200
C	3.56171800	-1.16542500	-1.24194300
C	4.18169200	-1.58548200	-0.05258800
C	3.71607200	-0.68183000	0.89333700
C	0.12327100	2.68606500	-1.83510100
C	0.70514400	3.88555000	-1.40351700
C	1.48253600	3.50960500	-0.31478200
H	2.60321900	1.92240700	1.71852100
Br	0.13812100	2.71655600	3.38225600
Br	-2.11621300	-0.07197500	4.45308100

Br	-1.01465700	-2.97004200	2.35839900
Br	2.57174300	4.63968500	0.75447800
Br	0.46357000	5.62754400	-2.12573600
Br	-1.02619000	2.40459100	-3.32009300
Br	4.08487300	-0.62947700	2.75744500
Br	5.35781400	-3.05959700	0.21717300
Br	3.70636300	-1.95156700	-2.97761800
C	-0.70363900	-2.29883300	-1.93914200
C	-0.32012500	-3.49226400	-1.24061700
C	0.96936900	-3.56238000	-0.65323200
C	-1.20884900	-4.58467600	-1.04821400
C	1.39350800	-4.70995800	-0.00175600
H	1.62669500	-2.70772300	-0.73602000
C	-0.79952500	-5.70445000	-0.33730500
H	-2.23305000	-4.49704900	-1.39081300
C	0.50725700	-5.78081200	0.16093400
H	2.39627200	-4.75628500	0.41073700
H	-1.49494600	-6.52024400	-0.16466400
H	0.82508700	-6.66600600	0.70460200
C	-1.67165200	-2.37552000	-3.00442500
C	-2.46477200	-1.23781100	-3.30104800
C	-1.84124400	-3.53495600	-3.80531700
C	-3.43400200	-1.28269100	-4.29163000
H	-2.32387500	-0.33654500	-2.71424900
C	-2.77643800	-3.55604200	-4.83259600
H	-1.19869200	-4.39198500	-3.63867400
C	-3.58635000	-2.43981600	-5.06520600
H	-4.05716500	-0.41316500	-4.47754500
H	-2.87777500	-4.43989200	-5.45498200
H	-4.32368100	-2.46439000	-5.86251600
C	-4.28347900	-0.74900000	-0.42765200
C	-5.52226900	-0.91227100	0.44338500
C	-5.56171300	-0.36851500	1.73339200
C	-6.61495600	-1.65395200	-0.01288800
C	-6.67314900	-0.56820400	2.55132500
H	-4.72587000	0.22532900	2.09262500
C	-7.73177300	-1.85028100	0.80107200
H	-6.58533700	-2.08444200	-1.01084800
C	-7.76256700	-1.30863900	2.08668100
H	-6.69203400	-0.13738000	3.54865900
H	-8.57578200	-2.42641700	0.43166400
H	-8.63099400	-1.45910200	2.72184800
C	-3.80410700	0.70171500	-0.43925100
C	-2.71827300	1.13802500	0.32427900

C	-4.50394600	1.64119100	-1.20942500
C	-2.34604700	2.48539000	0.32931900
H	-2.16208700	0.42854100	0.92635400
C	-4.13492900	2.98417400	-1.20851500
H	-5.35657800	1.31198200	-1.79988000
C	-3.05189800	3.41208100	-0.43450500
H	-1.49919600	2.80608400	0.92574100
H	-4.68924600	3.69900900	-1.81054700
H	-2.75577300	4.45711300	-0.43672400
N	-3.29313500	-1.75022800	-0.01105400
H	-2.36099000	-1.47833900	-0.31841400
H	-3.25592900	-1.80546000	1.00370900
H	-4.57560800	-0.99411600	-1.45700300

TS1-N'

Zero-point correction=	0.514080 (Hartree/Particle)
Thermal correction to Energy=	0.567336
Thermal correction to Enthalpy=	0.568280
Thermal correction to Gibbs Free Energy=	0.415316
Sum of electronic and zero-point Energies=	-2022.082399
Sum of electronic and thermal Energies=	-2022.029143
Sum of electronic and thermal Enthalpies=	-2022.028199
Sum of electronic and thermal Free Energies=	-2022.181163

Ag	0.59611500	0.53740600	-0.40743000
N	-1.10725400	-0.09348300	1.78869500
N	-2.25882900	-0.69039500	1.35455300
N	-1.65244500	1.66899200	-0.98689900
N	-2.74309200	1.13786300	-0.35867700
N	-0.70180800	-1.31203700	-1.09582300
N	-2.06807300	-1.20118100	-1.12570300
B	-2.84242300	-0.37956700	-0.04935700
C	-0.84938400	-0.61649400	2.97661600
C	-1.81073500	-1.56713500	3.35466800
C	-2.68799800	-1.57939900	2.28301600
C	-1.90786600	2.95924300	-1.14942500
C	-3.16026300	3.31232900	-0.62601600
C	-3.65258100	2.11394500	-0.13228100
C	-0.37850700	-2.21535800	-2.01490600
C	-1.50931700	-2.70550600	-2.67941800
C	-2.55913700	-2.02837700	-2.07750400
H	-3.98130400	-0.70825500	-0.07473000
Br	-4.25518000	-2.65908500	2.08906900

Br	-1.90852100	-2.58887500	4.96495200
Br	0.67821000	-0.06274700	3.99488400
Br	-4.41390800	-2.19139900	-2.50230300
Br	-1.59424900	-4.00719900	-4.07068200
Br	1.43537900	-2.76139700	-2.27993400
Br	-5.32329900	1.82013900	0.74886500
Br	-4.00608000	5.02354500	-0.61110200
Br	-0.65715300	4.10509400	-2.03912800
C	2.39719000	1.70818600	-0.25778100
C	2.63001800	2.57525200	0.90036600
C	1.52127800	3.00874200	1.66074300
C	3.91623100	3.02267600	1.29291600
C	1.67834300	3.90474700	2.71725100
H	0.53142500	2.64839600	1.39910700
C	4.07546500	3.87465800	2.38043000
H	4.78916000	2.66474900	0.75804400
C	2.95584300	4.33329300	3.08572800
H	0.80684800	4.24748700	3.26732100
H	5.07197800	4.18748200	2.67863200
H	3.08206600	5.00953800	3.92645100
C	3.13398800	1.97950400	-1.49172100
C	3.15937200	1.00758100	-2.52340700
C	3.74135000	3.23152000	-1.76210100
C	3.79604200	1.24841000	-3.73415400
H	2.67023600	0.05550400	-2.34550200
C	4.34752200	3.48511400	-2.99170700
H	3.69867900	4.02194100	-1.02241000
C	4.39073900	2.49403500	-3.97489200
H	3.81393400	0.47837600	-4.50002200
H	4.78568100	4.46045500	-3.18255800
H	4.86871500	2.69357000	-4.92968500
C	4.99999500	-0.77454800	-0.07786500
C	6.27528700	-0.05190500	0.35763400
C	6.73133600	-0.12009400	1.68332200
C	7.01454800	0.69372000	-0.56980000
C	7.89291100	0.54831100	2.07177300
H	6.18230600	-0.70600600	2.41602400
C	8.18181700	1.35942300	-0.18396300
H	6.67426700	0.75506900	-1.60009600
C	8.62340500	1.28981200	1.13828500
H	8.23153600	0.48502300	3.10224800
H	8.74261000	1.93194800	-0.91764900
H	9.52991300	1.80677800	1.44030200
C	5.11569800	-2.29518000	0.08697800

C	4.67139400	-2.96167100	1.23708600
C	5.69680300	-3.04928800	-0.94368000
C	4.80872200	-4.34775500	1.35549500
H	4.20847000	-2.40164000	2.04479500
C	5.83501600	-4.43338100	-0.82785900
H	6.04471900	-2.54555500	-1.84274100
C	5.39049300	-5.08833000	0.32461600
H	4.45777200	-4.84728800	2.25442400
H	6.28661500	-5.00014400	-1.63756200
H	5.49416000	-6.16582100	0.41598700
N	3.82660200	-0.18332200	0.58619200
H	3.02789100	-0.81118300	0.56038400
H	4.00959900	0.05126900	1.55845300
H	4.87964400	-0.58615400	-1.15195500

Int2-N'

Zero-point correction=	0.520194 (Hartree/Particle)
Thermal correction to Energy=	0.572142
Thermal correction to Enthalpy=	0.573087
Thermal correction to Gibbs Free Energy=	0.426404
Sum of electronic and zero-point Energies=	-2022.115699
Sum of electronic and thermal Energies=	-2022.063751
Sum of electronic and thermal Enthalpies=	-2022.062807
Sum of electronic and thermal Free Energies=	-2022.209490

Ag	-0.49278400	-0.05993700	1.71729000
N	-0.24188700	-1.96369400	-0.61268500
N	-0.56649600	-1.07481900	-1.60008600
N	-2.47776600	-0.46630100	0.98279000
N	-2.71319400	-0.55591300	-0.36047900
N	0.25021000	1.57478900	-1.05456500
N	-1.09722600	1.32348300	-0.95936600
B	-1.69927600	-0.04433300	-1.41277500
C	0.69153700	-2.75297600	-1.12830900
C	1.01259200	-2.39285000	-2.45123200
C	0.17356800	-1.31678400	-2.70771900
C	-3.55133400	-0.93715300	1.60585800
C	-4.52071400	-1.35190700	0.68287900
C	-3.94001200	-1.09246900	-0.55219900
C	0.38618900	2.89105400	-0.93425100
C	-0.85082600	3.53105000	-0.75166700
C	-1.76563700	2.48886100	-0.78672600
H	-2.27576600	0.10509200	-2.44484700

Br	0.04104000	-0.28302200	-4.29630900
Br	2.30776200	-3.17059500	-3.60928500
Br	1.37093700	-4.24941300	-0.14961500
Br	-3.66036200	2.58997900	-0.65856600
Br	-1.20117800	5.38475900	-0.50449800
Br	2.10624200	3.70961000	-1.00713100
Br	-4.69756400	-1.41178900	-2.26303200
Br	-6.23728100	-2.08699000	1.03745400
Br	-3.61891000	-0.98736100	3.50413500
C	1.57584600	0.40238900	2.14258500
C	2.04613400	-0.62147800	3.14398500
C	2.28541100	-0.28925800	4.48754900
C	2.15630500	-1.97959000	2.79035900
C	2.66552300	-1.26137800	5.41621100
H	2.16505600	0.73579500	4.81762900
C	2.54974000	-2.94478100	3.70696000
H	1.93034800	-2.30494800	1.78241300
C	2.81833100	-2.59164100	5.03267100
H	2.84598900	-0.96650900	6.44660600
H	2.64285500	-3.97713500	3.38217600
H	3.12526300	-3.34380100	5.75332800
C	1.81300000	1.85832100	2.50093300
C	0.80846900	2.81548000	2.29644600
C	3.03765400	2.31572800	3.02473900
C	1.01518800	4.16749000	2.57085200
H	-0.15375400	2.49072800	1.91219400
C	3.25535500	3.66545900	3.29833900
H	3.81839100	1.60027100	3.25987000
C	2.24444800	4.59966000	3.06581400
H	0.21526500	4.87853100	2.38993600
H	4.21197400	3.98459800	3.70290500
H	2.41057200	5.65155600	3.27947000
C	3.74278500	0.32866200	0.54881800
C	4.48299600	-0.92588300	0.96639700
C	4.41546700	-2.08395200	0.18412700
C	5.22134500	-0.94398700	2.15254700
C	5.04116100	-3.25482300	0.60673800
H	3.87540200	-2.07685900	-0.75790400
C	5.85538600	-2.11123100	2.57264200
H	5.27707200	-0.04983700	2.76570800
C	5.75885500	-3.27234900	1.80427500
H	4.96993800	-4.15027800	-0.00339200
H	6.41149900	-2.11687300	3.50505900
H	6.24532600	-4.18567800	2.13405100

C	4.05859800	0.75611300	-0.86909300
C	3.33821600	0.30679600	-1.97744800
C	5.14150100	1.62085700	-1.06394900
C	3.68812400	0.72315500	-3.26281200
H	2.48589100	-0.35032600	-1.85656700
C	5.49423100	2.03579400	-2.34578200
H	5.70507600	1.97366400	-0.20387600
C	4.76484800	1.58816700	-3.45065800
H	3.11011700	0.37152300	-4.11073700
H	6.33381100	2.71118400	-2.48253300
H	5.03407100	1.91477200	-4.45067700
N	2.23499100	0.14816100	0.74670900
H	1.72032700	0.74547300	0.07557200
H	1.98531000	-0.80374400	0.46693800
H	4.01108900	1.14710400	1.21202600

Int3-N'

Zero-point correction=	0.595912 (Hartree/Particle)
Thermal correction to Energy=	0.654808
Thermal correction to Enthalpy=	0.655752
Thermal correction to Gibbs Free Energy=	0.494555
Sum of electronic and zero-point Energies=	-2251.343820
Sum of electronic and thermal Energies=	-2251.284925
Sum of electronic and thermal Enthalpies=	-2251.283981
Sum of electronic and thermal Free Energies=	-2251.445177

Ag	0.36795800	-0.66497000	-0.62945900
N	-1.79640700	-0.66123100	-1.42738900
N	-2.78789900	-0.69109700	-0.47849000
N	-1.30177100	-2.03895700	1.50067500
N	-1.59538700	-0.72305200	1.75545600
N	-0.69015400	1.59802000	0.24436500
N	-2.01340600	1.45920500	0.53900600
B	-2.59064000	0.06164300	0.86259400
C	-2.36248700	-1.03980000	-2.56802300
C	-3.71710200	-1.35385700	-2.39475500
C	-3.94042500	-1.11258800	-1.04580500
C	-0.66173800	-2.47952100	2.57824900
C	-0.51073000	-1.47820500	3.55105100
C	-1.13766000	-0.37952200	2.98221000
C	-0.51499000	2.85411300	-0.12730000
C	-1.71856100	3.57810800	-0.08769200
C	-2.64913100	2.63832000	0.33841400

H	-3.64243900	0.18106900	1.40526700
Br	-5.56997700	-1.32605700	-0.09662100
Br	-4.96519200	-1.94675600	-3.70010600
Br	-1.42193100	-0.98639600	-4.22670800
Br	-4.51753200	2.88123200	0.59880600
Br	-2.01079700	5.41133400	-0.50898000
Br	1.23065700	3.46870000	-0.62535600
Br	-1.43552300	1.33030800	3.76032400
Br	0.34735400	-1.58929200	5.24240500
Br	-0.12880000	-4.31112100	2.72761200
C	2.49867300	-0.77770400	-0.00845400
C	2.97696100	-2.16115100	-0.31198200
C	3.68822000	-2.53422600	-1.46128900
C	2.62979500	-3.19094900	0.59179200
C	4.01977600	-3.86927800	-1.70670800
H	3.98030500	-1.79976700	-2.19914900
C	2.96259200	-4.51891600	0.34878600
H	2.09553300	-2.93204000	1.49834900
C	3.66120800	-4.87235700	-0.81204300
H	4.55943800	-4.11560400	-2.61658100
H	2.67653900	-5.27856900	1.07163900
H	3.91889000	-5.90865100	-1.00766300
C	2.75266300	-0.33052600	1.42209100
C	3.81881100	-0.85395500	2.17720300
C	1.98913900	0.69351200	2.00016500
C	4.11429700	-0.36462200	3.45021100
H	4.41826500	-1.66034200	1.76752600
C	2.29191000	1.19816100	3.26409800
H	1.14034100	1.08940900	1.45721100
C	3.35711700	0.67330600	3.99638100
H	4.94089200	-0.79227000	4.01117300
H	1.67863300	1.98988700	3.68242100
H	3.58480400	1.05708700	4.98670200
N	3.15021200	0.26620200	-0.93079900
H	2.64079700	1.13498800	-0.75050500
H	2.88852000	0.01351200	-1.91123200
O	1.12381400	-2.93853800	-3.17574300
H	0.56887200	-3.87378600	-0.30830400
H	0.41950400	-3.08598100	-3.82540300
O	1.96939400	-0.37257500	-3.38171700
H	1.12430700	0.10372200	-3.39150500
H	1.70032800	-1.33299300	-3.30716200
O	-0.21064800	-3.64805800	-0.84851200
H	-0.77364000	-3.11830900	-0.24768500

H	0.68247400	-3.11664900	-2.31003300
C	4.64771800	0.59925200	-0.76416000
H	4.96584800	0.00576700	0.09158600
C	4.75025200	2.06911500	-0.38791300
C	4.70062500	3.07833200	-1.35827100
C	4.80221900	2.42519500	0.96473600
C	4.68641300	4.42128300	-0.97916300
H	4.68696800	2.81849000	-2.41151300
C	4.79513900	3.76763700	1.34245700
H	4.83217100	1.65167800	1.72435200
C	4.73083400	4.76920900	0.37324000
H	4.64916300	5.19472800	-1.74086100
H	4.83124300	4.02697200	2.39626700
H	4.71963900	5.81467000	0.66692700
C	5.48589300	0.20209000	-1.95808600
C	6.74408900	-0.35990000	-1.71095800
C	5.08206800	0.40095700	-3.28429200
C	7.58600200	-0.71441300	-2.76265300
H	7.06104100	-0.52553000	-0.68460900
C	5.92136200	0.03625900	-4.33820200
H	4.09903200	0.79660600	-3.51355500
C	7.17530200	-0.51808000	-4.08247800
H	8.55674500	-1.15331500	-2.55155200
H	5.58664900	0.18267300	-5.36082900
H	7.82499200	-0.80235000	-4.90496100

Int3-N''

Zero-point correction=	0.745174 (Hartree/Particle)
Thermal correction to Energy=	0.808882
Thermal correction to Enthalpy=	0.809826
Thermal correction to Gibbs Free Energy=	0.636626
Sum of electronic and zero-point Energies=	-2579.601082
Sum of electronic and thermal Energies=	-2579.537374
Sum of electronic and thermal Enthalpies=	-2579.536430
Sum of electronic and thermal Free Energies=	-2579.709630

Ag	0.23815100	0.54956700	0.96622600
N	-1.68183900	-0.38360200	1.52624400
N	-2.88580700	-0.15416200	0.92392800
N	-1.71675200	2.47017500	0.89525600
N	-2.42911900	2.08158500	-0.20708700
N	-0.77780200	0.27728000	-1.59281100
N	-2.10374700	-0.03305300	-1.48594700

B	-2.97937900	0.65029500	-0.39351100
C	-1.91255400	-1.10749800	2.61230400
C	-3.27886900	-1.38940100	2.74377300
C	-3.85695200	-0.76250300	1.64744200
C	-1.31260100	3.70923400	0.64970900
C	-1.73147500	4.15722900	-0.61513500
C	-2.44025900	3.07830900	-1.12237800
C	-0.31608200	-0.42949200	-2.61433600
C	-1.31910600	-1.22252400	-3.19783900
C	-2.44593900	-0.92971400	-2.43903500
H	-4.11029600	0.66817500	-0.75914900
Br	-5.69934800	-0.75122300	1.17973900
Br	-4.13409300	-2.43320000	4.08186300
Br	-0.47988200	-1.59585100	3.76253300
Br	-4.18923200	-1.66981300	-2.59560800
Br	-1.18306500	-2.41294000	-4.67864500
Br	1.52782600	-0.32791900	-3.10827000
Br	-3.29311800	2.91450900	-2.81732500
Br	-1.41851000	5.84242300	-1.44507500
Br	-0.29511500	4.67931800	1.93974300
C	2.34834400	1.10819900	1.11010100
C	2.61223300	0.87959800	2.56990800
C	3.27775700	-0.23654800	3.10449600
C	2.03478100	1.77960200	3.49457900
C	3.35688500	-0.44813600	4.48311500
H	3.75553300	-0.95751000	2.45706000
C	2.11747900	1.57050700	4.86530800
H	1.48319800	2.63530200	3.12055100
C	2.78062900	0.44994500	5.37802300
H	3.89389400	-1.31933300	4.84983700
H	1.65001300	2.28378300	5.53881800
H	2.84618900	0.28650700	6.44953400
C	2.52073000	2.51621200	0.58571700
C	3.35332900	3.45776900	1.21188600
C	1.88488100	2.89557300	-0.60968200
C	3.53349000	4.73211100	0.67127600
H	3.84638300	3.19429100	2.14216000
C	2.07626700	4.16029400	-1.16131600
H	1.20307800	2.20076400	-1.09304500
C	2.89962700	5.08806400	-0.51958400
H	4.17372300	5.44616900	1.18256400
H	1.55689100	4.43009100	-2.07569100
H	3.03381000	6.08157700	-0.93727100
C	4.62013600	0.50726300	-0.13000700

C	5.55622400	-0.06892900	0.90915300
C	5.85956200	-1.43547300	0.95920000
C	6.10364200	0.78455900	1.87137100
C	6.65990000	-1.94043400	1.98255500
H	5.47148100	-2.10821900	0.20277500
C	6.91248700	0.28279700	2.88958500
H	5.86904100	1.84421000	1.83668900
C	7.18321100	-1.08402000	2.95441400
H	6.88244100	-3.00327300	2.01719000
H	7.31990700	0.95684500	3.63701900
H	7.80556700	-1.48007600	3.75150400
C	4.92643700	0.09980800	-1.55582800
C	4.57716100	-1.15820600	-2.06217600
C	5.56825200	1.01489400	-2.39785500
C	4.87240900	-1.49987000	-3.38252700
H	4.04915800	-1.87760400	-1.44783900
C	5.86680600	0.67540000	-3.71733000
H	5.82796500	1.99949200	-2.01798100
C	5.52004500	-0.58371300	-4.21292500
H	4.58476000	-2.47948700	-3.75104900
H	6.36221800	1.39716800	-4.36000600
H	5.74797800	-0.84607500	-5.24183200
N	3.15014700	0.18342300	0.18327200
H	2.66491500	0.22507000	-0.71667600
H	3.01054800	-0.81067500	0.50495400
H	4.66875000	1.59437000	-0.07151500
C	1.27610800	-2.87321000	-0.05000000
C	2.11205700	-3.71351900	-1.00105300
C	3.37908700	-4.20493100	-0.67186700
C	1.58796500	-3.99963100	-2.26816500
C	4.11356400	-4.95601400	-1.59491600
H	3.82145100	-3.98420300	0.29438900
C	2.31783300	-4.74274200	-3.19123500
H	0.60262100	-3.62953700	-2.53261200
C	3.58780700	-5.22428200	-2.85775500
H	5.09916900	-5.32417700	-1.32429800
H	1.89635100	-4.94237200	-4.17229200
H	4.15961300	-5.80446500	-3.57619000
C	0.03759900	-3.61633900	0.43407100
C	-1.22899700	-3.18367200	0.03597600
C	0.14091700	-4.71488700	1.29472400
C	-2.38061200	-3.82034400	0.50188700
H	-1.31298300	-2.33397200	-0.62907200
C	-1.00575800	-5.34689300	1.77205000

H	1.12142600	-5.07911800	1.59379400
C	-2.27089200	-4.89792100	1.38011600
H	-3.35689800	-3.46425200	0.18739700
H	-0.91473600	-6.19333400	2.44683100
H	-3.16376200	-5.38816100	1.75625500
N	2.09752000	-2.31960800	1.04452000
H	1.47916600	-1.80805600	1.67667600
H	2.49322100	-3.06768700	1.61228600
H	0.91930200	-2.00244900	-0.61064100

Int4-N'

Zero-point correction= 0.487074 (Hartree/Particle)
Thermal correction to Energy= 0.515979
Thermal correction to Enthalpy= 0.516923
Thermal correction to Gibbs Free Energy= 0.427273
Sum of electronic and zero-point Energies= -1287.585554
Sum of electronic and thermal Energies= -1287.556649
Sum of electronic and thermal Enthalpies= -1287.555705
Sum of electronic and thermal Free Energies= -1287.645355

C	1.03059100	0.86696000	0.65447900
C	1.99194400	-0.26811200	0.49047000
C	2.52380500	-0.65295800	-0.75218000
C	2.36965300	-1.02503700	1.61576900
C	3.34159200	-1.77345500	-0.87745500
H	2.26730100	-0.07751800	-1.63371000
C	3.17643800	-2.15853400	1.49478900
H	2.03795000	-0.70733100	2.59985500
C	3.65908200	-2.54322600	0.24319900
H	3.71408700	-2.05822700	-1.85778500
H	3.44741700	-2.72412100	2.38286400
H	4.28890800	-3.42309800	0.14444900
C	1.18350800	2.10755000	-0.09833100
C	2.45268200	2.51921300	-0.57645800
C	0.12154500	3.03064400	-0.27991200
C	2.63590300	3.74440500	-1.20547000
H	3.30486000	1.86323300	-0.43467900
C	0.31288600	4.25657700	-0.91392200
H	-0.87299900	2.80962300	0.10002000
C	1.56840400	4.62777500	-1.39263400
H	3.62943200	4.01260100	-1.55736000
H	-0.53497300	4.92784300	-1.02974300
H	1.71484700	5.58105600	-1.89144300

N	-0.39449700	0.37065000	0.70725000
H	-0.93593100	1.04305200	1.26365300
H	-0.41204700	-0.50304000	1.28676200
O	0.27157400	-0.50465000	4.52327400
H	0.84527900	1.42947100	2.21496500
H	-0.38705000	-0.40765700	5.22674000
O	-0.49533900	-1.88672400	2.36726100
H	0.34481400	-2.30434400	2.11314500
H	-0.28779600	-1.45174300	3.24377600
O	0.34284000	1.72581800	3.10347100
H	0.80944400	2.52059600	3.40270500
H	0.33354900	0.39908000	4.09258700
C	-1.16868600	0.13561800	-0.59307100
H	-0.97483000	1.02762400	-1.18754200
C	-2.65328100	0.07952200	-0.29560400
C	-3.52111500	0.85060000	-1.07742000
C	-3.18416200	-0.71476700	0.73058600
C	-4.89675700	0.82219800	-0.85387800
H	-3.11280000	1.47473900	-1.86842800
C	-4.56029000	-0.73530000	0.95777500
H	-2.53125100	-1.31428700	1.35760800
C	-5.42009500	0.02780400	0.16674200
H	-5.55690800	1.42435500	-1.47161800
H	-4.96061500	-1.35277300	1.75695100
H	-6.49102200	0.00668500	0.34726400
C	-0.63038500	-1.05093500	-1.36223100
C	-0.15719000	-0.83893100	-2.66048300
C	-0.60069600	-2.34563400	-0.83136700
C	0.36229100	-1.89268400	-3.41139500
H	-0.17510600	0.16516800	-3.07634900
C	-0.06615200	-3.39563800	-1.57384300
H	-0.98171400	-2.53612500	0.16526500
C	0.41982400	-3.17332100	-2.86385200
H	0.73202100	-1.70915400	-4.41621400
H	-0.03022800	-4.39232700	-1.14350600
H	0.83621100	-3.99548200	-3.43884800

TS2-N'

Zero-point correction= 0.484722 (Hartree/Particle)
Thermal correction to Energy= 0.512803
Thermal correction to Enthalpy= 0.513747
Thermal correction to Gibbs Free Energy= 0.427007
Sum of electronic and zero-point Energies= -1287.583519

Sum of electronic and thermal Energies= -1287.555438
Sum of electronic and thermal Enthalpies= -1287.554494
Sum of electronic and thermal Free Energies= -1287.641233

C	1.04849600	0.81614200	0.77237300
C	2.01432800	-0.30964000	0.52352800
C	2.48114000	-0.66301000	-0.75264200
C	2.44716500	-1.08560000	1.61359500
C	3.30412600	-1.77130800	-0.94206600
H	2.17551700	-0.07488700	-1.60921400
C	3.26309400	-2.20402400	1.42860200
H	2.13899000	-0.80632300	2.61657800
C	3.68875400	-2.55541900	0.14662100
H	3.62935100	-2.03200300	-1.94548000
H	3.57862300	-2.78813500	2.28925600
H	4.32365600	-3.42483400	-0.00088300
C	1.20324800	2.09634300	0.05613500
C	2.44572300	2.49028100	-0.48608200
C	0.16082300	3.05141100	0.00416000
C	2.62335300	3.74234400	-1.06763900
H	3.28441500	1.80480000	-0.43760500
C	0.34017600	4.30008300	-0.58655600
H	-0.80405000	2.83391400	0.45498600
C	1.57121500	4.65750800	-1.13633100
H	3.59708600	4.00257900	-1.47542500
H	-0.49099200	5.00069000	-0.60776100
H	1.71049000	5.63013800	-1.59882700
N	-0.38862900	0.30034400	0.75291900
H	-0.90569900	0.89660300	1.41341100
H	-0.38336900	-0.63715000	1.22801500
O	0.29315000	-0.89570800	4.40509800
H	0.87896900	1.17569700	2.17876300
H	-0.37177100	-0.87835800	5.10907900
O	-0.41946700	-2.10860900	2.15838100
H	0.43186200	-2.48792500	1.88300300
H	-0.22635000	-1.73764700	3.07262800
O	0.27778400	1.38867600	3.18362600
H	0.71775800	2.14846700	3.59307500
H	0.31189400	0.05020100	4.02948000
C	-1.17686800	0.22008700	-0.54272000
H	-1.00392700	1.17604100	-1.03600700
C	-2.65741600	0.11217200	-0.23071400
C	-3.54911200	0.92844200	-0.93535600
C	-3.15977100	-0.78077800	0.72588300

C	-4.92124300	0.85125900	-0.70211800
H	-3.16280400	1.62704200	-1.67343700
C	-4.53259800	-0.85068000	0.96316000
H	-2.48817300	-1.42087400	1.29010500
C	-5.41662000	-0.03986700	0.25030400
H	-5.60050200	1.49080000	-1.25851600
H	-4.91098900	-1.54424100	1.70875200
H	-6.48475900	-0.09934300	0.43854100
C	-0.65457200	-0.86737700	-1.45794100
C	-0.20796500	-0.50687000	-2.73244500
C	-0.61802100	-2.21422100	-1.07913200
C	0.29250400	-1.46828000	-3.61028700
H	-0.23088000	0.53850900	-3.02943400
C	-0.10515300	-3.17274500	-1.94952200
H	-0.97344400	-2.51595100	-0.10036900
C	0.35455400	-2.80397800	-3.21554900
H	0.64214000	-1.17100600	-4.59487200
H	-0.06442600	-4.21246200	-1.63780700
H	0.75388500	-3.55526600	-3.89089200

TS2-N"

Zero-point correction= 0.596353 (Hartree/Particle)
Thermal correction to Energy= 0.654480
Thermal correction to Enthalpy= 0.655424
Thermal correction to Gibbs Free Energy= 0.496422
Sum of electronic and zero-point Energies= -2251.322753
Sum of electronic and thermal Energies= -2251.264625
Sum of electronic and thermal Enthalpies= -2251.263681
Sum of electronic and thermal Free Energies= -2251.422684

Ag	0.34609100	-0.76979600	-1.06510100
N	-1.94737400	-1.20400500	-1.86347400
N	-2.80682300	-0.80597300	-0.87693000
N	-1.64596700	-0.66511300	1.71525600
N	-2.15561100	0.49257000	1.19484000
N	-0.45803800	1.36856300	-1.05402800
N	-1.81726800	1.52302800	-1.08977900
B	-2.73269800	0.60328800	-0.24442300
C	-2.31441100	-2.43637800	-2.19026300
C	-3.40616300	-2.87974400	-1.43206000
C	-3.68435000	-1.79659000	-0.60589800
C	-1.38888700	-0.41331700	2.99274500
C	-1.72513500	0.90365600	3.34526500

C	-2.20511300	1.43991800	2.16120600
C	0.06270200	2.37454200	-1.74709300
C	-0.93726400	3.21246500	-2.25522000
C	-2.11816800	2.62614700	-1.81199200
H	-3.82125100	1.08609000	-0.20010300
Br	-5.03744900	-1.63944500	0.71541000
Br	-4.30388400	-4.55457900	-1.50893200
Br	-1.39364400	-3.38080700	-3.57917400
Br	-3.88782200	3.23471700	-2.13170100
Br	-0.73002600	4.79440200	-3.29077200
Br	1.95620400	2.54815300	-1.90644800
Br	-2.80469800	3.21554700	1.83135700
Br	-1.57049700	1.75709000	5.03606200
Br	-0.62968300	-1.75161000	4.11960500
C	2.63355400	-1.02611000	1.07602400
C	3.19310800	-2.33664000	1.42464800
C	3.98456400	-3.10476300	0.54027500
C	2.83606200	-2.96091800	2.64730100
C	4.40488900	-4.39404400	0.86328100
H	4.27356500	-2.71471400	-0.42697900
C	3.25898200	-4.24532900	2.96350400
H	2.19446500	-2.43103500	3.34162300
C	4.05605900	-4.97870000	2.07868200
H	5.01281800	-4.94103600	0.14723000
H	2.95815900	-4.68067000	3.91336700
H	4.38611100	-5.98229800	2.32898500
C	2.40589800	0.01099100	2.12338400
C	3.11578200	0.05098800	3.33925900
C	1.50791400	1.06574000	1.86792400
C	2.94441100	1.10039700	4.24352400
H	3.81698700	-0.74405100	3.57087400
C	1.35994700	2.13087000	2.75102800
H	0.90770000	1.04339900	0.96379500
C	2.07772200	2.15493100	3.94912300
H	3.50620400	1.10234700	5.17408000
H	0.66034000	2.92823200	2.51670700
H	1.94605800	2.97245900	4.65152500
N	3.34457100	-0.38266500	-0.08797200
H	2.82700000	0.46682400	-0.32242600
H	3.25200400	-0.98886100	-0.91565100
O	1.23484000	-4.20947000	-1.31801400
H	0.93591900	-2.06844700	0.90425700
H	0.45988500	-4.42491700	-1.86354400
O	2.13159800	-1.90161500	-2.20443300

H	2.02791500	-1.86257500	-3.16736900
H	1.87593200	-2.84109300	-1.92328100
O	0.13784700	-2.56053900	0.56068300
H	-0.64016200	-2.14668500	0.99873500
H	0.83923300	-3.84530400	-0.49059100
C	4.84402600	0.05291100	0.09136200
H	5.06633300	-0.23222600	1.11987600
C	4.92925200	1.56501800	-0.03706200
C	5.28434800	2.19743000	-1.23683100
C	4.58768700	2.35743700	1.06800500
C	5.27311800	3.59047400	-1.33429700
H	5.59151200	1.60753700	-2.09256100
C	4.57674400	3.74747700	0.96919700
H	4.31960400	1.88598600	2.00646100
C	4.91270700	4.37005200	-0.23376900
H	5.55501900	4.06418800	-2.27033000
H	4.30097600	4.33976700	1.83635400
H	4.90191500	5.45313500	-0.31232600
C	5.73852600	-0.72033700	-0.84403500
C	6.90930400	-1.29927200	-0.34265000
C	5.43321900	-0.88056300	-2.20274400
C	7.75933800	-2.02155000	-1.17707800
H	7.14343500	-1.19622100	0.71310500
C	6.27913700	-1.61115200	-3.03809900
H	4.52742100	-0.44594900	-2.61610100
C	7.44496900	-2.18259600	-2.52820300
H	8.66095500	-2.46922900	-0.76973800
H	6.02603600	-1.73147300	-4.08749600
H	8.10207300	-2.75306900	-3.17774400

TS2-N'''

Zero-point correction=	0.739711 (Hartree/Particle)
Thermal correction to Energy=	0.804954
Thermal correction to Enthalpy=	0.805898
Thermal correction to Gibbs Free Energy=	0.628605
Sum of electronic and zero-point Energies=	-2579.559637
Sum of electronic and thermal Energies=	-2579.494394
Sum of electronic and thermal Enthalpies=	-2579.493450
Sum of electronic and thermal Free Energies=	-2579.670742

Ag	0.19087500	1.07869100	0.28193900
N	-1.47907700	-0.19265200	1.52049100
N	-2.62901900	-0.55062700	0.87280000

N	-1.79090300	2.13429700	-0.31106900
N	-2.85054300	1.39005500	-0.75076300
N	-0.58501900	-0.34893000	-1.69302800
N	-1.86093000	-0.81954200	-1.54915400
B	-2.89176800	-0.14630000	-0.60111400
C	-1.62014000	-0.56614500	2.78349000
C	-2.85665200	-1.19150400	2.99890400
C	-3.46433200	-1.15735600	1.75232400
C	-2.09128700	3.40612600	-0.52051200
C	-3.35835800	3.53474400	-1.11153300
C	-3.80182500	2.22596500	-1.23775500
C	0.02331700	-1.16328500	-2.54718200
C	-0.82987800	-2.19515500	-2.97022700
C	-2.02260300	-1.92963800	-2.30945200
H	-3.96960700	-0.52636900	-0.90146300
Br	-5.17438400	-1.85643900	1.29767300
Br	-3.53802400	-1.92712700	4.61391500
Br	-0.26953300	-0.17250900	4.07006700
Br	-3.62115800	-2.95818900	-2.36684300
Br	-0.43923000	-3.63781700	-4.14852000
Br	1.84817200	-0.90635900	-3.04348000
Br	-5.45824000	1.64055300	-1.97039600
Br	-4.26168000	5.13137300	-1.61672000
Br	-0.85868600	4.76653500	-0.03066000
C	2.64209300	1.34662400	0.68097500
C	2.90889500	1.54804800	2.15749800
C	3.93672200	0.88434200	2.84099500
C	2.02108000	2.32724500	2.92290200
C	4.08503400	1.00614300	4.22492900
H	4.64120900	0.28259900	2.28283800
C	2.17580500	2.46691100	4.29857100
H	1.18422100	2.81719600	2.43064300
C	3.21196400	1.80302700	4.96394600
H	4.90259300	0.48639900	4.71758500
H	1.47292500	3.08041000	4.85570900
H	3.33276800	1.90837200	6.03827800
C	2.46560300	2.65298100	-0.10403300
C	2.83134000	3.90553100	0.41494500
C	2.07868300	2.61097500	-1.45939100
C	2.78549900	5.06046600	-0.37131600
H	3.16905900	3.98198400	1.44138700
C	2.03436700	3.75837400	-2.24613300
H	1.82805900	1.65785900	-1.91190200
C	2.38689900	4.99742100	-1.70562300

H	3.07968300	6.01167300	0.06498700
H	1.72131700	3.68115300	-3.28365500
H	2.35270900	5.89531300	-2.31541500
C	4.82022600	1.05530200	-0.63747600
C	5.95160800	1.23219300	0.36432800
C	6.70612000	0.14543600	0.82299300
C	6.20382200	2.50017000	0.89364400
C	7.67119300	0.32152600	1.81291800
H	6.53038400	-0.84230300	0.40876800
C	7.16915900	2.68085900	1.88541000
H	5.61774800	3.34554100	0.54359000
C	7.90147800	1.59012000	2.35290600
H	8.24797900	-0.53072800	2.16261000
H	7.34343400	3.67149200	2.29616000
H	8.65128500	1.72594000	3.12727300
C	5.21047600	0.21052200	-1.83772600
C	5.13610500	-1.18952100	-1.81859300
C	5.63622500	0.84462300	-3.01096700
C	5.48201500	-1.93398800	-2.94788000
H	4.78772600	-1.70173000	-0.92894300
C	5.98357700	0.10280800	-4.13995800
H	5.68711400	1.93009900	-3.03804500
C	5.90651000	-1.29167200	-4.11190600
H	5.41018400	-3.01628100	-2.91069900
H	6.30762300	0.61339800	-5.04252100
H	6.17266300	-1.87157400	-4.99110900
N	3.62171000	0.45319000	0.00361400
H	3.12136800	-0.02970100	-0.73663100
H	2.99405400	-1.15348600	1.59018600
H	4.58389000	2.05393700	-1.02297400
C	1.48106900	-2.28683200	0.68588900
C	2.61122000	-3.20015200	0.23119100
C	3.75222200	-3.43201900	1.00652200
C	2.48385700	-3.85755600	-0.99700800
C	4.76077300	-4.28200500	0.54813600
H	3.86936100	-2.95467600	1.97589900
C	3.48404500	-4.71339500	-1.45267700
H	1.59894700	-3.69368100	-1.60340500
C	4.63159700	-4.92281800	-0.68423700
H	5.64529000	-4.44293200	1.15749200
H	3.37016900	-5.20727400	-2.41330000
H	5.41733400	-5.58168300	-1.04180600
C	0.38415300	-3.06790900	1.39099900
C	-0.80291100	-3.34569700	0.70965800

C	0.54814300	-3.54375500	2.69593000
C	-1.82226200	-4.07214100	1.32421300
H	-0.93162300	-2.98363000	-0.30300000
C	-0.47296200	-4.26199200	3.31704100
H	1.47438200	-3.35531000	3.23323200
C	-1.66116600	-4.52730100	2.63307500
H	-2.74402100	-4.26621800	0.78418800
H	-0.34057800	-4.61462000	4.33557000
H	-2.45854900	-5.07901500	3.12111900
N	1.97806900	-1.16139700	1.51608400
H	1.84247900	0.07022300	0.99629300
H	1.56527000	-1.13871300	2.44672300
H	1.03446600	-1.84202400	-0.20684600

33''

Zero-point correction=	0.412035 (Hartree/Particle)
Thermal correction to Energy=	0.433716
Thermal correction to Enthalpy=	0.434660
Thermal correction to Gibbs Free Energy=	0.361403
Sum of electronic and zero-point Energies=	-1058.402528
Sum of electronic and thermal Energies=	-1058.380847
Sum of electronic and thermal Enthalpies=	-1058.379903
Sum of electronic and thermal Free Energies=	-1058.453160

C	-0.97799500	0.95147400	-1.15974500
C	-2.22231100	0.05987400	-1.10985600
C	-2.17905500	-1.32792600	-0.97345600
C	-3.47470900	0.67515700	-1.26457200
C	-3.35577100	-2.08233800	-0.96776000
H	-1.23051200	-1.83433200	-0.86098400
C	-4.64862600	-0.07185600	-1.25914800
H	-3.52401000	1.75549100	-1.37964900
C	-4.59366200	-1.46036900	-1.10644500
H	-3.29382900	-3.16063100	-0.84918800
H	-5.60690400	0.42716100	-1.37610300
H	-5.50802100	-2.04715200	-1.10021600
C	-0.95389800	2.01388600	-0.06467200
C	-1.67082100	1.88445800	1.12825800
C	-0.13774700	3.13781600	-0.24352300
C	-1.57245900	2.85845800	2.12421900
H	-2.30988100	1.02057500	1.27551300
C	-0.03623200	4.11032500	0.74876400
H	0.43391400	3.23276300	-1.16274100

C	-0.75516100	3.97289200	1.93857000
H	-2.13683300	2.74366200	3.04562700
H	0.60211900	4.97579500	0.59426800
H	-0.67929000	4.73074700	2.71321600
C	1.11128700	-0.03852200	-0.04026700
C	0.68517400	-1.34054900	0.62751800
C	1.01521300	-2.57693100	0.05607700
C	-0.11883300	-1.32051600	1.76970100
C	0.51833700	-3.76304700	0.59264800
H	1.66536900	-2.60446400	-0.81480900
C	-0.61537300	-2.50628800	2.31313800
H	-0.37433600	-0.36609500	2.22133400
C	-0.30606900	-3.73033400	1.72075800
H	0.77679000	-4.71404700	0.13470200
H	-1.24758400	-2.47273600	3.19620600
H	-0.69585200	-4.65423100	2.13866600
C	2.60950000	-0.00960500	-0.35115800
C	3.10189700	0.59406500	-1.51152000
C	3.52420100	-0.54332300	0.56734900
C	4.47544500	0.65390200	-1.75564400
H	2.39430400	1.01714700	-2.21532600
C	4.89536900	-0.47790900	0.32912400
H	3.15698700	-1.01754600	1.47276700
C	5.37784300	0.11863200	-0.83772600
H	4.83902000	1.12260400	-2.66650400
H	5.58802900	-0.89695400	1.05415900
H	6.44671900	0.16493900	-1.02744200
N	0.33603900	0.29784300	-1.23989900
H	-1.06807900	1.50589600	-2.10694000
H	0.32399000	-0.47040900	-1.90442900
H	0.92235900	0.76835400	0.67336800

Int1-O

Zero-point correction= 0.315402 (Hartree/Particle)
Thermal correction to Energy= 0.358188
Thermal correction to Enthalpy= 0.359133
Thermal correction to Gibbs Free Energy= 0.226712
Sum of electronic and zero-point Energies= -1541.027268
Sum of electronic and thermal Energies= -1540.984482
Sum of electronic and thermal Enthalpies= -1540.983538
Sum of electronic and thermal Free Energies= -1541.115959

Ag	0.85724400	-0.21976400	1.26793600

N	0.75795200	-0.50265900	-1.96290600
N	-0.47148000	-0.85272100	-1.51272100
N	-1.41559400	-0.57471400	1.21637000
N	-2.19070400	-0.15388400	0.19249800
N	0.02374300	1.79042000	-0.04415800
N	-0.85533300	1.59039300	-1.05015000
B	-1.55604700	0.22336400	-1.17724300
C	1.33539600	-1.62146100	-2.35904200
C	0.50452900	-2.73365400	-2.17258400
C	-0.64337300	-2.18434800	-1.63805400
C	-2.22521200	-0.87426500	2.21429300
C	-3.56182100	-0.65847700	1.86652900
C	-3.48289000	-0.19721800	0.56563600
C	0.45263200	3.02941500	-0.16606700
C	-0.13162100	3.67738500	-1.26022100
C	-0.96120200	2.70943000	-1.79316800
H	-2.40238100	0.25607200	-2.01483000
Br	-2.24868100	-3.07969400	-1.18868100
Br	0.85424900	-4.54905000	-2.56188700
Br	3.08630700	-1.61753900	-3.08884700
Br	-2.08453800	2.87089100	-3.30389000
Br	0.13699500	5.44634900	-1.85862600
Br	1.72114600	3.73022400	1.06391100
Br	-4.91671200	0.29775100	-0.55906600
Br	-5.10492100	-0.93366500	2.91628700
Br	-1.53187400	-1.49391400	3.86390200
C	2.95280500	-0.47332600	1.78991500
C	3.74856800	-1.58877800	1.35292700
C	3.16782400	-2.52804700	0.47161300
C	5.06663000	-1.82664300	1.81161800
C	3.89018200	-3.61389400	0.00573000
H	2.14050500	-2.37242300	0.14792300
C	5.77326300	-2.93769900	1.37883500
H	5.50959000	-1.15170600	2.53616500
C	5.19255000	-3.81937200	0.46471000
H	3.43827600	-4.30545600	-0.69778600
H	6.77553000	-3.12323800	1.75039300
H	5.75289400	-4.68398400	0.12192700
C	3.60914300	0.66788700	2.40162300
C	4.80069000	1.21698500	1.88250500
C	2.98308200	1.30930300	3.48811300
C	5.34105300	2.36851700	2.44054800
H	5.24295500	0.76917300	0.99772300
C	3.56794400	2.41581900	4.08827500

H	2.03861600	0.91493900	3.85539500
C	4.73972300	2.95528900	3.55503400
H	6.23491100	2.80876600	2.01067000
H	3.09274200	2.88311500	4.94450800
H	5.17549300	3.84522600	3.99852400
O	3.08744900	0.83556800	-0.63264900
H	2.92108700	1.70849600	-0.24467800
H	2.29154700	0.64621900	-1.16526500

TS1-O

Zero-point correction= 0.316544 (Hartree/Particle)
Thermal correction to Energy= 0.358729
Thermal correction to Enthalpy= 0.359673
Thermal correction to Gibbs Free Energy= 0.230020
Sum of electronic and zero-point Energies= -1541.017669
Sum of electronic and thermal Energies= -1540.975484
Sum of electronic and thermal Enthalpies= -1540.974540
Sum of electronic and thermal Free Energies= -1541.104192

Ag	0.72937500	-0.24943700	1.49401100
N	0.85384100	-0.67689300	-1.76773300
N	-0.42410100	-0.90319800	-1.36974100
N	-1.51626000	-0.38375000	1.31393600
N	-2.17961900	0.02447400	0.20872300
N	0.20650900	1.80568800	-0.06562200
N	-0.65951200	1.59168400	-1.07966100
B	-1.44081200	0.26814200	-1.14124000
C	1.32352100	-1.84141300	-2.17947200
C	0.37369800	-2.86181700	-2.05477200
C	-0.72699800	-2.20569600	-1.54111200
C	-2.41623200	-0.56358900	2.26284900
C	-3.70314800	-0.27735000	1.80073300
C	-3.49546000	0.09467700	0.48533500
C	0.72891700	2.99643900	-0.27051000
C	0.22114600	3.59827600	-1.42802600
C	-0.66467000	2.65437800	-1.91002000
H	-2.24213700	0.29528100	-2.02165300
Br	-2.43415000	-2.93066100	-1.17077800
Br	0.54061200	-4.69063300	-2.49193600
Br	3.09135500	-2.00334900	-2.84622200
Br	-1.73435900	2.77887600	-3.46303600
Br	0.63573900	5.29043000	-2.15208000
Br	2.03576600	3.68685900	0.92727700

Br	-4.80725800	0.62039100	-0.76679600
Br	-5.33435100	-0.37870600	2.74188900
Br	-1.88797200	-1.12590800	3.99095000
C	2.90997100	-0.43376800	1.58013000
C	3.56098200	-1.71293800	1.26627300
C	2.78576400	-2.73147700	0.68114200
C	4.90276500	-2.00030300	1.57854800
C	3.33297700	-3.96709400	0.36788900
H	1.73451500	-2.53801000	0.47376800
C	5.44851600	-3.24396300	1.28376100
H	5.51163100	-1.25590900	2.07903900
C	4.67048200	-4.22297900	0.66814200
H	2.71773100	-4.72977300	-0.09874600
H	6.48220200	-3.45321400	1.53954900
H	5.10149800	-5.19244900	0.43794400
C	3.69214600	0.61188100	2.27479100
C	4.90304400	1.13240500	1.78476200
C	3.17100200	1.13541300	3.46620100
C	5.56313100	2.14692300	2.46776200
H	5.29577700	0.75972300	0.84299900
C	3.85748000	2.11737400	4.17409700
H	2.21549200	0.76517400	3.83054200
C	5.04864100	2.63391900	3.66989600
H	6.48346900	2.55726500	2.06432100
H	3.44518100	2.50091500	5.10175300
H	5.57009600	3.42117400	4.20508600
O	3.09550800	0.43484600	-0.28266200
H	2.95990000	1.37537800	-0.06521700
H	2.30359300	0.18120800	-0.81446900

Int2-O

Zero-point correction= 0.317401 (Hartree/Particle)
Thermal correction to Energy= 0.358703
Thermal correction to Enthalpy= 0.359648
Thermal correction to Gibbs Free Energy= 0.232520
Sum of electronic and zero-point Energies= -1541.013649
Sum of electronic and thermal Energies= -1540.972347
Sum of electronic and thermal Enthalpies= -1540.971403
Sum of electronic and thermal Free Energies= -1541.098531

Ag	0.78767300	-0.08934000	1.58674000
N	1.14797000	-0.49961600	-1.64511800
N	-0.06654200	-0.98384900	-1.26632400

N	-1.38158800	-0.61212300	1.37930300
N	-2.06202600	-0.43899800	0.22311000
N	-0.05717000	1.85514200	-0.19611800
N	-0.93201500	1.39986000	-1.11973300
B	-1.34363500	-0.08135200	-1.11760200
C	1.85670000	-1.54181500	-2.05279700
C	1.13683700	-2.73333300	-1.94310000
C	-0.08314300	-2.31979000	-1.44580900
C	-2.26126200	-0.95022100	2.30365400
C	-3.55288000	-1.00717300	1.77527700
C	-3.37031400	-0.67138400	0.44644900
C	0.01850400	3.15773000	-0.37046000
C	-0.80441300	3.59819900	-1.41256500
C	-1.39480500	2.43155400	-1.85835500
H	-2.09342300	-0.30006100	-2.01635200
Br	-1.60356300	-3.39122000	-1.11039900
Br	1.68938800	-4.48244900	-2.37545800
Br	3.61971500	-1.33017300	-2.70446000
Br	-2.64711000	2.25973000	-3.26305400
Br	-1.04797400	5.35466000	-2.05513400
Br	1.20745800	4.17911600	0.70744100
Br	-4.70624200	-0.55112400	-0.88229400
Br	-5.16017700	-1.44317600	2.65969200
Br	-1.70121000	-1.27688000	4.08082600
C	2.92176100	0.33526000	1.18510700
C	3.80228000	-0.85351800	0.87300100
C	3.26787100	-2.14488200	0.82728900
C	5.17048800	-0.69249300	0.61374600
C	4.06824300	-3.24631600	0.52806600
H	2.20748100	-2.28867700	1.03027300
C	5.97241200	-1.78557100	0.30781400
H	5.60779200	0.30098700	0.65996200
C	5.42232100	-3.06668200	0.26473100
H	3.63179500	-4.23987000	0.50208500
H	7.03009300	-1.64096200	0.11002400
H	6.04962100	-3.92141700	0.03149600
C	3.58301100	1.34083900	2.08335400
C	4.32654200	2.43737500	1.62756000
C	3.46587700	1.15207100	3.46764400
C	4.91056600	3.32793700	2.52877500
H	4.46317200	2.60233500	0.56212900
C	4.06993000	2.02358800	4.36406600
H	2.88101100	0.31079700	3.83365800
C	4.78783700	3.12552700	3.89851700

H	5.47360100	4.17616100	2.15099700
H	3.96372800	1.85444400	5.43110700
H	5.24415200	3.81790900	4.59837600
O	2.81725600	1.08999400	-0.22079600
H	2.37631100	1.94604000	-0.02397900
H	2.13757400	0.57686800	-0.79040800

Int3-O

Zero-point correction=	0.368592 (Hartree/Particle)
Thermal correction to Energy=	0.411116
Thermal correction to Enthalpy=	0.412060
Thermal correction to Gibbs Free Energy=	0.288950
Sum of electronic and zero-point Energies=	-1693.841518
Sum of electronic and thermal Energies=	-1693.798995
Sum of electronic and thermal Enthalpies=	-1693.798051
Sum of electronic and thermal Free Energies=	-1693.921161

Ag	0.25805100	1.24929400	-0.40924600
N	-1.75528700	0.39250500	-0.70108300
N	-1.99476400	-0.93265000	-0.46224600
N	0.62686100	-1.16791000	-1.43549100
N	0.25561000	-2.04759200	-0.45327500
N	0.68955600	-0.72182400	1.95371100
N	-0.61909300	-0.95939400	1.64792400
B	-1.00932300	-1.78458800	0.37801200
C	-2.72896900	0.81967000	-1.49715400
C	-3.63335600	-0.20717000	-1.79274800
C	-3.11899700	-1.30473000	-1.11216100
C	1.77191500	-1.61785500	-1.92213200
C	2.18652600	-2.79454700	-1.27233800
C	1.18552200	-3.02373300	-0.33883600
C	0.68207700	-0.09191000	3.11743600
C	-0.62439600	0.11480500	3.60226700
C	-1.42069700	-0.47339600	2.62272900
H	-1.53945200	-2.80132000	0.69979400
Br	-3.84198300	-3.05717700	-1.04919000
Br	-5.20355800	-0.12010100	-2.85928500
Br	-2.75979300	2.61667100	-2.13722100
Br	-3.31499100	-0.62866500	2.58388900
Br	-1.17995100	0.90840300	5.24252900
Br	2.32882100	0.45163500	3.93708800
Br	1.06745300	-4.42025100	0.94607200
Br	3.74416700	-3.83955200	-1.59568400

Br	2.65026500	-0.68366000	-3.33333100
C	2.06827800	2.39047200	-0.14338600
C	1.91182300	3.66046800	-0.91457500
C	1.90214800	4.93864400	-0.33341300
C	1.68524300	3.56297300	-2.30319700
C	1.63639600	6.07608500	-1.10905700
H	2.10905800	5.05184600	0.72488000
C	1.44051200	4.69513700	-3.07119300
H	1.68568300	2.58052600	-2.76861500
C	1.39926500	5.96294500	-2.47693600
H	1.62494700	7.05218900	-0.63177700
H	1.26767800	4.58813800	-4.13840400
H	1.19081000	6.84457300	-3.07498600
C	3.32029800	1.59774300	-0.40765300
C	4.39390400	2.11836000	-1.14584900
C	3.44546300	0.30910000	0.14239200
C	5.55148100	1.36555600	-1.34164000
H	4.31825100	3.11111200	-1.57631200
C	4.61435800	-0.42939200	-0.02852300
H	2.60778200	-0.13029100	0.67572700
C	5.66857800	0.09295100	-0.77961800
H	6.36839300	1.77969100	-1.92610700
H	4.68577000	-1.42521900	0.39730900
H	6.57107400	-0.49147700	-0.93308500
H	1.23906100	2.90645300	1.81859200
O	-1.13878800	4.46629400	0.37871000
H	-0.33337400	4.80182200	-0.06327600
H	-1.52649800	3.84762000	-0.26471700
O	-0.02998300	3.10176900	2.36617900
H	-0.47033200	2.24454200	2.50977900
H	-0.55738900	3.59671000	1.66705000
O	2.21626000	2.81812000	1.36459900
H	2.68736200	2.08914000	1.81915100

Int4-O

Zero-point correction= 0.256301 (Hartree/Particle)
Thermal correction to Energy= 0.271414
Thermal correction to Enthalpy= 0.272359
Thermal correction to Gibbs Free Energy= 0.214360
Sum of electronic and zero-point Energies= -730.082101
Sum of electronic and thermal Energies= -730.066987
Sum of electronic and thermal Enthalpies= -730.066043
Sum of electronic and thermal Free Energies= -730.125342

C	0.13744900	0.51830700	-0.06612800
C	-1.03791500	-0.34582900	-0.12919500
C	-2.07448200	-0.21167100	-1.08213000
C	-1.23726800	-1.31493200	0.88732700
C	-3.23018200	-0.99635400	-1.02434900
H	-1.97625700	0.48863600	-1.90862700
C	-2.38179900	-2.09739800	0.93237600
H	-0.47693300	-1.42963800	1.65439000
C	-3.39765600	-1.94501700	-0.02070100
H	-3.99794000	-0.86346900	-1.78276600
H	-2.49257200	-2.82885700	1.72895600
H	-4.29677600	-2.55139300	0.02596700
C	1.51652100	0.00395600	-0.08015200
C	1.82071100	-1.37102300	-0.04834800
C	2.60082300	0.90777400	-0.11472500
C	3.14115600	-1.81689400	-0.02371200
H	1.01496700	-2.09711700	-0.05463700
C	3.91632500	0.45785600	-0.11622100
H	2.39508900	1.97360200	-0.13581100
C	4.19971500	-0.90969800	-0.06116700
H	3.34095900	-2.88526100	0.00363100
H	4.72789400	1.18057300	-0.14585300
H	5.22788900	-1.25969700	-0.04814200
H	-0.85943000	2.23751900	-0.83418300
O	-0.72978700	2.21628900	1.95100000
H	-0.24658400	1.52266200	1.34660600
H	-1.26333300	1.67170200	2.55130900
O	-1.91094600	2.82459500	-0.24769700
H	-2.61123300	2.14959300	-0.31836500
H	-1.58048900	2.74198100	0.72367600
O	0.04409300	1.63107300	-1.09178600
H	0.02747600	1.29903900	-2.01552000

TS2-O

Zero-point correction= 0.362435 (Hartree/Particle)
Thermal correction to Energy= 0.408403
Thermal correction to Enthalpy= 0.409347
Thermal correction to Gibbs Free Energy= 0.274349
Sum of electronic and zero-point Energies= -1693.822239
Sum of electronic and thermal Energies= -1693.776271
Sum of electronic and thermal Enthalpies= -1693.775327
Sum of electronic and thermal Free Energies= -1693.910325

Ag	0.88207500	1.08193500	-0.03274700
N	-1.46028100	1.27521000	-0.33375900
N	-2.10022100	0.15964700	-0.80181900
N	0.49371300	-0.47196500	-1.73977500
N	-0.28925100	-1.51338800	-1.31561600
N	0.07761200	-1.59906700	1.44246900
N	-1.19248700	-1.31388500	1.02997400
B	-1.55903700	-1.25340100	-0.48084200
C	-2.12800700	2.31585200	-0.81522800
C	-3.21837900	1.91825400	-1.59858100
C	-3.15463200	0.53011800	-1.56209900
C	1.48510100	-1.00607900	-2.44537900
C	1.38819500	-2.40357900	-2.48282200
C	0.24110000	-2.67696700	-1.74570500
C	0.01249600	-1.72169400	2.76094900
C	-1.29283900	-1.50358500	3.24747400
C	-2.02606500	-1.25273700	2.09184100
H	-2.39581600	-2.06235300	-0.73085500
Br	-4.33318400	-0.69608900	-2.40422000
Br	-4.49644300	3.01105500	-2.48457900
Br	-1.56347200	4.10606600	-0.44096900
Br	-3.88440700	-0.88920900	1.92373100
Br	-1.93265900	-1.60345000	5.03794400
Br	1.56894000	-2.14993300	3.78791600
Br	-0.51641700	-4.36938800	-1.33772900
Br	2.55461300	-3.64137600	-3.33195200
Br	2.77717400	0.11395300	-3.27129800
C	2.78106000	1.69167000	1.20688000
C	3.39086800	3.07103700	1.07605200
C	4.15551500	3.64298500	2.09526300
C	3.18958700	3.79660500	-0.10678400
C	4.71914500	4.91017200	1.93055700
H	4.29352000	3.09840600	3.02293800
C	3.76216800	5.05432000	-0.27903500
H	2.57933100	3.36199200	-0.89785500
C	4.52999100	5.61852800	0.74419800
H	5.30684500	5.34416900	2.73495800
H	3.60053900	5.59873500	-1.20535300
H	4.96701300	6.60492500	0.61900500
C	3.60544600	0.63329200	0.46781600
C	4.77192300	0.95229500	-0.24559200
C	3.25374100	-0.72697400	0.58066100
C	5.54491200	-0.04967700	-0.83547400

H	5.07983500	1.98587300	-0.34356000
C	4.02082600	-1.72324100	-0.01765000
H	2.35354800	-1.00796300	1.11303800
C	5.17457300	-1.38954800	-0.73021800
H	6.44327100	0.22530800	-1.38132200
H	3.70687700	-2.75949900	0.06768200
H	5.77223600	-2.16452600	-1.20068800
H	1.15476000	1.07374100	3.50484700
O	0.65831900	3.19461400	2.04432800
H	1.35917600	2.44592100	1.38204500
H	-0.07789500	3.47490500	1.46373700
O	0.23658600	1.36236800	3.73144100
H	-0.34361100	0.62918500	3.46220800
H	0.29345100	2.54826700	2.76414300
O	2.70044800	1.27416600	2.60373900
H	3.44272500	0.67063700	2.77961500

TS2-O'

Zero-point correction= 0.255753 (Hartree/Particle)
Thermal correction to Energy= 0.270718
Thermal correction to Enthalpy= 0.271662
Thermal correction to Gibbs Free Energy= 0.213975
Sum of electronic and zero-point Energies= -730.081925
Sum of electronic and thermal Energies= -730.066960
Sum of electronic and thermal Enthalpies= -730.066016
Sum of electronic and thermal Free Energies= -730.122203

C	0.13598400	0.52320700	-0.06975800
C	-1.04104500	-0.34197100	-0.13109600
C	-2.07719800	-0.20446100	-1.08378500
C	-1.24397200	-1.30877300	0.88643500
C	-3.23651100	-0.98393400	-1.02478000
H	-1.97350200	0.49340000	-1.91168000
C	-2.39187300	-2.08647800	0.93296800
H	-0.48379300	-1.42591100	1.65334700
C	-3.40749500	-1.93058200	-0.01974900
H	-4.00391600	-0.84910500	-1.78323500
H	-2.50556400	-2.81665400	1.73032500
H	-4.30912500	-2.53318200	0.02797500
C	1.51383700	0.00164900	-0.08243600
C	1.81344100	-1.37401000	-0.04802100
C	2.60087800	0.90176100	-0.11943100
C	3.13248200	-1.82431800	-0.02330000

H	1.00546300	-2.09765000	-0.05304400
C	3.91503000	0.44764200	-0.11943700
H	2.39817600	1.96805500	-0.14408600
C	4.19400300	-0.92068900	-0.06202400
H	3.32875500	-2.89331700	0.00554200
H	4.72895500	1.16767300	-0.15059500
H	5.22103800	-1.27406800	-0.04862900
H	-0.89281400	2.25948400	-0.80842300
O	-0.70171800	2.20770000	1.93267700
H	-0.22642200	1.50007300	1.31630900
H	-1.22821500	1.67106800	2.54646300
O	-1.89090600	2.81441500	-0.22733700
H	-2.59490700	2.14263400	-0.30187500
H	-1.54590800	2.71847000	0.75089800
O	0.04208400	1.63642900	-1.08465300
H	0.04073600	1.30641200	-2.00831200

33'

Zero-point correction= 0.211836 (Hartree/Particle)
Thermal correction to Energy= 0.223135
Thermal correction to Enthalpy= 0.224079
Thermal correction to Gibbs Free Energy= 0.174247
Sum of electronic and zero-point Energies= -577.342167
Sum of electronic and thermal Energies= -577.330868
Sum of electronic and thermal Enthalpies= -577.329924
Sum of electronic and thermal Free Energies= -577.379756

C	-0.03221400	1.17384400	-0.11859500
C	1.29065600	0.45413400	-0.08686900
C	1.67610000	-0.27149400	1.04745700
C	2.15842300	0.53195200	-1.17961300
C	2.90027700	-0.93640600	1.07358400
H	1.01468700	-0.30379700	1.90766000
C	3.38525700	-0.13488300	-1.15384200
H	1.88006900	1.12922900	-2.04222800
C	3.75486700	-0.87512100	-0.03064300
H	3.19079900	-1.49910400	1.95623800
H	4.05419700	-0.06542100	-2.00693800
H	4.70946500	-1.39314600	-0.00894300
C	-1.28724600	0.31427600	-0.10605400
C	-1.26867000	-1.08081500	-0.03520800
C	-2.52453400	0.96834800	-0.21120600
C	-2.46369800	-1.80341100	-0.05038200

H	-0.32124500	-1.60475100	0.01643700
C	-3.71756000	0.24940600	-0.22194700
H	-2.56080300	2.04731200	-0.32671000
C	-3.68944300	-1.14378000	-0.13788900
H	-2.43318400	-2.88797000	-0.00185600
H	-4.66355100	0.77494700	-0.31519600
H	-4.61506300	-1.71088900	-0.15957700
H	-0.06848200	1.77984800	-1.03820000
O	-0.03962900	2.09053300	1.01168500
H	-0.96314200	2.33319000	1.19307700

Int1-N[a]

Zero-point correction=	0.352265 (Hartree/Particle)
Thermal correction to Energy=	0.398662
Thermal correction to Enthalpy=	0.399606
Thermal correction to Gibbs Free Energy=	0.261994
Sum of electronic and zero-point Energies=	-1597.549284
Sum of electronic and thermal Energies=	-1597.502888
Sum of electronic and thermal Enthalpies=	-1597.501943
Sum of electronic and thermal Free Energies=	-1597.639555

Ag	0.26842700	-0.31137400	1.93400300
N	1.37719800	-1.37777300	0.07073700
N	1.25305500	-1.05076000	-1.24671400
N	-1.48053800	-0.57888600	-1.28731000
N	-0.69856400	0.45256900	-1.72749400
N	0.53332600	2.02152700	0.23689100
N	1.37260800	1.39192000	-0.63467200
B	0.83994400	0.36954300	-1.68128900
C	1.68879700	-2.66585100	0.10979700
C	1.77141900	-3.21705700	-1.17624500
C	1.48023700	-2.14309600	-2.00790400
C	-2.72624700	-0.21000400	-1.53421000
C	-2.79879000	1.06257400	-2.13008400
C	-1.47007300	1.44223500	-2.23495100
C	1.28521300	2.87023900	0.92144900
C	2.63493700	2.80949600	0.53310000
C	2.63411300	1.84621600	-0.47231800
H	1.31425100	0.62224000	-2.74807500
Br	1.37414100	-2.13137500	-3.90432600
Br	2.19603900	-5.00398900	-1.67314200
Br	1.97572400	-3.54289700	1.78426500
Br	4.10441000	1.17516800	-1.47133600

Br	4.11586700	3.80095500	1.20678400
Br	0.49905900	3.99272900	2.26064000
Br	-0.75012800	3.05899500	-2.93579000
Br	-4.34133700	2.04386100	-2.66507100
Br	-4.20333000	-1.34505300	-1.09421500
C	-1.73867000	-0.67998500	2.37483900
C	-2.06014800	-2.01932400	2.82188500
C	-1.18186400	-2.68984500	3.70814400
C	-3.19082700	-2.72685000	2.34156100
C	-1.45644900	-3.97668800	4.14845200
H	-0.29525300	-2.16768900	4.05383600
C	-3.42643700	-4.03880900	2.73700000
H	-3.83265600	-2.25612200	1.60781000
C	-2.57379600	-4.65969100	3.65354000
H	-0.78881200	-4.46289400	4.85321400
H	-4.27577600	-4.57984800	2.33075500
H	-2.76744000	-5.68075000	3.96913400
C	-2.80144200	0.25315300	2.12253700
C	-4.08974400	0.13269000	2.71400300
C	-2.55069100	1.37386400	1.28819200
C	-5.07980300	1.06735100	2.44928400
H	-4.28217100	-0.67552400	3.41012500
C	-3.55687300	2.28325400	0.99901100
H	-1.56580300	1.49526400	0.85218500
C	-4.82170800	2.13190500	1.57655300
H	-6.05365600	0.97497700	2.92041100
H	-3.35727500	3.11239000	0.32866900
H	-5.60349100	2.85464600	1.36113900
N	2.32391000	0.23060600	2.71680000
H	2.15810700	0.69975900	3.61106700
H	2.82275400	0.86389500	2.09441900
H	2.90686700	-0.59011300	2.86746000
O	0.49397700	1.35432000	4.76269700
H	-0.05803000	0.74197000	4.24309600
H	0.35125000	2.20723800	4.31863900

TS1-N[a]

Zero-point correction= 0.349876 (Hartree/Particle)
Thermal correction to Energy= 0.395744
Thermal correction to Enthalpy= 0.396688
Thermal correction to Gibbs Free Energy= 0.261419
Sum of electronic and zero-point Energies= -1597.535497
Sum of electronic and thermal Energies= -1597.489629

Sum of electronic and thermal Enthalpies= -1597.488685
Sum of electronic and thermal Free Energies= -1597.623954

Ag	-0.71018800	0.95725600	-0.91148600
N	1.38042700	1.41126100	-0.37117000
N	1.98245000	0.86870300	0.73202000
N	-0.58087300	0.73589600	1.87073400
N	0.14080000	-0.42467300	1.89730500
N	0.34726800	-1.61289600	-0.67779300
N	1.48420800	-1.50053800	0.07314500
B	1.55205100	-0.52086500	1.26699400
C	2.01031200	2.55159100	-0.63510900
C	3.04335400	2.78149800	0.28159500
C	2.98387500	1.68266300	1.13160800
C	-1.73711100	0.45752800	2.45669100
C	-1.80694200	-0.88256100	2.87367900
C	-0.57695900	-1.40409000	2.49220500
C	0.64481300	-2.44872000	-1.66258300
C	1.97344400	-2.89929700	-1.58865400
C	2.46859400	-2.25800200	-0.46182300
H	2.35023700	-0.90548300	2.06065400
Br	4.10439200	1.32453500	2.62219800
Br	4.25404500	4.24583700	0.34458300
Br	1.46071900	3.64345800	-2.09410900
Br	4.22722600	-2.34192200	0.25761500
Br	2.88315700	-4.11144300	-2.73929900
Br	-0.65690900	-2.91029700	-2.98467000
Br	0.07102000	-3.18086400	2.69954600
Br	-3.23734400	-1.78429600	3.74949700
Br	-3.09283100	1.79442400	2.60923500
C	-2.78128300	1.05549100	-1.18610000
C	-3.35904400	2.35460900	-1.42770000
C	-2.72952200	3.22358500	-2.35451200
C	-4.48238900	2.83456800	-0.70697800
C	-3.23491600	4.49428900	-2.58411100
H	-1.89099300	2.83486400	-2.92060100
C	-4.94385000	4.13205300	-0.89984900
H	-4.93173600	2.20718500	0.05394300
C	-4.33259000	4.95769200	-1.84720800
H	-2.76522900	5.13805800	-3.32161400
H	-5.77851000	4.50208000	-0.31194700
H	-4.70254900	5.96693700	-2.00385500
C	-3.63395000	-0.06297000	-0.87551300
C	-5.01404200	-0.10361100	-1.21295100

C	-3.06288800	-1.20877200	-0.26355400
C	-5.78557100	-1.21865500	-0.90959100
H	-5.45341800	0.73005600	-1.74833400
C	-3.84534400	-2.30923700	0.06476200
H	-1.99812400	-1.21950200	-0.06100700
C	-5.20674600	-2.31521700	-0.25511300
H	-6.83705200	-1.24220400	-1.18046900
H	-3.39341700	-3.16538300	0.55438500
H	-5.81439000	-3.18345100	-0.01739400
N	-1.90160400	0.53179500	-4.26284100
H	-2.58140900	-0.22152000	-4.12539600
H	-1.01081900	0.15217100	-3.94414200
H	-1.80818100	0.65286100	-5.27158100
O	-3.73932300	-1.90447500	-3.60674700
H	-3.22510600	-2.07278400	-2.80103100
H	-4.61397000	-1.65834900	-3.26859800

Int4-N[a]

Zero-point correction= 0.274754 (Hartree/Particle)
Thermal correction to Energy= 0.290815
Thermal correction to Enthalpy= 0.291760
Thermal correction to Gibbs Free Energy= 0.231345
Sum of electronic and zero-point Energies= -710.223820
Sum of electronic and thermal Energies= -710.207759
Sum of electronic and thermal Enthalpies= -710.206815
Sum of electronic and thermal Free Energies= -710.267230

C	0.10320400	0.48455800	-0.18615400
C	-1.06680500	-0.38090300	-0.13523000
C	-2.19546300	-0.22228400	-0.97523500
C	-1.16067500	-1.38903900	0.85842100
C	-3.33181700	-1.02412100	-0.83666800
H	-2.20386000	0.52464200	-1.76520500
C	-2.28755200	-2.18626700	0.98384700
H	-0.33375600	-1.51804200	1.54963900
C	-3.39084400	-2.01540600	0.13690300
H	-4.17313300	-0.86787500	-1.50774400
H	-2.31502200	-2.94471700	1.76227100
H	-4.27460200	-2.63661000	0.24506800
C	1.48672200	-0.02423900	-0.12572300
C	1.81919000	-1.39187500	-0.20872700
C	2.56218300	0.88389700	0.00824400
C	3.14363300	-1.82014500	-0.15661500

H	1.02547000	-2.12130500	-0.32863500
C	3.88703200	0.45873500	0.02996000
H	2.35043700	1.94596000	0.14107500
C	4.18994100	-0.90224800	-0.04632100
H	3.36091600	-2.88325800	-0.22501400
H	4.68432500	1.19041400	0.13360500
H	5.22126400	-1.24054300	-0.01174000
N	-0.03111400	1.51798900	-1.27649000
H	0.83182300	2.06039800	-1.33216200
H	-0.82926800	2.17601100	-1.03948000
H	-0.19063600	1.11509500	-2.21690100
O	-0.60000200	2.25907100	1.89975000
H	-0.24524800	1.52856800	1.28345400
H	0.18278900	2.75873900	2.17682800
O	-2.03672800	3.01347600	-0.21514500
H	-2.74643400	2.35146800	-0.16048800
H	-1.58592200	2.93077200	0.67382200

TS2-N[a]

Zero-point correction=	0.269874 (Hartree/Particle)
Thermal correction to Energy=	0.285122
Thermal correction to Enthalpy=	0.286066
Thermal correction to Gibbs Free Energy=	0.227427
Sum of electronic and zero-point Energies=	-710.220073
Sum of electronic and thermal Energies=	-710.204825
Sum of electronic and thermal Enthalpies=	-710.203881
Sum of electronic and thermal Free Energies=	-710.262520

C	0.05278200	0.54567000	-0.07582600
C	-1.10746200	-0.37354500	-0.03812700
C	-2.23834400	-0.23818400	-0.86442700
C	-1.14051100	-1.39200600	0.93805900
C	-3.33925700	-1.08902700	-0.73434900
H	-2.28858900	0.54226500	-1.61888500
C	-2.23365300	-2.23903500	1.06248200
H	-0.29239700	-1.50428800	1.60640600
C	-3.34489500	-2.09758800	0.22395800
H	-4.19465500	-0.95553700	-1.39165400
H	-2.22376000	-3.01328800	1.82511800
H	-4.20035200	-2.75871900	0.32536200
C	1.43286700	-0.04533400	-0.06509500
C	1.71846300	-1.35916100	-0.46892900
C	2.50873700	0.77347400	0.32082600

C	3.02930300	-1.83492400	-0.48364500
H	0.90307300	-2.01132700	-0.76591500
C	3.82158600	0.30726300	0.28998900
H	2.29987400	1.78214400	0.67232000
C	4.08674700	-1.00348300	-0.11058400
H	3.22654400	-2.85672400	-0.79713300
H	4.63482800	0.95925200	0.59721900
H	5.10722500	-1.37554400	-0.12470900
N	-0.05157900	1.47456200	-1.27809000
H	0.82586700	1.99006300	-1.36670500
H	-0.83466000	2.19852700	-1.09157500
H	-0.20620000	0.97840000	-2.16631700
O	-0.48928700	2.43973500	1.64397700
H	-0.13254000	1.47662100	0.94538900
H	0.29765300	2.95260100	1.88596500
O	-1.77315500	3.25891600	-0.37571000
H	-2.65155700	2.87098800	-0.23718000
H	-1.28794300	3.08871200	0.53679500

Int4-N[b]

Zero-point correction=	0.249516 (Hartree/Particle)
Thermal correction to Energy=	0.263649
Thermal correction to Enthalpy=	0.264593
Thermal correction to Gibbs Free Energy=	0.208381
Sum of electronic and zero-point Energies=	-633.807718
Sum of electronic and thermal Energies=	-633.793585
Sum of electronic and thermal Enthalpies=	-633.792641
Sum of electronic and thermal Free Energies=	-633.848853

C	0.07501900	0.64539600	-0.10350900
C	-1.16634200	-0.07979100	-0.10619600
C	-2.33375600	0.38885500	-0.77256000
C	-1.32602900	-1.27437400	0.65332800
C	-3.54782400	-0.29757100	-0.70226500
H	-2.31137800	1.30079000	-1.36512300
C	-2.53541300	-1.94655900	0.71144200
H	-0.48283700	-1.64748400	1.22423700
C	-3.66687500	-1.47361200	0.03119200
H	-4.40759100	0.10087300	-1.23626200
H	-2.60401000	-2.85278400	1.30865700
H	-4.61199300	-2.00459500	0.08528600
C	1.42421000	0.09380200	-0.04141600
C	1.71283800	-1.27391300	-0.25581700

C	2.53298200	0.93894000	0.21388200
C	3.01739700	-1.75533800	-0.21538400
H	0.90127700	-1.95489100	-0.48632400
C	3.83949000	0.45930700	0.22159100
H	2.36239000	1.98550000	0.46908200
C	4.09597700	-0.89677300	0.01363700
H	3.19623700	-2.81334500	-0.39167000
H	4.65883100	1.14517300	0.42240200
H	5.11243700	-1.27787800	0.03807400
N	0.01235100	1.89403500	-0.93249100
H	0.94999800	2.29637700	-0.99769300
H	-0.61599100	2.57752900	-0.45470300
H	-0.32701000	1.74063200	-1.89997800
H	-0.86113600	2.04113300	1.38334300
O	-1.43431600	2.81741900	1.18486100
H	-2.30120400	2.39515900	1.06166800

TS2-N[b]

Zero-point correction= 0.244608 (Hartree/Particle)
Thermal correction to Energy= 0.257771
Thermal correction to Enthalpy= 0.258715
Thermal correction to Gibbs Free Energy= 0.204950
Sum of electronic and zero-point Energies= -633.794631
Sum of electronic and thermal Energies= -633.781468
Sum of electronic and thermal Enthalpies= -633.780524
Sum of electronic and thermal Free Energies= -633.834289

C	-0.00010000	0.77205500	-0.03707200
C	-1.27562100	0.03207700	-0.00731600
C	-2.45450700	0.52903600	-0.60189500
C	-1.39092600	-1.16415000	0.73467800
C	-3.66849200	-0.15089200	-0.49457200
H	-2.45228400	1.48044700	-1.12876800
C	-2.60270500	-1.83578400	0.84296800
H	-0.51289700	-1.56185100	1.23264200
C	-3.75372600	-1.34222100	0.22127600
H	-4.55321500	0.26430500	-0.97056800
H	-2.64905200	-2.75672600	1.41859500
H	-4.69818200	-1.87142300	0.30497400
C	1.30093700	0.02714000	-0.01888600
C	1.47617100	-1.24077600	-0.59846900
C	2.42183600	0.66061800	0.54475200
C	2.72619800	-1.85787500	-0.60439900

H	0.62209100	-1.74487700	-1.04105700
C	3.67650700	0.05140800	0.52532800
H	2.28846300	1.63464500	1.00937500
C	3.83158200	-1.21246300	-0.04566800
H	2.84060600	-2.84059100	-1.05449500
H	4.52941800	0.55738400	0.96962200
H	4.80558900	-1.69390400	-0.05219300
N	0.02517900	1.78023900	-1.20175300
H	0.98155700	1.88117800	-1.54627900
H	-0.24150500	2.68830700	-0.65861000
H	-0.58864800	1.54294600	-1.98795500
H	-0.06856800	1.89824600	0.82571400
O	-0.15000400	3.14180800	0.87764700
H	-1.00251700	3.31673200	1.30575800

Int3-O[a]

Zero-point correction=	0.393171 (Hartree/Particle)
Thermal correction to Energy=	0.440275
Thermal correction to Enthalpy=	0.441219
Thermal correction to Gibbs Free Energy=	0.304778
Sum of electronic and zero-point Energies=	-1770.267926
Sum of electronic and thermal Energies=	-1770.220822
Sum of electronic and thermal Enthalpies=	-1770.219878
Sum of electronic and thermal Free Energies=	-1770.356319

Ag	1.35361500	0.65382500	-0.73616600
N	0.25863800	-1.96490100	0.69014600
N	-0.14880200	-0.91618200	1.46572800
N	-0.15218300	1.75551200	0.36842800
N	-1.26133800	1.30435600	1.02024200
N	-2.43118600	-0.08485000	-1.10246000
N	-2.27199800	-0.79238600	0.06318200
B	-1.51894300	-0.19647400	1.26580500
C	1.36373400	-2.44428100	1.25598900
C	1.70866900	-1.71946200	2.40568100
C	0.70838200	-0.75939300	2.49935200
C	-0.23136100	3.08635600	0.33164800
C	-1.40359500	3.53007000	0.95505600
C	-2.02569900	2.35927300	1.37304600
C	-3.31752700	-0.76667500	-1.81633400
C	-3.75179200	-1.92993200	-1.15390900
C	-3.06075000	-1.89625300	0.04503900
H	-2.16236900	-0.32333500	2.26286800

Br	0.49028400	0.59025800	3.81370600
Br	3.17311000	-1.98913800	3.58500900
Br	2.26733000	-3.96150800	0.53391700
Br	-3.11222300	-3.14338300	1.47845500
Br	-4.98787200	-3.24473000	-1.75207700
Br	-3.88233500	-0.14370100	-3.53126600
Br	-3.68793700	2.18369000	2.26921600
Br	-2.00254300	5.31805200	1.18685800
Br	1.13966100	4.10750100	-0.49810600
C	2.94370700	0.16838300	-2.09630200
C	3.98140100	-0.77245000	-1.53465800
C	4.71167300	-1.64493000	-2.35769200
C	4.27043000	-0.77842900	-0.16040900
C	5.67790700	-2.50220400	-1.82634400
H	4.52554700	-1.66212900	-3.42759300
C	5.25760500	-1.60662200	0.36918100
H	3.69726800	-0.13188200	0.50030700
C	5.96051200	-2.48288400	-0.46033900
H	6.21842200	-3.17501000	-2.48668300
H	5.45474600	-1.58793600	1.43616200
H	6.71448100	-3.14532300	-0.04566300
C	3.46764200	1.54151600	-2.43649400
C	4.56152400	2.11092800	-1.75883200
C	2.82950400	2.32314000	-3.42005200
C	4.97873800	3.41075800	-2.03539800
H	5.08351200	1.53264500	-1.00473900
C	3.25863100	3.61882200	-3.70496300
H	1.96108600	1.93075600	-3.94046700
C	4.33254700	4.17619500	-3.00995000
H	5.82438300	3.82424500	-1.49248900
H	2.74191000	4.19606500	-4.46688700
H	4.66491000	5.18691100	-3.22709700
H	0.54770400	-0.16439400	-3.27914300
O	-0.36809200	-2.55878300	-1.85798200
H	2.15111200	-2.14555100	-3.11040300
H	-0.25023100	-2.32245200	-0.87116700
O	-0.40975300	-0.38840400	-3.20176700
H	-0.84032800	0.27285200	-2.62662900
H	-0.49961800	-1.67150200	-2.38696800
O	1.92633300	-3.06208700	-2.80783100
H	2.58569300	-3.24080500	-2.11502100
H	0.56012200	-2.89622900	-2.20199000
O	2.34226900	-0.41746600	-3.31637600
H	2.87556600	-0.14928100	-4.09067600

TS2-O[a]

Zero-point correction= 0.387781 (Hartree/Particle)
Thermal correction to Energy= 0.435754
Thermal correction to Enthalpy= 0.436698
Thermal correction to Gibbs Free Energy= 0.298616
Sum of electronic and zero-point Energies= -1770.238681
Sum of electronic and thermal Energies= -1770.190709
Sum of electronic and thermal Enthalpies= -1770.189764
Sum of electronic and thermal Free Energies= -1770.327847

Ag	-0.88024700	0.48761400	-0.95362500
N	1.29554000	1.41503500	-0.47685600
N	1.73394800	1.08674300	0.78223300
N	-0.92289200	0.63694000	1.38409300
N	-0.03981600	-0.27766300	1.89855300
N	0.63365900	-2.33912600	0.14611500
N	1.59622700	-1.38550500	0.31385600
B	1.42655300	-0.29716600	1.40275400
C	1.58803500	2.70573600	-0.64228800
C	2.21887600	3.24331900	0.48376600
C	2.28193300	2.17052900	1.36727900
C	-2.04391500	0.51761100	2.09070300
C	-1.92948600	-0.48503200	3.06228100
C	-0.63271700	-0.96152200	2.89751100
C	1.16650300	-3.24523700	-0.65624400
C	2.47899400	-2.91649700	-1.04254400
C	2.71281100	-1.71980100	-0.38155400
H	2.16831300	-0.49574400	2.31473300
Br	3.00310900	2.16988900	3.12144500
Br	2.85772500	5.01311200	0.74750200
Br	1.16292900	3.64174200	-2.25182500
Br	4.30400100	-0.67542100	-0.36189900
Br	3.64951900	-3.85868100	-2.20735700
Br	0.16726900	-4.80089800	-1.17676500
Br	0.24381500	-2.34232600	3.85721400
Br	-3.22882800	-1.05552000	4.32642000
Br	-3.49238700	1.71558700	1.79043700
C	-2.54338600	-0.14211900	-2.55482400
C	-3.40609800	0.99178800	-3.05696900
C	-4.09068200	0.92363100	-4.27442200
C	-3.50066300	2.17290000	-2.30248400
C	-4.86509400	1.99890500	-4.71663300

H	-3.99672100	0.03371100	-4.88674000
C	-4.28212100	3.24146000	-2.73475700
H	-2.95935300	2.24810400	-1.36000100
C	-4.96957800	3.15869900	-3.94965100
H	-5.38473500	1.92812600	-5.66832200
H	-4.34448800	4.14213800	-2.13013200
H	-5.56926200	3.99480600	-4.29711200
C	-3.14789600	-0.90596800	-1.37911000
C	-4.36058400	-0.55182800	-0.76933100
C	-2.47973700	-2.05416900	-0.90353100
C	-4.85976100	-1.29269700	0.30427700
H	-4.90614700	0.31637100	-1.11887200
C	-2.97030200	-2.78163300	0.17550700
H	-1.55116700	-2.35646500	-1.37079900
C	-4.16605700	-2.39967600	0.79012600
H	-5.79395500	-0.99042800	0.76881900
H	-2.40711200	-3.63427900	0.54144600
H	-4.54890500	-2.95772500	1.63884900
H	-0.67982200	-1.84371500	-3.64117400
O	1.62842500	0.04985300	-3.16513300
H	-1.23046800	0.62480500	-3.06556100
H	1.72624200	0.25767600	-2.21519700
O	0.25766900	-2.15442200	-3.61340200
H	0.25622800	-2.98048500	-3.10830200
H	1.29879600	-0.89362300	-3.22964300
O	-0.53245600	1.08691000	-3.88727000
H	-0.53711200	2.04937900	-3.73156600
H	0.44025500	0.72569700	-3.63705400
O	-2.27492800	-1.11408000	-3.60565700
H	-2.99287400	-1.77374100	-3.58474600

Int3-O[b]

Zero-point correction= 0.341828 (Hartree/Particle)
Thermal correction to Energy= 0.385570
Thermal correction to Enthalpy= 0.386514
Thermal correction to Gibbs Free Energy= 0.257060
Sum of electronic and zero-point Energies= -1617.425087
Sum of electronic and thermal Energies= -1617.381345
Sum of electronic and thermal Enthalpies= -1617.380401
Sum of electronic and thermal Free Energies= -1617.509855

Ag	0.63822200	1.16764100	0.36042400
N	-1.45547500	1.13281100	-0.34387500

N	-1.94712300	0.07674500	-1.06003700
N	0.75686200	-0.31341800	-1.79017100
N	0.03338200	-1.43757800	-1.49383000
N	-0.01804600	-1.45114900	1.31025600
N	-1.22325500	-1.56853600	0.68050000
B	-1.36245700	-1.34722600	-0.85236600
C	-2.19409500	2.19069000	-0.66060900
C	-3.18832900	1.85685000	-1.58740600
C	-2.98811200	0.49786600	-1.81011900
C	1.92619800	-0.73912300	-2.24833600
C	2.00396700	-2.14219900	-2.25501000
C	0.77015200	-2.53978800	-1.75883300
C	-0.27669000	-1.58452600	2.60230100
C	-1.64296800	-1.77960100	2.85932300
C	-2.20636300	-1.75881200	1.58973100
H	-2.10661300	-2.15091900	-1.31754400
Br	-3.98566200	-0.63786800	-2.95638700
Br	-4.51406800	2.98610700	-2.34847100
Br	-1.82960200	3.89399100	0.11844100
Br	-4.03823800	-1.91517700	1.10507900
Br	-2.52090500	-2.02784400	4.52897400
Br	1.14751000	-1.50447300	3.89193100
Br	0.13819800	-4.30390300	-1.43389500
Br	3.44703100	-3.24696400	-2.81907700
Br	3.26520100	0.50395300	-2.79286100
C	2.53949600	1.67805700	1.21749200
C	2.86849500	3.12883800	1.02995100
C	3.31374400	3.98031900	2.05409200
C	2.71460300	3.66745600	-0.26142900
C	3.56226600	5.33432700	1.80034700
H	3.48219000	3.58572200	3.05036700
C	2.99164400	5.00652800	-0.51636600
H	2.36689900	3.02198400	-1.06291000
C	3.40440600	5.85410600	0.51781800
H	3.89410000	5.97540100	2.61249000
H	2.86586400	5.39507800	-1.52293600
H	3.59899200	6.90425400	0.32222800
C	3.64837900	0.71647900	0.91620400
C	4.97453500	1.14659600	0.74909900
C	3.36249600	-0.65353200	0.77790500
C	5.98277600	0.23436400	0.44211400
H	5.21147000	2.20106200	0.84299700
C	4.37393500	-1.56732200	0.49299100
H	2.33694400	-0.99837600	0.86435400

C	5.68632600	-1.12442300	0.31647300
H	7.00154700	0.58463600	0.30204500
H	4.13112400	-2.61918000	0.37997700
H	6.47202300	-1.83377200	0.07277000
O	2.36820400	1.53995900	2.86541800
H	2.14297800	0.59716600	3.02921800
H	1.52732900	2.08744300	3.06770500
O	0.35037200	3.16973700	3.08624400
H	-0.29249100	2.86220200	2.42002800
H	0.81800200	3.90186800	2.64077200

TS2-O[b]

Zero-point correction=	0.336679 (Hartree/Particle)
Thermal correction to Energy=	0.380307
Thermal correction to Enthalpy=	0.381251
Thermal correction to Gibbs Free Energy=	0.249028
Sum of electronic and zero-point Energies=	-1617.395962
Sum of electronic and thermal Energies=	-1617.352335
Sum of electronic and thermal Enthalpies=	-1617.351390

Ag	-0.89517900	-1.08036800	0.08058000
N	1.18900500	-1.16043000	-0.78979900
N	1.85714700	0.00025800	-1.05871600
N	-0.86585100	0.86170600	-1.19982300
N	0.07349700	1.76279900	-0.76844800
N	0.26462300	0.80163400	1.88739600
N	1.43946100	0.98274300	1.21288700
B	1.47506200	1.29793100	-0.30302500
C	1.72391800	-2.10698400	-1.55093700
C	2.76698800	-1.58914000	-2.33281600
C	2.81064100	-0.24371700	-1.98525700
C	-1.88284700	1.57012400	-1.67847100
C	-1.65239500	2.94519800	-1.54292800
C	-0.39273000	3.01533300	-0.95803100
C	0.60985600	0.49198900	3.12979300
C	2.00221100	0.44965500	3.30209800
C	2.48733700	0.76858100	2.04113700
H	2.27939800	2.14045700	-0.53610600
Br	3.98982300	1.08459400	-2.65658500
Br	3.87540000	-2.51182700	-3.57160000
Br	1.03918700	-3.88176000	-1.51898300
Br	4.29452500	0.88339200	1.46322700
Br	2.99732300	0.06816300	4.87689100

Br	-0.72524200	0.19665800	4.47560400
Br	0.57825600	4.57097900	-0.46569500
Br	-2.79429100	4.38152100	-2.03767400
Br	-3.37196500	0.70894900	-2.48107400
C	-2.79319300	-1.70281900	1.14841200
C	-3.35757100	-2.93442600	0.48782300
C	-3.90980800	-3.97248000	1.24727900
C	-3.34437400	-3.05401800	-0.91026600
C	-4.41786800	-5.11556700	0.62118900
H	-3.95035300	-3.87871300	2.32638000
C	-3.86167600	-4.18529600	-1.53270300
H	-2.91602400	-2.25545900	-1.50988600
C	-4.39508300	-5.22835700	-0.76726100
H	-4.83751900	-5.91414700	1.22660700
H	-3.83777100	-4.25937500	-2.61609000
H	-4.78732900	-6.11722200	-1.25232000
C	-3.62329800	-0.44331500	0.95222000
C	-4.95596800	-0.52896900	0.52375100
C	-3.10792300	0.81683700	1.29862800
C	-5.74623700	0.61674000	0.43258600
H	-5.37610400	-1.49225600	0.25924500
C	-3.89702900	1.96082700	1.19789800
H	-2.07330700	0.91682100	1.61121100
C	-5.21987500	1.86587100	0.76225900
H	-6.77580800	0.53013200	0.09708700
H	-3.47026800	2.92742000	1.44867300
H	-5.83243200	2.75848000	0.67696400
O	-2.64645200	-1.95607000	2.54232200
H	-2.39457300	-1.12475800	2.98084600
H	-1.33783800	-3.26848800	2.58995100
O	-0.66571200	-3.72788900	2.03913000
H	-0.99282100	-2.42998400	0.95512100
H	-1.17101600	-4.41439000	1.57179100
